# Targeted Protein Modification with an Antibody-Based System

**DOI:** 10.1021/acscentsci.5c00651

**Published:** 2025-07-07

**Authors:** Oded Rimon, Juraj Konc, Inga Černauskienė, Montader Ali, Vaidehi Roy Chowdhury, Pietro Sormanni, Gonçalo J. L. Bernardes, Michele Vendruscolo

**Affiliations:** #Centre for Misfolding Diseases, §Yusuf Hamied Department of Chemistry, 2152 University of Cambridge, Cambridge, CB2 1EW, United Kingdom; ∥ Translational Chemical Biology Group, Spanish National Cancer Research Centre (CNIO), C/ Melchor Fernández Almagro, 3. 28029 Madrid, Spain

## Abstract

The chemical modification of proteins is one of the major mechanisms used to regulate the properties and functions of these macromolecules in the cell. It is therefore of great interest to develop tools to exploit this type of modification for applications in molecular biology, medicine, and biotechnology. Here we present a method of using antibodies to perform post-translational covalent modifications of endogenous proteins in complex environments by exploiting proximity-driven chemistry. The method is based on the ability of antibodies to hold a weakly reactive group close to its intended site of reaction by binding the target protein on a nearby epitope. We characterize this approach by modifying the green fluorescent protein in increasingly complex environments and illustrate its applicability by targeting the disease-associated protein beta-2 microglobulin.

## Graphical abstract

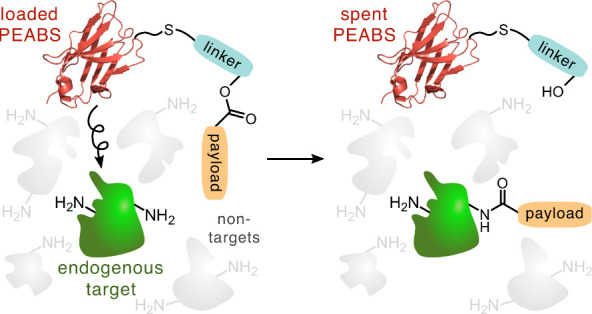

Chemically modifying a specific protein in a complex environment, such as that within a cell, is highly challenging but holds great promise. As well as being versatile labels for monitoring the behavior of proteins in their natural milieu, post-translational modifications (PTMs) can effectively influence protein structure and function.^1^ This phenomenon has long been exploited pharmacologically: covalent inhibitors such as aspirin bind, or attach a covalent payload, to the active sites of enzymes. Following this paradigm, rationally designed targeted covalent inhibitors (TCIs) adapt noncovalent ligands to react with nucleophilic residues adjacent to their binding site.^2^ However, disordered and misfolded proteins, as well as proteins that lack ligand binding sites, are largely inaccessible to both traditional drugs and TCIs.^3^ Such proteins are intimately involved in the mechanisms of diseases ranging from systemic amyloidosis^4^ to Alzheimer’s and Parkinson’s diseases^5^ and certain cancers.^6^ Notably, however, the very reasons that make these proteins difficult to target with traditional small-molecule drugs make them near-ideal candidates for modulation via PTMs.^7^

It is therefore important to develop technologies for targeted post-translational modification (TPTM)—the covalent modification of endogenous proteins in complex environments at a specific surface-exposed site—for these proteins. Recent years have seen tremendous advances in TPTM tools for ligandable targets. Noncovalent ligands and substrates have been converted into covalent inhibitors.^2^ They have been used as bases for larger molecules that recruit naturally available PTM and proteolysis machinery to proteins of interest (POIs), such as proteolysis targeting chimaeras (PROTACs),^8^ lysosome-targeting chimaeras (LYTACs)^9^ and other proximity agents.^10^ They have been fused to exogenous PTM inducers to label all proteins in the vicinity of a POI (proximity labeling),^11^ and they have been redesigned to carry modifying groups and transfer them directly to a POI (ligand-directed chemistry).^12^ Applications of covalent protein modification for drug design and development have recently been reviewed.^13^ In parallel, tools for binding and manipulating undruggable proteins have become more sophisticated and common. From novel methods for generating antibodies against challenging targets^14^ through protein binders of various alternative scaffolds including cyclic peptides,^15,16^ to the utilization of these binders for proximity labeling the environment of a POI,^17^ the range of tools for characterizing and manipulating nonligandable targets is constantly expanding. Combining the two emerging fields to create a technology for directly transferring a modification to a nonligandable protein could pave a promising new path for developing drugs against challenging targets and disease mechanisms.

To be therapeutically relevant, such technology would need to be stable in circulation, successfully make its way to—and then engage—an endogenous, nonligandable target, and react with it with target- and site-selectivity, leaving no trace on the target apart from the desired PTM, with the rest of the molecule effectively cleared from the system.

In more details, we consider two sets of requirements. At the chemical level, a TPTM agent for an undruggble target should: (i) Engage endogenous proteins, which are genetically and chemically unmodified; (ii) Bind chosen sites, without relying on proximity to naturally occurring binding sites on the target, as these are either not available for the target or are not positioned nearby residues of interest for PTM; (iii) Be traceless,^18^ i.e., break up, with the noncovalent binding moiety dissociating and only leaving behind a covalent payload^19^ (traceless reactions uncouple the reactivity and selectivity of the reaction from the identity of the covalent payload, uncovering a vast chemical derivatization landscape and leaving no junk atoms behind that may cause haptenization and other unpredictable behaviors^2^); (iv) Be selective for the target, in order to minimize adverse effects due to nonspecific modification of other macromolecules and small-molecule nucleophiles; and (v) Be site-selective, to reduce the immunogenicity of the modified product and produce consistent and uniform effects.^20^ At the pharmacological level, it should also: (vi) Remain stable in plasma in the presence of nontarget proteins and other molecules, retaining the covalent payload until it can be transferred to the desired target; (vii) Be able to penetrate the tissues or cells containing the target protein; and (viii) Possess favorable pharmacokinetic properties, including an appropriate elimination half-life (long enough to reach the target and enable payload transfer, yet short enough to effectively eliminate the spent molecule after it has transferred its payload).

To our knowledge, no technique or compound exists at this time that fulfils all the requirements listed above. Here, we present an approach for developing such tools. While keeping the pharmacological requirements in mind, we focus here on meeting as many of the chemical and biochemical requirements with our approach. First, we establish the approach and demonstrate its potential and limitations by targeting the green fluorescent protein (GFP) in environments of increasing complexity. We then apply the lessons learned from this model system to modify the disease-associated protein beta-2 microglobulin (β2m) in complex mixtures. We believe that developing tools that fulfill the chemical requirements for TPTM of nonligandable disease-associated proteins would be an important first step toward modulating the behavior of these proteins *in vivo*.

The targeted post-translational modifiers that we introduce in this work are, in essence, hybrid molecules consisting of a high-affinity antibody and a synthetic linker featuring a weakly reactive group susceptible to induction through the proximity effect.

## PEABS: An Antibody-Based Design for Targeted Post-translational Modification

A key feature of TPTM is selectivity. In complex biological environments, no chemical functionality can be expected to be unique to one macromolecule, and reactive chemical groups nonspecifically modify almost inevitably off-target proteins at least to some extent. Therefore, weak reactivity is required for any ligand-directed chemistry, warhead for TCI or reactive group for TPTM. On the other hand, to accomplish its task, the reactive moiety of any covalent drug must act during the period of time that its noncovalent moiety is bound, i.e. within its residence time.^2^

A way to bridge these two seemingly contradictory requirements is through the exploitation of the proximity effect. Noncovalent binding of a covalent drug to a macromolecule reduces its translational and rotational entropy. As a result, the activation energy for its covalent reaction with a nearby functional group loses a portion of its entropic penalty and is therefore decreased. This effect can account for more than a 3 orders of magnitude acceleration in reaction rates.^21^

To achieve the long residence times required for TPTM, one can increase the interaction surface area. This is often challenging with small-molecule drugs, but less so with biological macromolecules – and particularly antibodies.^22^ Antibodies bind to unmodified endogenous proteins, fulfilling the first requirement of TPTM. With methods ranging from immunization to *in silico* rational design, antibodies can be developed to target any unique feature on the surface of a target protein, with high affinity and selectivity.^14^ This fulfils the second requirement for TPTM, allowing one to create a TPTM agent for a desired target residue, rather than choosing the target residue based on its proximity to a known, well-structured binding pocket.

Thanks to the versatility of antibodies, the choice of a target epitope can be made with additional considerations in mind. The first of these would be the choice of a naturally occurring nucleophile on the target protein to be used for substitution with the payload. While cysteine is a common choice in TCI design due to its high nucleophilicity and low abundance, targeting it would limit the range to surface-exposed, free-form cysteine residues. Primary amines, on the other hand, are common on the surfaces of proteins. As well as the N-terminal amine (on nonacetylated proteins), lysine residues constitute 6% of the human proteome, and they are quite nucleophilic despite being protonated at physiological pH,^23^ with some 9000 lysine residues estimated as available for nucleophilic attack in human proteomes.^24^

With these observations in mind, we opted for a design in which an antibody is produced recombinantly with an engineered cysteine residue at a carefully selected position (see below). A synthetic linker is then conjugated to the engineered cysteine *in vitro* to form the active hybrid species. Upon the binding of this species to its target antigen, its activated ester moiety is positioned precisely to transfer the payload on to one amine group of the target, forming an amide link between them and releasing the spent antibody, which can then be cleared without loss of the PTM (Figure 1a).

**Figure 1:**
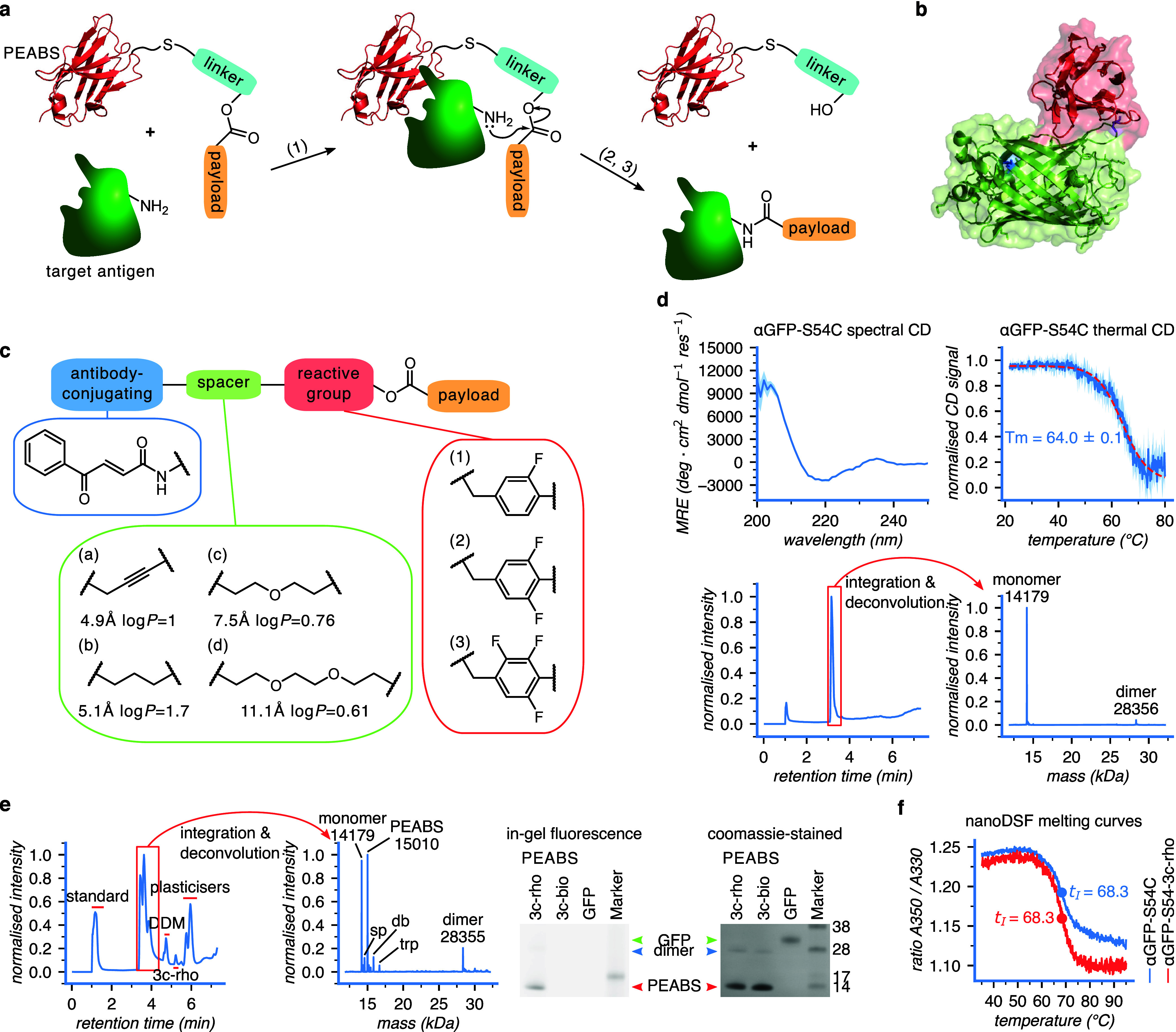
Design of a protein-editing antibody-based system (PEABS). (a) General scheme of a PEABS reaction. A linker featuring an amine-reactive group is bioconjugated to an antibody prior to the reaction; the noncovalent binding of antibody and antigen brings the reactive group in proximity to a primary amine group of the target (1); a nucleophilic attack transfers the payload onto the target, with the linker leaving (2); the dissociation of the spent PEABS leaves no trace on the target except the (iso)­peptide-bound payload (3). (b) Structure of the antibody–antigen pair used in this work, which consists of GFP (green, with lysine 41 in blue) and anti-GFP nanobody (red with residue 54, chosen for linker conjugation, in purple) [PDB: 3OGO].^25^ (c) Schematic structure of a loaded PEABS linker. Linkers are named ***#l*** according to their reactive group number (#) and spacer letter (*l*), e.g., **2b**. A benzoylacrylic-based Michael acceptor (blue) is responsible for conjugation with the engineered cysteine on the antibody. Spacers (green) connect it to a reactive group; values under each spacer indicate its fully extended length and partition coefficient as predicted by ChemDraw. Reactive groups (red) are responsible for carrying the payload and transferring it tracelessly to the target; different fluorophenols were employed for this purpose. (d) Characterization of αGFP-S54C, the cysteine mutant used as a basis for PEABS systems, using circular dichroism (CD), heat-induced denaturation monitored by CD, and intact protein LC-MS. (e) Confirmation of bioconjugation of **3c-rho** to αGFP-S54C using LC-MS and SDS-PAGE; >40% of the nanobody was converted, with very low residual free linker. αGFP-S54C *exp.* 14179 *obs.* 14179; PEABS *exp.* 15010 *obs.* 15010; sp (spent PEABS) *exp.* 14586 *obs.* 14586; dbl (PEABS + **3c-rho**) *exp.* 15841 *obs.* 15842; trp (PEABS + 2 × **3c-rho**) *exp.* 16673 *obs.* 16676; DDM dodecyl maltoside. (f) nanoDSF profiles confirm minimal destabilization of the nanobody structure following bioconjugation.

Referring to its ability to post-translationally modify proteins, as well as extending them by forming new peptide bonds at the N-terminus, we termed this design the Protein-Editing Antibody-Based System, or *PEABS*.

## Results

### Design and Characterization of GFP-Modifying PEABS

For the noncovalent binding moiety of our designs, we chose single-domain antibodies (sdAbs), which are heavy-chain-only, functional antibody fragments that weigh 12–15 kDa, lack the constant region (*fragment crystallizable region*, or Fc) that facilitates the immune response, and feature stability, solubility and expressibility in bacteria and yeast.^26,27^ Despite the small size of sdAbs, they can be highly specific and have low-nanomolar and even picomolar affinity for their targets. sdAbs have been used for various therapeutic applications, from treatment of infectious diseases^28^ to cancer and autoimmune conditions.^29^ In 2018, the thrombotic thrombocytopenic purpura drug Caplacizumab was the first sdAb to be approved as a drug.^30^ One sdAb utilized herein, nanobody 24 (nb24), has been shown to modify the tissue distribution of its target, β2m, in mice,^31^ showing promise for therapeutic utilization. Although its pharmacokinetics prior to target binding have not yet been studied, the metabolic stability of other nanobodies has been shown to be modifiable by relatively simple measures^29^ and improving their tissue and cell-permeability is an active and increasingly successful area of research.^32^ Due to their ability to bind less-accessible epitopes, their high affinity, their small size contributing to improved tissue penetration, and their low immunogenicity on injection into bloodstream (at least when humanized),^29^ we anticipate that nanobodies and sdAbs in general will become increasingly used for diagnostics and therapeutics. We have therefore chosen to base our design on this scaffold.

We began with the ∼1 nM binding, llama-derived αGFP nanobody (also referred to as GFP-enhancer), reportedly discovered and crystallized independently by Kirchhofer et al.^33^ and Kubala et al.^25^ Serine 54 was then chosen as the site of mutation to cysteine and subsequent bioconjugation to the synthetic linker, based on the minimal impact of serine to cysteine mutations, its low conservation score in multiple sequence alignment of sdAbs, and its position in the bound structure (Figure 1b).

On the linker side, a three-part modular design was envisaged. On one end, we included a cysteine-selective, strong electrophile, to enable its *in vitro* bioconjugation to the antibody. On the other end, we placed the payload to be carried on a weakly reactive group. This payload—on proximity with a primary amine—is meant to undergo nucleophilic substitution, transferring the payload to the target. Between the electrophile and the payload, a spacer domain was inserted to determine the reach, flexibility and interaction profile of the linker, thereby affecting the site-selectivity of the reaction and its rate by varying the level of entropic restriction (Figure 1c).

Cysteine-selective, fast-reacting benzoyl acrylate derivatives proved highly suitable for the first purpose.^34^ Spacers ranging 4.9–11.1 Å in length, corresponding to full linker lengths of about 12–18 Å (Figure S12) and varying in hydrophobicity (with predicted partition coefficients in the 0.6–1.7 range) were explored.

For the amine-reactive chemistry, the range of options can be divided into four groups: substitution at carbon, substitution at sulfur, direct addition on carbonyl carbon, and conjugate addition, all varying in chemoselectivity to primary amines, stability in aqueous environments, uninduced and proximity-induced reaction rates, and the reversibility and tracelessness of the conjugation reaction.^35^ Here, we chose substitution at carbon and used phenols with varying levels of fluorination, covering a range of reaction rates while providing protection from hydrolysis.^36^

Finally, as the only part of our compound to stay behind on the target, the payload is in essence the PTM itself. To enable various techniques of monitoring the reaction and characterizing its products, the payloads we chose were biotin, a vitamin and commonly used affinity tag (*bio*), and rhodamine B, a water-soluble and photostable fluorescent dye (*rho*). Later in the work, we explored other payloads as well (Figure 5d).

Medium-length, trifluorophenol-based linkers loaded with rhodamine B (**3c-rho**) or biotin (**3c-bio**) were synthesized, and a gene for the αGFP nanobody was cloned into a bacterial expression vector with and without introducing an S54C mutation. αGFP and αGFP-S54C were expressed and purified, and prior to conjugation of any linkers, the fold and thermal stability of the mutant were assessed using circular dichroism (CD) (Figure 1d). These spectra and melting temperatures were consistent with correctly folded, active sdAbs.^37^ The mass and purity of the cysteine mutant were also confirmed by SDS-PAGE and LCMS (Figure 1d). Binding assays using surface plasmon resonance (SPR) confirmed binding of the cysteine mutant to GFP, with a 25-fold reduced affinity compared to the wild type, but still well within the high-picomolar/low-nanomolar range (Table S1).

To form the PEABS molecule αGFP-S54-3c-rho, αGFP-S54C was reduced, desalted and mixed with 10 equiv of **3c-rho**. After a 50 min incubation at 25 °C, the formed PEABS molecule was purified by desalting into the reaction buffer (40 mM potassium phosphate and 40 mM NaCl, pH 7.2). Intact protein LC-MS was used to validate the formation and purity of PEABS (Figure 1e). Peaks in the chromatogram were first classified into broad categories (e.g., nanobody derivatives, surfactants, free linker species) by manual inspection. The peaks corresponding to nanobody derivatives were then marginalised over retention time to produce a spectrum, which was deconvoluted using the UniDec software^38^ to afford the final mass/intensity spectrum. SDS-PAGE analysis comparing in-gel fluorescence of rhodamine B (prior to staining) and Coomassie-stained total protein further confirmed the conjugation of the antibody and the small-molecule linker (Figure 1e). In this system, we achieved partial conversion of the nanobody to PEABS (40–50% of the nanobody population). This was improved upon in subsequent iterations (Figure 5b).

Finally, we compared the stabilities of the fully formed PEABS molecules to those of the antibody they are based on. Nanodifferential scanning fluorimetry (nanoDSF) was used to establish its unfolding inflection temperature. This method gave comparable results for the unconjugated αGFP-S54C and αGFP-S54-3c-rho, suggesting that the stability is unaffected by linker conjugation (Figure 1f). Furthermore, conjugation of a PEABS linker without a payload (giving spent PEABS, the nanobody left over after a PEABS reaction) had no measurable effect on the affinity of the nanobody to its target. Critically, its complex with the target dissociates at a similar rate (Table S1), conferring a degree of tracelessness to the system.

Concerned by the potential of PEABS to self-acylate, either in *cis* (intramolecularly) or *trans* (from one PEABS molecule to another), we monitored the mass of PEABS over time with no other components in solution. As *cis* self-acylation results in the same mass as PEABS, its product could not be directly measured. However, gradual loss of the payload over time, at any temperature, reflected in increasing population of spent PEABS, demonstrated that there is no significant level of *cis* self-acylation; and lack of a PEABS + payload mass suggests a low likelihood for *trans* self-acylation as well (Figure S2a,b). The rate of hydrolysis is temperature-dependent (Figure S2a) and pH-dependent (Figure S2b). As a whole, these results may point toward potential issues with long-term storage of active PEABS molecules, but suggest they are stable enough to be used in complex environments to modify targets without considerable self-reactivity.

### PEABS Post-translationally Modifies Its Target at a Single Position

Several methods to monitor the reaction between the PEABS and its target were explored. Many, including following the fluorescence polarization of rhodamine B on its transfer and using in-gel fluorescence SDS-PAGE, presented difficulties in detecting any changes, mostly due to high background signals from minute residual amounts of rhodamine B in solution (released from slow hydrolysis of PEABS at the reactive group or present in the linker preparation). Serving as the gold standard for the analysis of protein PTMs,^39^ mass spectrometry–based methods were thus sought.

Intact-protein LC-MS may not be strictly quantitative when comparing different species, as they may be ionized to different degrees and fly differently. However, when comparing very similar species, such as unmodified nanobody vs PEABS or GFP vs rhodamine-conjugated GFP (GFP-rho), this technique proved to be highly informative. To obtain the most quantitative results, ion series corresponding to the expected masses of species were identified, and extracted ion chromatograms (XICs) for each such series were isolated from the chromatogram. Subsequently, the time segment containing the peaks corresponding to all species in a set (e.g., all GFP species) was identified, and the integrated intensity over that segment was used as a surrogate for its quantity (Figure 2a). As expected, the integrals of very dissimilar species, such as nanobody and GFP, do not accurately capture their relative concentrations in solution. Therefore, each species was normalized to the sum of its own group of species (Figure 2a). This proved useful for monitoring reaction progress in a quantitative way (see below).

With this tool in hand, we set out to examine the reaction between PEABS and its target. αGFP-S54-3c-rho was prepared as above, and its concentration estimated by its fraction of total nanobody species and the known starting nanobody concentration. Then, GFP, α-synuclein, BSA or all three were added at an approximate 1:1 molar ratio relative to the initial nanobody concentration. After 5 h at 25 °C, samples were analyzed by LC-MS. GFP was modified to 27 ± 1% when alone in solution, and its level of modification increased to 38 ± 3% in the presence of α-synuclein or BSA; the latter two proteins were unmodified by PEABS, even if they were the only proteins in solution (Figure 2b).

To further validate that the antibody determines target-selectivity, free **3c-rho** linker or the small-molecule conjugate **TCEP-3c-rho** were incubated with GFP, or with the three-protein mixture. GFP alone was very minimally modified by free linkers (4.5 ± 2.5%), and in a mixture was not modified. This is in contrast to the difference observed with PEABS, indicating that GFP does not have a unique property causing its modification, and it is truly brought about by the proximity effect conferred by the antibody.

To better understand the payload transfer reaction, we monitored its kinetics. PEABS was prepared as before, and GFP added at a 1:1 molar ratio relative to PEABS, taking the 40–50% conversion from nanobody into account. The mixture was incubated at 18 °C and samples were taken at six time-points ranging 5–1200 min after GFP addition and subjected to intact protein LC-MS (Figure 2c). A pattern of emergence of rhodamine-conjugated GFP was observed, and could be quantified by integrating extracted ion chromatograms and calculating ratios, as described above (Figure 2d). A rapidly increasing population of GFP-rho, with double-modified GFP-2 × rho only appearing in very small amounts and at a much slower rate, indicated selectivity toward a specific amine of the GFP target. It is worth noting that these observed reaction rates were measured in the presence of a significant population of unmodified nanobody (Figure 1e), which competes with PEABS for binding. It is likely that the underlying rates of reaction for pure PEABS are in fact higher, as suggested by our later results (see below).

We employed a two-step process to identify the preferred site of modification by PEABS. First, GFP modified using PEABS was isolated by SDS-PAGE and subjected to chymotrypsin digestion followed by LC-MS/MS analysis (Cambridge Centre for Proteomics). Such analysis is sensitive to low levels of modified peptides, and may flag sites of low-abundance and nonspecific modification. On the other hand, it does not cover the entire length of the protein, and may therefore miss sites of modification even if they are abundant. Therefore, we used it for preliminary identification of candidates for further investigation. Sites that were covered by the analysis but did not show the modification were ruled out; sites that appeared modified in either GFP-rho or GFP-bio but not both were deemed low-likelihood candidates; sites that appeared in both were deemed high-likelihood candidates; and sites that were not covered had to be investigated further to be decided. We found four low-likelihood sites, identified in GFP-bio but not GFP-rho, and no high-likelihood candidates (Figure 2e). These four sites, along with four sites that were outside the covered regions, and four additional sites that were suspected for structural reasons, were chosen for further investigation (Figure 2e). In the second step, the suspected lysine residues were mutated out one by one, and modification by αGFP-S54-3c-rho was monitored. Most mutations had very minor or no effect on modification by PEABS, except for K41R, which reduced the modification rate as well as the final modification level to a fraction of their normal values, indicating that K41 is the preferred site of modification by αGFP-S54-3c-rho (Figure 2f). We note that the LC-MS peak for the modified GFP-K41-rho is shifted compared to unmodified GFP, whereas the nonspecific modification does not shift the peak (Figure S8). This helped us verify that the majority of modification in wild type GFP is indeed at K41 (Figure 2c).

### PEABS Is Selective toward Its Target

After observing an apparent specificity of PEABS to its target GFP in a simple mixture of three proteins (Figure 2b), we measured its target-selectivity in a complex mixture of cellular components. For this purpose, a lysate of BL21­(DE3) bacteria was prepared and its concentration measured using the Bradford assay. 1.25 μg of GFP alone, 125 μg of lysate, or a mixture of the two were combined with αGFP-S54-3c-bio at a 1:1 molar equivalence to GFP, or an equivalent volume of buffer. The six mixtures were incubated at 25 °C for 18 h. Samples of each reaction were then run on two identical SDS-PAGE gels in parallel, in the same gel box. One gel was developed using standard Coomassie stain, while the other was blotted and developed using streptavidin–Alexa Fluor 647, to reveal biotinylation patterns.

Remarkably, GFP was selectively modified even when it constituted only 1% of the solution by weight, to about the same extent as when it is on its own (Figure 3a). In fact, GFP alone accounted for nearly 80% of the biotinylation in solution, with only a fraction distributed among the other 99% (Figure 3b). Assuming a uniform distribution of modifiable residues, we can approximate the selectivity ratio for on-target GFP activity as $\frac{79\%}{21\%}\times \frac{99\%}{1\%}\approx$ 380:1. It is likely that even this low level of nonspecific reactivity can be attributed to some nonspecific binding of the antibody rather than nonspecific reactivity of the PEABS reactive group. These findings indicate high target-selectivity, an important feature of TPTM agents.

### Characterization of the Targeted Post-translational Modification Reaction

During experimentation with **3c-rho** and **3c-bio**, we noticed differences in the level of modification that they caused at the tested time points. To verify that these differences arose from slight differences in reactivity, we measured the TPTM kinetics on pure GFP at a 1:1 molar ratio. We found that rhodamine B is transferred at a slightly higher rate than biotin (Figure 4a). This minor but noticeable effect from the payload may be an important consideration in the design of future implementations.

Based on the principles of the PEABS design and our results with trifluorophenol-based agents, we thought that reactivity and selectivity could also be fine-tuned by varying the spacers and electrophiles of the PEABS linkers. To investigate this possibility, we synthesized four more linkers carrying rhodamine B: a longer, more hydrophilic trifluorophenol-based linker (**3d-rho**) and three shorter, less hydrophilic and presumably less reactive monofluorophenol- and difluorophenol-based linkers (**1a-rho**, **2a-rho** and **2b-rho**).

The reaction rates of the linkers are clearly affected by the hydrophilicity of the spacer, with **1a-rho** and **2b-rho** displaying identical reaction rates despite the more reactive difluorophenol in **2b-rho**, and **3d-rho** reacting faster than **3c-rho** despite the identical reactive group (Figure 4a–b). No nonspecific reactivity toward α-synuclein or BSA was observed with any of the linkers. However, double modification was observed to different extents in all. We used this is an indirect measure of site nonspecificity. Here, the level of nonspecificity seems to follow mostly the intrinsic reactivity of the electrophile (Figure 4c).

Combining these results, we expect combinations of longer, more hydrophilic spacers, with reactive groups of lower intrinsic reactivity, to be ideal for future implementations of the PEABS approach.

Next, we wanted to examine the role of affinity in the rate of the TPTM reaction. For this purpose, we designed four mutants of the αGFP-S54C nanobody that should decrease its affinity toward GFP without negatively affecting its stability. PEABS formation proceeded similarly across this panel of mutant (Figure S9a). It was interesting to find that PEABS systems based on several of these mutants had significantly improved reactivity compared to the original (Figure 4d). In the mutants tested, this effect seemed to correlate with increased off-rates (Table S1). We postulate that there is an ideal window of affinity for a PEABS nanobody, where the competition between active PEABS and inactive nanobodies (e.g., unconjugated nanobodies or hydrolyzed PEABS) will be impeded if their affinity is too high, while insufficient affinity would not allow the reaction to occur. This would be an important consideration in future PEABS designs.

### General Applicability of the PEABS Approach

Having characterized the key features of the PEABS system and their effects on its TPTM reaction, we set out to test its applicability to different targets.

β2m is a major histocompatibility complex (MHC) I component involved in several renal diseases.^41^ It is known to accumulate and form amyloids in dialysis patients, a condition known as dialysis-related amyloidosis.^42^ It may also play a role in the progression of multiple myeloma.^6^ Nanobodies against this 11.9 kDa protein had been generated, and several had been shown to successfully inhibit its aggregation.^43^ Based on our results with αGFP mutants, we chose to base β2m PEABS on the midnanomolar–affinity camel-derived nb24,^43^ whose bound structure to β2m had been solved^44^ (Figure 5a).

Based on the bound structure, we chose residue D113 to be mutated to cysteine and used to bind PEABS linkers. Mutating this residue did not compromise stability or binding to β2m (Table S1 and Figure S10a). The same linkers used for GFP PEABS were repurposed, and formation of the PEABS molecule followed the same procedure (Figure 5b). The mass spectrometry-based technique that we developed for GFP PEABS proved effective for β2m PEABS as well (Figure 5c). Monitoring the reaction of nb24-D113–3c-bio with recombinant β2m at 25 °C revealed effective modification, with little to no double modification, indicating a site-selective TPTM reaction (Figure 5d). However, isolation of β2m species by SDS-PAGE followed by chymotrypsin digestion-LC-MS/MS (Cambridge Centre for Proteomics) did not confidently identify the site of modification. It did narrow the options down to four, three of which are supported by structural analysis (Figures S10c and 5a). As more PEABS systems are developed on the basis of diverse sdAbs against diverse targets, the rules governing the predominant site of modification will likely be revealed and hopefully allow for the rational design of systems to modify specific residues of interest for therapeutic purposes.

Curiously, the two payloads previously tested, rhodamine B and biotin, had the inverse effect on the reaction rate toward β2m compared to GFP (Figure 5d, top panels; compare Figure 4a). We hypothesize that this may be affected by the more negative charge of GFP (pI 5.4) compared to β2m (pI 6.0) (Supplementary Methods), and by the local environment of the modified lysine side-chain.

These results suggest a potential influence of the payload, its bulkiness and its charge on the reaction rate. We wanted to test this, and to also take another step toward applicability by attempting a PTM of known biological relevance. To this end, we tried to use PEABS to directly phosphorylate β2m. Linker **3c** was loaded with a phosphate group (**3c-pho**), and PEABS nb24-D113–3c-pho was constructed and incubated with β2m. Despite PEABS forming successfully and efficiently (Figure S2c), even at the 20 h time point the level of phosphorylated β2m could not be distinguished from noise (Figure 5d, bottom-left). Moreover, observing the phosphorylating PEABS construct in solution demonstrated excessive stability, with minimal loss of the phosphate group over 48 h, in contrast to active PEABS constructs (Figure S2c). A sulfate-carrying PEABS linker was synthesized as well, but in contrast to **3c-pho**, was not stable enough to sustain even the mildly conditions needed to purify it (not shown). Given this instability and the excessive stability of **3c-pho** PEABS, we chose to use a close analogue instead. Like phosphate and sulfate groups, 4-sulphobenzoic acid carries a negative charge, and is known to improve the water solubility of molecules to which it is attached.^45^ Modifying lysine residues this way may be used as a mimetic to study some of the properties of the otherwise elusive phospholysine,^46^ or more directly for inverting the charge of the lysine residue to change the biophysical properties of the target protein. To achieve this modification, linker **3c** was loaded with 4-sulphobenzoate (**3c-sb**) and used to form PEABS, which successfully transferred it to β2m at a comparable rate to biotin modification (Figure 5d, bottom right). These results suggest that carboxylic acids, both alkanoic and aromatic, can be used as PEABS payloads, and that the system supports various payloads of neutral, positive and negative charge. Further studies are required to determine the full range of modifications that can be effected by PEABS.

So far, we demonstrated the utility of our method on recombinant proteins at relatively high concentrations. We wanted to examine whether β2m could be modified at its endogenous concentration as well. Serum levels of β2m exceeding 4 μg/mL (0.33 μM) have been shown to correlate with poorer health outcomes.^47^ We used this cutoff concentration to test our system at more physiological conditions. While the lower concentration reaction proceeded at a slightly lower rate than our standard 1.5 μM reaction and reached a lower level at the 20 h mark (39% vs 52%), it was successful in modifying the target (Figure 5e), warranting cautious optimism about its potential use in human serum.

Finally, target-selectivity in a complex mixture was measured using **3c-bio**, giving an approximate selectivity ratio of 70:1 (Figure 5f–g). This value is lower than that of GFP PEABS, most likely due to lower specificity of the nanobody employed, but is still indicative of a highly effective TPTM agent formed against this disease-causing target. Similar activity was observed when 3 mg/mL HeLa cell lysate in radioimmuno­precipitation assay (RIPA) buffer was spiked with 0.5 μM β2m and treated with 1 μM PEABS (Figure 5h). Despite the high concentration of free amines—including Tris from the buffer—as well as the diverse molecular contents of a mammalian cells, exogenous β2m was modified to completion, producing a signal indistinguishable from preformed 0.5 μM β2m-bio (Figure 5h, red arrow). Strikingly, despite its low natural abundance, endogenous β2m in the HeLa cell lysate could also be directly modified and detected, albeit with limited specificity and signal strengths in the concentrations of lysate and PEABS used (Figure 5h and S11). This points to some of the optimizations needed to bring PEABS to *in vivo* applications and eventually into the clinic, while also showing great promise for its versatility, ability to work in a wide range of conditions, and applicability to diverse biological systems.

## Discussion and Conclusions

In this work, we considered the set of requirements that a system for targeted post-translational modification (TPTM) of nonligandable proteins would need to fulfill to become a viable tool for addressing problems in the complex environment of a living organism at the protein level. These requirements include having an endogenous target, binding pocket independence, tracelessness, target selectivity and site selectivity, as well as pharmacological requirements such as plasma stability, tissue penetration, and favorable pharmacokinetics.

Toward meeting these conditions, we have reported PEABS, an antibody-based system for chemically modifying proteins, and illustrated its use by targeting two distinct proteins, GFP and β2m. Targeting endogenous proteins and being independent of ligand binding pockets is built into the design by using antibodies for their noncovalent affinity and specificity. Tracelessness is also built into the design, by employing substitution-at-carbon electrophiles—here, fluorophenols—for the reactive group. Target selectivity was shown first in pure protein solutions and simple mixtures, where LC-MS analysis could determine explicitly that GFP PEABS only modifies GFP and not α-synuclein or BSA (Figure 2c). Then, target selectivity was further monitored in a lysate, where the majority of a biotin payload was transferred to the desired target, despite it accounting for only 1% of the total protein mass (Figures 3 and 5f–h). Both systems featured insignificant double-modification, suggesting selectivity to a specific site on the target. In GFP, we showed that this site is lysine 41. It is not immediately obvious from the antibody–GFP structure exactly what factors determined this site of modification by PEABS (Figure 1b). As more PEABS systems are designed and tested or simulated, especially targeting the same protein but utilizing sdAbs that bind to different surfaces, we expect to learn more and improve our ability to design systems targeting specific residues of interest.

**Figure 2:**
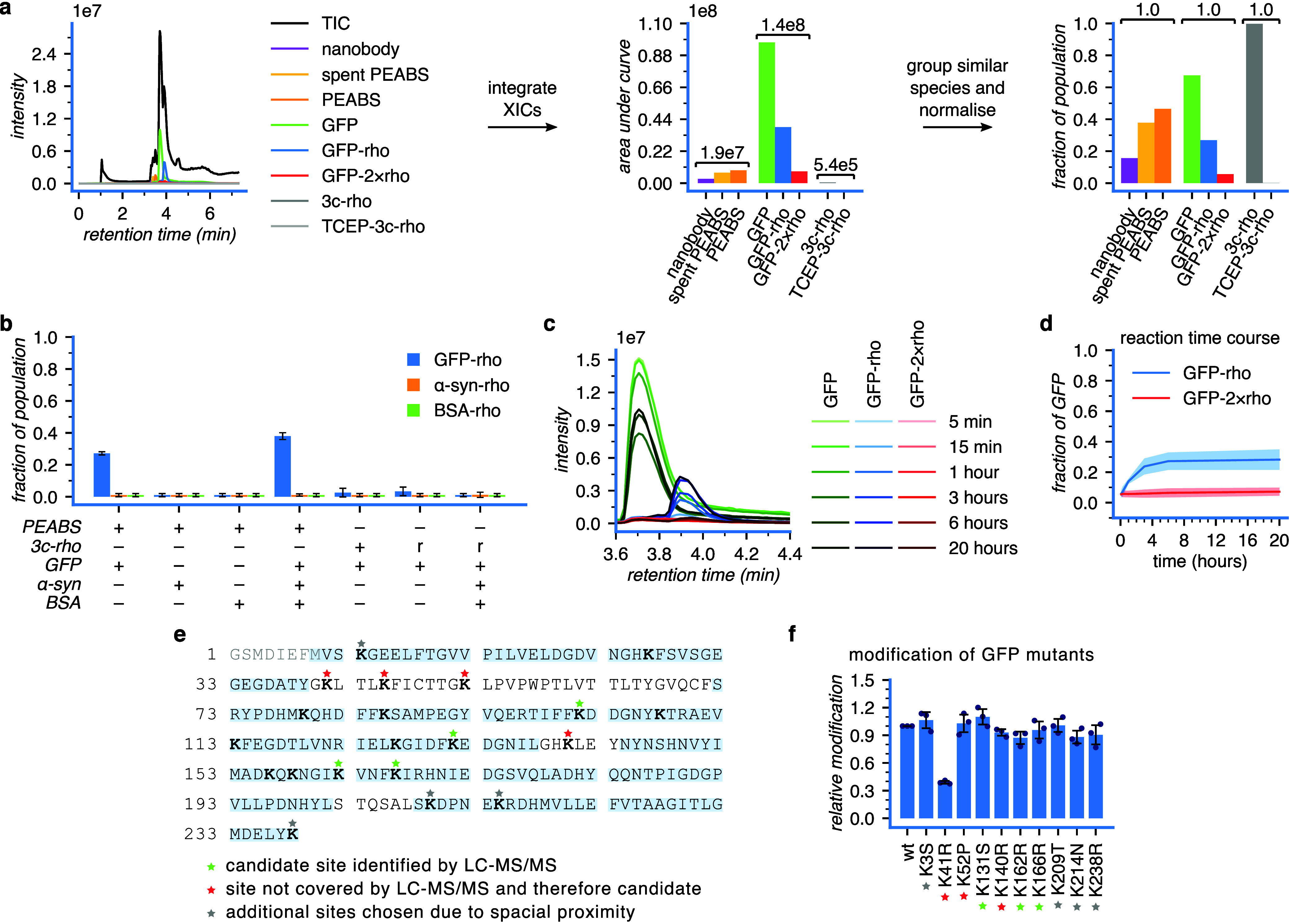
αGFP PEABS post-translationally modifies GFP in a target-selective and site-selective manner. αGFP-S54-3c-rho was used in all cases shown. (a) Representative example of an intact-protein LC-MS chromatogram and its extracted ion chromatograms corresponding to the experimentally relevant species in solution. Analysis of the abundance of different species in solution based on summation of signals allows semiquantitative comparison of similar species (e.g., GFP vs GFP-rho), but not of dissimilar ones (e.g., GFP vs nanobody). (b) For each experimental condition in *N* = 3 independent experiments, semiquantitative analysis as in panel (a) was used to derive the relative populations of similar species out of the total population of that species group. In solution, PEABS transfers the rhodamine B payload to GFP, but not to α-synuclein or to BSA and finds its target in a mixture of the three. Free linkers, whether in their benzoylacrylic form (*+*) or bound to TCEP (*r*), only modify GFP to a very small extent and not at all when other proteins are present. Values under 1% are within the uncertainty of measurements and are therefore represented as 1 ± 1%. (c) A representative example of intact-protein LC-MS monitoring of the rhodamine payload transfer reaction at 18 °C. (d) Kinetics of GFP modification as derived from *N* = 3 independent experiments; see panel (c), Figure S3 and Table S2. Shaded area represents 95% confidence intervals. Single-site modification is significantly preferred to multiple-site modification, suggesting selectivity for a specific site. **TIC** total ion count; **XIC** extracted ion count. (e) GFP modified by αGFP-S54-3c-bio was digested with chymotrypsin and analyzed by LC-MS/MS. Regions covered by the analysis are highlighted blue. Four sites were identified as potential candidates for the modification site (green stars), and four more sites were in regions not covered by the analysis. Four additional sites were chosen for further inspection due to their proximity to residue S54C of the nanobody in the bound structure. (f) The 12 candidate residues identified in the analysis were systematically mutated out and subjected to modification by αGFP-S54-3c-rho. Data at the 3 h time point reveal a clear preference to modification at residue 41. GFP-K101R and GFP-K45R could not be produced. Means represent *N* = 3 independent experiments, and error bars standard deviations; full data in Figure S8.

**Figure 3:**
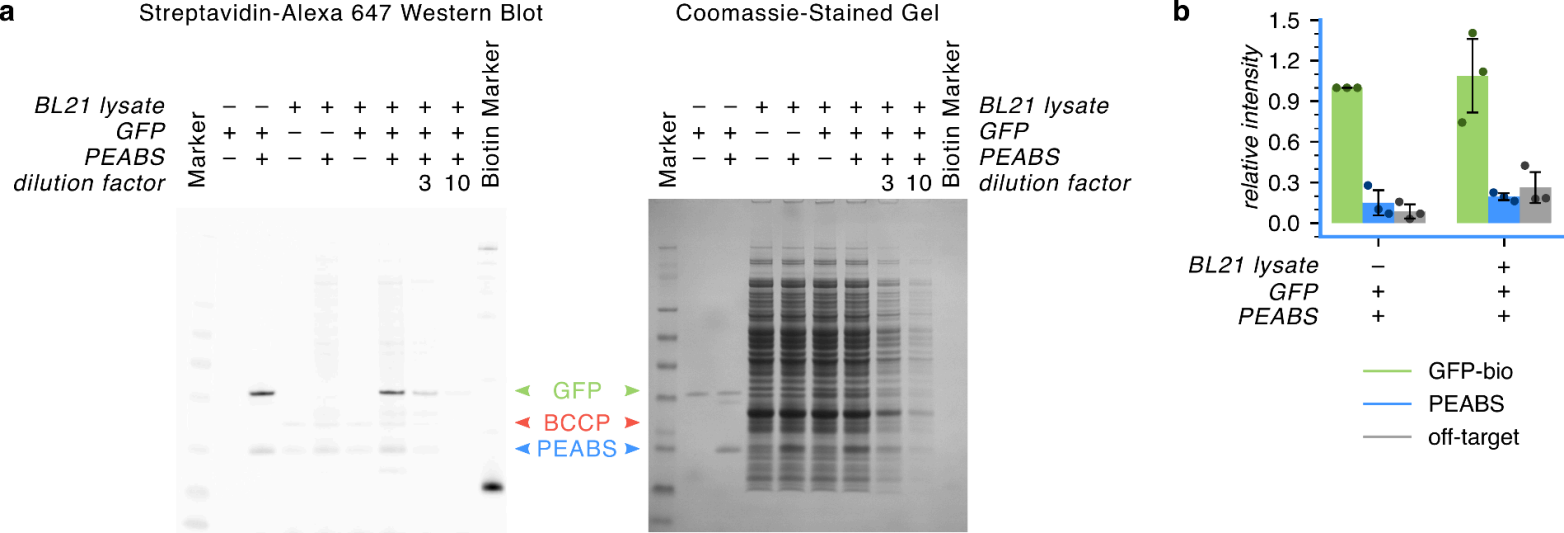
PEABS finds and modifies its target in a complex milieu of cellular components. 1.25 μg (45 pmol) of GFP was incubated with 45 pmol of αGFP-S54-3c-bio (or buffer), with or without 125 μg of soluble protein lysate from *E. coli*. (a) Western blot using biotinylated protein-detecting Streptavidin–Alexa 647. No signal is detected from unmodified GFP (lane 2), but after overnight incubation with PEABS it shows a strong signal, indicating its successful biotinylation (lane 3). Lysate not containing GFP presents two biotinylated bands at ∼14 kDa and ∼20 kDa (likely biotin carboxyl carrier protein;^40^ lane 4), and when incubated overnight with PEABS, only a faint nonspecific signal is added (lane 5). In a lysate containing GFP, only the lysate signals appear (lane 6), but with PEABS, GFP becomes highly biotinylated, whereas other proteins in solution are hardly affected (lanes 7–9). (b) Quantification of the intensities of the bands corresponding to biotinylated GFP and PEABS, and the total signal from all off-target bands in the Western blot (lanes 3 and 7) confirms that GFP is modified to a similar extent with and without off-target proteins in solution and that, despite constituting only 1% of the protein mass in solution, it accounts for 79 ± 11% of the signal. Bars represent *N* = 3 independent experiments and are internally normalized to the intensity of the treated GFP-only band, with error bars representing standard deviations (Figure S5).

With this knowledge, the PEABS design strategy comprising two independent components, an antibody and a synthetic linker, would allow to choose the site of modification first, and only then construct a TPTM agent to modify it by developing antibodies that bind an adjacent epitope. Additionally, the two-component design decouples the choice of binding site from the choice of chemistry; as we have shown, an existing linker can be fitted onto different antibodies, targeting different targets. Similarly, the same antibody can deploy different payloads, and even react with different adjacent residues, by attaching a different linker to its engineered cysteine. We explored the effects that the payload and linker play in specific and nonspecific reactivity of PEABS in Figure 4. We also note that, while we have only explored lysine amidation in this proof-of-concept work, the same principles underlying our linkers could be utilized for other substitution reactions, such as histidine alkylation^48^ or cysteine alkylation.^49^ Potential applications include blocking of undesired natural PTMs^50^ (one example may be blocking proteolysis at lysine contributing the β2m amyloidosis^51^), control of protein–protein interactions^52^ and prevention of disulfide bond formation.^53^

**Figure 4:**
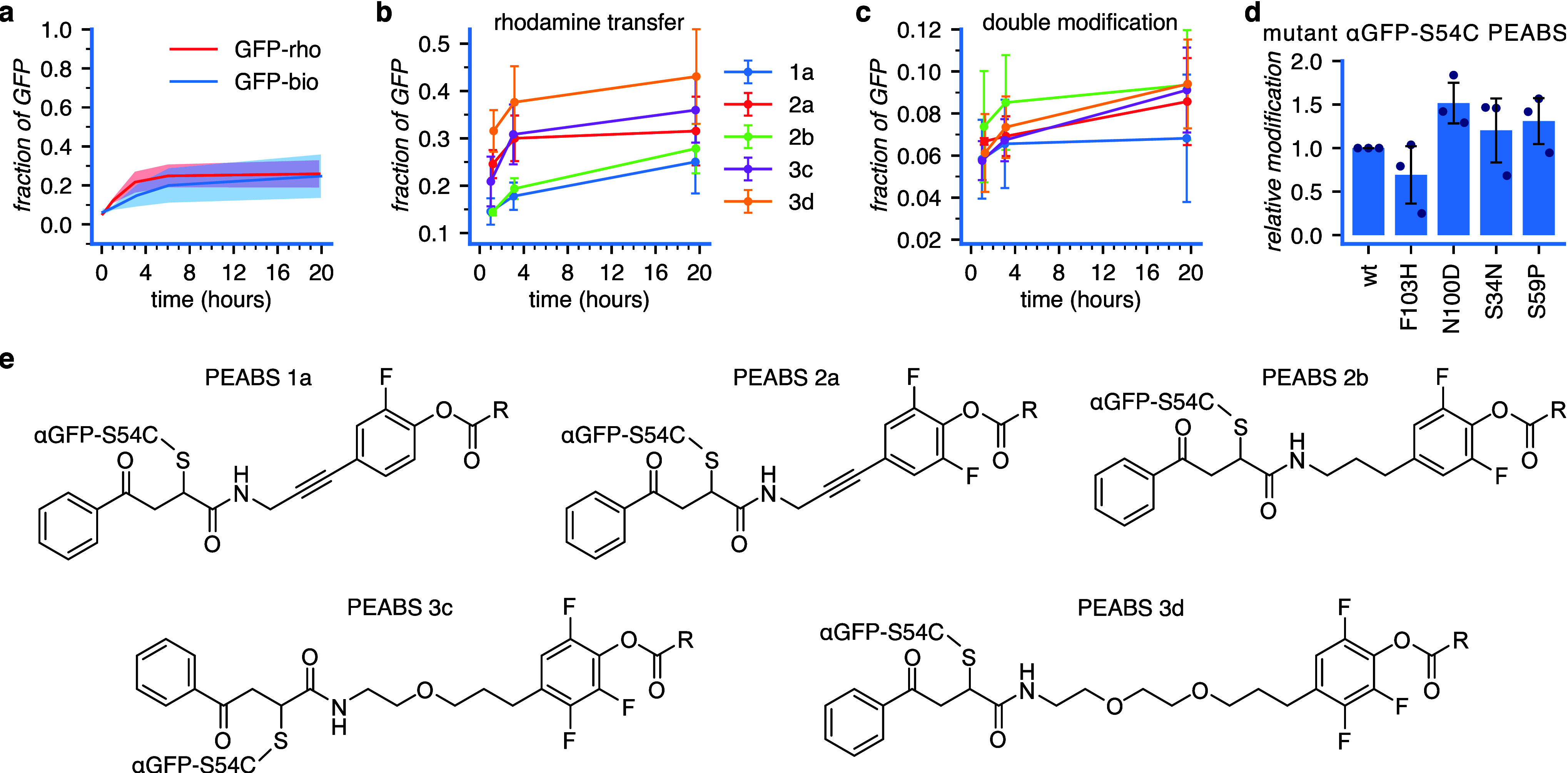
Parameters affecting the specific and nonspecific activities of PEABS. (a) Comparison of payload transfer reaction time courses between αGFP-S54-3c-rho and αGFP-S54-3c-bio, differing only by the payload they carry. While minor differences can be detected, the overall reaction rate is similar. (b) Time-course comparison of αGFP-S54C PEABS based on different linkers, all carrying rhodamine B as their payload. The intrinsic reactivity of the fluorophenol and the hydrophilicity of the linker both contribute to the overall reactivity of the system. (c) Time-course of second rhodamine B transfer. Note the different *y* scale. Here, the intrinsic reactivity of the fluorophenol is the stronger determinant of site nonspecific reaction. (d) Relative modification by mutants of αGFP-S54-3c-rho at the 3 h time point. Mutations were designed to alter the affinity of the nanobody to GFP without negatively affecting its stability. Several mutants show faster modification of GFP and reach overall higher modification levels. Shaded areas represent 95% confidence intervals, error bars represent standard deviations, all errors from *N* = 3 independent experiments (Figures S7, S9b–f). (e) Structures of the PEABS constructs used in panels (b) and (c).

We note that the target selectivity of PEABS can only be as good as that of the antibody it is based on. Of note, higher affinity was also found to not always be better, suggesting that further improvements can be made by minor modifications to the nanobody on which the system is based. Site selectivity was achieved in both GFP and β2m PEABS. However, absolute site specificity is challenging, as high payload transfer activity requires longer, more hydrophilic linkers (Figure 4b), but these also have more nucleophiles of the target within their reach.

While relatively resistant to hydrolysis, PEABS made with fluorophenyl esters do hydrolyze in solution over time, especially at physiological pH or above room temperature (Figure S2b). Improving stability would be a necessary step toward using PEABS systems *in vivo* for therapeutic purposes. At the same time, high (albeit not complete) inverse correlation was observed between the rate of payload transfer by a PEABS system (Figure 4b) and its stability (S2a). The reactions presented here were performed at a 1:1 PEABS:target ratio, leading to no more than 55% modification of the target in most scenarios. Studying the effects of the PEABS–target concentration ratio, the balance between stability and reactivity, the absolute reaction concentration (briefly examined in Figure 5e), and the reaction temperature, would allow for better and safer implementations of PEABS toward therapeutic uses.

**Figure 5:**
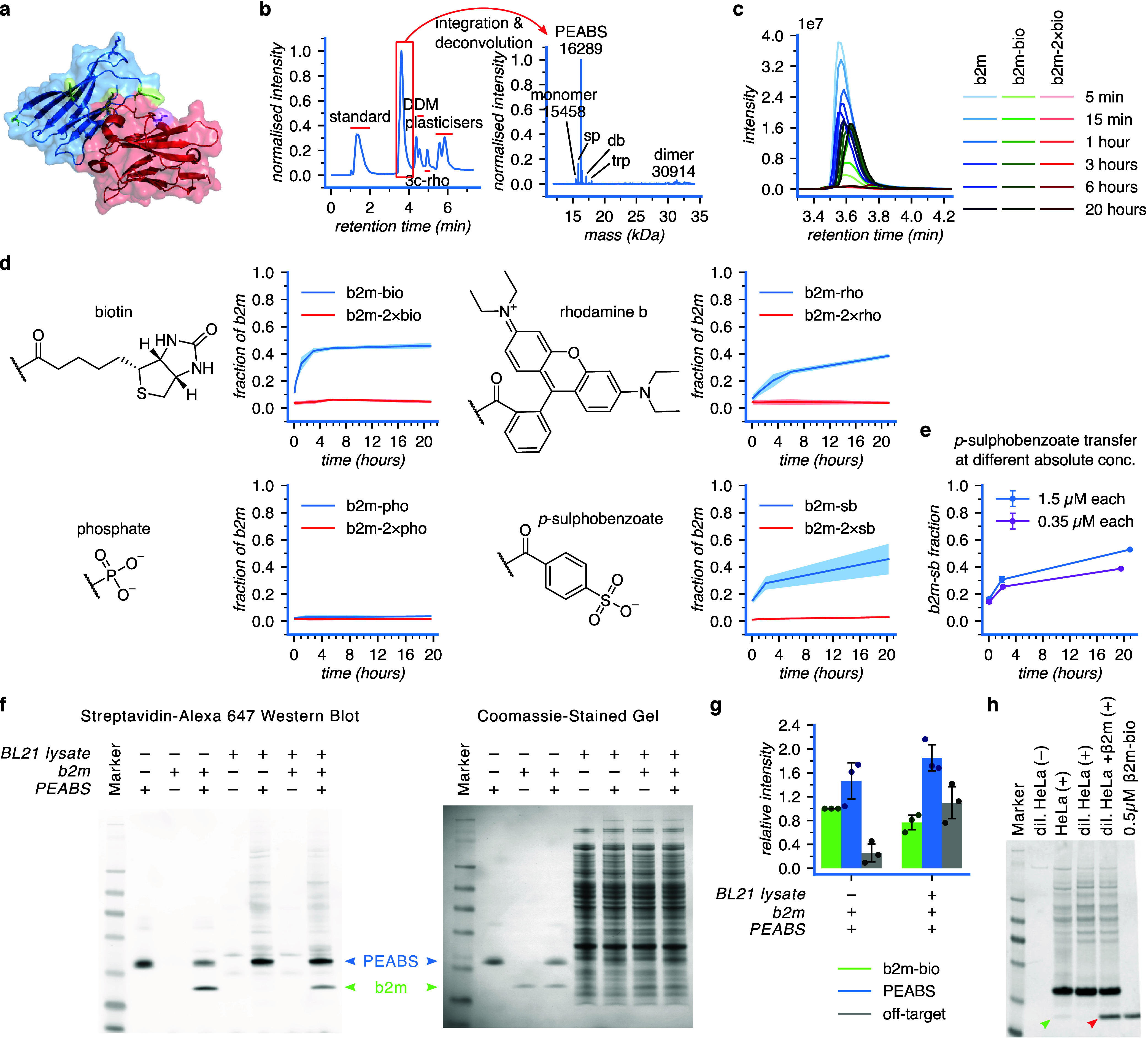
Toward application of PEABS. (a) Structure of the antibody–antigen pair consisting of β2m (blue, with residues K41, K48, K75 (front) and K58 (back) in green) and nanobody nb24 (red with residue 113, chosen for linker conjugation, in purple) [PDB: 4KDT].^44^ (b) Confirmation of bioconjugation of **3c-rho** to nb24-D113C using LC-MS; >80% of the nanobody was converted, with low residual free linker. nb24-D113C *exp.* 14179 *obs.* 14179; PEABS *exp.* 16289 *obs.* 16289; sp (spent PEABS) *exp.* 15865 *obs.* 15865; dbl (PEABS + **3c-rho**) *exp.* 17121 *obs.* 17121; trp (PEABS + 2×**3c-rho**) *exp.* 17953 *obs.* 17953. (c) A representative example of intact-protein LC-MS monitoring of the biotin payload transfer reaction at 25 °C. (d) Kinetics of β2m modification with biotin, rhodamine B, phosphate, or sulfobenzoate. Also see Figure S4 and Tables S4, S5. Here, transfer of biotin occurs more rapidly than transfer of rhodamine B. (e) The nb24-D113-3c-sb reaction with β2m was performed twice at the standard 1.5 μM:1.5 μM PEABS:β2m condition (blue) and twice at a lower 0.35 μM each. The reaction is slightly, but not drastically, slower at the lower absolute concentration. The sb data shown in (d) were derived from a combination of both the low and the standard concentration experiments (*N* = 4). (f) Western blot for detection of off-target modification by nb24-D113-3c-bio using Streptavidin–Alexa 647. (g) Quantification of the intensities of the bands corresponding to biotinylated β2m and PEABS, and the total signal from all off-target bands in the Western blot (lanes 4 and 8) shows lower specificity of the nb24-based PEABS compared to αGFP-based PEABS; however, the on-target signal still accounts for 42 ± 4% of the non-PEABS signal, giving an estimated selectivity ratio of 70:1. Bars represent *N* = 3 independent experiments and are internally normalized to the intensity of the treated β2m-only band (Figure S6). (h) HeLa whole-cell extract diluted to 3 mg/mL was spiked with either 0.5 μM recombinant β2m or the same volume of buffer and then treated with 1 μM PEABS (+) or the same volume of buffer (−). The undiluted extract (∼4 mg/mL) was also treated with the same concentration of PEABS. Samples were incubated at 25 °C for 6.5 h, then analyzed by Western blot using Streptavidin–Alexa 647. Arrowheads indicate signals from PEABS-modified endogenous (green) or recombinant (red) β2m. For more conditions and full analysis see Figure S11. Except where specified otherwise, means represent *N* = 3 independent experiments, shaded areas represent 95% confidence intervals, and error bars represent standard deviations.

One technical limitation of the system presented herein is the incomplete bioconjugation of the linker to the nanobody, leaving some unmodified and overmodified nanobodies and trace amounts of unreacted linkers in solution. The former can compete with PEABS for binding the target, while the latter may contribute to off-target reactivity. We have shown that free linkers are not responsible for significant reactivity (Figure 2b), and that the reaction proceeds well despite the presence of inactive nanobody species (Figure 2d, Figure 3). For medical applications, however, more homogeneous products would still be desired. Utilizing more stable nanobodies was shown to improve the yield of the bioconjugation reaction (compare Figure 1e and Figure 5b). Further improvements may come from exploring cysteine-specific chemistries other than the benzoylacrylic group. Scaling the reaction up would likely also contribute to overcoming some of these challenges (purification of the PEABS construct, removal of surfactants and plasticizers), while it may introduce new challenges, predominantly with storage of the product due to its aforementioned limited stability. Additionally, the pharmacological requirements for TPTM agents, which we have not focused on in this work, would need to be addressed for PEABS to become a viable pharmacological tool. Exploring and improving the pharmacological properties of single-domain antibodies is an active area of research,^28,54^ and we expect that many of the developments in this field would be applicable to PEABS and help bring it closer to the clinic.

In perspective, we expect that this method will be applicable in a wide range of settings in basic research, diagnostics and therapeutics, from labeling specific proteins in the complex biological milieu to post-translationally modifying disease-associated proteins to promote their degradation or protect them from aggregation. Due to the important role played by high-affinity, high-specificity antibodies against selected epitopes, novel approaches to rational antibody design will undoubtedly be crucial for future implementations of PEABS for currently undruggable targets.

## Methods

### Experimental Design

In vitro PEABS experiments were designed to test the stability, efficacy, target-, and site-selectivity and reaction rate of this newly described antibody-based system for targeted post-translational modification of proteins. The target proteins and respective nanobodies were selected for their availability, affinity, being well-characterized with publicly available bound structures, and diagnostic or therapeutic potential. Reaction conditions, transferable payloads, off-target proteins and time-course time points were chosen to balance the requirements of mass spectrometry and other measurement techniques with the aspiration to conduct experiments in near-physiological conditions, while capturing as much information about the characteristics of the PEABS reaction as possible. Specifically, the concentration of PEABS is limited to 7–10 μM by the process of its formation, which includes multiple diluting desalting steps and a precipitation-prone linker. This often determines the upper limit on PEABS concentration in the final reaction. The ratio of PEABS to target protein must be at least 1:1 to theoretically allow full conversion of the target, and the concentration of the target should be at least ∼1 μM for quantitative monitoring by mass spec. This often determines the lower limit on both target and PEABS concentration in the final reaction. In target-specificity experiments, the “background” set of proteins should represent a wide range of sizes, solubilities and nucleophilic reactivities. For small-scale experiments, BSA and α-synuclein were selected for their availability, good resolution on LC-MS, and excellent separation from GFP and from each other on both LC-MS and SDS-PAGE. For large-scale experiments, a bacterial lysate was employed in conjunction with a biotin payload, to simultaneously observe the effect of PEABS on an entire proteome using labeled streptavidin, with minimal background from endogenously biotinylated proteins. The specific parameters of the PEABS formation reaction are discussed in detail in Supplementary Note 2.

### Chemical Linker Synthesis

Linkers **1a**, **2a**, **2b**, **3c** and **3d** were synthesized as detailed in the Supplementary Methods. Biotin (**3c-bio**) or rhodamine B (**1a-rho**, **2a-rho**, **2b-rho**, **3c-rho**, and **3d-rho**) were loaded by esterification and the products purified. A few milligrams of each loaded linker were dissolved in anhydrous dimethyl sulfoxide (DMSO) at 20 mM, divided into 6 μL (120 nmol) aliquots, lyophilized and stored at 4–10 °C in the dark. Just before use, one aliquot was taken out, dissolved back into 6 μL DMSO, and diluted into 6 μL of 4% n-dodecyl-β-d-maltoside (DDM) in water, for a final 10 mM in 50:48:2 DMSO: H_2_O: DDM.

### PEABS Preparation

A 25 nmol (e.g., 400 μL at 63 μM) stock of purified cysteine mutant nanobody (αGFP-S54C or nb24-D113C) in 40 mM 2-ethanesulfonic acid (MES) + 40 mM NaCl, pH 6.1 was thawed from −80 °C to room temperature quickly, incubated on ice for 45 min, centrifuged to remove aggregates (20 min/4 °C/20000 × g) and monomerised by reduction using 8 mol equiv tris­(2-carboxyethyl)­phosphine (TCEP) (2 h at 25 °C, no shaking). A stock of loaded linker (e.g., **3c-rho**) was prepared at 10 mM in 50:48:2 DMSO: H_2_O: DDM. The monomerised antibody was then desalted using PD MiniTrap desalting columns with Sephadex G-25 resin (Cytiva) to remove excess TCEP (which competes with the cysteine for the reaction with the benzoylacrylic moiety) and its concentration was determined using a NanoDrop spectrophotometer. The nanobody was diluted to 20 μM, mixed with a 1:50-diluted linker (final 200 μM, 10 equiv) and supplemented with 60 μM TCEP, then incubated for 50 min at 25 °C without shaking. These conditions were used to achieve maximal formation of PEABS while minimizing overmodified nanobody species (Figure S1, Supplementary Note 2). Finally, the formed PEABS molecule was purified from the unreacted excess linker, surfactant and DMSO by desalting into TPTM reaction buffer (40 mM potassium phosphate + 40 mM NaCl, pH 7.2) and centrifuging to remove suspended material (15 min/4 °C/20000 × g).

### GFP Selectivity Assay

PEABS αGFP-S54-3c-rho was prepared as above. Small-molecule linker controls were prepared by diluting a 10 mM stock of **3c-rho** into the TPTM reaction buffer in the absence (*+* condition) or presence of 2 mol equiv of TCEP (*r* condition). Protein stocks of GFP, α-synuclein and BSA in the TPTM reaction buffer were prepared at 30 μM, and used to prepare 1.8 μM solutions of each or an equimolar mixture of all three (1.8 μM each). 90 pmol (e.g., 9 μL at 10 μM) of either PEABS, free **3c-rho** or TCEP-3c-rho were added to a 50 μL sample of each protein or mixture thereof, representing a 1:1 molar ratio between PEABS and the target. The reaction was allowed to proceed at 25 °C for 5 h (or at 18 °C for 3 h and then at 25 °C for 3 h) and analyzed by LC-MS.

### Time-Course Experiments

PEABS molecules αGFP-S54–3c-rho and αGFP-S54-3c-bio, or nb24-D113-3c-rho and nb24-D113-3c-bio, were prepared as described. A solution of 1.8 μM target (GFP or β2m) in the TPTM reaction buffer was prepared, and to a 100 μL sample of it, 180 pmol (e.g., 18 μL at 10 μM) PEABS was added for a final 1:1 (PEABS: target) molar ratio. The reaction was allowed to proceed at 18 or 25 °C, while samples were analyzed at 5, 15, 60, 180, 360, and 1200 min by LC-MS. Reaction starting times were staggered by 30 min increments to allow parallel analysis. For rhodamine-loaded PEABS comparisons, PEABS molecules were prepared as above, except the final desalting step which was performed using 7K MWCO, 0.7 mL polyacrylamide spin desalting columns (Pierce) in small scale (100 μL + 20 μL stacker). Reactions were started at 10 min intervals to allow parallel analysis, allowed to proceed at 25 °C, and measured by LC-MS at the 1, 3, and 20 h time points.

### Selectivity in a Complex Environment

PEABS αGFP-S54-3c-bio or nb24-D113–3c-bio was prepared as described. For bacterial lysate experiments, BL21­(DE3) clarified lysate was prepared as described in the Supplementary Methods. 50 μg/mL target (1.8 μM GFP or 4.6 μM β2m), lysate (5 mg/mL) or a mixture of both (1:100 target: lysate) were supplemented with an equimolar amount of PEABS, or the same volume of buffer (*untreated controls*). For human cell lysate experiments, 6 μg/mL β2m (0.5 μM), HeLa whole-cell lysate (3–4 mg/mL in RIPA buffer), or a mixture of both, was supplemented with 1 μM PEABS or the same volume of buffer. Reactions were allowed to proceed at 25 °C overnight. Samples of each condition were loaded onto two identical SDS-PAGE gels, which were run in parallel; one was Coomassie-stained and the other analyzed by Western blot to detect biotinylated proteins as described in the Supplementary Methods.

### Other Materials and Methods

For further discussion of the considerations that went into the above procedures, please refer to Supplementary Note 2. Gene and protein sequences and methods for site-directed mutagenesis, protein expression and purification, organic synthesis of compounds, mass spectrometry, Western blot, and protein and small molecule quantification and characterization are available in the Supplementary Methods.

## Supplementary Material



## Data Availability

All substantial data to support the results in this paper are contained within it and its Supporting Information. The mass spectrometry proteomics data supporting Figures 2e and S10c have been deposited to the ProteomeXchange Consortium via the PRIDE partner repository with the data set identifier PXD049693. Raw intact protein mass spectrometry data are available on request from the corresponding authors.

## Notes

Additional data and fully detailed reports, Supplementary Notes about linker design and the PEABS experimental protocol, and Supplementary Methods including protein sequences and properties, detailed experimental and synthetic procedures, and NMR spectra of compounds (PDF)

## Notes

Code for all analyses from raw files is available on GitHub at https://github.com/vendruscolo-lab/PEABS-LC-MS. It is dependent on the MassLynx API, which is freely available from Waters at https://support.waters.com/KB_Inst/Mass_Spectrometry/WKB1775_What_is_the_MassLynx_Raw_Data_Reader_Interface_Library.

## †

The Francis Crick Institute, London, UK

## ‡

St. Anna Children’s Cancer Research Institute (CCRI) and CeMM Research Center for Molecular Medicine of the Austrian Academy of Sciences, Vienna, Austria.
