# Supplementary material for: Targeted Protein Modification with an Antibody-Based System
Source: ACS Cent Sci. 2025 Jul 7;11(8):1364–76. doi: 10.1021/acscentsci.5c00651 (PMC12395305; doi:10.1021/acscentsci.5c00651)
Supplement: Supplementary file 1 [file oc5c00651_si_001.pdf]

Targeted protein modification with an antibody-based system

Oded Rimon[1,†,✉], Juraj Konc[2,‡], Inga Černauskienė[2], Montader Ali[1], Vaidehi Roy Chowdhury[1], Pietro Sormanni[1], Gonçalo J. L. Bernardes[2,3] and Michele Vendruscolo[1,✉]

Supplementary Information

[1]Centre for Misfolding Diseases, Yusuf Hamied Department of Chemistry, University of Cambridge, Cambridge, CB2 1EW, United Kingdom
[2]Yusuf Hamied Department of Chemistry, University of Cambridge, Cambridge, CB2 1EW, United Kingdom
[3]Translational Chemical Biology Group, Spanish National Cancer Research Centre (CNIO), C/ Melchor Fernández Almagro, 3. 28029 Madrid, Spain

† Currently at: The Francis Crick Institute, London, UK
‡ Currently at: St. Anna Children's Cancer Research Institute (CCRI) and CeMM Research Center for Molecular Medicine of the Austrian Academy of Sciences, Vienna, Austria

✉ Corresponding authors:

Prof. Michele Vendruscolo	Dr Oded Rimon
Email mv245@cam.ac.uk	Email oded.rimon@crick.ac.uk
Tel. +44 (0)1223 763873	

Contents

Extended Data **S4**

Supplementary Notes **S20**

Linker design S20

The PEABS experimental protocol S20

In vitro PEABS experimental design S21

Nanobody reduction S22

Preventing aggregation S22

Linker stoichiometry S23

Supplementary Methods **S24**

Sequences S24

Gene: αGFP-S54C as cloned into pET-29b(+) S24

Primers: Increase nanobody pI and extend His-tag S24

Gene: αGFP-S54C, final construct S24

Protein: αGFP-S54C S24

Primers: αGFP-S54C F103H S24

Primers: αGFP-S54C N100D S25

Primers: αGFP-S54C S34N S25

Primers: αGFP-S54C S59P S25

Gene: GFP in pHAT3 (modified) S25

Protein: GFP (uncleaved) S25

Protein: GFP (thrombin-cleaved) S26

Primers: GFP K3S S26

Primers: GFP K41R S26

Primers: GFP K52P S26

Primers: GFP K131S S26

Primers: GFP K140R S26

Primers: GFP K162R S26

Primers: GFP K166R S26

Primers: GFP K209T S26

Primers: GFP K214N S27

Primers: GFP K238R S27

Gene: nb24 . . . . S27

Protein: nb24 . . . . S27

Primers: nb24 D113C S27

Primers: nb24 G114C S27

Protein properties S28

Molecular biology S28

Chemically competent bacterial cells . . . S28
Choice of site mutations . . . S29
Site-directed mutagenesis . . . S29
Protein expression and purification . . . S30
General procedure: expression and affinity purification of soluble proteins . S30
Organic synthesis . . . S32
General methods . . . S33
Linker 1a . . . S34
Linker 2a . . . S40
Linker 2b . . . S45
Group 3 linkers . . . S48
Linker 3c . . . S49
Linker 3d . . . S66
Analytical techniques . . . S72
SDS-PAGE and in-gel fluorescence . . . S72
Western blot for detection of biotinylated proteins . . . S72
Traditional Western blot for beta 2-microglobulin . . . S72
UV/vis spectrophotometry . . . S72
Far-UV CD spectroscopy . . . S73
Intact protein LC-MS . . . S73
Surface plasmon resonance (SPR) . . . S75
Biolayer interferometry (BLI) . . . S75
Small molecule nuclear magnetic resonance (NMR) spectroscopy . . . S76
Small molecule preperative HPLC . . . S76
Small molecule LC-MS . . . S76

Instrument Reports **S77**

References **S172**

Extended Data

Ligand	Analyte	k_a ($M^{-1}s^{-1}$)	k_d (s^{-1})	k_D (M)
αGFP	GFP	1.093×10^{6}	4.756×10^{-5}	4.350×10^{-11}
αGFP-S54C	GFP	1.690×10^{5}	1.883×10^{-4}	1.115×10^{-9}
αGFP-S54-2b	GFP	2.222×10^{5}	1.087×10^{-4}	4.894×10^{-10}
αGFP-S54C-F103H	GFP	7.248×10^{4}	4.868×10^{-4}	6.716×10^{-9}
αGFP-S54C-S34N	GFP	3.827×10^{5}	6.635×10^{-3}	1.733×10^{-8}
αGFP-S54C	*GFP-K41R*	1.503×10^{5}	1.740×10^{-4}	1.158×10^{-9}
αGFP-S54C	*GFP-K131S*	1.445×10^{5}	1.680×10^{-4}	1.162×10^{-9}
αGFP-S54C	*GFP-K140R*	1.619×10^{5}	1.693×10^{-4}	1.046×10^{-9}
αGFP-S54C	*GFP-K162R*	1.354×10^{5}	1.709×10^{-4}	1.263×10^{-9}
αGFP-S54C	*GFP-K166R*	1.579×10^{5}	1.753×10^{-4}	1.110×10^{-9}
β2m	*nb24*	1.708×10^{5}	1.795×10^{-2}	1.051×10^{-7}
β2m	*nb24-D113C*	1.819×10^{5}	1.795×10^{-2}	9.869×10^{-8}

Table S1 | Kinetic parameters of binding of αGFP and its variants to GFP. Surface plasmon resonance (SPR) analysis, fitted by a 1:1 kinetic binding model reveals that the serine to a cysteine mutation at position 54 reduces the affinity by a factor of about 9. Conjugation of a PEABS linker at the cysteine does not have a measurable additional effect on the affinity. Full SPR reports are found in pages S77 (αGFP), S83 (αGFP-S54C), S88 (αGFP-S54-2b), S93 (αGFP-S54C-F103H), S98 (αGFP-S54C-S34N), S103 (GFP-K41R), S108 (GFP-K131S), S113 (GFP-K140R), S118 (GFP-K162R), S123 (GFP-K166R). BLI data appear in Figure S10b.

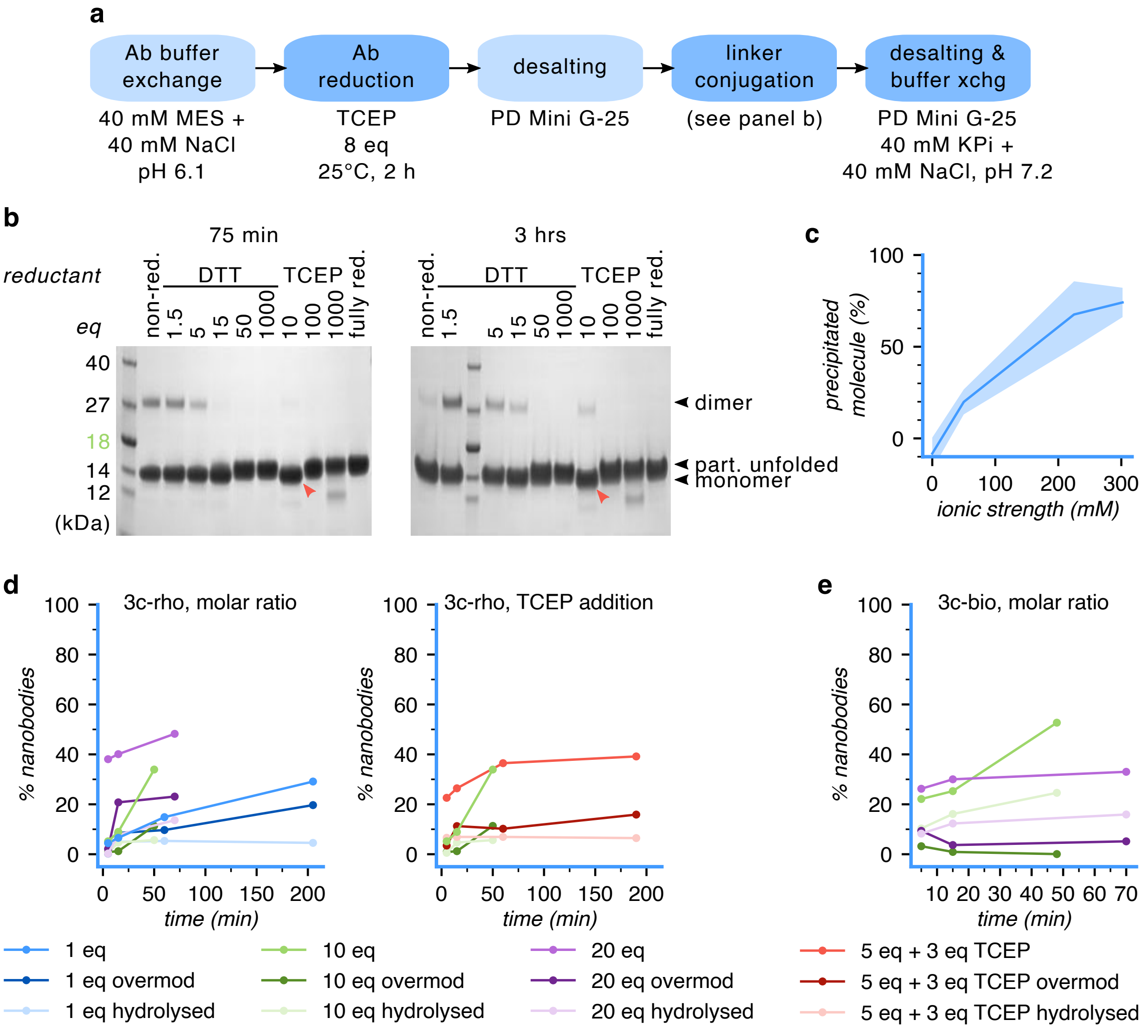

Figure S1 | Parameters in PEABS experimental design. **(a)** General procedure for the bioconjugation of an antibody and a synthetic linker to form PEABS molecules. **(b)** Optimisation of reduction conditions of DesAb-HET-A112C for the elimination of dimers while retaining the intramolecular disulphide bond. Non-reduced control and under-reduced samples show presence of dimers, whereas fully-reduced control and over-reduced samples unfold due to loss of disulphide, evident by the higher apparent protein size. 10 eq TCEP removes most of the dimers while retaining the folded form of the protein (red arrowhead). **(c)** Buffer ionic strength effect on compound 2b-rho precipitation in buffered aqueous solutions. **(d)** Time course measurements of the conjugation of αGFP-S54C to **3c-rho** at 1 : 1, 1 : 10 and 1 : 20 molar ratios, or a 1 : 3 : 5 ratio of nanobody : TCEP : linker. Reintroduction of sub-stoichiometric levels of TCEP may prove useful for suppressing over-modification (right-hand panel). **(e)** Time course measurements of the conjugation of αGFP-S54C to **3c-bio** at 1 : 10 and 1 : 20 molar ratios shows similar trends.

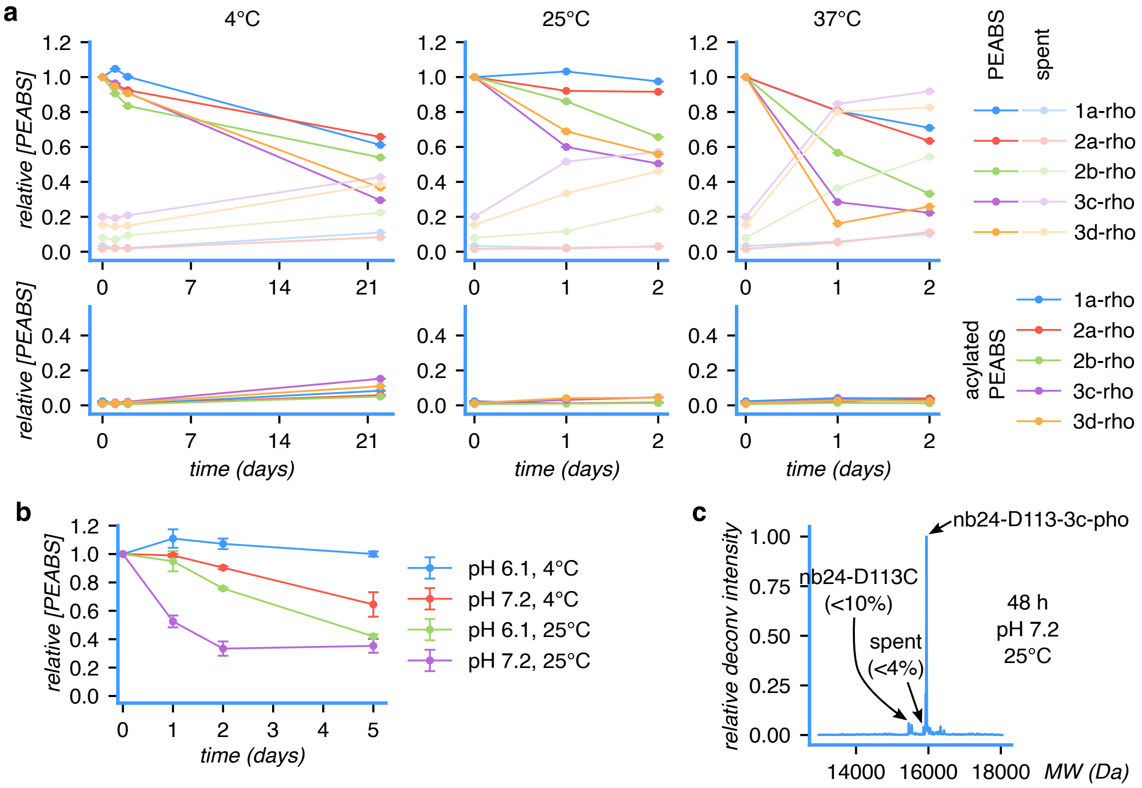

Figure S2 | Stability of PEABS (a) nb24-D113 PEABS constructs were prepared as usual, divided into three portions and incubated at different temperatures. The populations of spent PEABS (PEABS that has lost its payload) increases at a similar rate to the decrease in PEABS population, and continues to rise for weeks at 4 degrees or until the proteins degrade at higher temperatures, suggesting no significant *cis* self-acylation occurs (top panels). Minimal to no appearance over time of PEABS + payload (the result of one PEABS molecule transferring its payload to another, identical, PEABS molecule), perhaps except for the most reactive linkers incubated for over three weeks at 4 °C, suggests minimal to no *trans* self-acylation occurs either (bottom panels). At all temperatures, the rate of loss of payload to solution roughly follows the order expected from the reactivity of the five PEABS constructs. **(b)** αGFP-S54-3c-rho constructs were prepared according to the general procedure and either desalted into the linker-conjugation buffer (pH 6.1) or buffer exchanged into the TPTM reaction buffer (pH 7.2). Then, each was divided into two portions, with one kept at 25 °C and the other in the refrigerator (4–6 °C). At several time points over the course of five days, the fraction of PEABS in solution, relative to total nanobody species, was measured by LC-MS and compared to the starting fraction after desalting. Error bars represent standard deviations over N=2 trials. **(c)** The stability of a PEABS construct is not independent of the payload. nb24-D113-3c-pho, carrying a phosphate payload, does not decompose to an appreciable level even after 2 days at room temperature and physiological pH.

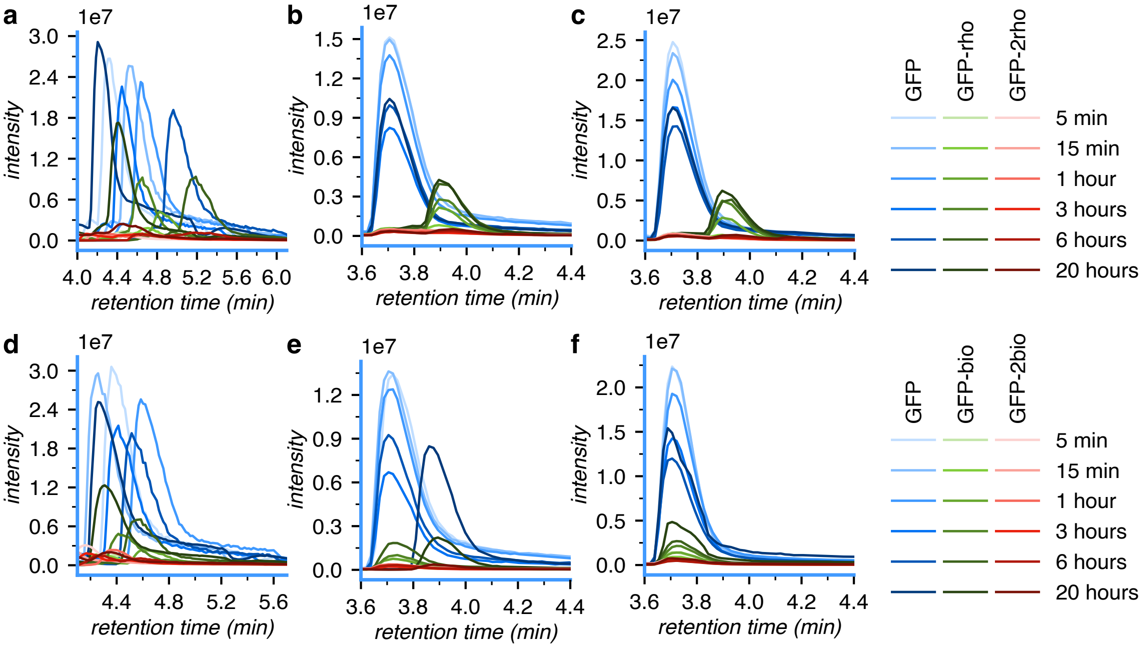

Figure S3 | GFP PEABS 3c-rho and 3c-bio TPTM reaction kinetics. **(a)** Extracted ion chromatograms of GFP species in a time-course experiment of TPTM reaction by αGFP-S54-3c-rho. The area under the curve in the shown region is used to arrive at the numbers in Tables S2, S3. **(b)** As in (a), from second independent experiment. **(c)** As in (a), from third independent experiment. **(d)** Extracted ion chromatograms of GFP species in a time-course experiment of TPTM reaction by αGFP-S54-3c-bio. **(e)** As in (d), from second independent experiment. **(f)** As in (d), from third independent experiment.

Repeat	Time / min	Area under XIC curve			Fraction	
		GFP	GFP-rho	GFP-2×rho	GFP-rho	GFP-2×rho
1	5	7409077	436579	578135	0.051827	0.068631
	15	7777425	571442	581340	0.063990	0.065098
	61	6791886	1086520	572438	0.128569	0.067737
	177	5890933	2132684	673085	0.245229	0.077396
	361	6011579	2720353	1005434	0.279373	0.103255
	1174	7907271	4003605	1547130	0.297489	0.114960
2	5	2804594	163784	239754	0.051053	0.074733
	15	2786424	215579	246050	0.066372	0.075753
	61	2486069	367317	233117	0.119008	0.075528
	188	1406268	417924	148423	0.211863	0.075242
	359	1689374	610572	194301	0.244792	0.077900
	1188	1697486	639569	224706	0.249660	0.087716
3	5	3696606	243289	298701	0.057399	0.070472
	14	3585675	258747	285838	0.062647	0.069206
	60	3087006	444708	257112	0.117374	0.067861
	178	2603006	679532	239255	0.192951	0.067936
	362	2273538	717894	241005	0.222091	0.074558
	1195	2700101	913475	347605	0.230607	0.087753
Mean	5	–	–	–	0.053 ± 0.003	0.071 ± 0.003
	15	–	–	–	0.064 ± 0.002	0.070 ± 0.004
	60	–	–	–	0.122 ± 0.005	0.070 ± 0.004
	181	–	–	–	0.217 ± 0.022	0.074 ± 0.004
	360	–	–	–	0.249 ± 0.024	0.085 ± 0.013
	1186	–	–	–	0.259 ± 0.028	0.097 ± 0.013

Table S2 | GFP PEABS 3c-rho TPTM kinetics data

Repeat	Time / min	Area under XIC curve			Fraction	
		GFP	GFP-bio	GFP-2×bio	GFP-bio	GFP-2×bio
1	5	8193952	492116	276705	0.054907	0.030873
	14	8084148	545100	278458	0.061194	0.031260
	59	7525904	761278	285724	0.088800	0.033329
	189	5911625	1358086	306361	0.179260	0.040438
	372	5800194	2061179	397301	0.249577	0.048107
	1194	7342661	3626413	774775	0.308793	0.065973
2	5	2612749	174809	109369	0.060343	0.037754
	15	2626029	190911	112801	0.065163	0.038502
	60	2377203	212471	103902	0.078881	0.038574
	200	1230673	192526	59693	0.129832	0.040255
	363	1653664	351145	85875	0.167957	0.041075
	1193	1425613	386208	86338	0.203465	0.045485
3	5	3182689	262106	153983	0.072832	0.042788
	14	3146701	263822	149630	0.074104	0.042029
	59	2900510	289386	135704	0.087018	0.040806
	181	2164031	351398	106481	0.134024	0.040612
	361	1879393	443739	105922	0.182680	0.043606
	1200	2641549	844339	185604	0.229972	0.050553
Mean	5	–	–	–	0.063 ± 0.008	0.037 ± 0.005
	15	–	–	–	0.067 ± 0.005	0.037 ± 0.004
	59	–	–	–	0.085 ± 0.004	0.038 ± 0.003
	190	–	–	–	0.148 ± 0.022	0.040 ± 0.000
	365	–	–	–	0.200 ± 0.036	0.044 ± 0.003
	1195	–	–	–	0.247 ± 0.045	0.054 ± 0.009

Table S3 | GFP PEABS 3c-bio TPTM kinetics data

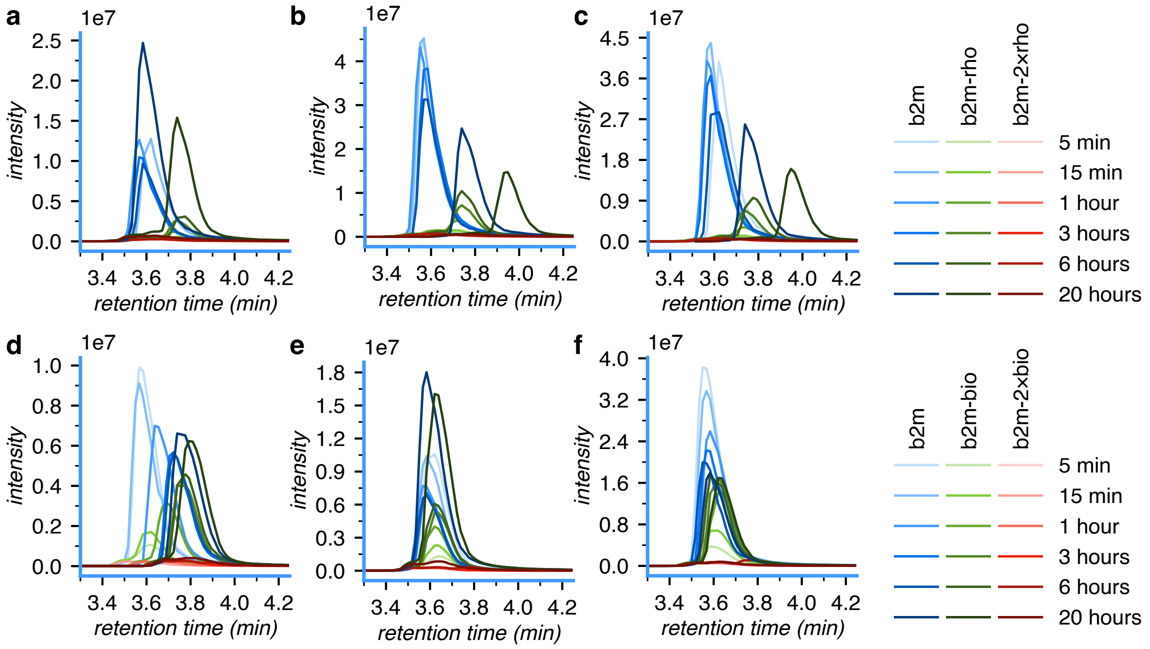

Figure S4 | β2m PEABS 3c-rho and 3c-bio TPTM reaction kinetics. (a) Extracted ion chromatograms of β2m species in a time-course experiment of TPTM reaction by nb24-D113-3c-rho. The area under the curve in the shown region is used to arrive at the numbers in Tables S4, S5. **(b)** As in (a), from second independent experiment. **(c)** As in (a), from third independent experiment. **(d)** Extracted ion chromatograms of GFP species in a time-course experiment of TPTM reaction by nb24-D113-3c-bio. The area under the curve in the shown region is used to arrive at the numbers in Table S5. **(e)** As in (d), from second independent experiment. **(f)** As in (d), from third independent experiment.

Repeat	Time / min	Area under XIC curve			Fraction	
		β2m	β2m-rho	β2m-2×rho	β2m-rho	β2m-2×rho
1	5	1445045	150804	91827	0.089356	0.054410
	15	1618163	175134	96002	0.092698	0.050814
	65	1494737	241245	89441	0.132159	0.048998
	223	1286270	410735	97070	0.228940	0.054106
	364	1159384	477503	95804	0.275585	0.055292
	1396	3041238	2071383	235439	0.387315	0.044023
2	5	5071651	391030	230152	0.068688	0.040428
	13	5322618	441631	225708	0.073729	0.037681
	60	5030704	696689	225463	0.117035	0.037875
	177	4547620	1105995	225969	0.188108	0.038433
	356	3810849	1477146	215106	0.268421	0.039088
	1196	3030144	1939555	183582	0.376373	0.035624
3	5	4538128	303686	197653	0.060262	0.039221
	15	5021024	378708	204066	0.067581	0.036416
	63	4810821	592880	198878	0.105823	0.035498
	180	4301518	1049453	210908	0.188687	0.037920
	361	3914993	1436387	206121	0.258459	0.037089
	1202	3175390	2153607	202716	0.389320	0.036646
Mean	5	–	–	–	0.073 ± 0.012	0.045 ± 0.007
	15	–	–	–	0.078 ± 0.011	0.042 ± 0.007
	62	–	–	–	0.118 ± 0.011	0.041 ± 0.006
	193	–	–	–	0.202 ± 0.019	0.043 ± 0.008
	360	–	–	–	0.267 ± 0.007	0.044 ± 0.008
	1264	–	–	–	0.384 ± 0.006	0.039 ± 0.004

Table S4 | β2m PEABS 3c-rho TPTM kinetics data

Repeat	Time / min	Area under XIC curve			Fraction	
		β2m	β2m-bio	β2m-2×bio	β2m-bio	β2m-2×bio
1	5	1269601	189706	62611	0.124650	0.041140
	15	1159051	270585	62237	0.181373	0.041717
	60	953156	439904	62487	0.302226	0.042931
	182	777006	594840	70453	0.412425	0.048848
	356	768573	664823	89302	0.436609	0.058648
	1202	1030000	936602	110816	0.450849	0.053343
2	5	1448105	217787	68154	0.125595	0.039304
	15	1360543	337859	70472	0.191002	0.039840
	60	989107	521824	69226	0.330235	0.043810
	180	872514	691828	78630	0.421083	0.047859
	355	912546	815093	117660	0.441713	0.063762
	1376	2360401	2184922	221366	0.458373	0.046440
3	5	5011896	633873	160465	0.109171	0.027637
	16	4339945	1052935	166461	0.189399	0.029943
	81	3468777	1963449	183289	0.349647	0.032640
	177	2847450	2309091	196406	0.431368	0.036691
	361	2647693	2426693	347660	0.447560	0.064120
	1201	2351248	2261082	197440	0.470102	0.041050
Mean	5	–	–	–	0.120 ± 0.008	0.036 ± 0.006
	15	–	–	–	0.187 ± 0.004	0.037 ± 0.005
	67	–	–	–	0.327 ± 0.019	0.040 ± 0.005
	179	–	–	–	0.422 ± 0.008	0.044 ± 0.006
	357	–	–	–	0.442 ± 0.004	0.062 ± 0.002
	1260	–	–	–	0.460 ± 0.008	0.047 ± 0.005

Table S5 | β2m PEABS 3c-bio TPTM kinetics data

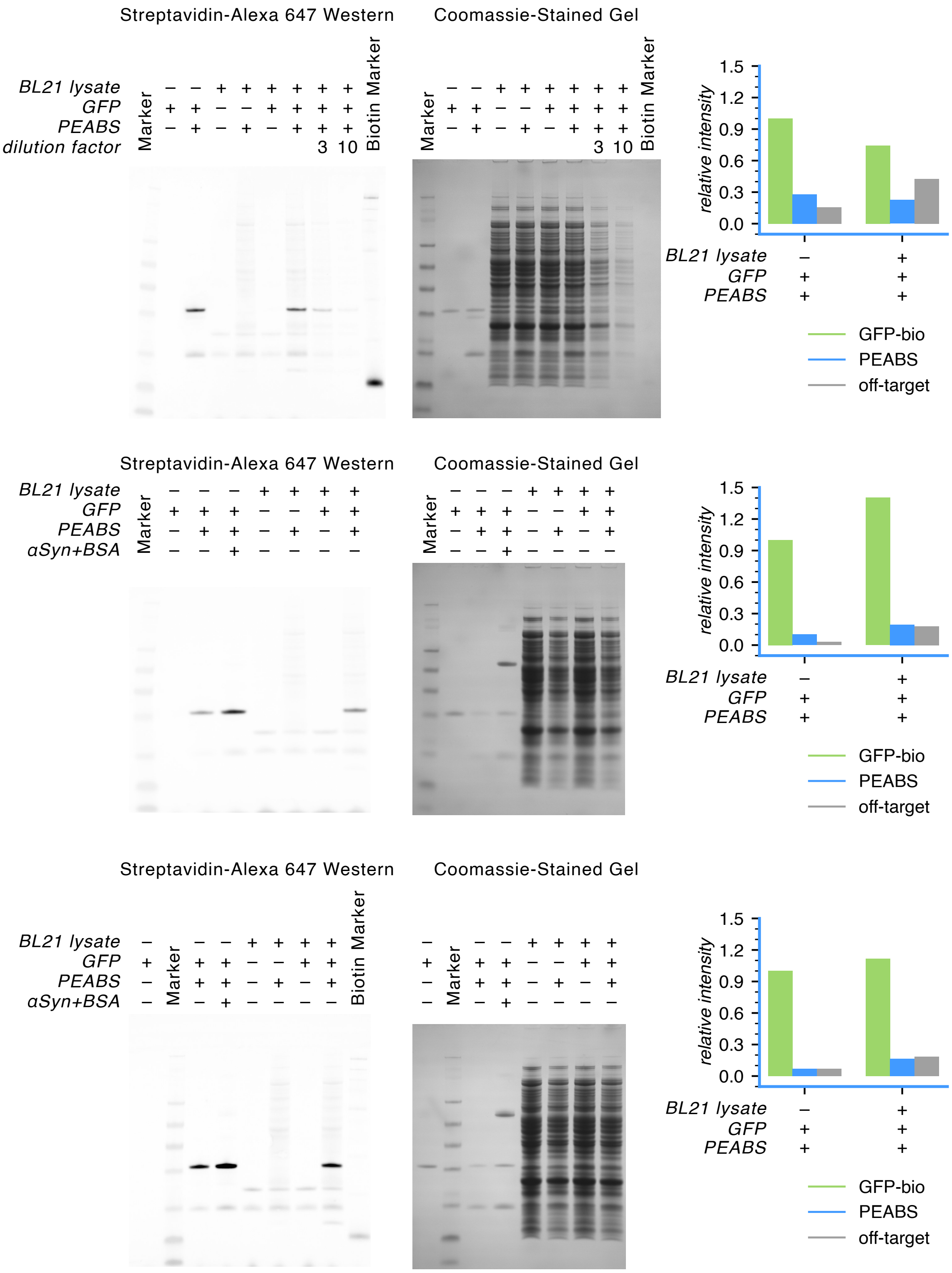

Figure S5 | GFP PEABS modifies its target in a complex milieu of cellular components. Three repeats of the biotin-detecting western blot experiment, with their respective quantification plots. Full report on pages S128, S136, S144.

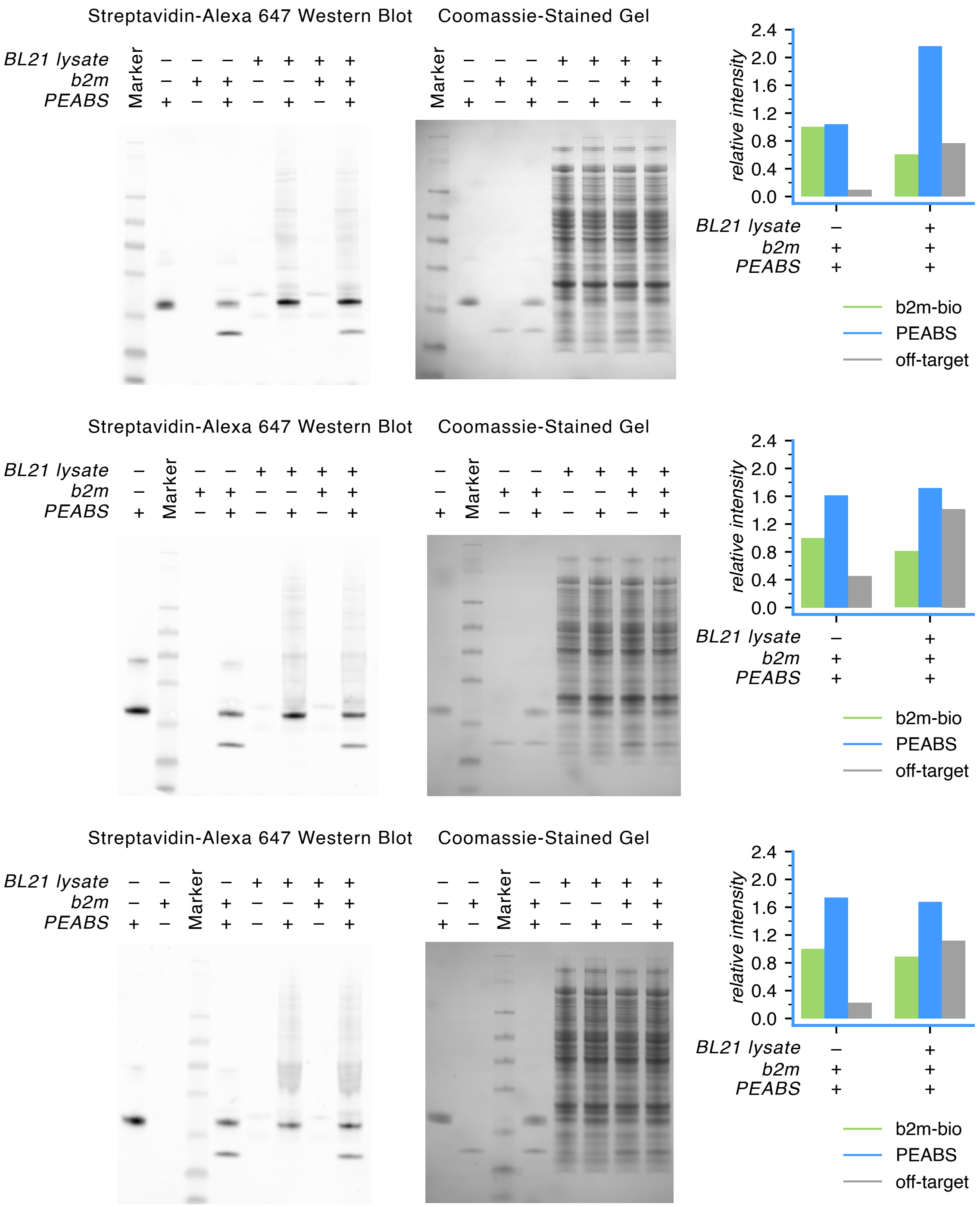

Figure S6 | β2m PEABS modifies its target in a complex milieu of cellular components. Three repeats of the biotin-detecting western blot experiment, with their respective quantification plots. Full report on pages S151, S158, S165.

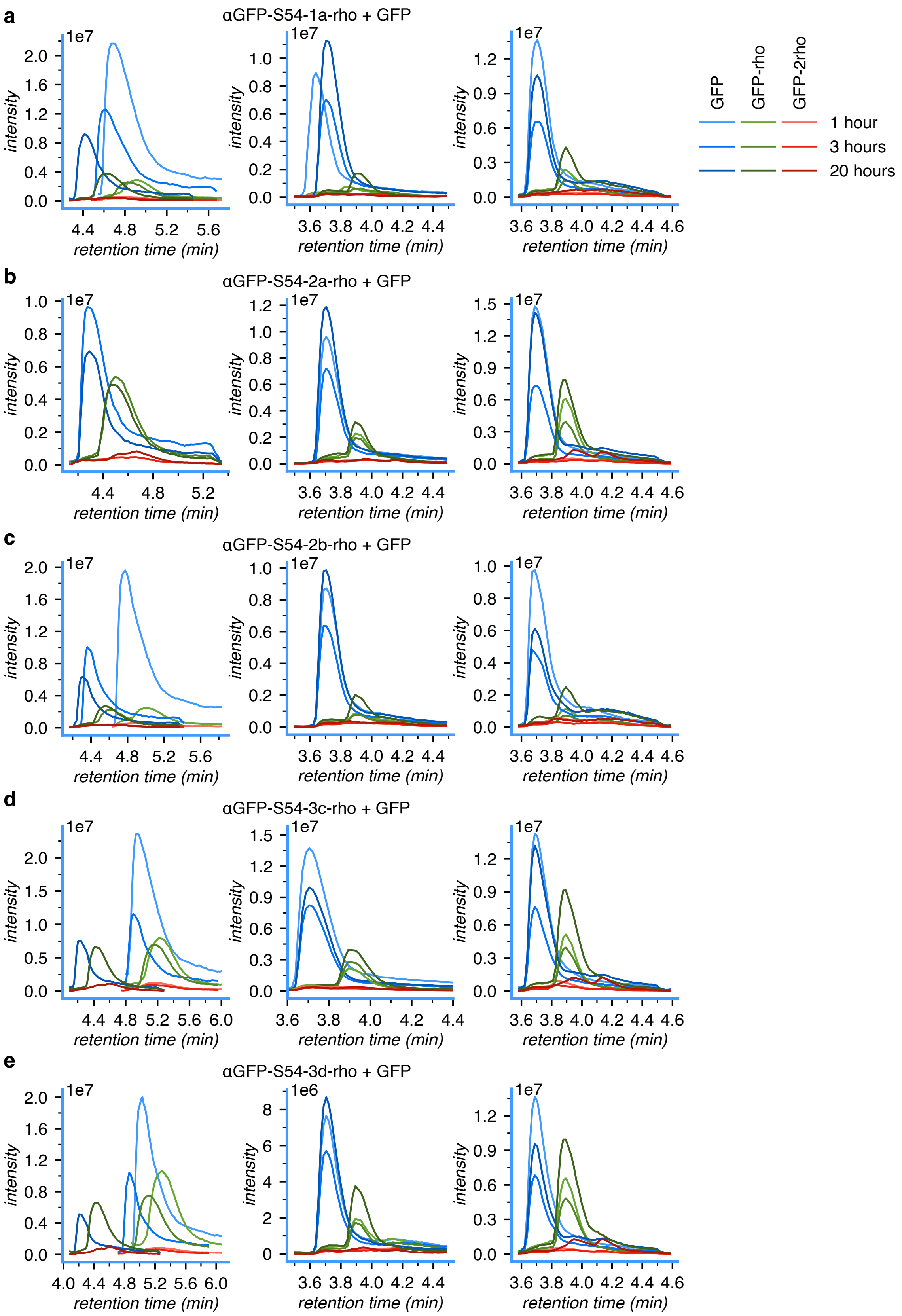

Figure S7 | GFP PEABS 1a-rho, 2a-rho, 2b-rho, 3c-rho and 3d-rho TPTM reaction kinetics. Extracted ion chromatograms of GFP species in three independent time-course experiments of each TPTM reaction by αGFP-S54-1a-rho (a), 2a-rho (b), 2b-rho (c), 3c-rho (d) and 3d-rho (e).

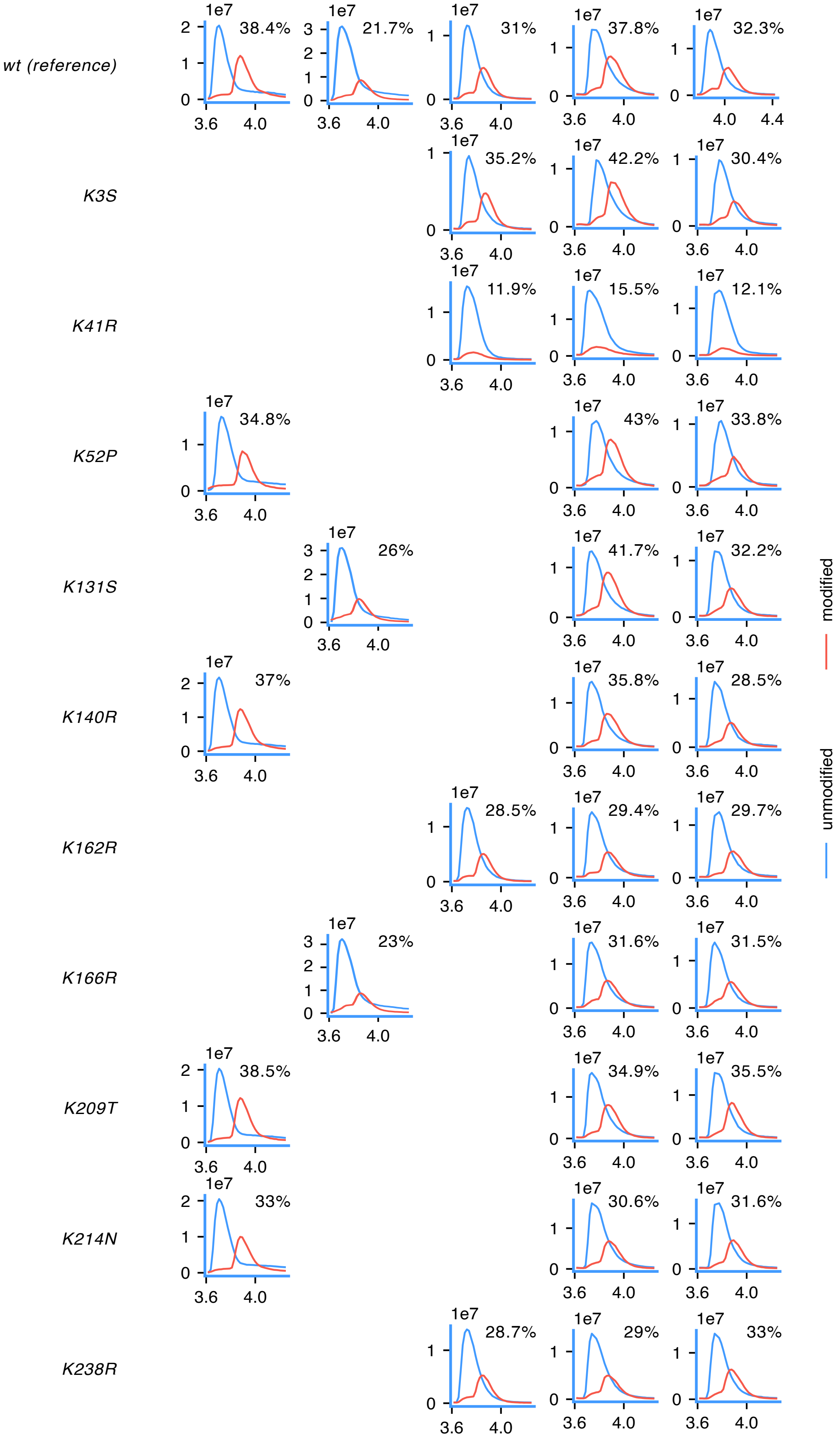

Figure S8 | Systematic removal of lysine residues from GFP. 3 hour data.

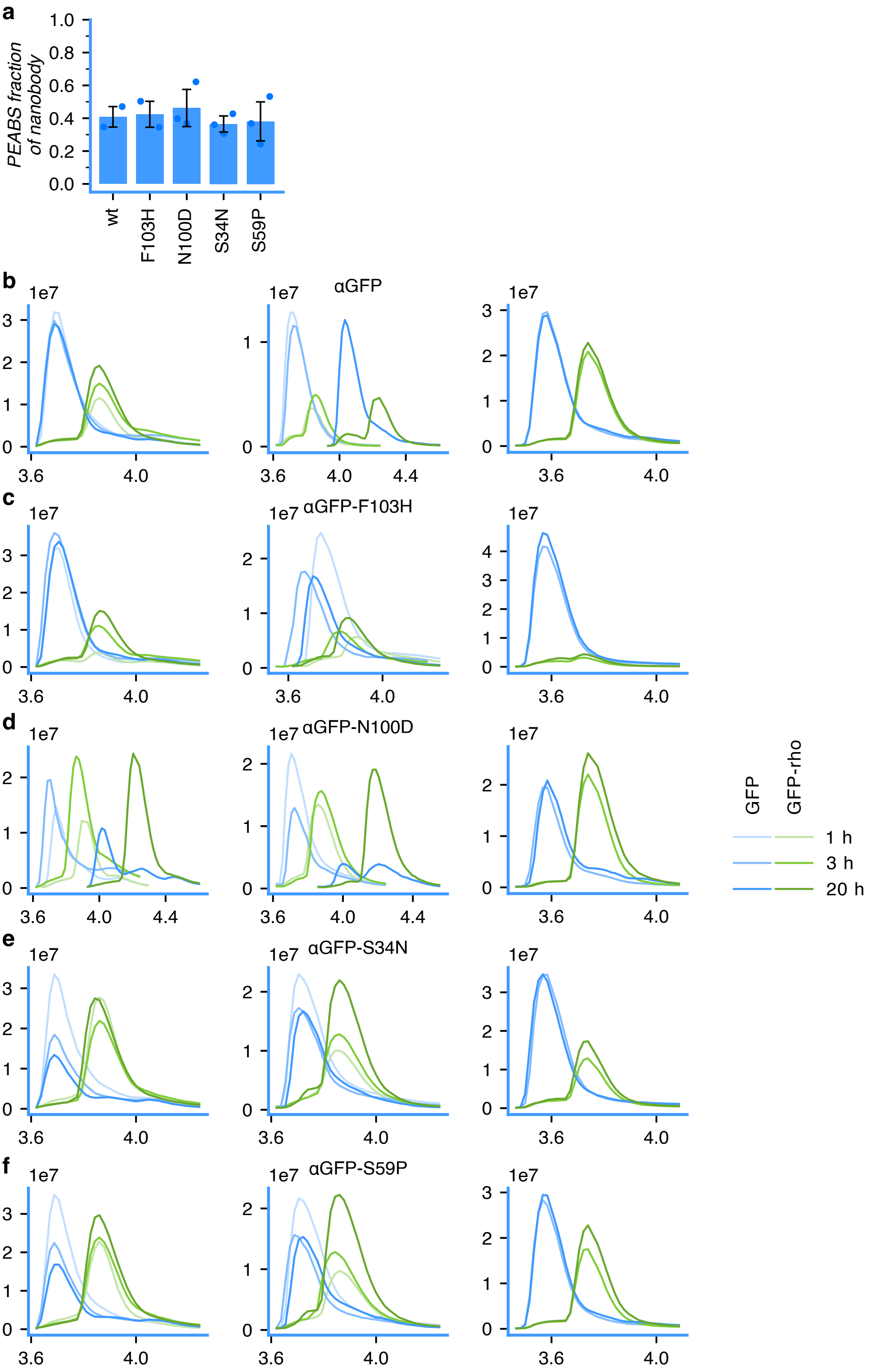

Figure S9 | αGFP nanobodies with altered affinity and their TPTM reaction kinetics. Mutations were designed to affect the affinity of the nanobody to GFP without reducing its stability, and PEABS variants were formed from all at the same time and tested against the same batch of GFP. **(a)** The mutations do not have a significant effect on the efficiency of the bioconjugation reaction for forming PEABS constructs using **3c-rho**. **(b–f)** Modification was measured at 1 hour, 3 hours and 20 hours into incubation at 20 °C.

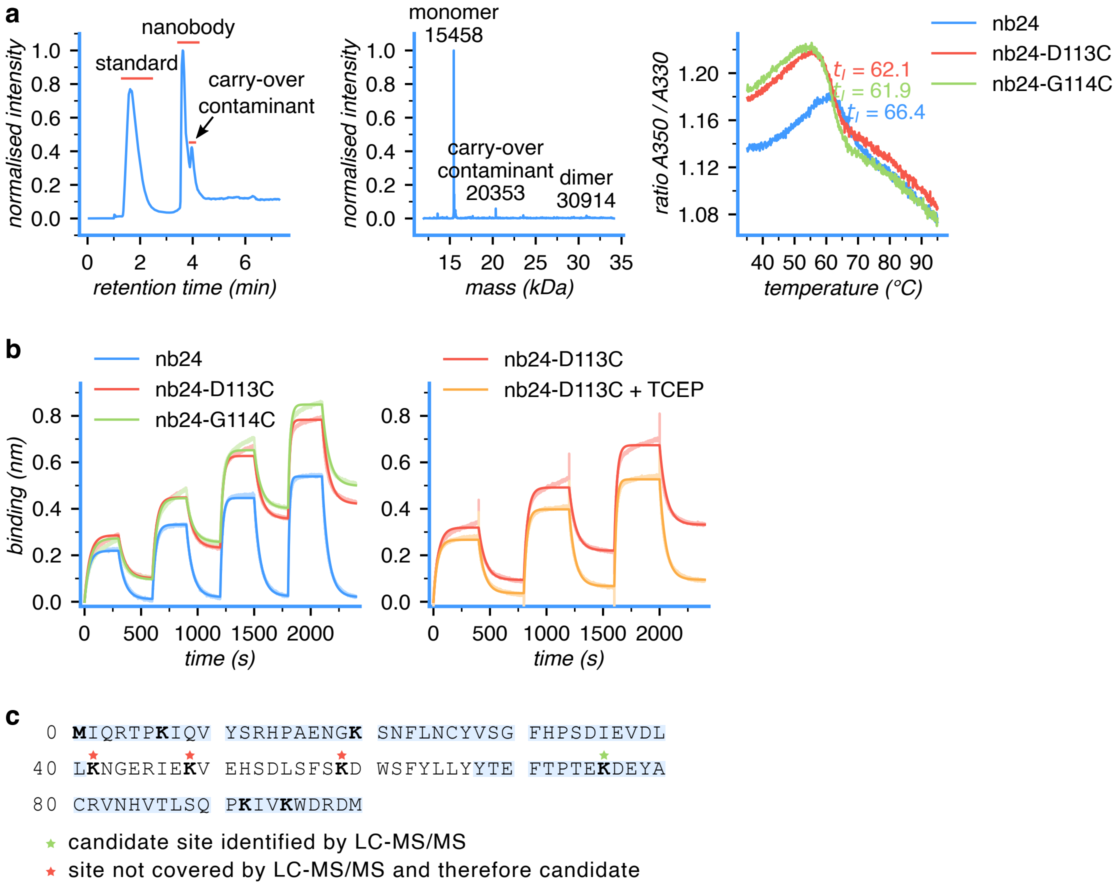

Figure S10 | Characterisation of nb24-based PEABS. (a) Characterisation of nb24-D113C, the cysteine mutant used as basis for PEABS systems, using intact protein LC-MS and nanoDSF melting profiles. **(b)** BLI binding profiles of nb24 and its cysteine mutants to β2m (biotinylated and immobilised to streptavidin biosensors) show comparable dissociation constants among the variants. Adding 8 eq TCEP improves the fit and brings the fitted k_D of nb24-D113C to ~100 nM, essentially identical to that of wild-type nb24 (~105 nM). **(c)** β2m modified by nb24-D113-3c-bio was digested with chymotrypsin and analysed by LC-MS/MS. Regions covered by the analysis are highlighted blue. One site, K75, was identified as a potential candidate for the modification site (green star), and three more sites – K41, K48 and K58, were in regions not covered by the analysis, and are therefore potential sites as well.

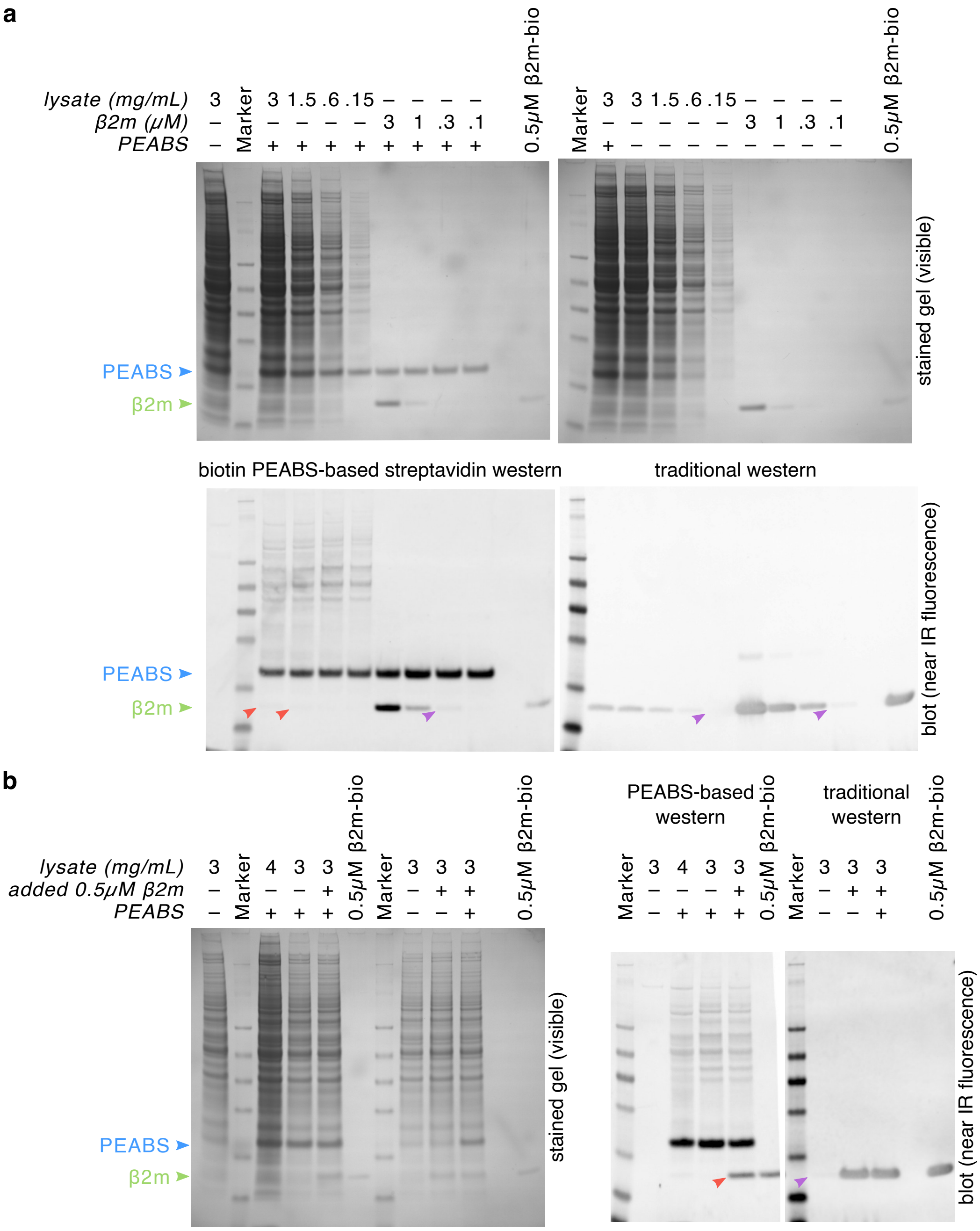

Figure S11 | PEABS can target endogenous β2m in human cell lysate. HeLa whole-cell lysate in RIPA (gift from Dr Alicia González Díaz), measured at ~4 mg/mL (BCA against BSA), was diluted to 3 mg/mL. **(a)** Serial dilutions of lysate and of β2m were incubated with or without 1 µM nb24-D113-3c-bio PEABS at 25 °C for 19 h. Samples were analysed by SDS-PAGE (top), and by either Streptavidin-Western (bottom-left) or traditional Western using rabbit anti-β2m and IRDye-680–bound donkey anti-rabbit antibodies (bottom-right). At high enough concentrations of lysate, endogenous β2m can be detected (red arrows), albeit with low signal-to-noise ratios. The overall detection limit of Streptavidin-Western is seemingly higher than that of traditional Western blot (purple arrows). **(b)** β2m, HeLa whole-cell lysate, or a mixture of both, was incubated with or without 1 µM PEABS for 6.5 h at 25 °C, then all ran together on two SDS-PAGE gels. One gel was stained and imaged (left), while the other transferred to a nitrocellulose membrane, blocked, then cut in half. One half was analysed by Streptavidin-Western (middle) and the other by traditional Western (right). Spiking the sample with β2m allows the signal to cross the non-linear 'toe' portion of the detection curve, revealing that PEABS modifies β2m with some selectivity even under the harsh conditions of a whole-cell lysate (red arrow). Note that in the diluted lysate, even traditional Western blot struggles to detect endogenous β2m (purple arrow).

Supplementary Notes

Linker design

To estimate the lengths of the various PEABS linkers, starting structures were drawn in ChemDraw and imported into GaussView as MOL files. After initial structure clean up and automatic addition of hydrogen atoms, geometry was optimised using density functional theory (DFT) calculations with the B3LYP hybrid functional [1, 2] and the 6-31G basis set [3] as implemented in Gaussian 03 [4]. Converged structures were exported as PDB files and loaded into PyMOL for visualisation.

Small-scale *in vitro* experiments with fluorophenols led to two observations: (1) that these leaving groups would increase the reactivity of their esters towards amines with increasing fluorination; and (2) that the fluorine atoms in the *ortho* position relative to the hydroxyl protect them from hydrolysis. To gain better understanding of this, we performed DFT simulations of the aminolysis of 2,6-difluorophenyl acetate and 2,3,5,6-tetrafluorophenylacetate. First, geometry optimisations of starting materials, intermediates and products in the gas phase were carried out using DFT calculations with the B3LYP hybrid functional (Becke [1], 3 parameter Lee-Yang-Parr [2]) and the 6-31++G basis set [3] as implemented in Gaussian 03 [4]. The tetrahedral intermediate was used as a starting structure for scanning the coordinate of the alcohol hydrogen along the axis of its movement towards the nitrogen (i.e. towards the starting material) or towards the phenol oxygen (i.e. towards the product). These scans were conducted at loose convergence criteria and using the 6-31G basis set without diffuse functions. The transition states were identified as the highest energy coordination along the scan, and optimised as described above with the 6-31++G basis set using the Berny algorithm for transition states [5]. All transition states were verified by intrinsic reaction coordinate (IRC) [6].

Several reaction mechanisms were explored, with the only one that consistently converged and predicted the correct compounds forming being that shown in Figure S12b. The predicted activation energies ($\Delta G^{\ddagger}{}_1$), 31.89 kcal/mol (difluorophenol) and 29.98 kcal/mol (tetrafluorophenol), correspond to a predicted 25-fold higher reaction rate in the latter and mirror our first experimental observation. The high energy tetrahedral transition state TS1 helps capture the potential effect of steric hindrance by the *ortho* position fluorine atoms and explain our second observation. While the conditions of these simulations were not physiological and do not take solvent effects into account, they helped point towards more fluorinated phenols, and particularly 2,3,6-trifluorphenol and 2,3,5,6-tetrafluorophenol, as stable but more reactive groups for the next generation of cleavable linkers.

The PEABS experimental protocol

In this note, we discuss the considerations and observations that have informed the different parameters of the PEABS formation protocol, as well as the experimental protocols used to test it *in vitro*.

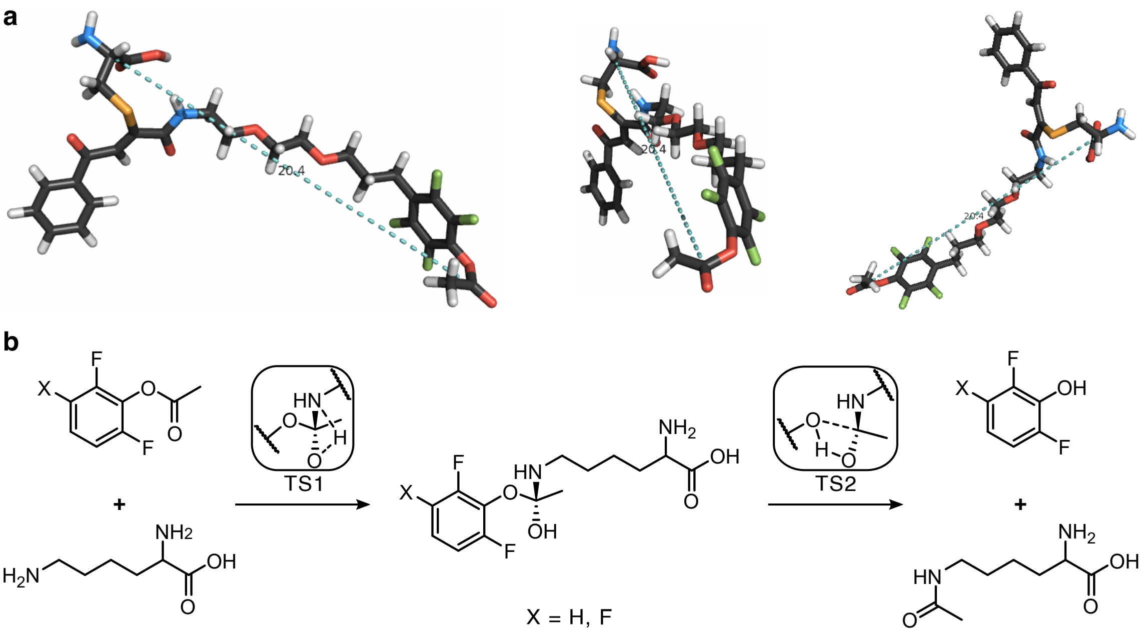

Figure S12 | *In silico* analysis of PEABS linkers. (a) A representative optimised structure of a loaded linker (**3d-acetate**) bound to cysteine at the antibody-binding domain, calculated using density functional theory (DFT). The same structure is viewed from several angles. The distance between the Cα atom of the cysteine and the reactive carbonyl carbon is predicted to be 20.4 Å. **(b)** Mechanism of fluorophenyl ester aminolysis according to simulations. Step 1 is the rate determining step, and is expected to be significantly influenced by steric hindrance from the *ortho* position fluorine atoms. **TS** transition state (only parts differing from the intermediate are shown).

In vitro PEABS experimental design

In vitro PEABS experiments were designed to test the stability, efficacy, target- and site-selectivity and reaction rate of this newly described antibody-based system for targeted post-translational modification of proteins. The target proteins and respective nanobodies were selected for their availability, affinity, being well-characterised with publicly available bound structures, and diagnostic or therapeutic potential. Reaction conditions, transferable payloads, off-target proteins and time-course time points were chosen to balance the requirements of mass spectrometry and other measurement techniques with the aspiration to conduct experiments in near- physiological conditions, while capturing as much information about the characteristics of the PEABS reaction as possible.

The concentration of PEABS is limited to 7–10 µM by the process of its formation, which includes multiple diluting desalting steps and a precipitation-prone linker (see above). This often determines the upper limit on PEABS concentration in the final reaction. The ratio of PEABS to target protein must be at least 1 : 1 to theoretically allow full conversion of the target, and the concentration of the target should be at least ~1 µM for quantitative monitoring by mass spec. This often determines the lower limit on both target and PEABS concentration in the final reaction.

In target-specificity experiments, the "background" set of proteins should represent a

wide range of sizes, solubilities and nucleophilic reactivities. For small-scale experiments, BSA and α-synuclein were selected for their availability, good resolution on LC-MS, and excellent separation from GFP and from each other on both LC-MS and SDS-PAGE. For large-scale experiments, a bacterial lysate was employed in conjunction with a biotin payload, to simultaneously observe the effect of PEABS on an entire proteome using labelled streptavidin, with minimal background from endogenously biotinylated proteins.

Specific parameters of the PEABS formation reaction are discussed below.

Nanobody reduction

Due to the small size and dynamic nature of nanobodies, as well as the strategic placement of engineered cysteine residues for PEABS linker conjugation, cysteine-mutant nanobodies were found to be highly reactive, forming nanobody dimers and oxidised cysteine species *in vitro*. These species cannot be conjugated to the benzoylacrylic functional group, and must therefore be reduced prior to conjugation. However, the structurally necessary internal disulphide bond within these proteins must be retained during reduction.

Using a designed nanobody (DesAb) with an exposed cysteine on its CDR3 loop (A112C), we determined that 5–10 molar equivalents of TCEP successfully diminished the dimer population without significantly affecting the folding state of the monomeric nanobody (Figure S1b). In later experiments, an 8-fold molar ratio of TCEP and quiescent conditions during a 2-hour incubation were found to ideally balance the integrity of the cysteine mutant nanobodies and the reactivity of their cysteine residue towards the PEABS linkers.

Depending on the specific requirements in applications of the PEABS method, the populations of doubly and triply conjugated nanobodies can be lessened by using lower concentrations of TCEP at this step, at the price of a potentially lower yield of PEABS.

Preventing aggregation

In preliminary experiments using the synthesised PEABS linkers, we found that under standard aqueous Michael addition conditions, the linker conjugation reaction was inconsistent in its rate. Often, the linker would precipitate out of solution before binding the nanobody. Systematic examination revealed that parameters contributing to linker precipitation included alkaline pH, high reaction temperature, low DMSO content, salt content (Figure S1c) and most notably shaking of the reaction vessel. On the other hand, higher DMSO concentrations caused misfolding of the nanobody, leading to its aggregation and off-target reactions with the linker.

To solve this, we tested an array of mass spec–compatible detergents, and found that addition of n-dodecyl-β-maltoside (DDM) at a final concentration of 0.04 % allowed the linker to remain soluble for long periods of time under quiescent conditions and react with the nanobody's free cysteine at molar ratios as low as 1 : 1. Reducing the ionic strength of

the solution to 80 mM and lowering the pH to 6.1 (see below) had no observable negative effects on the reaction and contributed further to linker solubility.

We also found that the payload is a significant determiner of loaded linker solubility. Specifically, when attempting PEABS with 4-pentynoic acid and 2-(Fmoc-amino)-4-pentynoic acid (Fmoc-propargylglycine) as payloads on linker **3c**, the solubility of the compounds in the dissolution mixture (50 : 48 : 2 DMSO : H_2O : DDM) was insufficient to proceed with the bioconjugation reaction. This should be considered when choosing payloads for various applications of PEABS.

Once the reaction is completed, desalting allows the removal of the detergent, excess linker and DMSO and readjustment of pH and ionic strength to physiological conditions, without affecting the stability of the PEABS construct. Remaining free linker after desalting precipitates readily and is removed by centrifugation as described in the main text. A small residual quantity of free small molecule may remain in solution in small-volume preparations. For therapeutic purposes, a larger volume reaction would allow this residual amount to be removed completely using size-exclusion chromatography; at the same time, for small-scale experiments, we have found the purity achieved with desalting columns to be more than sufficient.

Linker stoichiometry

Cysteine-reactive electrophiles, and particularly "soft" ones such as the benzoylacrylic functional group, are often used in considerable stoichiometric excess to facilitate full conversion of cysteine residues [7]. However, nanobodies are small and dynamic and feature many sites of potential reactivity, including the two cysteine residues involved in the intramolecular disulphide bond. By varying the stoichiometric ratio of linker to nanobody, as well as reaction temperature, duration, pH and reduction potential, we found the conditions described in the main text – namely, 1 : 10 nanobody : linker ratio, 25 °C without shaking, 50 min at pH 6.1 with 0–3 equivalents of TCEP – led to good conversion of the nanobody to PEABS with minimal double- and triple-modified nanobody (Figure S1d–e).

To maintain the balance between on-target conjugation, off-target reactivity, lab-side practicality and procedure scalability, we would recommend using our experimental conditions as good starting points for case-by-case optimisation, rather than immutable protocols, in applications of the PEABS method to other protein targets.

Supplementary Methods

Sequences

Gene: αGFP-S54C as cloned into pET-29b(+)

The amino acid sequence for anti-GFP nanobody was derived from the crystal structure PDB:3OGO. Reverse translation was performed using a Markov chain-based algorithm developed in-house, generating bacterial-like codon sequences by preferentially selecting codon combinations (pairs, triplets) that co-occur naturally in the *E. coli* genome, then ranking them based on features that are known to inhibit or promote soluble expression in bacteria.

catATGCAAGTCCAGTTAGTTGAATCCGGCGGCGCGTTAGTGCAGCCTGGCGGTTCACTGCGCCTGTCTTGC
GCTGCGTCCGGGTTCCCTGTCAACCGTTATTCCATGCGCTGGTACCGCCAGGCACCAGGTAAAGAACGCGAG
TGGGTAGCGGGTATGAGT*TGC*GCGGGCGATCGCTCCAGCTATGAGGATTCTGTCAAAGGCCGTTTCACCATT
TCACGTGACGACGCGCGCAACACCGTTTATCTGCAAATGAACAGCCTGAAACCTGAAGATACCGCCGTCTAC
TACTGCAACGTGAATGTTGGTTTTGAATACTGGGGCCAGGGCACTCAGGTCACCGTCAGTAGCCTCGAG

Cloning sites: NdeI/XhoI

Primers: Increase nanobody pI and extend His-tag

fwd GTCAGTAGCCGCAGtggcggtcatcaccaccaccaccaccac

rev CTGCGGCTACTGACggtgacctgagtgccctg

Gene: αGFP-S54C, final construct

ATGCAAGTCCAGTTAGTTGAATCCGGCGGCGCGTTAGTGCAGCCTGGCGGTTCACTGCGCCTGTCTTGCGCT
GCGTCCGGGTTCCCTGTCAACCGTTATTCCATGCGCTGGTACCGCCAGGCACCAGGTAAAGAACGCGAGTGG
GTAGCGGGTATGAGT*TGC*GCGGGCGATCGCTCCAGCTATGAGGATTCTGTCAAAGGCCGTTTCACCATTTCA
CGTGACGACGCGCGCAACACCGTTTATCTGCAAATGAACAGCCTGAAACCTGAAGATACCGCCGTCTACTAC
TGCAACGTGAATGTTGGTTTTGAATACTGGGGCCAGGGCACTCAGGTCACCGTCAGTAGCCGCAGTGGCGGT
CATCACCACCACCACCACCACTGA

Protein: αGFP-S54C

MQVQLVESGGALVQPGGSLRLSCAASGFPVNRYSMRWYRQAPGKEREWVAGMS*C*AGDRSSYEDSVKGRFTIS
RDDARNTVYLQMNSLKPEDTAVYYCNVNVGFEYWGQGTQVTVSSRSGGHHHHHHH

Primers: αGFP-S54C F103H

fwd gttggtcatgaatactgGGGCCAGGGCACTC

rev cagtattcatgaccaacATTCACGTTGCAGTAGTAGACG

Primers: αGFP-S54C N100D

fwd `caacgtggatgttgGTTTTGAATACTGGGGCCAG`

rev `caacatccacgttgCAGTAGTAGACGGCGGTATC`

Primers: αGFP-S54C S34N

fwd `ccgttataacatgcgCTGGTACCGCCAGGC`

rev `cgcatgttataacggTTGACAGGGAACCCGG`

Primers: αGFP-S54C S59P

fwd `gatcgcccgagcTATGAGGATTCTGTCAAAGGCCG`

rev `gctcgggcgatcGCCCGCGCAACTCATAC`

Gene: GFP in pHAT3 (modified)

pHAT3 vector carrying the gene for GFP was generously gifted by Dr Marko Hyvönen and Dr Matthew Watson (Department of Biochemistry, University of Cambridge), and minor adjustments were made at the C-terminus.

```
ATGAACACCATTCATCACCATCACCATCACAACACTAGTGGATCTGGTGGTGGTGGCGGCCGCCTGGTTCCG
AGGGGATCCATGGATATCGAATTCATGGTGAGCAAGGGCGAGGAGCTGTTCACCGGGGTGGTGCCCATCCTG
GTCGAGCTGGACGGCGACGTAAACGGCCACAAGTTCAGCGTGTCCGGCGAGGGCGAGGGCGATGCCACCTAC
GGCAAGCTGACCCTGAAGTTCATCTGCACCACCGGCAAGCTGCCCGTGCCCTGGCCCACCCTCGTGACCACC
CTGACCTACGGCGTGCAGTGCTTCAGCCGCTACCCCGACCACATGAAGCAGCACGACTTCTTCAAGTCCGCC
ATGCCCGAAGGCTACGTCCAGGAGCGCACCATCTTCTTCAAGGACGACGGCAACTACAAGACCCGCGCCGAG
GTGAAGTTCGAGGGCGACACCCTGGTGAACCGCATCGAGCTGAAGGGCATCGACTTCAAGGAGGACGGCAAC
ATCCTGGGGCACAAGCTGGAGTACAACTACAACAGCCACAACGTCTATATCATGGCCGACAAGCAGAAGAAC
GGCATCAAGGTGAACTTCAAGATCCGCCACAACATCGAGGACGGCAGCGTGCAGCTCGCCGACCACTACCAG
CAGAACACCCCCATCGGCGACGGCCCCGTGCTGCTGCCCGACAACCACTACCTGAGCACCCAGTCCGCCCTG
AGCAAAGACCCCAACGAGAAGCGCGATCACATGGTCCTGCTGGAGTTCGTGACCGCCGCCGGGATCACTCTC
GGCATGGACGAGCTGTACAAATGA
```

Protein: GFP (uncleaved)

```
MNTIHHHHHHNTSGSGGGGGRLVPRGSMDIEFMVSKGEELFTGVVPILVELDGDVNGHKFSVSGEGEGDATY
GKLTLKFICTTGKLPVPWPTLVTTLTYGVQCFSRYPDHMKQHDFFKSAMPEGYVQERTIFFKDDGNYKTRAE
VKFEGDTLVNRIELKGIDFKEDGNILGHKLEYNYNSHNVYIMADKQKNGIKVNFKIRHNIEDGSVQLADHYQ
QNTPIGDGPVLLPDNHYLSTQSALSKDPNEKRDHMVLLEFVTAAGITLGMDELYK
```

Protein: GFP (thrombin-cleaved)

GSMDIEFMVSKGEELFTGVVPILVELDGDVNGHKFSVSGEGEGDATYGKLTLKFICTTGKLPVPWPTLVTTL
TYGVQCFSRYPDHMKQHDFFKSAMPEGYVQERTIFFKDDGNYKTRAEVKFEGDTLVNRIELKGIDFKEDGNI
LGHKLEYNYNSHNVYIMADKQKNGIKVNFKIRHNIEDGSVQLADHYQQNTPIGDGPVLLPDNHYLSTQSALS
KDPNEKRDHMVLLEFVTAAGITLGMDELYK

Primers: GFP K3S

fwd gagcagcggcgAGGAGCTGTTCACCGGG

rev cgccgctgctcACCATGAATTCGATATCCATGGATCC

Primers: GFP K41R

fwd cggccgcctgACCCTGAAGTTCATCTGCAC

rev caggcggccgTAGGTGGCATCGCCCTC

Primers: GFP K52P

fwd cggcccgctgCCCGTGCCCTGGC

rev cagcgggccgGTGGTGCAGATGAACTTCAGG

Primers: GFP K131S

fwd cttcagcgaggacGGCAACATCCTGGGGC

rev gtcctcgctgaagTCGATGCCCTTCAGCTC

Primers: GFP K140R

fwd gcaccgcctggAGTACAACTACAACAGCCACAAC

rev ccaggcggtgcCCCAGGATGTTGCCGTC

Primers: GFP K162R

fwd catccgcgtgaacTTCAAGATCCGCCACAACATC

rev gttcacgcggatgCCGTTCTTCTGCTTGTCGG

Primers: GFP K166R

fwd cttccgcatccgCCACAACATCGAGGACG

rev cggatgcggaagTTCACCTTGATGCCGTTC

Primers: GFP K209T

fwd gagcaccgaccCCAACGAGAAGCGCG

rev ggtcggtgctcAGGGCGGACTGGG

Primers: GFP K214N

fwd cgagaaccgcgATCACATGGTCCTGCTG

rev cgcggttctcgTTGGGGTCTTTGCTCAG

Primers: GFP K238R

fwd ctgtaccgctgataagGATGCATAAGCTTGAGTATTCTATAGTGTC

rev cttatcagcggtacagCTCGTCCATGCCGAGAG

Gene: nb24

The amino acid sequence for the anti-β2m nanobody nb24 was derived from the crystal structure PDB:4KDT. Reverse translation was performed using the same method described for αGFP above.

ccATGgaaCAAGTGCAGTTACAGGAATCTGGTGGTGGTTCCGTGCAGGCGGGCGGCAGTTTACGCCTGTCGT
GTGCTGCTTCAGGCTATACCGATTCTCGCTACTGCATGGCATGGTTTCGACAAGCGCCAGGCAAGGAACGTG
AATGGGTAGCACGTATTAACAGCGGTCGCGATATTACTTACTATGCGGACAGTGTTAAGGGGCGCTTTACCT
TCAGTCAGGATAACGCAAAGAACACCGTTTATCTGCAGATGGATTCGCTGGAACCAGAAGATACCGCCACTT
ATTACTGCGCGACAGATATTCCTTTACGCTGCCGTGATATCGTGGCAAAAGGCGGTGATGGCTTTCGCTATT
GGGGTCAGGGAACTCAAGTTACTGTCAGTTCACTCGAG

Cloning: using NcoI/XhoI into pET-28b(+)

Protein: nb24

MEQVQLQESGGGSVQAGGSLRLSCAASGYTDSRYCMAWFRQAPGKEREWVARINSGRDITYYADSVKGRFTF
SQDNAKNTVYLQMDSLEPEDTATYYCATDIPLRCRDIVAKGGDGFRYWGQGTQVTVSSLEHHHHHH

Primers: nb24 D113C

fwd cggttgcggctttcGCTATTGGGGTCAGGGAACTCAAG

rev gaaagccgcaaccgCCTTTTGCCACGATATCACGGCAG

Primers: nb24 G114C

fwd ggtgattgctttcgCTATTGGGGTCAGGGAAC

rev cgaaagcaatcaccGCCTTTTGCCACGATATC

Protein properties

protein	amino acids	unprocessed molecular weight (Da)[a]	final molecular weight (Da)[b]	attenuation coefficient ε ($M^{-1} \cdot cm^{-1}$)[a]	isoelectric point[a]
αGFP	127	14164.6	14162.6	27000 ± 60	8.6[c]
αGFP-S54C	127	14180.7	14178.7	27000 ± 60	8.5[c]
αGFP-S54C-F103H	127	14170.7	14168.7	27000 ± 60	8.5
αGFP-S54C-N100D	127	14181.7	14179.7	27000 ± 60	7.8
αGFP-S54C-S34N	127	14207.7	14205.7	27000 ± 60	8.5
αGFP-S54C-S59P	127	14190.8	14188.7	27000 ± 60	8.5
nb24	138	15474.1	15470.0	28550 ± 130	6.3
nb24-D113C	138	15462.1	15458.1	28550 ± 130	6.5
nb24-G114C	138	15520.1	15516.1	28550 ± 130	6.3
His-GFP	271	30357.2	30337.2	21950 ± 60	6.0
GFP	246	27721.3	27701.3	21950 ± 60	5.4
GFP-K>S	246	27680.2	27660.2	21950 ± 60	5.3
GFP-K>R	246	27749.4	27729.3	21950 ± 60	5.4
GFP-K52P	246	27690.3	27670.2	21950 ± 60	5.3
GFP-K209T	246	27694.3	27674.2	21950 ± 60	5.3
GFP-K214N	246	27707.3	27687.2	21950 ± 60	5.3
β2m	100	11862.4	10860.3	20000 ± 60	6.0

[a] Values predicted by Expasy ProtParam. [b] Predicted mass following disulphide bond or active site formation. [c] We found it is beneficial to keep the pI of the nanobody outside the range experienced in PEABS experiments (6.1–7.4), and therefore introduced the modified C-terminus shown above.

Molecular biology

Chemically competent bacterial cells

This is a modified version of Inoue's method [8]. XL10 gold (Agilent) bacteria were plated on LB+Agar plates with selective antibiotics, and single colonies were used to inoculate 10 mL LB cultures and incubated at 37 °C overnight. 240 mL LB media containing 10 mM $MgSO_4$ and 10 mM $MgCl_2$ were inoculated with a 1:100 diluted O/N culture and

incubated at 37 °C until $0.3 < OD_{600} < 0.5$. The culture was incubated on ice for 30 min, then cells were pelleted (15 min / 4 °C / 4000 × g), re-suspended in 240 mL ice-cold 100 mM $MgCl_2$, incubated on ice 10 min and pelleted again under same conditions. Pellet was resuspended in 120 mL ice-cold "TB Buffer" (250 mM KCl + 10 mM HEPES stock + 15 mM $CaCl_2$ + 55 mM $MnCl_2$), incubated at 4 °C for 10 min and pelleted. Pellet was then resuspended again in 5 mL ice-cold "TB Buffer", incubated at 4 °C for 10 min and pelleted one last time. Finally, pellet was re-suspended in 5 mL ice-cold "TB Buffer" + 7 % DMSO (v/v) and incubated on ice. The cells were aliquoted into 100 µL portions, shock-frozen in liquid N_2 and stored at -80 °C.

Choice of site mutations

Site mutations for affinity reduction were chosen using a two-step process. First, the unbound structure of the αGFP nanobody was uploaded to the CamSol Combination tool [9], and sites on the GFP-binding surface where mutations were found to stabilise – or at least not dramatically destabilise – the free nanobody, were noted. Then, the GFP-bound structure was uploaded to the same platform, and the sites identified in the previous scan were explicitly listed as custom mutation sites. Mutations that had a predicted destabilising effect on the complex, but not on the nanobody itself, were selected as candidates for affinity reduction.

Substitute residues for GFP lysines were selected similarly. The structure of GFP was uploaded to CamSol Combination [9] and the lysine residues to be removed were listed as custom mutation sites. For each site, the substitute that was predicted to cause the least destabilisation to the structure was selected. Where no suitable substitutes were identified using this tool, arginine (K → R) was used by default.

Site-directed mutagenesis

Template plasmids were mixed with forward and reverse primers, dNTP mix, HF buffer, DMSO and Phusion polymerase enzyme (NEB) according to the manufacturer's protocol. PCR reactions were performed on a Bio-Rad C1000 Touch Thermal Cycler using the sequence: 98 °C for 2 minutes; 20 × (98 °C for 10 sec; annealing temperature (varies) for 30 sec; 72 °C for 165 sec); then another 5 × (98 °C for 10 sec; annealing temperature - 10 °C for 30 sec; 72 °C for 165 sec); and finally 72 °C for 10 min. The annealing temperature for primers designed using the Naismith method [10] was the primer–primer overlap's melting temperature plus 3 to 4 °C. Products were digested using DpnI (NEB), purified using a GeneJET PCR Purification kit (Thermo Scientific), quantified on a NanoDrop and transformed at 1 µL (typically, 100–150 ng) into chemically competent XL10 bacteria (made as described). Bacteria were selected on antibiotics-containing LB+agar plates. Several colonies were picked up, grown overnight, and used to harvest plasmids using a GeneJET Plasmid Miniprep kit (Thermo Scientific).

Protein expression and purification

Gene integrity and correctness was verified by sequencing at the DNA Sequencing Facility, Department of Biochemistry, University of Cambridge. Plasmids were then transformed into chemically competent *E. coli* BL21-gold(DE3) (Agilent) or SHuffle T7 (NEB) by adding 10 ng of plasmid DNA into a vial of competent bacteria and applying heat shock of 42 °C for 45 seconds. Transformed cells were diluted 10-fold into pre-warmed SOC medium, recovered for 45 minutes at 37 °C under 300 rpm shaking, then selected by resistance to antibiotics (ampicillin or kanamycin) on LB-agar plates. Colonies from overnight growth were picked into liquid LB, grown overnight, supplemented with glycerol (final 10 %), flash frozen in liquid N_2 and kept at -80 °C. For expression tests, solubility tests and protein purifications, fresh antibiotics selection plates were streaked with bacteria from the -80 °C stocks, and colonies from overnight-grown plates were picked and used to inoculate starter cultures, which in turn – upon overnight growth – were used to inoculate expression cultures by 100-fold dilution.

General procedure: expression and affinity purification of soluble proteins

1 L of LB media was inoculated with an O/N starter culture of cells. Cells were grown to OD_{600} of 0.7–0.9 at 37 °C; then expression was induced by adding IPTG to a final concentration of 0.5 mM. Cells were cultured overnight at 18 °C, then harvested from the media by centrifugation (25 min / 4 °C / 6200 × g) and resuspended in 50 mL buffer. The resuspended bacteria were then pelleted again under similar conditions, resuspended in 50 mL buffer + 10 mM imidazole and supplemented with a tablet of cOmplete EDTA-free protease inhibitor cocktail (Roche). After 30 min incubation on ice, the cells were sonicated using a Sonics Vibra-Cell for 5 minutes of active time at 40 % amplitude, in 15 sec on 45 sec off pulses. The clarified lysate was centrifuged (15 min / 4 °C / 39000 × g) to remove cell debris and insoluble or aggregated proteins. Proteins were purified from the 0.45 µm–filtered supernatant of the lysate using a gravitational column loaded with 4 mL Ni-NTA Agarose resin (Thermo) and equilibrated against buffer + 10 mM imidazole. The flow through was re-introduced into the column for maximal binding; this was repeated twice more. Non-specifically bound proteins were then washed with 50 mL buffer + 55 mM imidazole, and the tagged protein was eluted with 6 × 8 mL fractions of buffer + 300 mM imidazole and immediately supplemented with 0.2 mM ethylenediaminetetraacetic acid (EDTA) to prevent aggregation on nickel. Fractions were assessed by SDS-PAGE, and the more pure product-containing fractions were pooled.

αGFP nanobody and its variants pET-29b(+) vector carrying the gene for αGFP or one of its variants was transformed into SHuffle T7 bacteria as described, and selected for kanamycin resistance. Expression and affinity purification followed the general procedure, with 50 mM potassium phosphate + 500 mM NaCl, pH 7.0 as the buffer. Unwanted proteins were precipitated using ammonium sulphate at 30 % saturation (170 mg/mL)

on a tube roller at 4 °C for at least 1 hour, and pelleted by centrifugation (15 min / 4 °C / 10000 × g). The supernatant was decanted into a fresh tube, supplemented with an additional 300 mg/mL ammonium sulphate (final saturation 75 %), incubated on a tube roller at 4 °C for at least 1 hour, and pelleted by centrifugation. The supernatant was discarded, and the pellet resuspended in 3 mL buffer, mixed thoroughly at 4 °C, and centrifuged to remove suspended material (5 min / 4 °C / 5000–10000 × g). The clarified supernatant was reduced using 200 µM TCEP for 2 h at rt, then loaded onto a HiLoad 16/600 Superdex 75 prep-grade size exclusion chromatography column (GE Healthcare) on an Äkta Prime system (GE Healthcare). The buffer used for resuspension, equilibration and elution was 40 mM MES + 40 mM NaCl, pH 6.1. Peak fractions were collected, pooled, flash frozen in liquid N_2 and stored at -80 °C.

GFP GFP in pHAT3 was transformed into BL21(DE3) cells (New England Biolabs) as described, and selected for ampicillin resistance. Expression and affinity purification followed the general procedure, with PBS as the buffer and 40 mM imidazole in the washing step. Imidazole was removed by dialysis to PBS at 4 °C overnight. The His-tag was cleaved using thrombin protease (GE Healthcare) according to manufacturer instructions. The cleaved protein was purified by reverse affinity chromatography followed by size-exclusion chromatography, eluting with PBS. Peak fractions were collected, pooled, flash frozen in liquid N_2 and stored at -80 °C.

GFP variants Mutated GFP in pHAT3 was transformed into BL21(DE3) and selected for ampicillin resistance. Expression and affinity purification followed the general procedure, with PBS as the buffer and 40 mM imidazole in the washing step. Unwanted proteins were precipitated using ammonium sulphate at 30 % saturation (170 mg/mL) on a tube roller at 4 °C for at least 1 hour and pelleted by centrifugation (15 min / 4 °C / 5000 × g). The supernatant was decanted into a fresh tube, supplemented with an additional 300 mg/mL ammonium sulphate (final saturation 75 %), incubated on a tube roller at 4 °C overnight, and pelleted by centrifugation (15 min / 4 °C / 12000 × g). The supernatant was discarded, and the pellet resuspended in 2 mL buffer, mixed thoroughly on ice, and centrifuged to remove suspended material (10 min / 4 °C / 20000 × g). Protein in the clarified supernatant was quantified on NanoDrop and approx. 2 mg of protein was transferred to a fresh tube, supplemented with 20 U bovine thrombin (Antibodies.com) and incubated on a tube roller in the dark at room temperature overnight. The digested GFP variant was then loaded onto a HiLoad 16/600 Superdex 75 prep-grade size exclusion chromatography column (GE Healthcare) on an Äkta Prime system (GE Healthcare). The buffer used for equilibration and elution was PBS pH 7.3. Peak fractions were collected, pooled, brought to 30 µM, flash frozen in liquid N_2 and stored at -80 °C.

nb24 and its variants pET-28b(+) vector carrying the gene for nb24 or one of its variants was transformed into SHuffle T7 bacteria as described, and selected for kanamycin resistance. Expression followed the general procedure with PBS as the buffer.

Affinity purification was conducted using an AmMag™ SA Plus Semi-automated System (Genscript), with PBS as buffer and PBS-washed His Mag Sepharose Excel magnetic beads (Cytiva) for capturing the his-tagged nanobodies. The nanobody was then loaded onto a Superdex 75 10/300 exclusion chromatography column (GE Healthcare) on an Äkta Pure system (GE Healthcare). The buffer used for equilibration and elution was 40 mM MES + 40 mM NaCl, pH 6.1. Peak fractions were collected, pooled, flash frozen in liquid N_2 and stored at -80 °C.

β2m Recombinant human beta-2 microglobulin was a gift from Dr Cristina Visentin and Prof. Stefano Ricagno (University of Milan), produced as previously described [11, 12].

Organic synthesis

symbol	*name*	*formula*	*mono-isotopic mass shift*
**1a**	Linker 1a / spent 1a-X	$C_{19}H_{14}FNO_3$	+323.10
**1a-rho**	Linker 1a Rhodamin B ester	$C_{47}H_{43}FN_3O_5^+$	+747.31[a]
**2a**	Linker 2a / spent 2a-X	$C_{19}H_{13}F_2NO_3$	+341.09
**2a-rho**	Linker 2a Rhodamin B ester	$C_{47}H_{42}F_2N_3O_5^+$	+765.30[a]
**2b**	Linker 2b / spent 2b-X	$C_{19}H_{17}F_2NO_3$	+345.12
**2b-rho**	Linker 2b Rhodamin B ester	$C_{47}H_{46}F_2N_3O_5^+$	+769.33[a]
**3c**	Linker 3c / spent 3c-X	$C_{21}H_{20}F_3NO_4$	+407.13
**3c-rho**	Linker 3c Rhodamin B ester	$C_{49}H_{49}F_3N_3O_6^+$	+831.34[a]
**3c-bio**	Linker 3c biotin ester	$C_{31}H_{34}F_3N_3O_6S$	+633.21
**3c-pho**	Linker 3c phosphate ester	$C_{21}H_{21}F_3NO_7P$	+487.10
**3c-sb**	Linker 3c 4-sulphobenzoate ester	$C_{28}H_{24}F_3NO_8S$	+591.12
**3c-pentynoate**	Linker 3c 4-pentynoate ester	$C_{26}H_{24}F_3NO_5$	+487.16
**3c-propargylglycine**	Linker 3c N-Fmoc-L-propargylglycine ester	$C_{41}H_{35}F_3N_2O_7$	+724.24
**3d**	Linker 3d / spent 3d-X	$C_{23}H_{24}F_3NO_5$	+451.16
**3d-rho**	Linker 3d Rhodamin B ester	$C_{51}H_{53}F_3N_3O_7^+$	+875.37[a]
**rho**	Rhodamin B amide	$C_{28}H_{29}N_2O_2^+$	+424.21[a]
**bio**	biotin amide	$C_{10}H_{14}N_2O_2S$	+226.08
**pho**	phosphoramidate	HO_3P	+79.96
**sb**	4-carbamoylbenzenesulphonate	$C_7H_4O_4S$	+183.98
**pentynoate**	4-pentynamide	C_5H_4O	+80.03
**propargylglycine**	N-Fmoc-L-propargylglycine amide	$C_{20}H_{15}NO_3$	+317.11

Table S6 | Calculated mass shifts for conjugations seen in this work. [a]Charged adduct masses are calculated as their neutral equivalent, e.g. 748.32-1.01 for **1a-rho**.

General methods

Where not mentioned otherwise, general procedures are for 1 mmol of compound.

Sonogashira coupling (1a-3, 2a-3, 3a-3 and 3b-3) Example for 2 mmol: Iodinated fluorophenol X_1 (2 mmol) was weighed in a round-bottom flask. Alkyne X_2 (3 mmol, 1.5 eq), 31 mg copper(I) iodide (0.16 mmol, 8 %mol) and 141 mg bis(triphenylphosphine) palladium(II) dichloride (0.2 mmol, 10 %mol) were added, dissolved in dry DMF (5 mL) and stirred under N_2 for 5 min. 558 µL triethylamine (405 mg, 4 mmol, 2 eq) was added and the mixture was stirred for X_3 (time) at RT. Purifications varied, see individual compounds.

Boc deprotection Boc-protected amine X_1 was weighed in a round-bottom flask and dissolved in anhydrous DCM at 0 °C for a final 0.1 M (10 mL). Trifluoroacetic acid (994 µL, 13 mmol, 13 eq) was added slowly and stirred on ice for 15 min. Reaction was then brought to room temperature and stirred for another X_2 (time). Reaction was monitored by TLC, and once completed, the solvent and residual acid were evaporated *in vacuo* as an azeotrope using toluene in 3–5 rounds.

HATU amide coupling (1a, 2a, 2b, 3c, 3d) 1.1 mmol acid X_1 (1.1 eq) and 475 mg HATU (1.25 mmol, 1.25 eq) were weighed into a dry round-bottom flask and dissolved in dry DMF (1.5 mL) at 0 °C. After 10 min, 436 µL *N*,*N*-diisopropylethylamine (2.5 mmol, 2.5 eq) were added dropwise and the solution was stirred for X_2 (temperature and time). In a separate vial, amine X_3 (1.0 mmol) was dissolved in dry DMF (1.5 mL), then added dropwise to the stirred reaction mixture. The reaction was brought to room temperature and stirred for X_4 (time). The reaction mixture was then diluted 20 times in EtOAc, acidified by washing twice with 3M HCl and then washed twice with H_2O and twice with brine. The organic layer was dried over $MgSO_4$, filtered and concentrated *in vacuo*, then purified by flash chromatography on silica gel (mobile phase X_5).

EDC esterification (1a-rho, 2a-rho, 2b-rho, 3c-rho, 3c-bio, 3d-rho) Example for 100 µmol: acid X_1 (2.5 eq), 23 mg *N*-(3-Dimethylaminopropyl)-*N′*-ethylcarbodiimide hydrochloride (EDC·HCl) (120 µmol, 1.2 eq) and 3.1 mg 4-dimethylaminopyridine (25 µmol, 0.25 eq) were weighed in an oven-dried round-bottom flask and dissolved in dry DCM (1.5 mL) at 0 °C. In a separate vial, X_2 was dissolved in a mixture of dry DMF (90 µL) and dry DCM (1.5 mL), then added dropwise over the course of 30 min to the stirred reaction mixture. The reaction was brought to room temperature and stirred for 4 h. The reaction mixture was then diluted 10-fold in DCM, washed once with H_2O then separated; aqueous phase was extracted with DCM, the two organic phases were joined, washed twice with H_2O and once with brine, dried over $MgSO_4$, filtered and dried *in vacuo*. The residue was purified by flash chromatography on silica gel (mobile phase X_3)

Alkyne reduction (2b-3, 3c-4, 3d-4) Alkyne X_1 was weighed in an oven-dried 100 mL round-bottom flask. X_2 (mass) palladium on carbon (1:10 w/w) was added and the flask was flushed with N_2. The mixture was suspended in anhydrous methanol (20 mL)

and stirred at room temperature for 10 min. 5–10 L of hydrogen gas were bubbled into the solution while stirring and venting with a needle, and the flask was then stirred under H_2 atmosphere for X_3 (time). The reaction was quenched by bubbling nitrogen gas as above. Once safe to open, the reaction mixture was filtered through 2 cm of celite, and washed several times with methanol. The solvent was then removed *in vacuo* to dryness, and products were used without further purification.

Linker 1a

Reaction scheme: Fig. S13

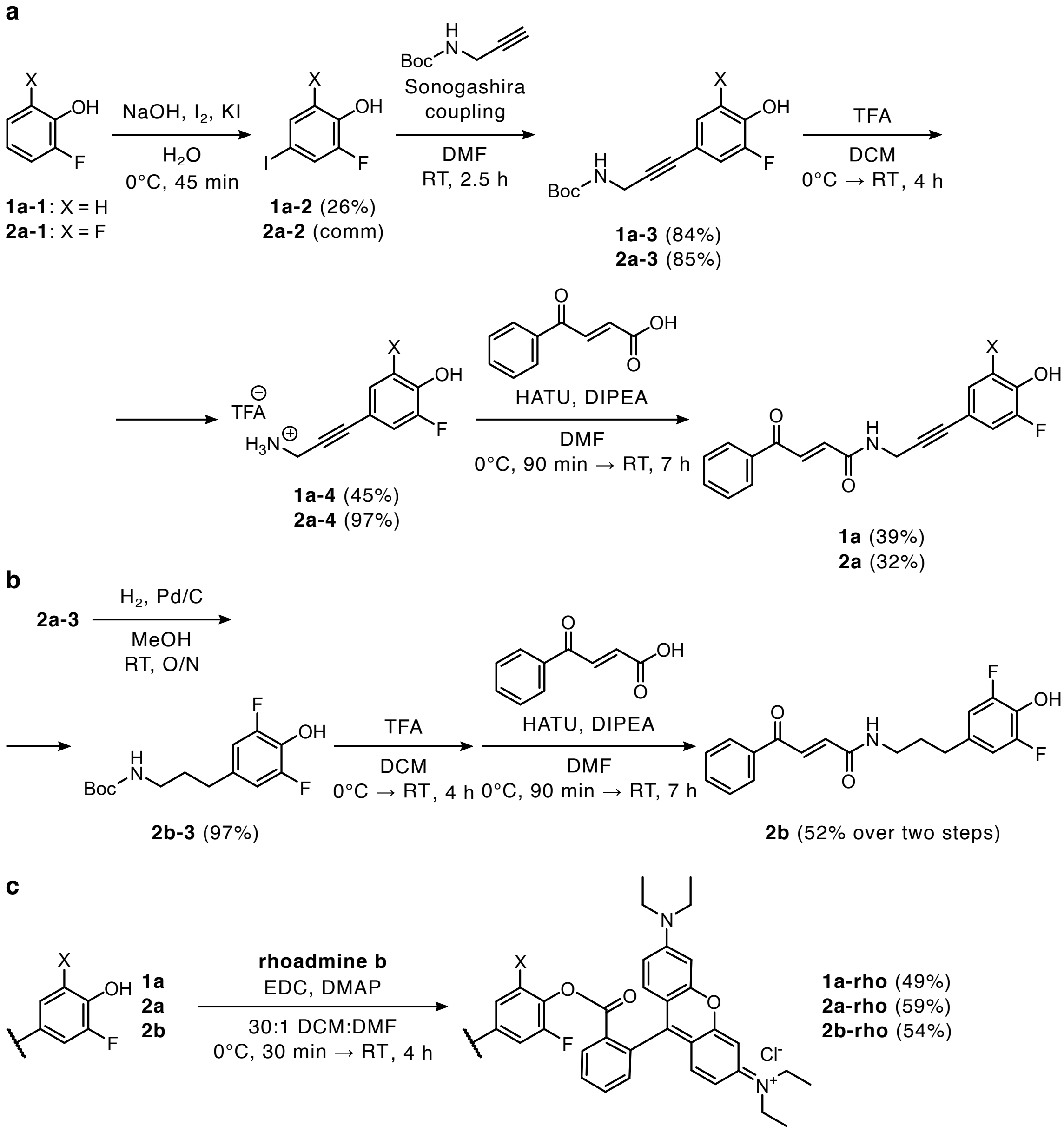

Figure S13 | Synthetic routes to groups 1 and 2 linkers. **(a)** Synthetic route to **1a** and **2a**. **(b)** Synthetic route to **2b**. **(c)** Loading rhodamine as a payload. **comm** commercially available; **TFA** trifluoroacetic acid; **DCM** dichloromethane; **DMF** dimethylformamide; **DIPEA** diisopropylethylamine; **EDC** ethyl(dimethylaminopropyl)carbodiimide; **DMAP** dimethylaminopyridine.

2-fluoro-4-iodophenol (1a-2): 238.0 g/mol 1.28 g sodium hydroxide (32 mmol, 2 eq) was dissolved in H_2O (40 mL, 800 mM) and cooled to 0 °C. To a stirred solution of 2.54 g iodine (10 mmol, 0.625 eq) and 1.66 g potassium iodide (10 mmol, 0.625 eq) in H_2O (40 mL) at 0 °C was added dropwise 1.79g **1a** (16 mmol) and stirred for 10 min, after which the cold sodium hydroxide solution was added slowly and stirred for 45 min at 0 °C. The reaction was quenched with a large excess of ammonium chloride (10 g) and 3.16 g sodium thiosulphate (20 mmol, 1.25 eq) was added to reduce any remaining iodine. The reaction mixture was acidified with HCl, extracted with DCM (3 × 25 mL) then washed with H_2O (3 × 100 mL) and with brine (100 mL). The organic layer was dried over $MgSO_4$, filtered and concentrated *in vacuo*, then purified by flash chromatography on silica gel (100 % DCM).

Yields 992 mg of **1a-2** (4.17 mmol, 26 %).

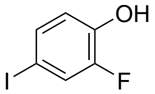

1a-2
C_6H_4FIO

^{1}H-NMR ($CDCl_3$, δ/ppm): 7.40 (1H, dd, J = 9.7, 2.0 Hz, H5), 7.34 (1H, dt, J = 8.5, 1.4 Hz, H3), 6.77 (1H, t, J = 8.7 Hz, H6), 5.13 (1H, s, OH); ^{19}F-NMR ($CDCl_3$, δ/ppm): -138.0.

4-(*N*-Boc-3-aminoprop-1-yn-1-yl)-2-fluorophenol (1a-3): 265.28 g/mol Sonogashira coupling of 3 mmol with X_1 = 714 mg **1a-2**; X_2 = 698 mg *N*-Boc-propargylamine; X_3 = 2 h 30 min. The solution was basified with 50 mL 10 % sodium hydroxide solution and stirred for 10 min. The solution was then diluted with 450 mL H_2O and impurities were extracted using DCM. The aqueous layer was acidified to pH 1–2 using HCl and the resulting protonated product was extracted with EtOAc (2 portions). This organic layer was then washed with H_2O (2 portions) and brine (2 portions), dried over $MgSO_4$, filtered and concentrated *in vacuo*. The residue was dry-loaded on silica gel and purified by flash chromatography (4:1 PE:EtOAc).

Yields 668 mg of **1a-3** (2.52 mmol, 84 %) as viscous liquid.

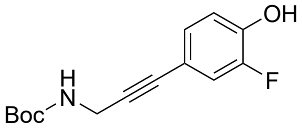

1a-3
$C_{14}H_{16}FNO_3$

^{1}H-NMR ($CDCl_3$, δ/ppm): 7.10 (1H, dd, J = 11.1, 1.8 Hz, H5), 7.06 (1H, dt, J = 8.5, 1.4 Hz, H3), 6.91 (1H, t, J = 8.6 Hz, H6), 5.96 (1H, s, OH), 4.80 (1H, s, NH), 4.11 (2H, s, CH_2), 1.47 (9H, s, Boc); ^{19}F-NMR ($CDCl_3$, δ/ppm): -139.7.

3-(3-fluoro-4-hydroxyphenyl)prop-2-yn-1-aminium trifluoroacetate (1a-4): 279.19 g/mol Boc deprotection of 1.6 mmol with X_1 = 425 mg **1a-3**; X_2 = 4 h. Loaded on silica gel and purified by flash chromatography (85:15 DCM:MeOH).

Yields 256 mg at 78 %wt purity of **1a-4** (199 mg pure product + 57 mg excess TFA) (0.71 mmol, 45 %), used without further purification.

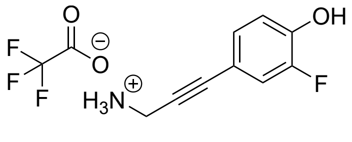

1a-4
$C_9H_9FNO^+ \cdot C_2F_3O_2^-$

^{1}H-NMR (MeOD, δ/ppm): 7.74 (1H, dd, J = 10.1, 1.6 Hz, H5), 7.02 (1H, dt, J = 8.4, 1.4 Hz, H3), 6.89 (1H, t, J = 8.6 Hz, H6), 3.99 (2H, s, CH_2); ^{19}F-NMR (MeOD, δ/ppm): -138.5, -77.0 (TFA).

Linker 1a: 323.32 g/mol HATU amide coupling of 0.52 mmol with X_1 = 119 mg trans-3-benzoylacrylic acid (0.66 mmol, 1.25 eq); X_2 = 0 °C for 90 min; X_3 = 167 mg **1a-4** (214 mg at 78 %wt); X_4 = 7 h; X_5 = 30:1 DCM:MeOH.

Yields 65 mg of **1a** (0.2 mmol, 39 %).

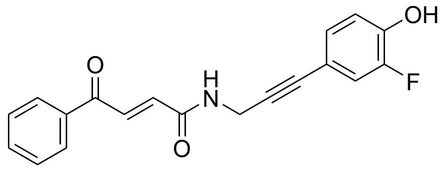

1a
$C_{19}H_{14}FNO_3$

^{1}H-NMR (MeOD, δ/ppm): 8.04 (2H, d, J = 7.5), 7.92 (1H, d, J = 15.3), 7.67 (1H, t, J = 7.5), 7.56 (2H, t, J = 7.7), 7.12 (1H, dd, J = 11.6, 1.5), 7.07 (1H, dt, J = 8.8, 1.0 Hz), 7.03 (1H, d, J = 15.3), 6.85 (1H, t, J = 8.7 Hz), 4.30 (2H, s); ^{13}C-NMR (176 MHz, DMSO-d_6, δ/ppm): 189.7, 163.2, 151.1, 149.7, 146.0, 145.9, 136.5, 135.6, 133.8, 132.6, 129.0, 128.7, 128.6, 128.6, 119.1, 119.0, 118.0 118.0, 112.8, 112.7, 84.7, 81.4, 29.1. ^{19}F-NMR (MeOD, δ/ppm): -138.9. LC-MS$^-$: calc. for $C_{19}H_{14}FNO_3$ 323.0964, found [M-H]$^-$ 322.0891 (diff 2.03 ppm). Full spectra and assignments appear in Fig. S14.

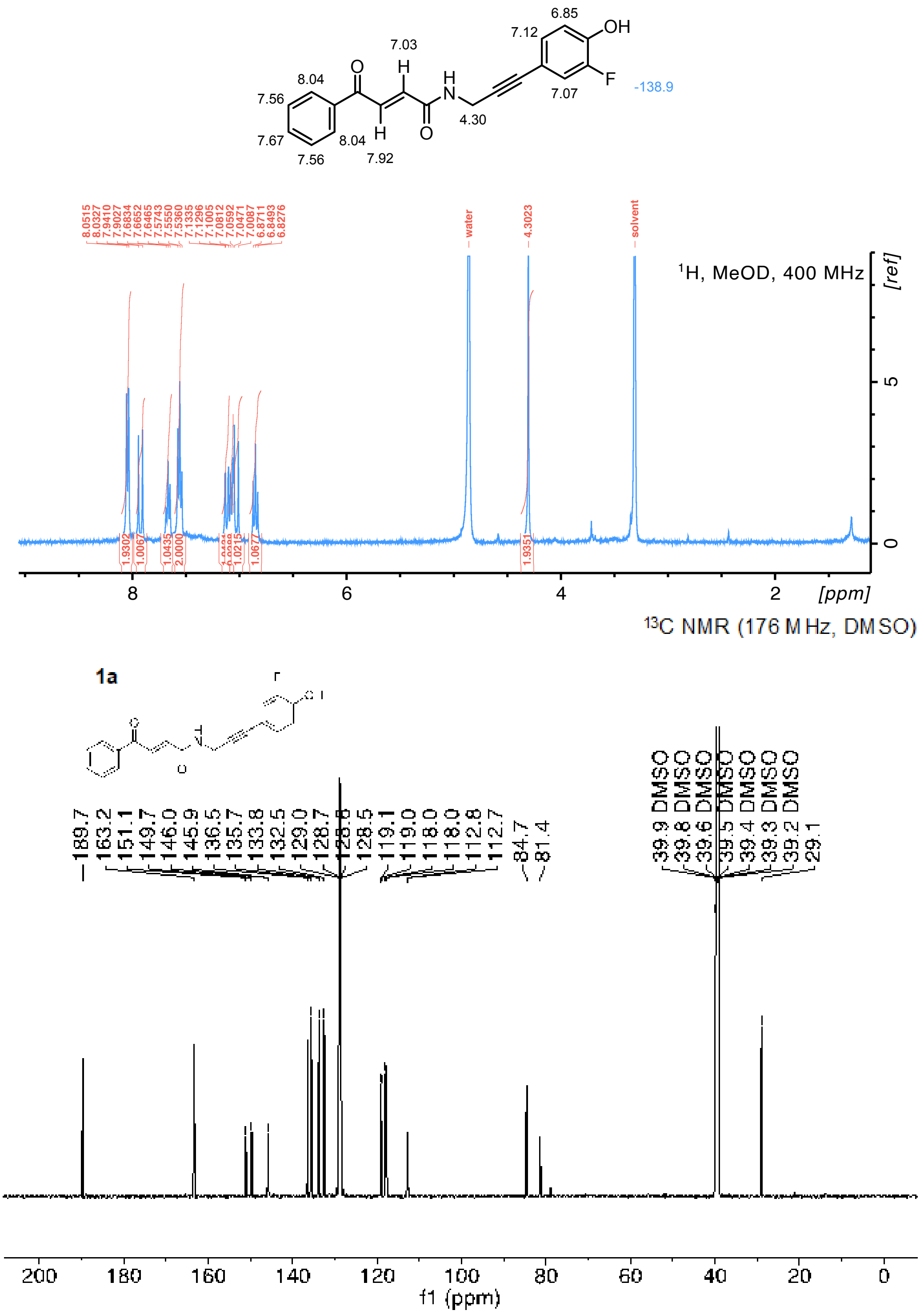

Figure S14 | Linker 1a NMR spectra

1a-rho: 784.33 g/mol **EDC esterification** of 85 µmol with X_1 = 100 mg Rhodamine B; X_2 = 27 mg **1a**; X_3 = 92:8 DCM:MeOH.

Yields 33 mg of **1a-rho** (42 µmol, 49 %).

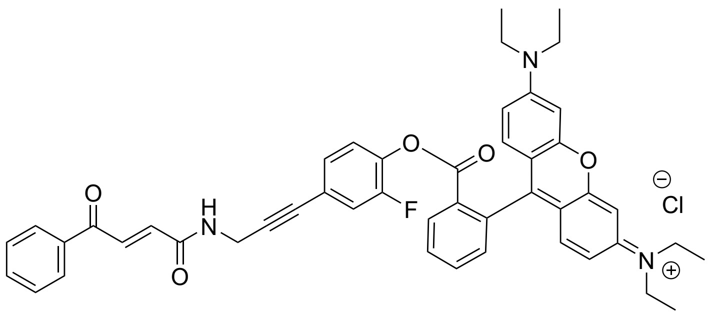

1a-rho

$C_{47}H_{43}FN_3O_5^+ \cdot Cl^-$

^{1}H-NMR ($CDCl_3$, δ/ppm): see Fig S15; ^{13}C-NMR (176 MHz, $CDCl_3$, δ/ppm): 190.6, 164.9, 162.6, 157.9, 157.8, 155.7, 152.4, 137.5, 137.4, 137.1, 136.0, 134.3, 134.2, 133.4, 133.1, 132.1, 131.2, 130.8, 130.7, 129.2, 128.8, 128.6, 128.5, 128.4, 123.2, 123.1, 123.0, 120.2, 120.0, 114.4, 113.6, 96.5, 87.5, 80.4, 46.3, 29.8, 12.8; ^{19}F-NMR ($CDCl_3$, δ/ppm): -128.5. LC-MS$^+$: calc. for $C_{47}H_{43}FN_3O_5$ 748.3181, found $[M]^+$ 748.3387 (diff 27.5 ppm).

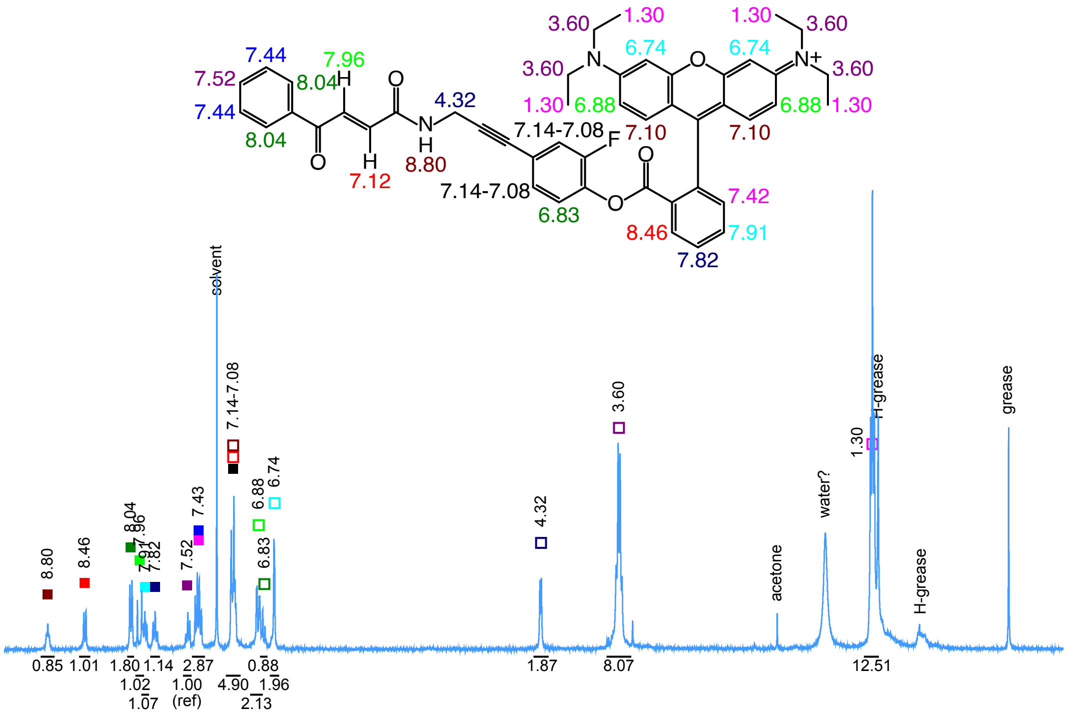

¹³C NMR (176 MHz, CDCl₃)

1a-rho

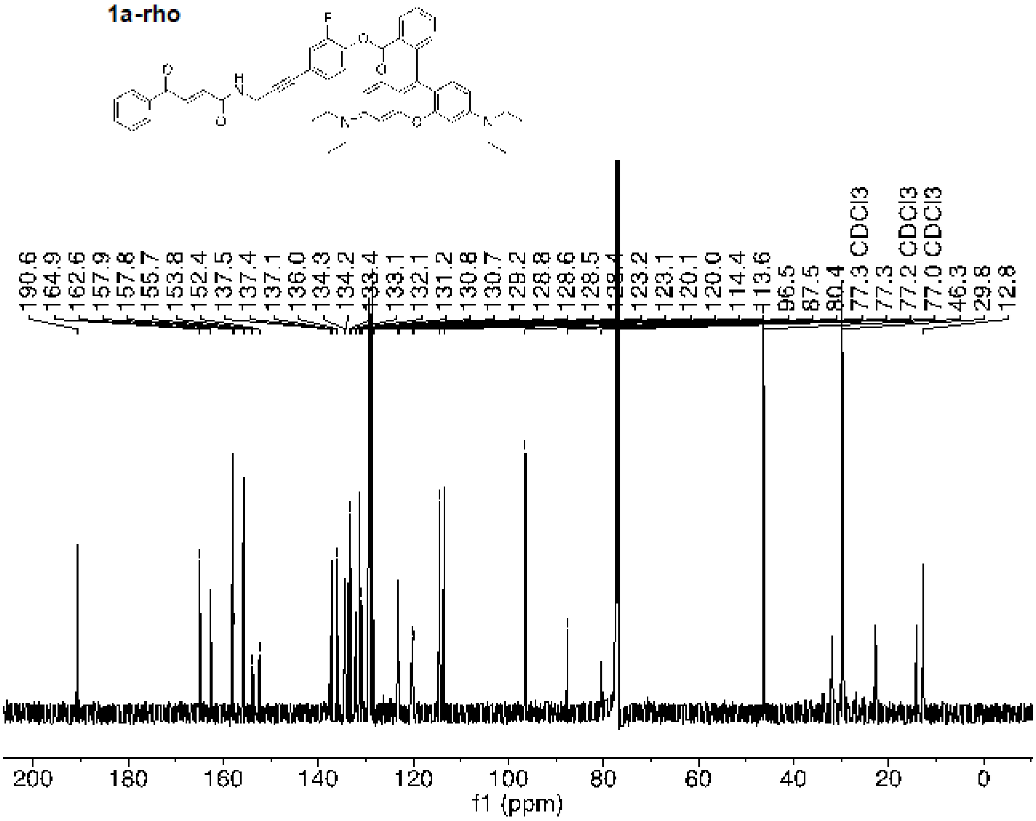

Figure S15 | 1a-rho NMR spectra

Linker 2a

Reaction scheme: Fig. S13

4-(*N*-Boc-3-aminoprop-1-yn-1-yl)-2,6-difluorophenol (2a-3): 283.27 g/mol **Sonogashira coupling** with X_1 = 512 mg **2a-2**; X_2 = 466 mg *N*-Boc-propargylamine; X_3 = 3 h. Solvent was removed *in vacuo* as an azeotrope using toluene (7 rounds of dilution and evaporation), and the residue was dry-loaded on silica gel and purified by flash chromatography (3:1 PE:EtOAc).

Yields 483 mg of **2a-3** (1.7 mmol, 85 %).

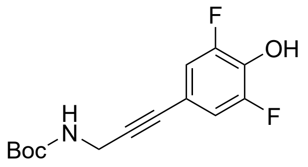

2a-3
$C_{14}H_{15}F_2NO_3$

^{1}H-NMR ($CDCl_3$, δ/ppm): 6.90 (2H, d, J = 8.3 Hz, H3 and H5), 6.25 (1H, s-br, OH), 4.82 (1H, s-br, NH), 4.10 (2H, s, CH_2), 1.47 (9H, s, Boc); ^{19}F-NMR ($CDCl_3$, δ/ppm): -134.9.

3-(3,5-difluoro-4-hydroxyphenyl)prop-2-yn-1-aminium trifluoroacetate (2a-4): 297.18 g/mol **Boc deprotection** with X_1 = 283 mg **2a-3**; X_2 = 2 h 30 min. Loaded on silica gel and purified by flash chromatography (85:15 DCM:MeOH).

Yields 346mg of **2a-4** at 83 %wt purity (288 mg pure product + 58 mg excess TFA) (0.97 mmol, 97 %) as a white crystalline solid, used without further purification.

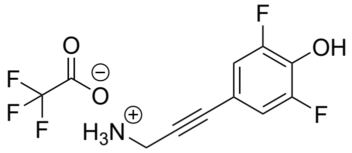

2a-4
$C_9H_8F_2NO^+ \cdot C_2F_3O_2^-$

^{1}H-NMR (MeOD, δ/ppm): 7.05 (2H, dd, J = 7.1, 1.7 Hz, H3 and H5), 3.99 (2H, s, CH_2); ^{19}F-NMR (MeOD, δ/ppm): -135.0, -77.0 (TFA).

Linker 2a: 341.31 g/mol **HATU amide coupling** of 0.4 mmol with X_1 = 70 mg trans-3-benzoylacrylic acid (0.4 mmol, 1.0 eq); X_2 = 0 °C for 1 hour; X_3 = 119 mg **2a-4**; X_4 = 2 h; X_5 = 64:36 PE:EtOAc.

Yields 44 mg of **2a** (129 µmol, 32 %).

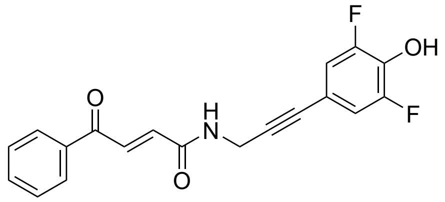

2a
$C_{19}H_{13}F_2NO_3$

^{1}H-NMR (MeOD, δ/ppm): 8.04 (2H, d, J = 7.2 Hz), 7.92 (1H, d, J = 15.3 Hz), 7.66 (1H, t, J = 7.4 Hz), 7.56 (2H, t, J = 7.6 Hz), 7.02 (1H, d, J = 15.3 Hz), 6.98 (2H, s), 4.30 (2H, s); ^{13}C-NMR (176 MHz, $CDCl_3$, δ/ppm): 189.7, 163.2, 152.6, 152.5, 151.2, 151.1, 136.5, 135.6, 135.3, 135.2, 135.1, 133.8, 132.6, 129.0, 128.7, 115.4, 115.4, 115.3, 115.3, 115.2, 115.2, 111.8, 111.7, 111.6, 85.8, 80.4, 78.0, 39.52, 28.98; ^{19}F-NMR (MeOD, δ/ppm): -135.5. LC-MS$^-$: calc. for $C_{19}H_{12}F_2NO_3$ 341.0858, found [M-H]$^-$ 340.0816 (diff 1.51 ppm). Full spectra and assignments appear in Fig. S16.

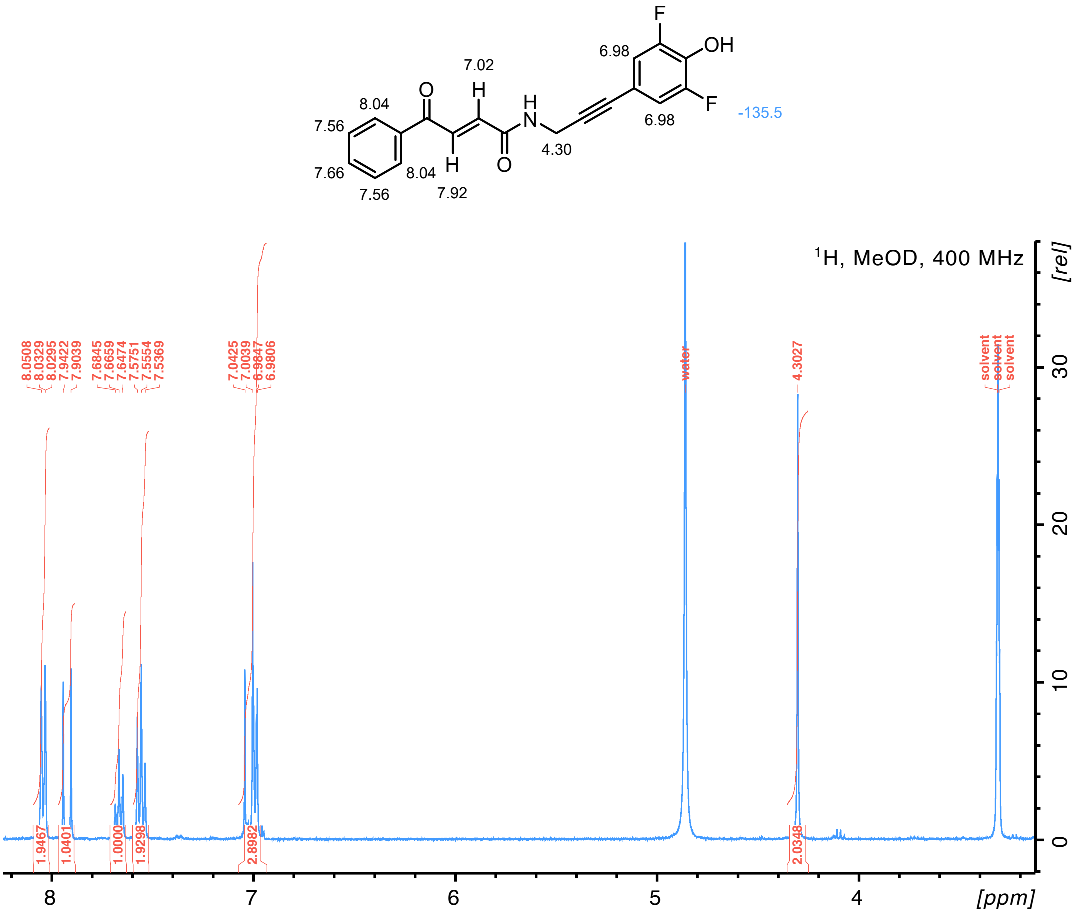

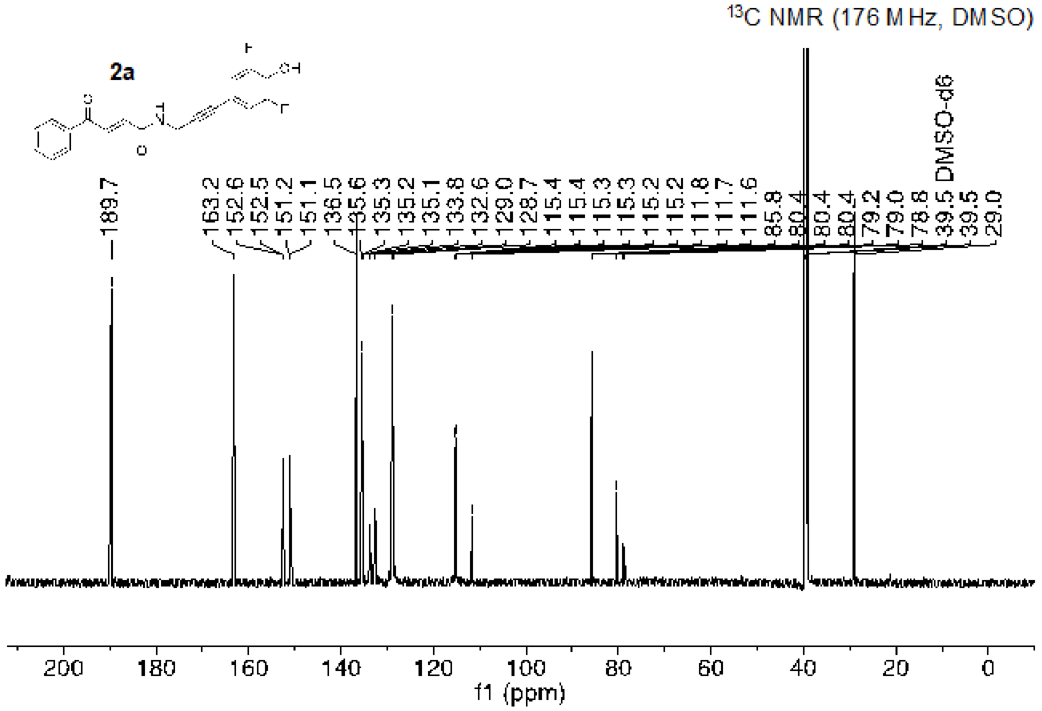

Figure S16 | Linker 2a NMR spectra

2a-rho: 802.32 g/mol **EDC esterification** of 70 µmol with X_1 = 81 mg Rhodamine B; X_2 = 25 mg **2a**; X_3 = 9:1 DCM:MeOH.

Yields 33 mg of **2a-rho** (41 µmol, 59 %).

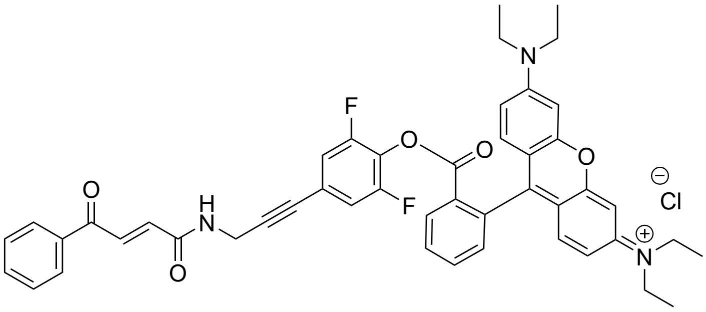

2a-rho
$C_{47}H_{42}F_2N_3O_5^+ \cdot Cl^-$

^{1}H-NMR ($CDCl_3$, δ/ppm): see Fig S17; ^{13}C-NMR (176 MHz, $CDCl_3$, δ/ppm): 190.7, 165.1, 161.9, 157.9, 157.3, 155.7, 155.0, 155.0, 153.6, 153.6, 137.1, 136.1, 134.4, 134.3, 133.3, 133.1, 132.1, 131.2, 130.9, 130.8, 129.2, 128.8, 128.2, 126.7, 122.9, 115.9, 115.8, 115.7, 115.7, 114.4, 113.5, 96.5, 89.1, 79.4, 46.3, 29.9, 12.7; ^{19}F-NMR ($CDCl_3$, δ/ppm): -126.4. LC-MS$^+$: calc. for $C_{47}H_{42}F_2N_3O_5$ 766.3080, found $[M]^+$ 766.3056 (diff 4.30 ppm).

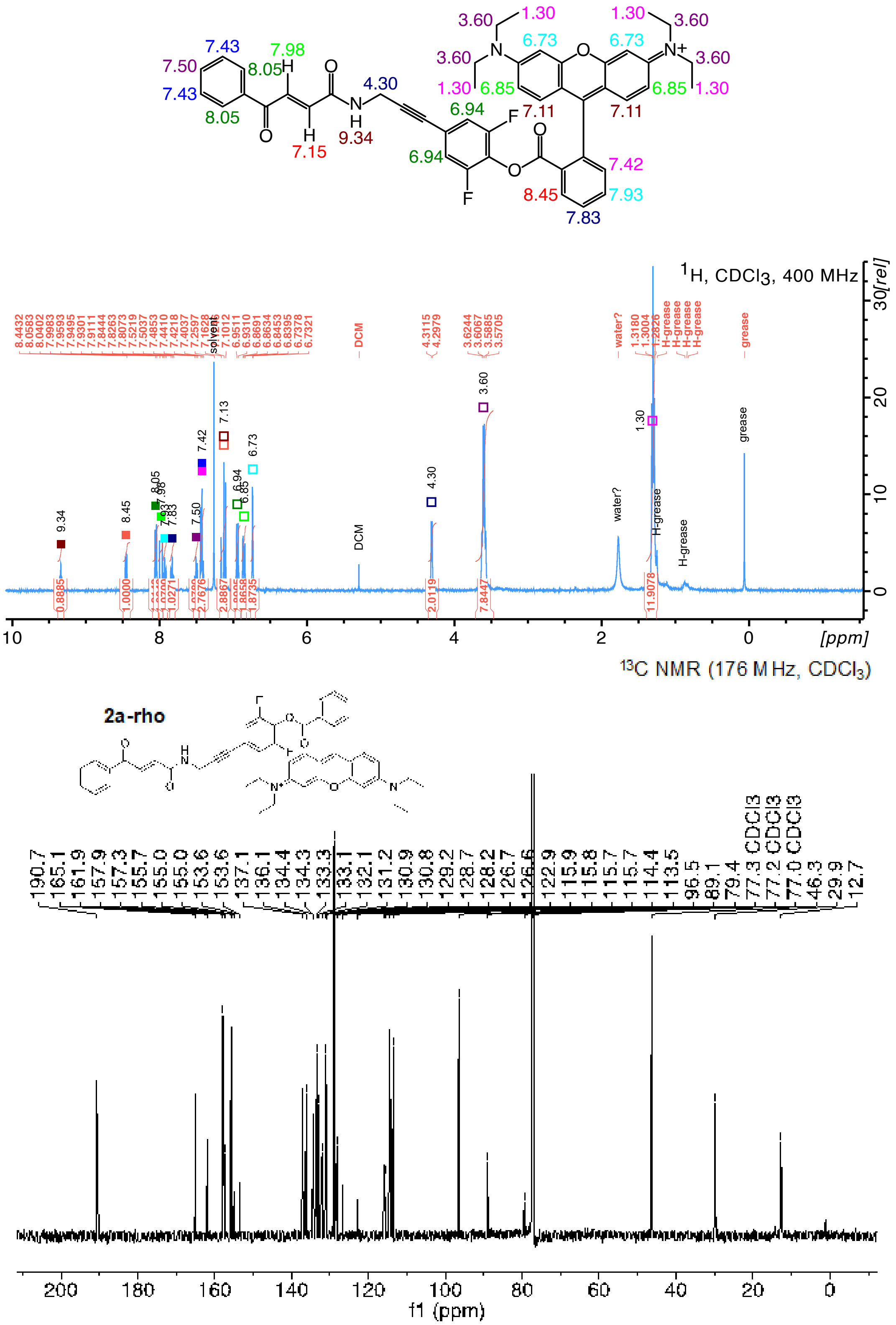

Figure S17 | 2a-rho NMR spectra

Linker 2b

Reaction scheme: Fig. S13

4-(*N*-Boc-3-aminopropan-1-yl)-2,6-difluorophenol (2b-3): 287.31 g/mol Alkyne reduction of 1.63 mmol with X_1 = 462 mg **2a-3**; X_2 = 46 mg; X_3 = 7 h.

Yields 454 mg of **2b-3** (1.58 mmol, 97 %).

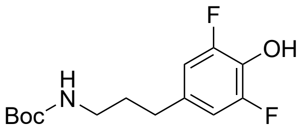

2b-3
$C_{14}H_{19}F_2NO_3$

^{1}H-NMR ($CDCl_3$, δ/ppm): 6.70 (2H, d, J = 8.6 Hz, H3 and H5), 5.65 (1H, s-br, OH), 4.55 (1H, s-br, NH), 3.13 (2H, d, J = 6.4 Hz, -HN-CH_2-), 2.54 (2H, t, J = 7.7 Hz, Ph-CH_2-), 1.75 (2H, quintet, J = 7.4 Hz, -CH_2-CH_2-CH_2-), 1.44 (9H, s, Boc); ^{19}F-NMR ($CDCl_3$, δ/ppm): -135.5.

Linker 2b: 345.35 g/mol Boc deprotection of 1.78 mmol with X_1 = 512 mg **2b-3**; X_2 = 2 h. Yields 89 %.

Followed by **HATU amide coupling** of 1.2 mmol, X_1 = 238 mg trans-3-benzoylacrylic acid (1.35 mmol, 1.125 eq); X_2 = 0 °C for 90 min; X_3 = 362 mg **2a-4**; X_4 = 7 h; X_5 = 64:36 PE:EtOAc.

Yields 215 mg of **2b** (0.62 mmol, 52 % over two steps).

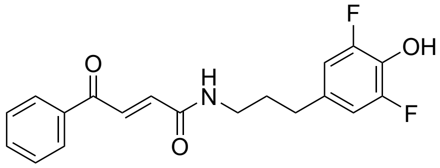

2b
$C_{19}H_{17}F_2NO_3$

^{1}H-NMR (MeOD, δ/ppm): 8.03 (2H, d, J = 7.4 Hz, Ph-*o*), 7.87 (1H, d, J = 15.3, alkene Ph side), 7.66 (1H, t, J = 7.4 Hz, Ph-*p*), 7.55 (2H, t, J = 7.8 Hz, Ph-*m*), 7.01 (1H, d, J = 15.3, alkene NH side), 6.78 (2H, d, J = 9.2 Hz, H3 and H5), 3.32 (2H, s, -HN-CH_2-), 2.59 (2H, t, J = 7.7 Hz, Ph-CH_2-), 1.85 (2H, quintet, J = 7.4 Hz, -CH_2-CH_2-CH_2-), 1.44 (9H, s, Boc); ^{13}C-NMR (176 MHz, DMSO-d_6, δ/ppm): 189.9, 163.5, 152.9, 152.3, 151.5, 151.5, 136.7, 136.6, 133.7, 132.5, 132.5, 132.4, 131.7, 131.5, 131.4, 131.3, 129.0, 128.6, 111.6, 111.6, 111.5, 111.5, 38.3, 31.3, 30.3; ^{19}F-NMR (MeOD, δ/ppm): -136.2. LC-MS$^-$: calc. for $C_{19}H_{17}F_2NO_3$ 345.1180, found [M-H]$^-$ 344.1107 (diff 0.97 ppm). Also see Figure S18.

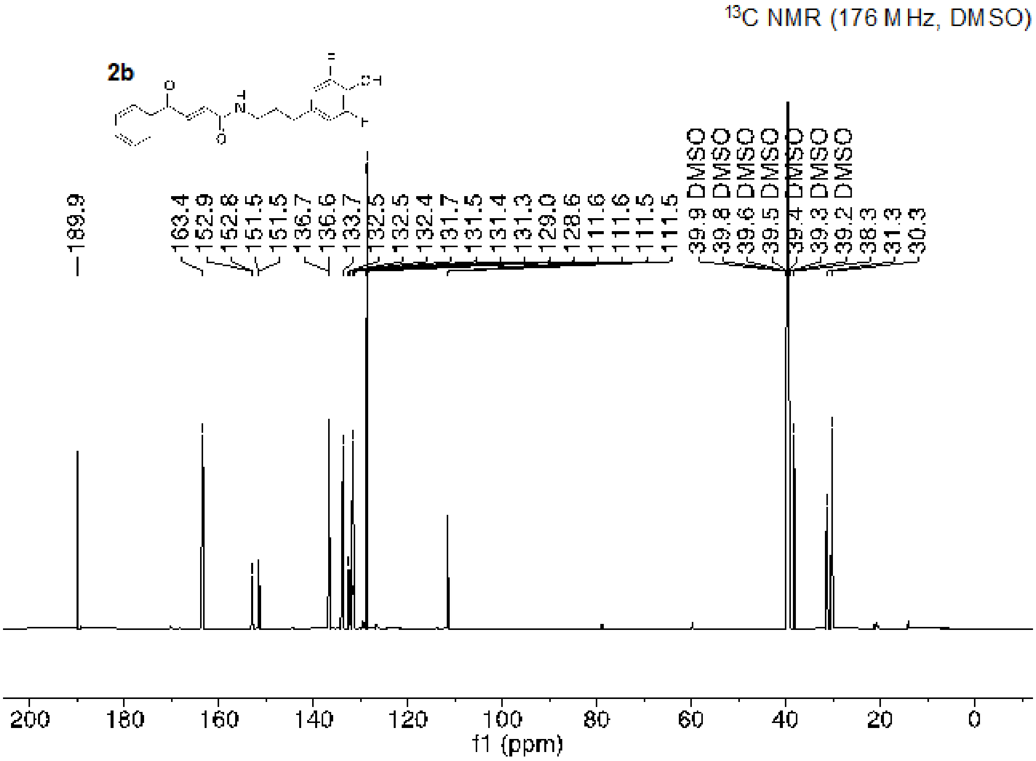

Figure S18 | 2b NMR spectra

2b-rho: 806.35 g/mol **EDC esterification** with X_1 = 120 mg Rhodamine B; X_2 = 35 mg **2b**; X_3 = 92:8 DCM:MeOH.

Yields 44 mg of **2b-rho** (54 µmol, 54 %).

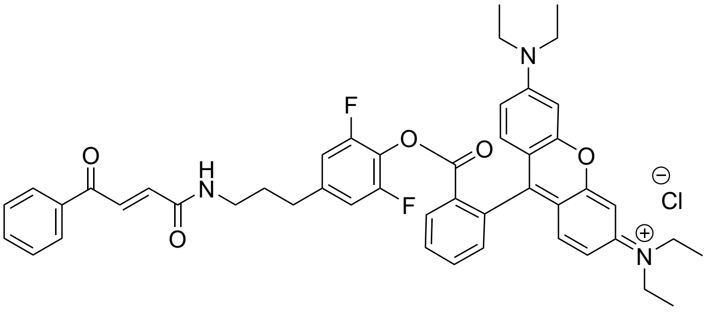

2b-rho
$C_{47}H_{46}F_2N_3O_5^+ \cdot Cl^-$

^{1}H-NMR ($CDCl_3$, δ/ppm): see Fig S19; ^{13}C-NMR (176 MHz, $CDCl_3$, δ/ppm): 191.0, 165.3, 162.4, 157.9, 157.5, 155.6, 155.1, 153.7, 142.9, 137.2, 137.0, 134.2, 134.1, 133.2, 132.3, 132.1, 131.2, 130.8, 130.6, 129.2, 128.7, 114.3, 113.6, 112.3, 112.2, 96.5, 46.3, 39.2, 32.0, 30.4, 12.7; ^{19}F-NMR ($CDCl_3$, δ/ppm): -127.5. LC-MS$^+$: calc. for $C_{47}H_{46}F_2N_3O_5$ 770.3400, found $[M]^+$ 770.3546 (diff 19.0 ppm).

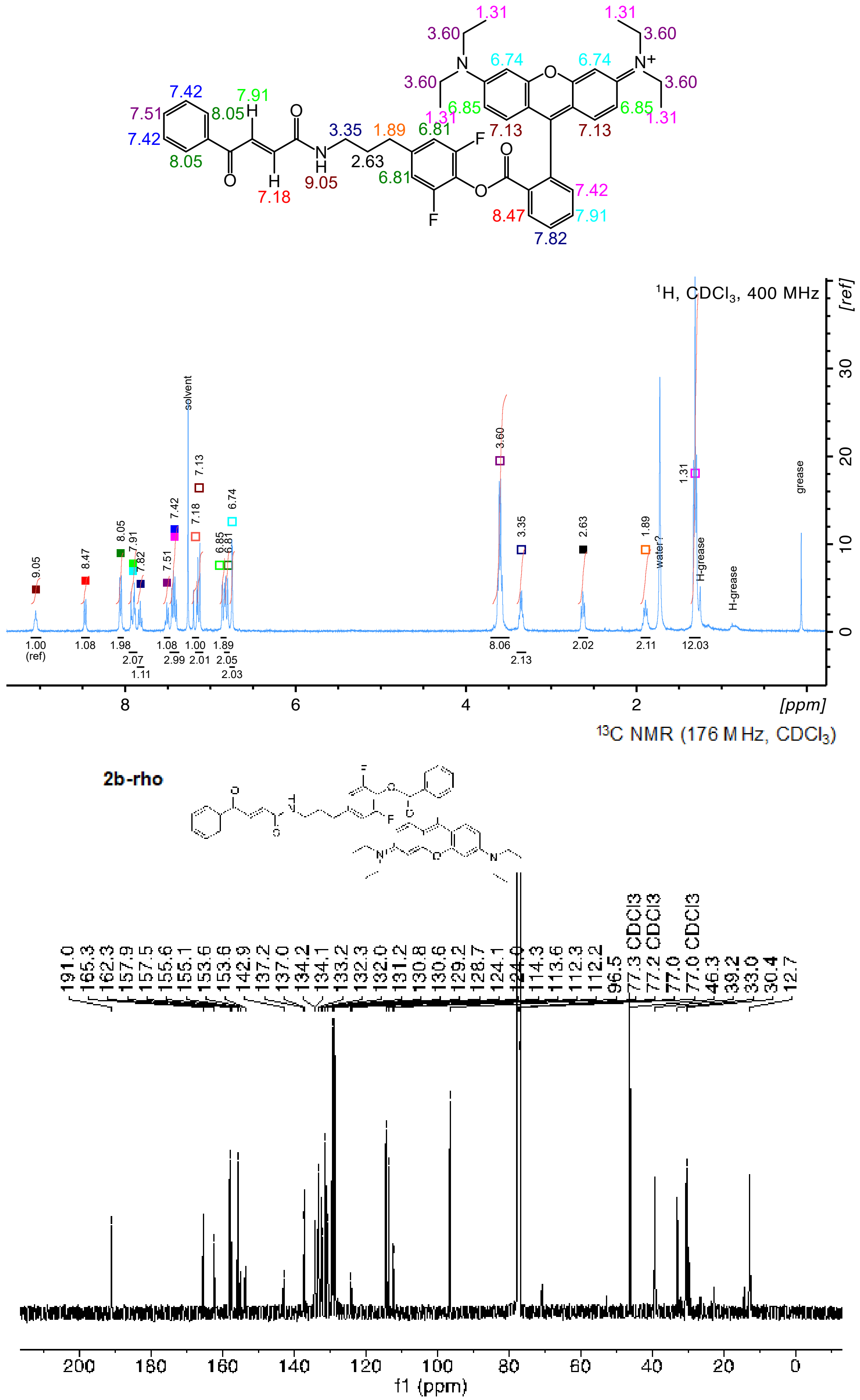

Figure S19 | 2b-rho NMR spectra

Group 3 linkers

The spacers used in linkers **3c** and **3d** were not commercially available with a terminal alkyne, but published syntheses were available and produced good yields (Fig. S20a). Additionally, the iodination of the fluorophenols had to be changed from the one used for 2-fluorophenol; a modified reaction optimised for 2,3,6-trifluorophenol led to a high yield at very high purity (Fig. S20b, first step) of this previously-unpublished compound.

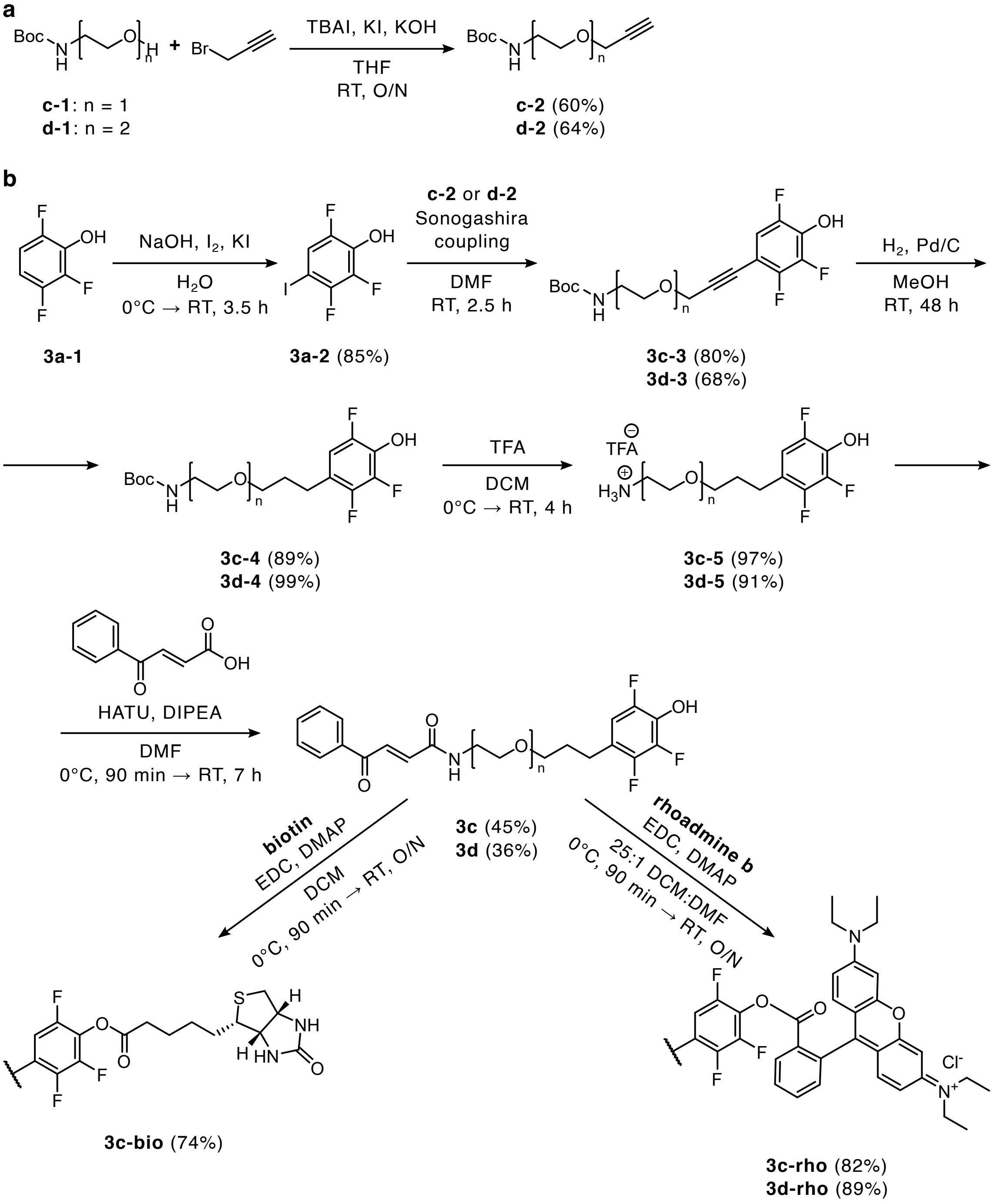

Figure S20 | Synthetic routes to group 3 linkers. **(a)** Route to spacer precursors **c-2** and **d-2**. **(b)** Route to linkers **3c** and **3d**, and on to **3c-bio**, **3c-rho** and **3d-rho**. **TBAI** tetrabutylammonium iodide; **THF** tetrahydrofuran.

Linker 3c

Reaction scheme: Fig. S20

***N*-Boc-2-(prop-2-yn-1-yloxy)ethan-1-amine (c-2): 199.25 g/mol** Williamson ether synthesis, based on refs [13, 14]. 3.23 g *N*-Boc-ethanolamine (20 mmol) was weighed in a round-bottom flask with 2.98 g of 80 %wt propargyl bromine in toluene stabilised with MgO (2.38 g net weight, 20 mmol, 1 eq), and then dissolved in tetrahydrofuran (20 mL). 476 mg tetra-(*n*-butyl)ammonium iodide (TBAI) (1.2 mmol, 0.06 eq), 498 mg potassium iodide (3 mmol, 0.15 eq) and 1.12 g potassium hydroxide (20 mmol, 1 eq) were added and the reaction mixture was stirred at room temperature O/N. On completion, the mixture was filtered and the filtrate was concentrated *in vacuo*, diluted with H_2O (20 mL) and extracted twice with EtOAc (50 mL). The organic phase was concentrated *in vacuo*, loaded on silica gel and purified by flash chromatography (5:1 PE:EtOAc).

Yields 2.39 g of **c-2** (12 mmol, 60 %) as a colourless oil.

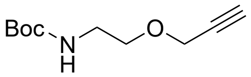

c-2
$C_{10}H_{17}NO_3$

^{1}H-NMR ($CDCl_3$, δ/ppm): 4.89 (1H, s-br, NH), 4.15 (2H, s, -CH_2-C), 3.58 (2H, t, J = 5.1 Hz, -O-CH_2-CH_2-), 3.34 (2H, q, J = 4.8 Hz, -O-CH_2-CH_2-), 2.44 (1H, s, CH), 1.44 (9H, s, Boc); ^{13}C-NMR (176 MHz, $CDCl_3$, δ/ppm): 156.0, 79.5, 79.3, 74.8, 7.15, 58.3, 40.4, 28.5. LC-MS$^+$: calc. for $C_{10}H_{17}NO_3$ 199.1202, found $[M+Na]^+$ 222.1096, (diff 2.13 ppm). Also see Figure S21.

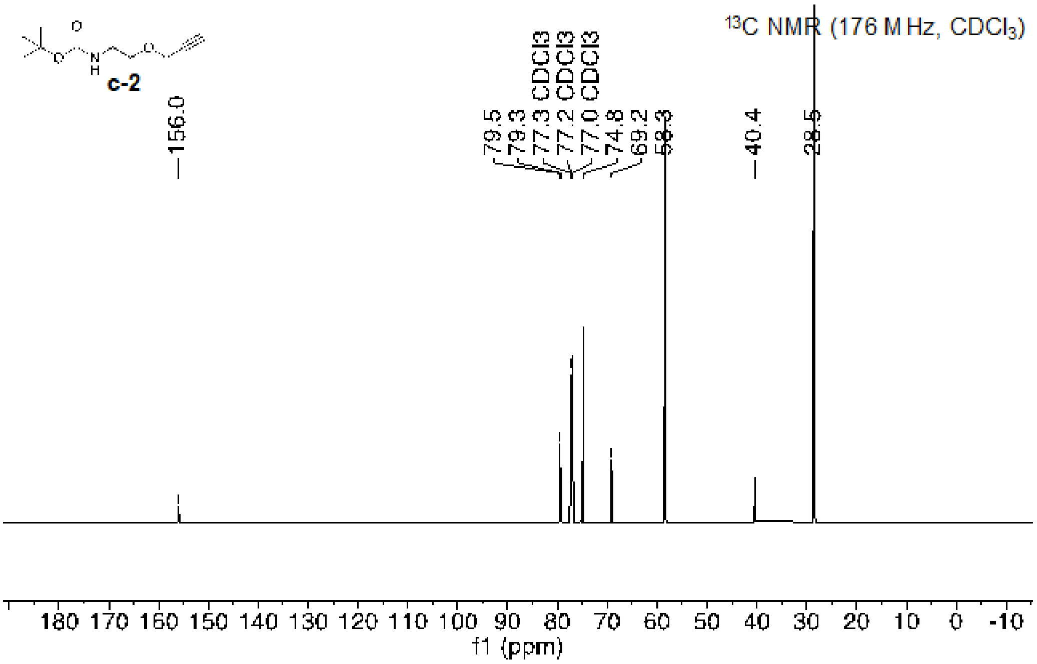

Figure S21 | c-2 NMR spectra

2,3,6-trifluoro-4-iodophenol (3a-2): 273.98 g/mol 540 mg of sodium hydroxide (13.5 mmol, 2 eq) was dissolved in H_2O (17 mL for final 800 mM) and cooled to 4 °C. To a stirred solution of 1.71 g iodine (6.75 mmol, 1 eq) and 1.12 g potassium iodide (6.75 mmol, 1 eq) in H_2O (17 mL) at 0 °C was added dropwise 1 g 2,3,6-trifluorophenol (6.75 mmol) and stirred for 10 min, after which the cold sodium hydroxide solution was added slowly and the flask was brought to room temperature and stirred for 3.5 hours. The reaction was quenched with saturated ammonium chloride and 1.34 g (8.45 mmol, 1.25 eq) sodium thiosulphate was added to reduce any remaining iodine. The reaction mixture was then carefully acidified with HCl, extracted with DCM (3 × 40 mL) then washed with H_2O (2 × 100 mL) and 100 mL brine. The organic layer was dried over $MgSO_4$, filtered and concentrated *in vacuo.*

Yields 1.56 g of **3a-2** (5.73 mmol, 85 %) at very high purity with no need for further purification.

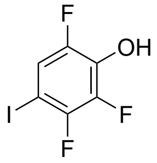

3a-2
$C_6H_2F_3IO$

^{1}H-NMR ($CDCl_3$, δ/ppm): 7.28 (1H, ddd, J = 6.6, 5.2, 2.5 Hz, H5), 5.54 (1H, s-br, OH); ^{13}C-NMR ($CDCl_3$, δ/ppm): 149.3, 146.9, 139.1, 134.8, 119.2, 67.6; ^{19}F-NMR ($CDCl_3$, δ/ppm): -119.8 (1F, dd, J = 10.6, 21.8 Hz, F3), -138.9 (1F, dd, J = 6.8, 10.6 Hz, F6), -153.3 (1F, dd, J = 6.8, 21.8 Hz, F2). LC-MS$^-$: calc. for $C_6H_2F_3IO$ 273.9101, found [M-H]$^-$ 272.9029, (diff 0.34 ppm). The spectra were analysed and compared to the starting material spectra to ascertain the correct position of the iodine on the ring – see Fig. S22.

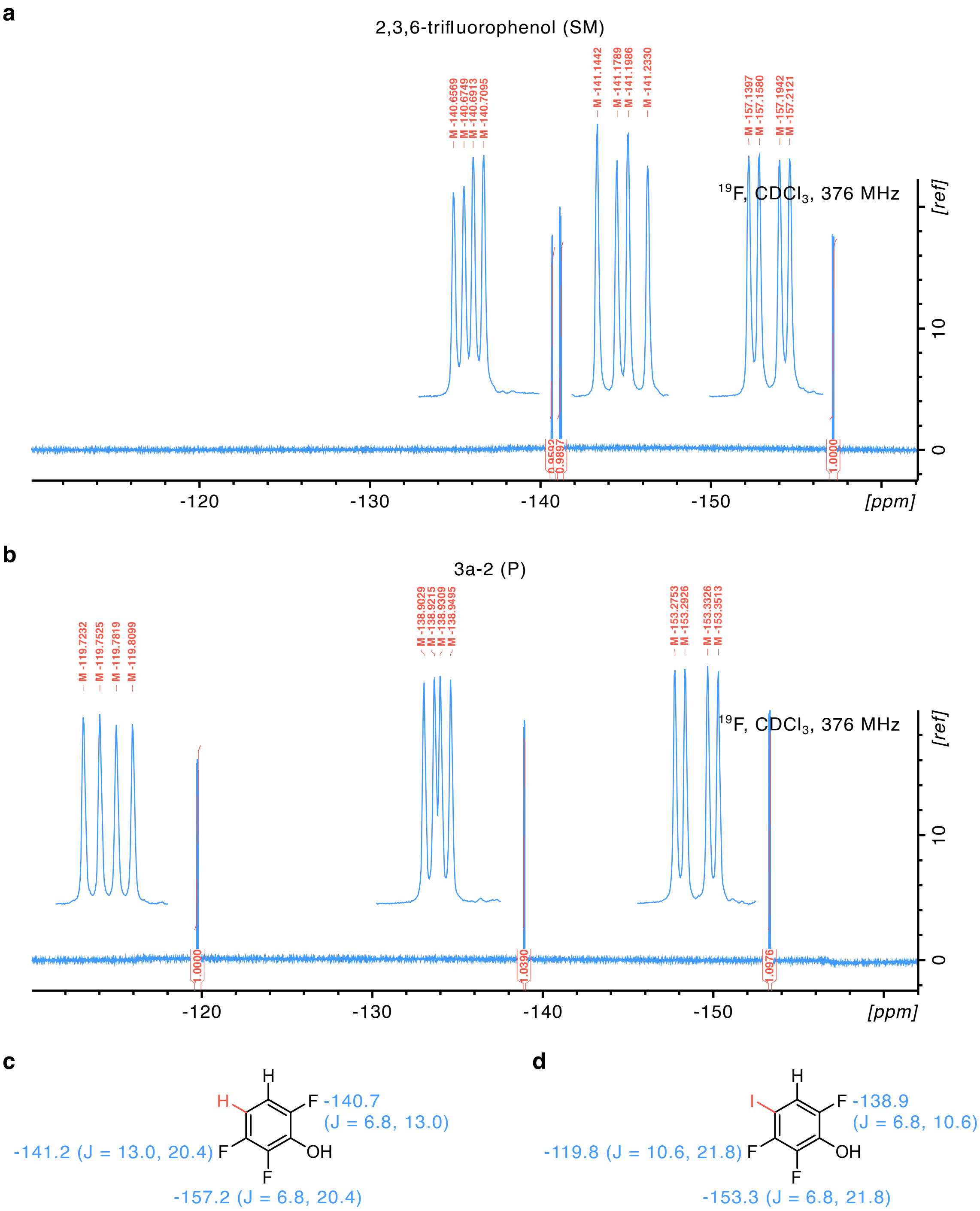

Figure S22 | Confirmation of the desired structure of the product 3a-2. ^{19}F-NMR spectra of 2,3,6-trifluorophenol (panels a,c) and product **3a-2** (panels b,d) identify fluorine 3 as the one experiencing the most significant shift on addition of the fluorine atom (+21.4 ppm), consistent with the predicted +19.9 ppm shift from an orthogonal iodine on a fluoroaryl compound [15]. The J-coupling pattern supports the identification of the new -119.8 ppm peak as F3, located at -141.2 ppm in the SM (panels c,d). These observations place the iodine atom at position 4, confirming the correct structure of compound **3a-2** (2,3,6-trifluoro-4-iodophenol).

4-(3-(2-(Boc-amino)ethoxy)prop-1-yn-1-yl)-2,3,6-trifluorophenol (3c-3): 345.32 g/mol **Sonogashira coupling** with X_1 = 548 mg **3a-2**; X_2 = 598 mg **c-2**; X_3 = 3 h. The reaction mixture was diluted into 100 mL saturated ammonium chloride (1:20) and immediately extracted with EtOAc (3 × 30 mL). The joined organic fractions were washed with water (2 × 100 mL) and brine (100 mL), dried over $MgSO_4$, filtered and concentrated *in vacuo*. The residue was purified by flash chromatography on silica gel (74:25:1 PE:EtOAc:AcOH).

Yields 610 mg of **3c-3** at 90 % purity (549 mg pure product, 1.59 mmol, 80 %) as a golden-brown viscous liquid, used without further purification.

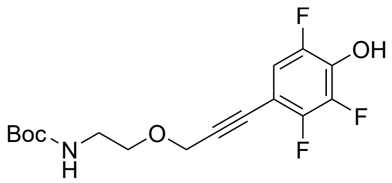

3c-3
$C_{16}H_{18}F_3NO_4$

^{1}H-NMR ($CDCl_3$, δ/ppm): 6.91 (1H, ddd, J = 10.4, 5.8, 1.7 Hz, H5), 4.99 (1H, s-br, NH), 4.38 (2H, s, -O-CH_2-C), 3.65 (2H, t, J = 5.1 Hz, -O-CH_2-CH_2-), 3.37 (2H, q, J = 4.9 Hz, -O-CH_2-CH_2-), 1.44 (9H, s, Boc); ^{13}C-NMR (176 MHz, $CDCl_3$, δ/ppm): 156.3, 148.8 (dd, J = 239, 11.4 Hz), 147.3 (d, J = 239 Hz), 140.9 (m), 135.9–13.7 (m), 113.6 (d, J = 21.7 Hz), 102.3, 90.2, 79.8, 78.0, 69.2, 59.0, 40.4, 28.4; ^{19}F-NMR ($CDCl_3$, δ/ppm): -138.6 (1F, dd, J = 19.7, 10.9 Hz, F3), -140.4 (1F, dd, J = 10.9, 7.9 Hz, F6), -156.9 (1F, dd, J = 19.7, 7.9 Hz, F2). LC-MS$^+$: calc. for $C_{16}H_{18}F_3NO_4$ 345.1191, found $[M+Na]^+$ 368.1085 (diff 1.25 ppm). Also see Figure S23.

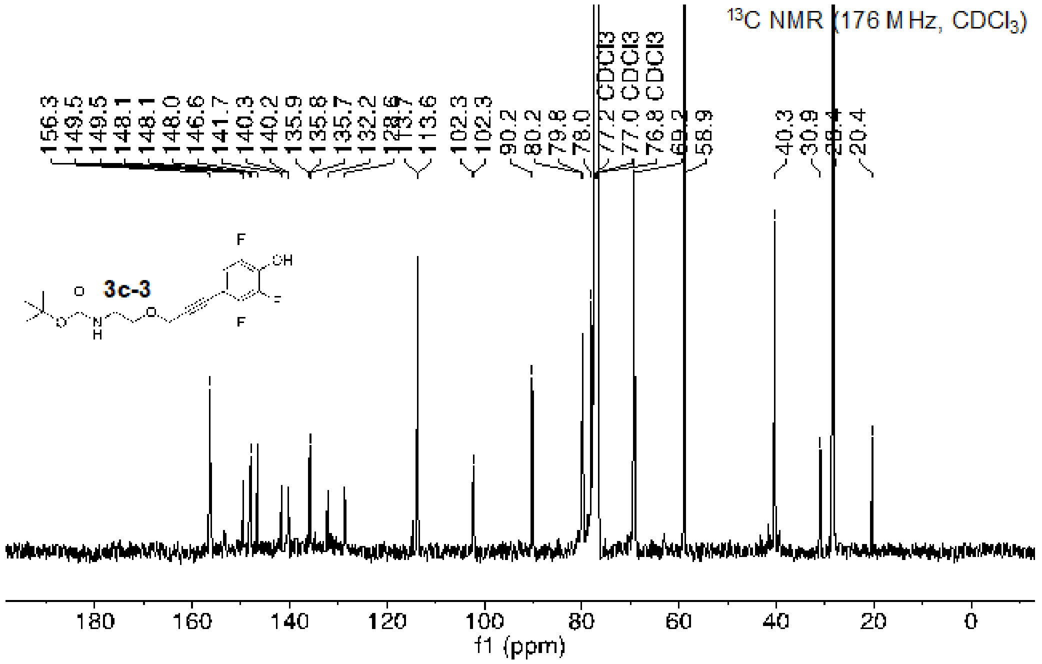

Figure S23 | 3c-3 NMR spectra

4-(3-(2-(Boc-amino)ethoxy)propyl)-2,3,6-trifluorophenol (3c-4): 349.35 g/mol **Alkyne reduction** of 1.2 mmol with X_1 = 462 mg **3c-3** at 90 %wt (416 mg pure compound); X_2 = 47 mg; X_3 = 42 h.

Yields 375 mg of **3c-4** (1.07 mmol, 89 %).

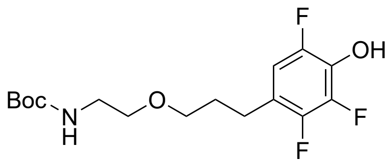

3c-4
$C_{16}H_{22}F_3NO_4$

^{1}H-NMR ($CDCl_3$, δ/ppm): 6.68 (1H, ddd, J = 10.7, 6.1, 1.7 Hz, H5), 4.91 (1H, s-br, NH), 3.47 (2H, t, J = 5.0 Hz, -O-CH_2-CH_2-NH-), 3.43 (2H, t, J = 6.3 Hz, -O-CH_2-CH_2-CH_2-), 3.31 (2H, dt, J = 5.8, 4.3 Hz, -O-CH_2-CH_2-NH-), 2.64 (2H, t, J = 7.5 Hz, Ar-CH_2-), 1.83 (2H, quintet, J = 6.9 Hz, Ar-CH_2-CH_2-), 1.45 (9H, s, Boc); ^{13}C-NMR ($CDCl_3$, δ/ppm): 156.4, 148.9, 147.3, 144.9, 132.6, 120.2, 110.5, 79.8, 69.97, 69.93, 40.6, 29.8, 28.5, 25.0; ^{19}F-NMR ($CDCl_3$, δ/ppm): -141.6 (1F, dd, J = 13.3, 3.3 Hz, F6), -147.5 (1F, dd, J = 19.5, 13.3 Hz, F3), -158.0 (1F, dd, J = 19.5, 3.3 Hz, F2). Low-res MS$^-$: calc. for $C_{16}H_{22}F_3NO_4$ 349.15, found $[M-H]^-$ 348.41.

2-(3-(2,3,5-trifluoro-4-hydroxyphenyl)propoxy)ethan-1-aminium trifluoroacetate (3c-5): 363.26 **Boc deprotection** with X_1 = 349 mg **3c-4**; X_2 = 5 h.

Yields 352 mg of **3c-5** (0.97 mmol, 97 %) as a flaky white solid.

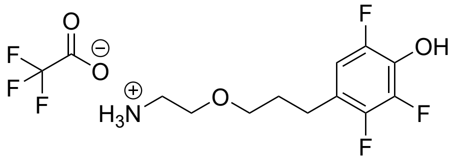

3c-5
$C_{11}H_{15}F_3NO_2^+ \cdot C_2F_3O_2^-$

^{1}H-NMR (MeOD, δ/ppm): 6.79 (1H, ddd, J = 11.1, 6.5, 2.1 Hz, H5), 3.64 (2H, t, J = 4.9 Hz, -O-CH_2-CH_2-NH-), 3.52 (2H, t, J = 6.3 Hz, -O-CH_2-CH_2-CH_2-), 3.12 (2H, dt, J = 4.9, 1.8 Hz, -O-CH_2-CH_2-NH-), 2.69 (2H, t, J = 7.6 Hz, Ar-CH_2-), 1.88 (2H, quintet, J = 7.0 Hz, Ar-CH_2-CH_2-); ^{19}F-NMR (MeOD, δ/ppm): -77.9 (3F, s, TFA), -142.0 (1F, dd, J = 12.7, 7.8 Hz, F6), -150.7 (1F, dd, J = 19.4, 12.7 Hz, F3), -160.4 (1F, dd, J = 19.4, 7.8 Hz, F2). Low-res MS$^+$: calc. for $C_{11}H_{14}F_3NO_2$ 249.10, found $[M+H]^+$ 250.52.

Linker 3c: 407.39 g/mol **HATU amide coupling** of 0.9 mmol with X_1 = 176 mg trans-3-benzoylacrylic acid (1.0 mmol, 1.1 eq); X_2 = RT for 1.5 h; X_3 = 327 mg **3c-5**; X_4 = O/N; X_5 = 100 % EtOAc (R_f = 0.74).

Yields 183 mg of **3c** (0.45 mmol, 45 %).

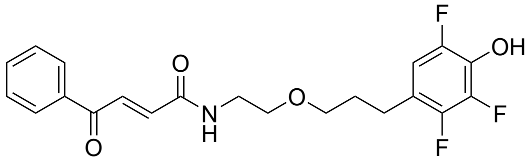

3c
$C_{21}H_{20}F_3NO_4$

^{1}H-NMR (DMSO-d_6, δ/ppm): 8.00 (2H, d, J = 7.8 Hz, Ph-*o*), 7.76 (1H, d, J = 15.3, alkene Ph side), 7.70 (1H, t, J = 7.5 Hz, Ph-*p*), 7.57 (2H, t, J = 7.4 Hz, Ph-*m*), 7.04 (1H, d, J = 15.3, alkene NH side), 6.96 (1H, ddd, J = 11.5, 6.6, 2.0 Hz, H5), 4.04 (1H, s-br, NH), 3.46 (2H, t, J = 5.3 Hz, -O-CH_2-CH_2-NH-), 3.40 (2H, t, J = 5.9 Hz, -O-CH_2-CH_2-CH_2-), 3.38 (2H, t, J = 5.0 Hz, -O-CH_2-CH_2-NH-), 2.60 (2H, t, J = 7.6 Hz, Ar-CH_2-), 1.76 (2H, quintet, J = 6.9 Hz, Ar-CH_2-CH_2-); ^{13}C-NMR (DMSO-d_6, δ/ppm): 189.9, 163.5, 146.8, 144.4, 142.8, 136.7, 136.4, 133.7, 132.9, 132.0, 129.0, 128.6, 119.0, 110.8, 69.1, 68.5, 39.0, 29.4, 24.1; ^{19}F-NMR (DMSO-d_6, δ/ppm): -137.5 (1F, dd, J = 12.7, 7.5 Hz, F6), -147.4 (1F, dd, J = 21.2, 12.7 Hz, F3), -156.2 (1F, dd, J = 21.2, 7.5 Hz, F2). Full spectra and assignments appear in Fig. S24. LC-MS$^+$: calc. for $C_{21}H_{20}F_3NO_4$ 407.1346, found $[M+H]^+$ 408.1418, (diff 0.08 ppm).

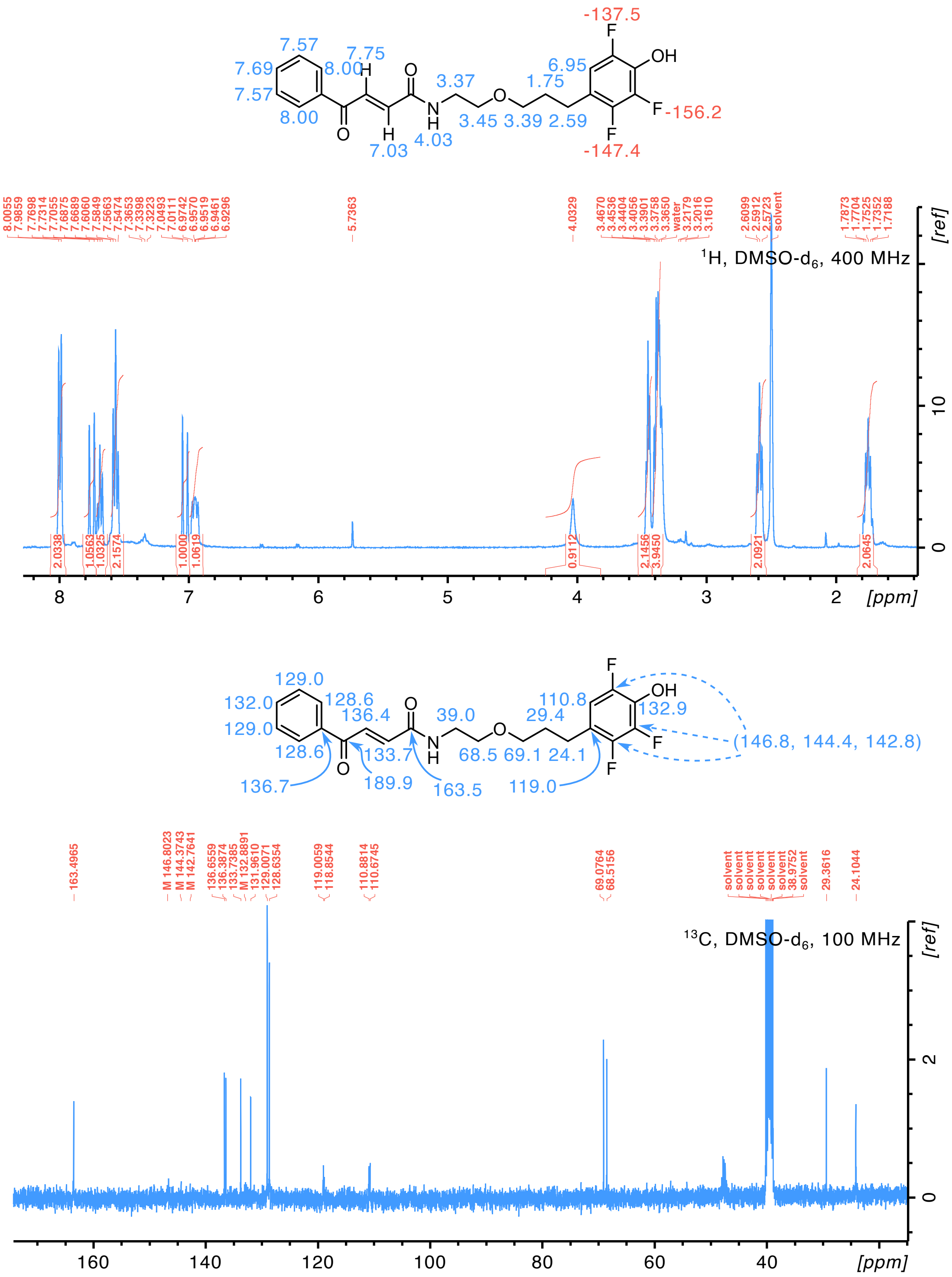

Figure S24 | Linker 3c NMR spectra

3c-rho: 868.39 g/mol Modified **EDC esterification**: 96 mg rhodamine B (200 µmol, 2 eq), 46 mg *N*-(3-Dimethylaminopropyl)-*N* -ethylcarbodiimide hydrochloride (EDC·HCl) (240 µmol, 2.4 eq) and 4.9 mg 4-dimethylaminopyridine (40 µmol, 0.4 eq) were weighed in an oven-dried round-bottom flask and dissolved in dry DCM (1.5 mL) at 0 °C. In a separate vial, 41 mg **3c** (100 µmol) was dissolved in a mixture of dry DMF (100 µL) and dry DCM

(1 mL), then added dropwise over the course of 45 min to the stirred reaction mixture. The reaction was brought to room temperature and stirred overnight. The reaction mixture was then diluted 20-fold in DCM, washed once with H_2O then separated; aqueous phase was extracted with DCM, the two organic phases were joined, washed twice with 100 mL H_2O and once with 100 mL brine, dried over $MgSO_4$, filtered and dried *in vacuo*. The residue was purified by flash chromatography on silica gel.

Yields 71 mg of **3c-rho** (82 µmol, 82 %).

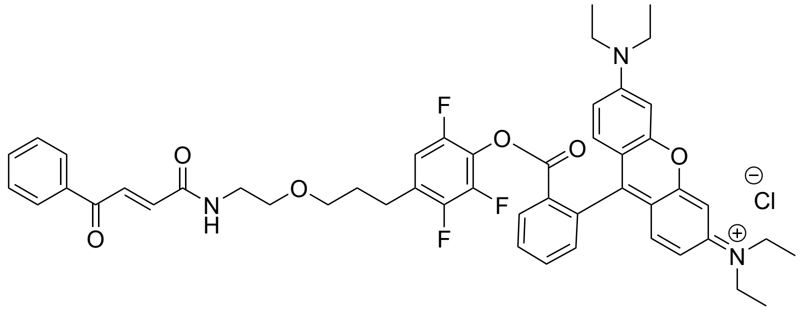

3c-rho
$C_{49}H_{49}F_3N_3O_6^+ \cdot Cl^-$

^{1}H-NMR ($CDCl_3$, δ/ppm): see Fig S25; ^{13}C-NMR ($CDCl_3$, δ/ppm): 245.0, 190.4, 164.7, 157.9, 157.1, 155.7, 137.1, 136.1, 134.6, 133.6, 133.0, 132.2, 131.1, 131.0, 130.9, 129.1, 128.9, 127.9, 114.5, 113.6, 96.6, 77.4, 69.7, 69.1, 46.3, 39.7, 29.8, 29.5, 25.6, 12.8; ^{19}F-NMR ($CDCl_3$, δ/ppm): -132.8 (1F, d, J = 13.4 Hz, F6), -145.8 (1F, dd, J = 21.0, 13.4 Hz, F3), -149.0 (1F, d, J = 21.0 Hz, F2). LC-MS$^+$: calc. for $C_{49}H_{49}F_3N_3O_6$ 832.3612, found $[M+H+Na]^{2+}$ 428.1769 (diff 3.84 ppm).

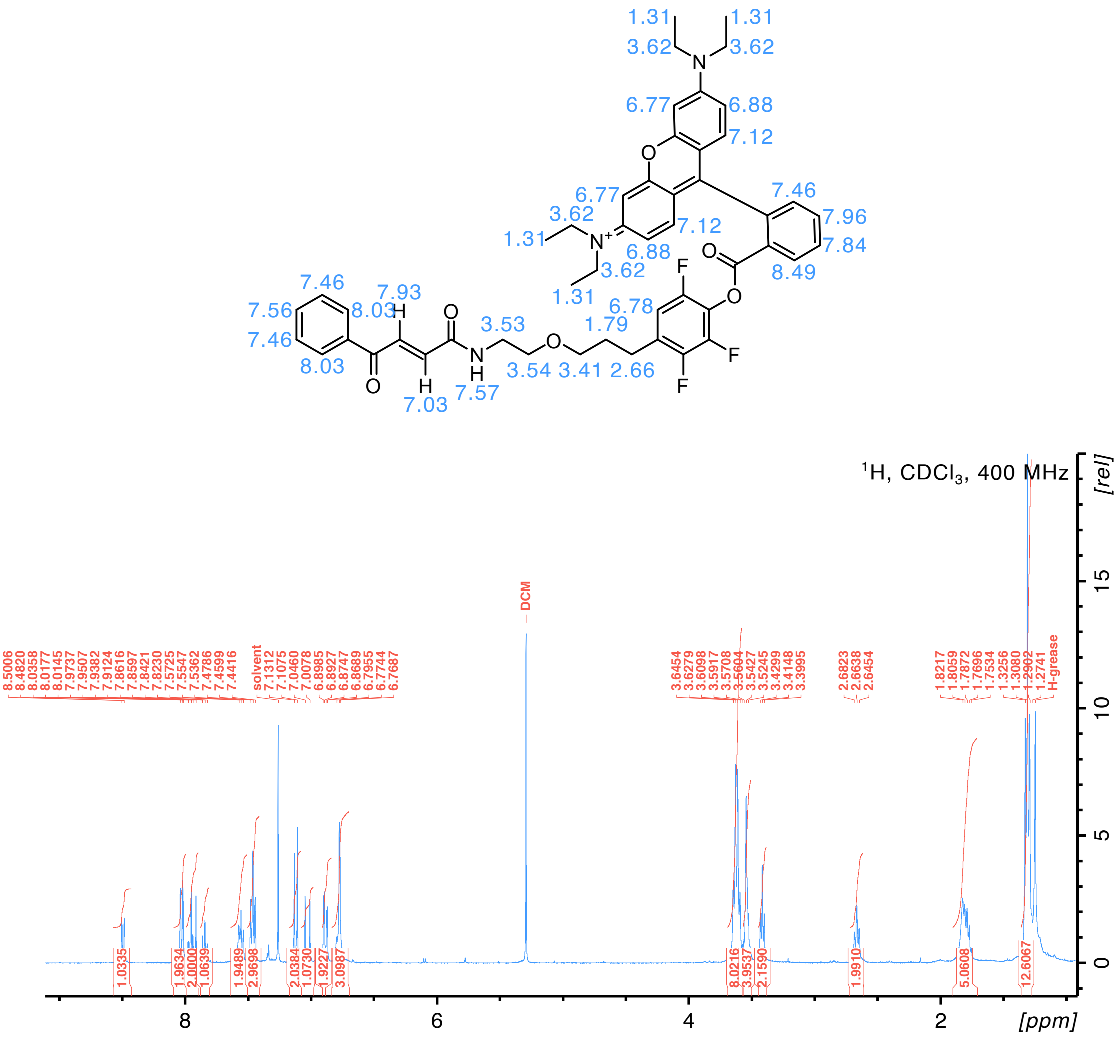

Figure S25 | 3c-rho NMR spectra

3c-bio: 633.68 g/mol Modified **EDC esterification**: 49 mg biotin (200 µmol, 2 eq), 46 mg *N*-(3-Dimethylaminopropyl)-*N* -ethylcarbodiimide hydrochloride (EDC·HCl) (240 µmol, 2.4 eq) and 4.9 mg 4-dimethylaminopyridine (40 µmol, 0.4 eq) were weighed in an oven-dried round-bottom flask and dissolved in dry DCM (1.5 mL) at 0 °C. In a separate vial, 41 mg **3c** (100 µmol) was dissolved in dry DCM (1 mL), then added dropwise over the course of 45 min to the stirred reaction mixture. The reaction was brought to room temperature and stirred overnight. The reaction mixture was then decanted into a 15 mL centrifuge tube using DCM and a precipitate was pelleted by centrifugation (3 min / 4000 × g) and the supernatant was purified by flash chromatography on silica gel (gradient 95:5 → 92:8 DCM:MeOH).

Yields 47 mg of **3c-bio** (74 µmol, 74 %).

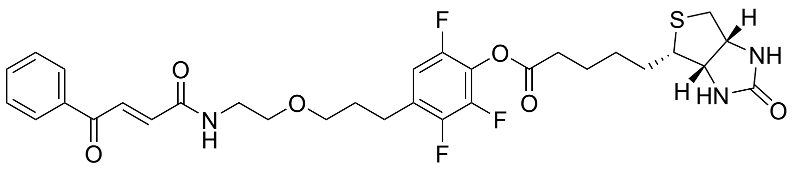

3c-bio

$C_{31}H_{34}F_3N_3O_6S$

^{1}H-NMR ($CDCl_3$, δ/ppm): see Fig S26; ^{13}C-NMR ($CDCl_3$, δ/ppm): 190.0, 170.2, 164.3, 163.4, 137.0, 135.4, 133.9, 133.3, 129.0, 129.0, 111.3, 111.1, 77.4, 70.0, 69.3, 62.0, 60.2, 55.4, 40.7, 39.9, 33.2, 29.6, 28.4, 28.3, 25.6, 24.8; ^{19}F-NMR ($CDCl_3$, δ/ppm): -132.8 (1F, d, J = 13.4 Hz, F6), -146.2 (1F, dd, J = 21.0, 13.4 Hz, F3), -148.8 (1F, d, J = 21.0 Hz, F2). LC-MS$^+$: calc. for $C_{31}H_{34}F_3N_3O_6S$ 633.212, found $[M+H]^+$ 634.224 (diff 7.4 ppm).

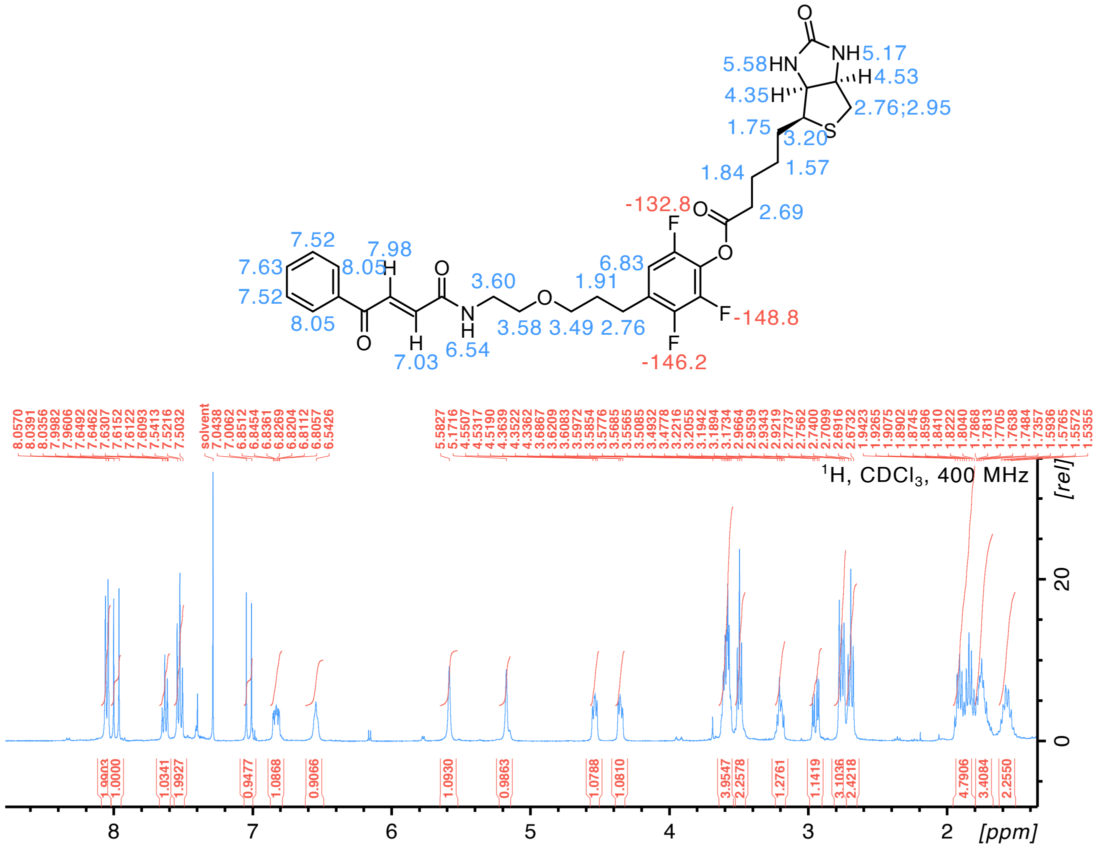

Figure S26 | 3c-bio NMR spectra

3c-pho: 487.37 g/mol **3c** (24 mg, 57 µmol, 1 eq.) was dissolved in acetonitrile (0.5 mL) and the solution was cooled on ice. Then, phosphoryl chloride ($POCl_3$) was added dropwise, and the solution was allowed to reach rt and stirred for 2 hours. Then, sodium bicarbonate ($NaHCO_3$, 14 mg, 3 eq.) was added in water (0.5 mL) and the solution was stirred for 1 hour. After, the solvent was removed under reduced pressure purified by HPLC to yield the product **3c-phosphate** as a white powder (yield 8 mg, 30 %), purity above 95 % (by UPLC-MS and NMR).

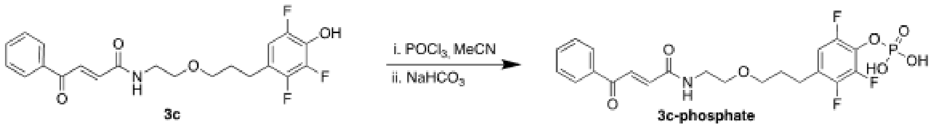

^{1}H-NMR (700 MHz, DMSO-d$_6$, δ/ppm): 8.71 (t, J = 5.7 Hz, 1H), 8.01 (d, J = 7.0 Hz, 2H), 7.76 (d, J = 15.3 Hz, 1H), 7.70 (t, J = 7.4 Hz, 1H), 7.58 (m, 2H), 7.11 (s, b, 1H), 7.03 (d, J = 15.4 Hz, 1H), 3.46 (t, J = 5.7 Hz, 2H), 3.42 (t, J = 6.3 Hz, 2H), 3.36 (q, J = 5.7 Hz, 2H), 2.67 (t, J = 7.7 Hz, 2H), 1.80 (dt, J = 14.0, 6.5 Hz, 2H); ^{13}C-NMR (176 MHz, DMSO-d$_6$, δ/ppm): 189.9, 163.5, 145.8, 144.4, 143.2, 136.6, 136.4, 133.7, 131.9, 129.0, 128.6, 111.1, 69.1, 68.5, 39.0, 29.1, 24.5; aromatic C signals on the fluorophenol ring that does not have H could not be resolved.; ^{19}F-NMR (376 MHz, DMSO-d$_6$, δ/ppm): -134.3 (1F, d, J = 13.5 Hz), -149.8 (1F, dd, J = 20.8, 13.5 Hz), -152.0 (1F, d, J = 20.8 Hz); ^{31}P-NMR (162 MHz, DMSO-d$_6$, δ/ppm): -5.12; LC-MS$^-$: calc. for $C_{21}H_{21}F_3NO_7P$ 487.1007, found [M-H]$^-$ 486.0933 (diff 0.30 ppm). Also see Figure S27 and Figure S28.

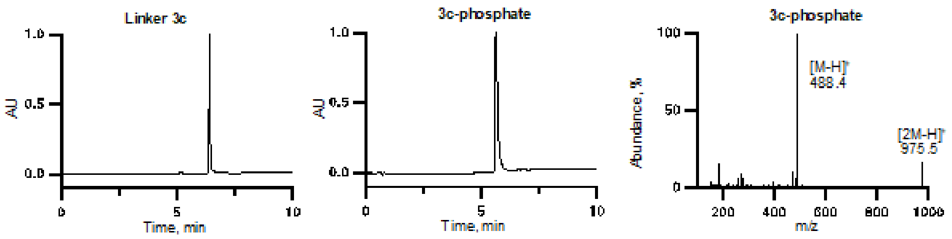

Figure S27 | Characterisation of 3c-phosphate 3c-pho by UPLC-MS

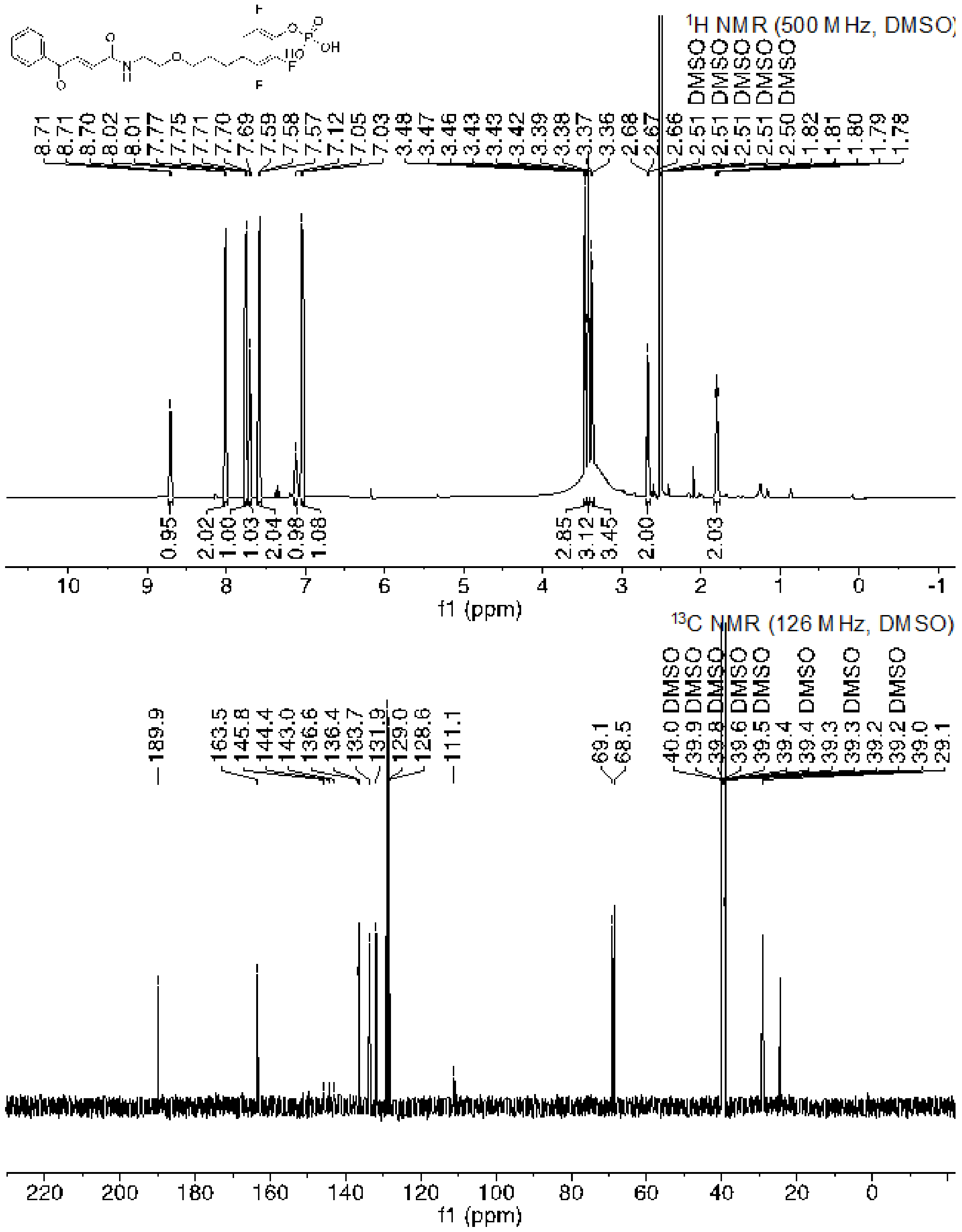

Figure S28 | 3c-pho NMR spectra

3c-sb: 591.55 g/mol **3c** (30.5 mg, 75 µmol, 1 eq.) was dissolved in DMF (0.3 mL). In a separate flask, 4-sulphobenzoic acid (23 mg, 113 µmol, 1.5 eq.) was dissolved in DMF (0.3 mL) and HATU (38 mg, 100 µmol, 1.35 eq) was added, following by DIPEA (in DME, 2M, 112 µL, 3eq.). The resulting solution was added to the solution of **3c** dropwise and the resulting mixture was stirred for 2 hours. Then, the reaction was poured onto 10 mL aqueous HCl (10 %) and extracted with ethyl acetate. Combined organic layers were washed with water, brine, dried over anhydrous $MgSO_4$ and the solvent was removed under reduced pressure. The crude mixture was purified by prep-HPLC to yield the product **3c-sb** as a white powder (yield 18 mg, 44 %).

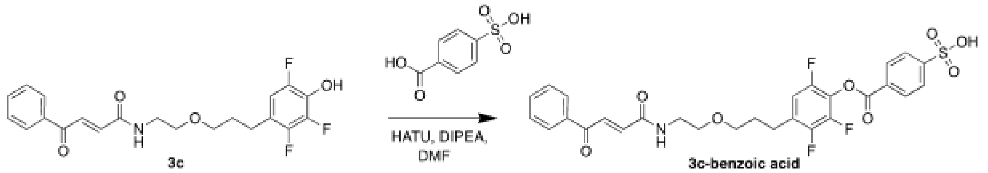

^{1}H-NMR (500 MHz, DMSO-d$_6$, δ/ppm): 8.15 (t, J = 5.7 Hz, 1H), 7.59 (d, J = 8.3 Hz, 2H), 7.45 (d, J = 7.0 Hz, 2H), 7.29 (d, J = 8.4 Hz, 2H), 7.21 (d, J = 15.4 Hz, 1H), 7.14 (t, J = 7.4 Hz, 1H), 7.02 (t, J = 7.8 Hz, 2H), 6.86 – 6.79 (m, 1H), 6.49 (d, J = 15.3 Hz, 1H), 4.01 (s, 2H), 2.97 – 2.87 (m, 4H), 2.87 – 2.80 (m, 2H), 2.21 (t, 2H), 1.31 (p, 2H); ^{13}C-NMR (126 MHz, DMSO-d$_6$, δ/ppm): 189.9, 163.5, 162.4, 154.3, 153.0, 149.0, 145.1, 136.6, 136.4, 133.7, 131.9, 130.2, 129.0, 128.6, 126.4, 126.2, 111.9, 69.0, 68.5, 39.1, 28.9, 24.7; ^{19}F-NMR (376 MHz, DMSO-d$_6$, δ/ppm): -133.5 (d, J = 13.6 Hz), -146.8 (dd, J = 22.5, 13.6 Hz), -150.6 (d, J = 22.5 Hz). LC-MS$^+$: calc. for $C_{28}H_{24}F_3NO_8S$ 591.1174, found $[M+H]^+$ 592.1246 (diff 0.25 ppm). Also see Figure S29.

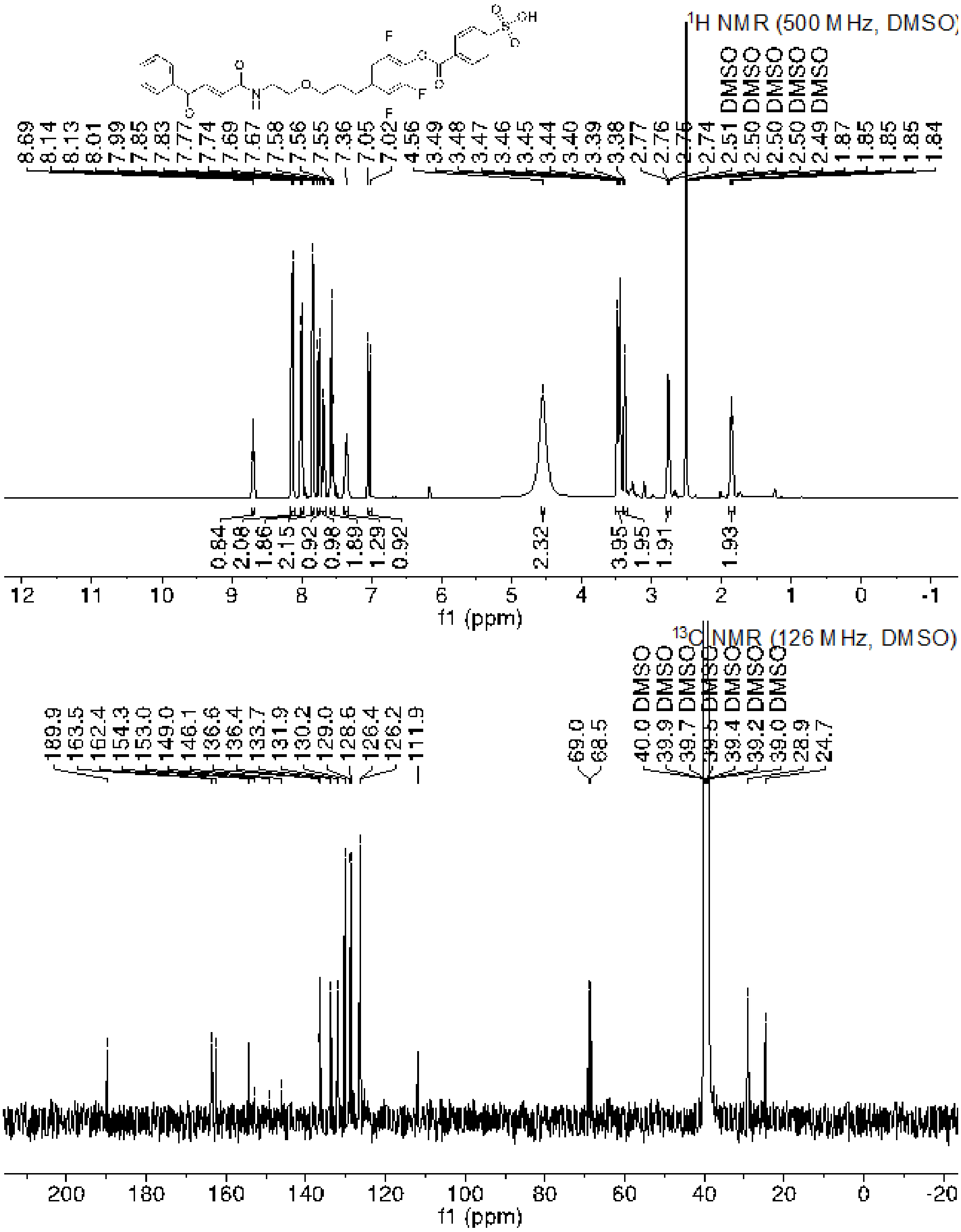

Figure S29 | 3c-sb NMR spectra

3c-pentynoate: 487.48 g/mol **3c** (30.5 mg, 75 µmol, 1 eq.) was dissolved in DMF (0.3 mL). In a separate flask, 4-pentynoic acid (11 mg, 112 µmol, 1.5 eq.) was dissolved in DMF (0.3 mL) and HATU (38 mg, 100 µmol, 1.35 eq) was added, following by DIPEA (in DME, 2M, 112 µL, 3eq.). The resulting solution was added to the solution of **3c** dropwise and the resulting mixture was stirred for 2 hours. Then, the reaction was poured onto 10 mL water and extracted with ethyl acetate. Combined organic layers were washed with water, brine, dried over anhydrous $MgSO_4$ and the solvent was removed under reduced pressure. The crude mixture was purified by prep-HPLC to yield the product **3c-pentynoate** as a white powder (yield 18 mg, 29 %).

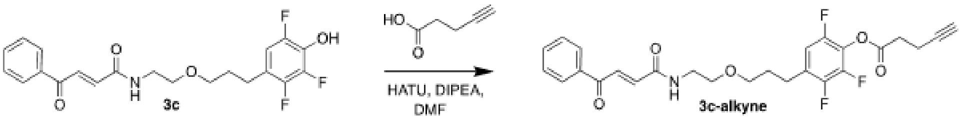

^{1}H-NMR (700 MHz, $CDCl_3$, δ/ppm): 8.03 (d, J = 8.4 Hz, 2H), 7.97 (d, J = 15.0 Hz, 1H), 7.62 (t, J = 7.4 Hz, 1H), 7.51 (t, J = 7.8 Hz, 2H), 6.97 (d, J = 15.0 Hz, 1H), 6.81 (m, 1H), 6.26 (s, 1H), 3.62 – 3.57 (m, 2H), 3.55 (t, J = 5.1 Hz, 2H), 3.48 (t, J = 6.1 Hz, 2H), 2.91 (t, J = 8.0 Hz, 2H), 2.74 (t, J = 7.5 Hz, 2H), 2.67–2.62 (m, 2H), 2.04 (t, J = 2.7 Hz, 1H), 1.90 (p, J = 6.6 Hz, 2H); ^{13}C-NMR (176 MHz, $CDCl_3$, δ/ppm): 189.7, 168.4, 164.1, 151.1, 149.7, 136.9, 135.0, 133.8, 133.5, 128.9 (d, J = 3.2 Hz), 128.9, 128.8, 125.8, 111.0, 81.4, 69.9, 69.7, 69.1, 39.8, 32.7, 29.5, 25.5, 14.4; ^{19}F-NMR (376 MHz, $CDCl_3$, δ/ppm): -132.25 (d, J = 13.4 Hz), -145.90 (dd, J = 21.1, 13.4 Hz), -148.22 (d, J = 21.1 Hz). LC-MS$^+$: calc. for $C_{26}H_{24}F_3NO_5$ 487.1614, found $[M+H]^+$ 488.1687 (diff 1.53 ppm). Also see Figure S30.

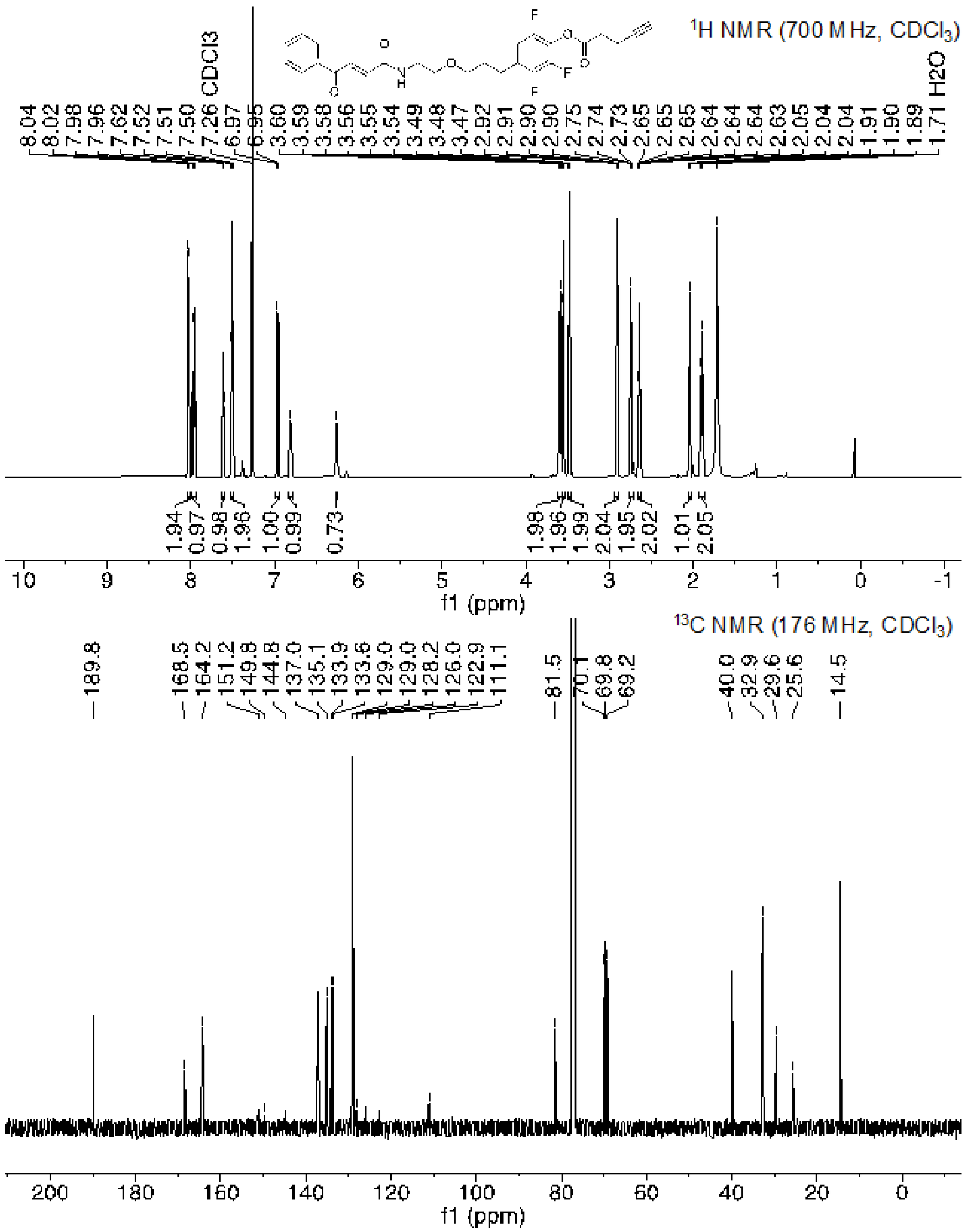

Figure S30 | 3c-pentynoate NMR spectra

3c-propargylglycine: 724.73 g/mol **3c** (30.5 mg, 75 µmol, 1 eq.) was dissolved in DMF (0.3 mL). In a separate flask, N-Fmoc-L-propargylglycine (38 mg, 113 µM, 1.5 eq.) was dissolved in DMF (0.3 mL) and HATU (38 mg, 100 µM, 1.35 eq) was added, following by DIPEA (in DME, 2M, 112 µL, 3eq.). The resulting solution was added to the solution of **3c** dropwise and the resulting mixture was stirred for 2 hours. Then, the reaction was poured onto 10 mL water and extracted with ethyl acetate. Combined organic layers were washed with water, brine, dried over anhydrous $MgSO_4$ and the solvent was removed under reduced pressure. The crude mixture was purified by prep-HPLC to yield the product **3c-propargylglycine** as a white powder (yield 15.5 mg, 29 %).

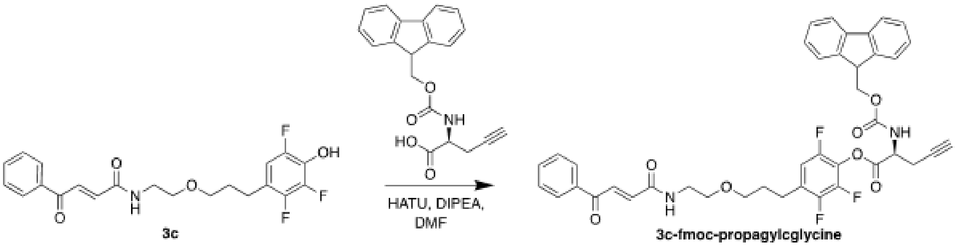

^{1}H-NMR (700 MHz, $CDCl_3$, δ/ppm): 8.03 (d, J = 7.8 Hz, 2H), 7.97 (d, J = 15.0 Hz, 1H), 7.77 (d, J = 7.5 Hz, 2H), 7.61 (t, J = 6.9 Hz, 2H), 7.50 (t, J = 7.6 Hz, 2H), 7.40 (t, J = 7.5 Hz, 2H), 7.31 (t, J = 7.6 Hz, 2H), 6.96 (d, J = 15.0 Hz, 1H), 6.84 (t, J = 7.8 Hz, 1H), 6.23 (s, 1H), 5.74 (d, J = 8.7 Hz, 1H), 4.94 (m, 1H), 4.44 (dd, J = 7.2, 2.9 Hz, 2H), 4.26 (t, J = 7.2 Hz, 1H), 3.60 (q, J = 5.4 Hz, 2H), 3.55 (t, J = 5.1 Hz, 2H), 3.48 (t, J = 6.1 Hz, 2H), 3.01 (d, J = 16.5 Hz, 1H), 2.92 (d, J = 17.0 Hz, 1H), 2.76 (t, J = 7.5 Hz, 2H), 2.16 (s, 1H), 1.91 (p, J = 6.6 Hz, 2H); ^{13}C-NMR (176 MHz, $CDCl_3$, δ/ppm): 189.7, 164.1, 143.7, 141.3, 136.6, 135.0, 133.8, 133.5, 128.9, 128.9, 127.8, 127.1, 125.1, 120.0, 72.6, 70.0, 69.1, 67.5, 52.4, 47.1, 39.8, 29.5, 25.6, 22.9; ^{19}F-NMR (376 MHz, $CDCl_3$, δ/ppm): -131.71 (d, J = 13.4 Hz), -131.88, -145.51 (dd, J = 20.9, 13.4 Hz), -147.56 (d, J = 20.9 Hz). LC-MS$^+$: calc. for $C_{41}H_{35}F_3N_2O_7$ 724.2410, found $[M+Na]^+$ 747.2328 (diff 5.00 ppm). Also see Figure S31.

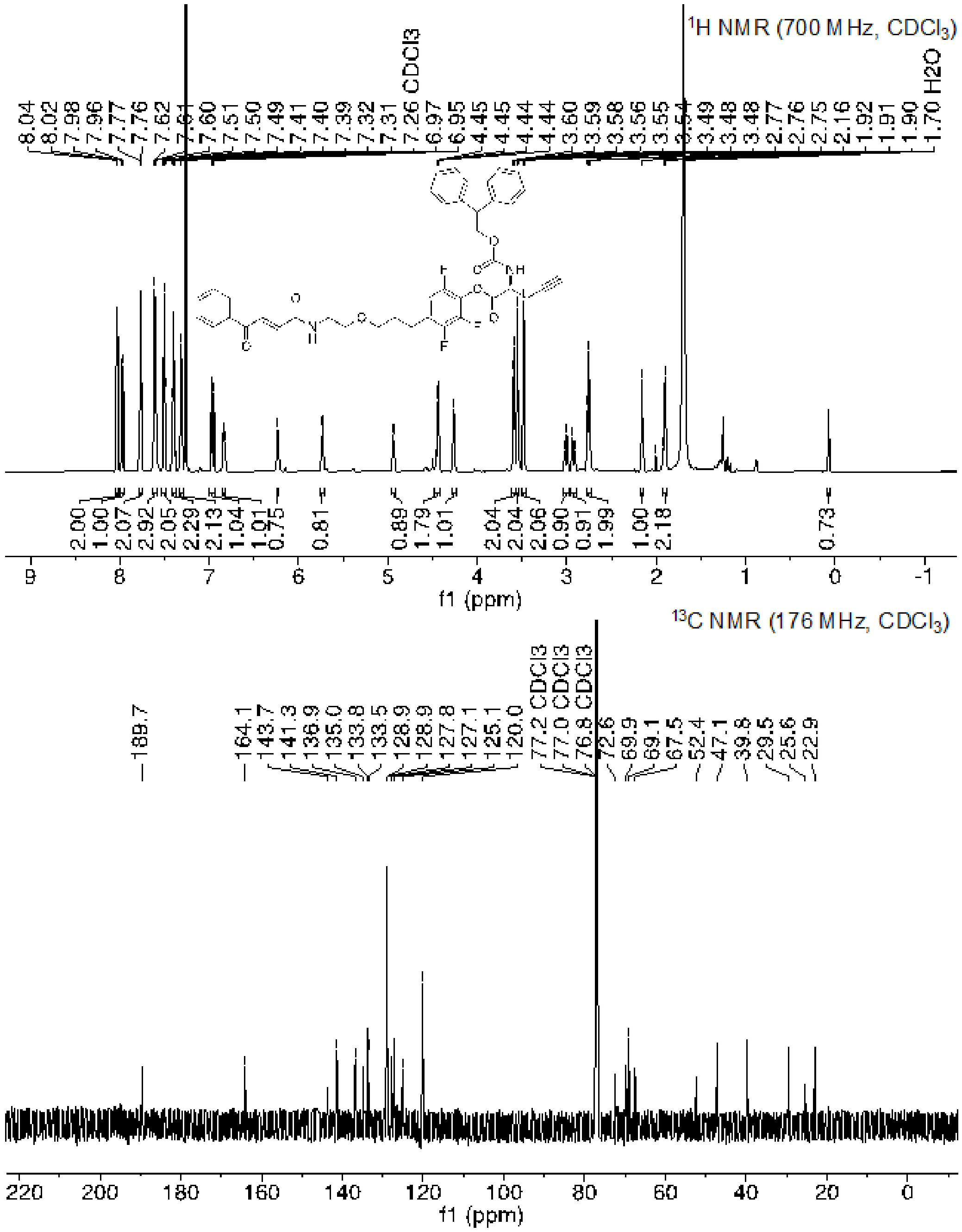

Figure S31 | 3c-propargylglycine NMR spectra

Linker 3d

Reaction scheme: Fig. S20

***N*-Boc-2-(2-(prop-2-yn-1-yloxy)ethoxy)ethan-1-amine (d-2): 243.30 g/mol** Williamson ether synthesis, based on refs [13, 14]. 4.11 g 2-[2-(Boc-amino)ethoxy]ethanol (20 mmol) was weighed in a round-bottom flask with 2.98 g of 80 %wt propargyl bromine in toluene stabilised with MgO (2.38 g net weight, 20 mmol, 1 eq), and then dissolved in tetrahydrofuran (20 mL). 476 mg tetra-(*n*-butyl)ammonium iodide (TBAI) (1.2 mmol, 0.06 eq), 498 mg potassium iodide (3 mmol, 0.15 eq) and 1.12 g potassium hydroxide

(20 mmol, 1 eq) were added and the reaction mixture was stirred at room temperature overnight. On completion, the mixture was filtered and the filtrate was concentrated *in vacuo*, diluted with H_2O (20 mL) and extracted twice with EtOAc (50 mL). The organic phase was concentrated *in vacuo*, loaded on silica gel and purified by flash chromatography (5:1 PE:EtOAc).

Yields 3.1 g of **d-2** (12.7 mmol, 64 %) as a colourless oil.

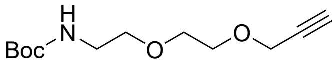

d-2
$C_{12}H_{21}NO_4$

^{1}H-NMR ($CDCl_3$, δ/ppm): 4.94 (1H, s-br, NH), 4.20 (2H, s, -CH_2C), 3.69 (2H, t, J = 4.5 Hz, -O-CH_2-CH_2-O-), 3.64 (2H, t, J = 4.5 Hz, -O-CH_2-CH_2-NH-), 3.54 (2H, t, J = 5.1 Hz, -O-CH_2-CH_2-NH-), 3.31 (2H, t, J = 5.0 Hz, -O-CH_2-CH_2-O-), 2.44 (1H, s, CH), 1.44 (9H, s, Boc); ^{13}C-NMR ($CDCl_3$, δ/ppm): 156.1, 79.7, 79.4, 74.8, 70.5, 70.3, 69.2, 58.6, 40.6, 28.5.

4-(3-(2-(2-(Boc-amino)ethoxy)ethoxy)prop-1-yn-1-yl)-2,3,6-trifluorophenol (3d-3): 389.37 **Sonogashira coupling** with X_1 = 548 mg **3a-2**; X_2 = 730 mg **d-2**; X_3 = 3 h. The reaction mixture was diluted into 100 mL saturated ammonium chloride (1:20) and immediately extracted with EtOAc (3 × 30 mL). The joined organic fractions were washed with water (2 × 100 mL) and brine (100 mL), dried over $MgSO_4$, filtered and concentrated *in vacuo*. The residue was purified by flash chromatography on silica gel (39:1 → 29:1 DCM:MeOH).

Yields 702 mg at 75 %wt purity of **3d-3** (529 mg pure product, 1.36 mmol, 68 %), used without further purification.

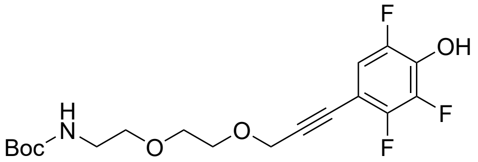

3d-3
$C_{18}H_{22}F_3NO_5$

^{1}H-NMR ($CDCl_3$, δ/ppm): 6.89 (1H, ddd, J = 10.3, 5.9, 2.0 Hz, H5), 5.05 (1H, s-br, NH), 4.42 (2H, s, -O-CH_2-C), 3.75 (2H, t, J = 4.3 Hz, -O-CH_2-CH_2-O-), 3.67 (2H, t, J = 4.3 Hz, -O-CH_2-CH_2-O-), 3.56 (2H, t, J = 5.0 Hz, -O-CH_2-CH_2-NH-), 3.33 (2H, t, J = 5.2 Hz, -O-CH_2-CH_2-NH-), 1.43 (9H, s, Boc); ^{13}C-NMR ($CDCl_3$, δ/ppm): 156.4, 113.87–113.47, 90.3, 79.7, 78.2, 77.4, 70.5, 70.3, 69.3, 59.2, 40.5, 28.5; ^{19}F-NMR ($CDCl_3$, δ/ppm): -138.8 (1F, dd, J = 20.1, 11.7 Hz, F3), -140.3 (1F, dd, J = 11.7, 8.0 Hz, F6), -156.9 (1F, dd, J = 20.1, 8.0 Hz, F2).

4-(2-(3-(2-(Boc-amino)ethoxy)propoxy)ethyl)-2,3,6-trifluorophenol (3d-4): 393.40 g/mol **Alkyne reduction** of 1.2 mmol with X_1 = 622 mg **3d-3** at 75 %wt (467 mg pure compound); X_2 = 50 mg; X_3 = 42 h. Full conversion by NMR.

Yields 470 mg of **3d-4** (1.19 mmol, 99 %).

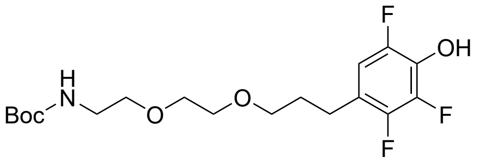

3d-4
$C_{18}H_{26}F_3NO_5$

(3-(3-(2,3,5-trifluoro-4-hydroxyphenyl)propoxy)propoxy)methanaminium trifluoroacetate (3d-5): 407.31 g/mol **Boc deprotection** with X_1 = 393 mg **3d-4**; X_2 = 5 h. Following toluene co-evaporation, the residue was re-dissolved in DCM and re-dried, several times.

Yields 403 mg of **3d-5** at 92 %wt (370 mg pure product, 0.91 mmol, 91 %) as a colourless oil, used without further purification.

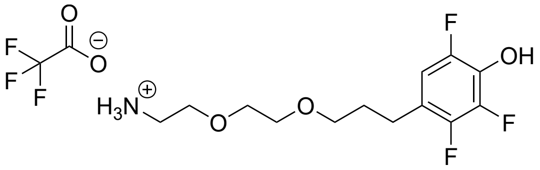

3d-5
$C_{13}H_{19}F_3NO_3^+ \bullet C_2F_3O_2^-$

^{1}H-NMR (MeOD, δ/ppm): 6.79 (1H, ddd, J = 11.3, 6.4, 2.1 Hz, H5), 3.70 (2H, t, J = 4.9 Hz, -O-CH$_2$-CH_2-O-), 3.67 (2H, t, J = 4.4 Hz, -O-CH_2-CH$_2$-NH-), 3.62 (2H, t, J = 4.6 Hz, -O-CH_2-CH$_2$-O-), 3.50 (2H, t, J = 6.3 Hz, -O-CH$_2$-CH_2-NH-), 3.13 (2H, t, J = 4.7 Hz, -O-CH_2-CH$_2$-CH$_2$-), 2.66 (2H, t, J = 7.5 Hz, Ar-CH_2-), 1.85 (2H, qui, J = 7.0 Hz, Ar-CH$_2$-CH_2-); ^{19}F-NMR (MeOD, δ/ppm): -77.8 (3F, s, TFA), -142.0 (1F, dd, J = 12.7, 7.8 Hz, F6), -150.8 (1F, dd, J = 19.6, 12.7 Hz, F3), -160.4 (1F, dd, J = 19.6, 7.8 Hz, F2).

Linker 3d: 451.44 g/mol **HATU amide coupling** of 0.9 mmol with X_1 = 176 mg trans-3-benzoylacrylic acid (1.0 mmol, 1.1 eq); X_2 = RT for >1 hour; X_3 = 400 mg **3d-5** at 91 %wt (0.9 mmol); X_4 = O/N; X_5 = 100 % EtOAc.

Yields 161 mg of **3d** (0.36 mmol, 36 %).

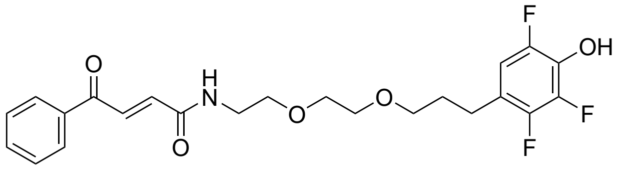

3d
$C_{23}H_{24}F_3NO_5$

^{1}H-NMR (MeOD, δ/ppm): 8.00 (2H, d, J = 7.7 Hz, Ph-*o*), 7.86 (1H, d, J = 15.3, alkene Ph side), 7.65 (1H, t, J = 7.4 Hz, Ph-*p*), 7.53 (2H, t, J = 7.4 Hz, Ph-*m*), 7.03 (1H, d, J = 15.3, alkene NH side), 6.76 (1H, ddd, J = 11.2, 6.4, 2.0 Hz, H5), 3.637 (2H, m, -O-CH$_2$-CH_2-O-), 3.625 (2H, m, -O-CH_2-CH$_2$-NH-), 3.60 (2H, m, -O-CH_2-CH$_2$-O-), 3.52 (2H, t, J = 5.1 Hz, -O-CH$_2$-CH_2-NH-), 3.47 (2H, t, J = 6.3 Hz, -O-CH_2-CH$_2$-CH$_2$-), 2.63 (2H, t, J = 7.6 Hz, Ar-CH_2-), 1.81 (2H, quintet, J = 6.9 Hz, Ar-CH$_2$-CH_2-); ^{13}C-NMR (MeOD, δ/ppm): 191.5, 166.7, 150.7, 148.4, 146.2, 138.2, 136.5, 134.9, 134.6, 134.0, 130.0,

129.8, 120.4, 111.6, 71.3, 71.2, 71.1, 70.3, 40.8, 31.0, 25.7; ^{19}F-NMR (MeOD, δ/ppm): -141.0 (1F, dd, J = 12.7, 7.8 Hz, F6), -149.7 (1F, dd, J = 19.4, 12.7 Hz, F3), -159.4 (1F, dd, J = 19.4, 7.8 Hz, F2). LC-MS$^+$: calc. for $C_{23}H_{24}F_3NO_5$ 451.161, found $[M+H]^+$ 452.162 (diff 13.2 ppm). Full spectra and assignments appear in Fig. S32.

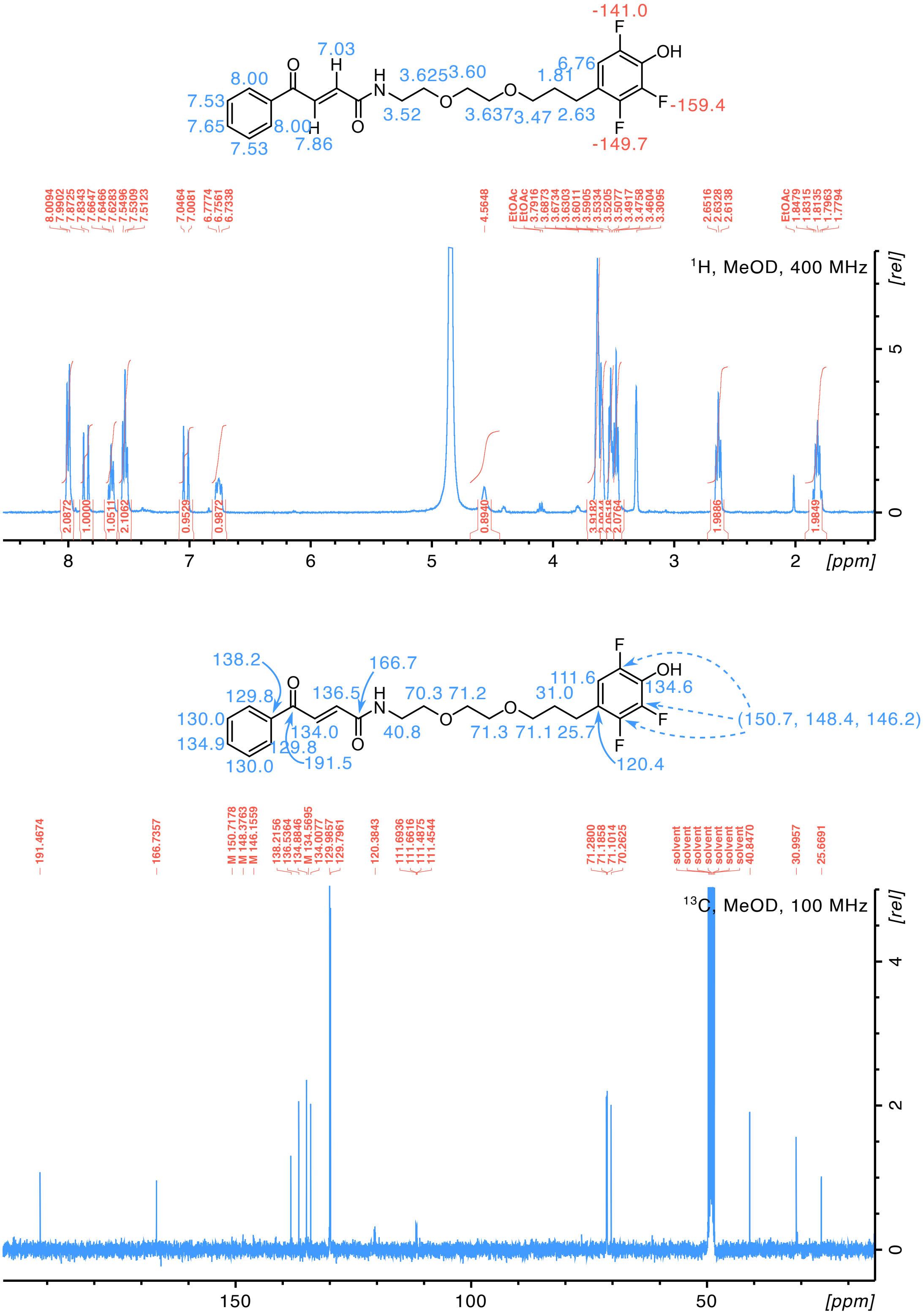

Figure S32 | Linker 3d NMR spectra

3d-rho: 912.44 g/mol Modified **EDC esterification**: See **3c-rho**.

Yields 81 mg of **3d-rho** (89 µmol, 89 %).

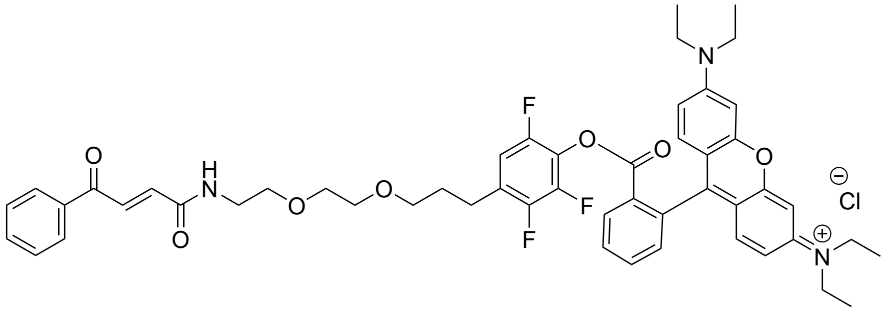

3d-rho
$C_{51}H_{53}F_3N_3O_7^+ \cdot Cl^-$

^{1}H-NMR ($CDCl_3$, δ/ppm): see Fig S33; ^{13}C-NMR ($CDCl_3$, δ/ppm): 190.3, 162.0, 157.9, 157.1, 155.7, 137.1, 136.0, 134.6, 133.6, 133.0, 132.2, 131.1, 131.0, 130.9, 129.1, 128.9, 127.8, 114.5, 113.5, 96.6, 70.4, 70.2, 69.8, 69.4, 46.3, 39.6, 29.8, 29.4, 25.4, 12.8; ^{19}F-NMR ($CDCl_3$, δ/ppm): -132.8 (1F, d, J = 13.4 Hz, F6), -145.8 (1F, dd, J = 21.0, 13.4 Hz, F3), -148.9 (1F, d, J = 21.0 Hz, F2). LC-MS$^+$: calc. for $C_{51}H_{53}F_3N_3O_7$ 876.383, found $[M]^+$ 876.376 (diff 8.0 ppm).

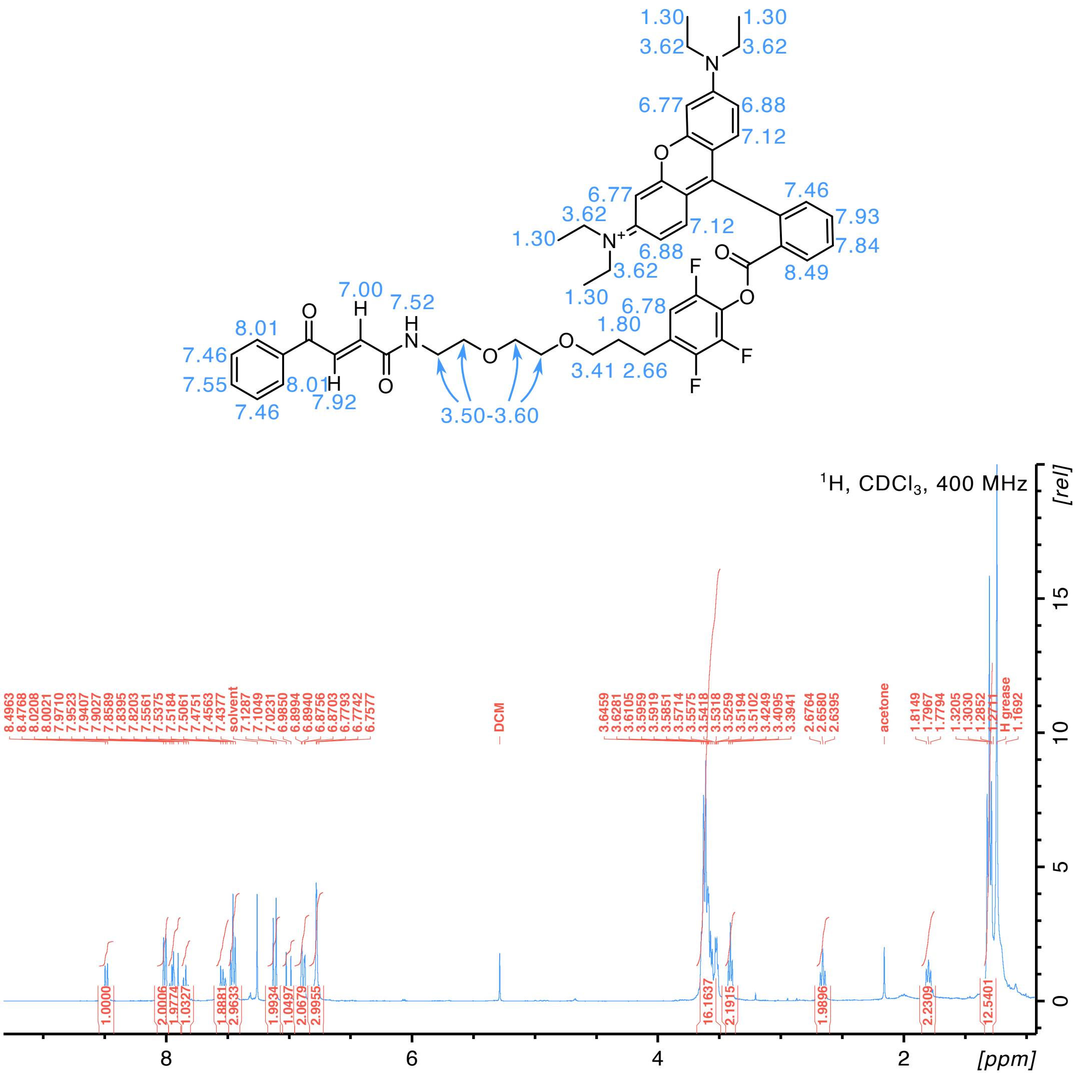

Figure S33 | 3d-rho NMR spectra

Analytical techniques

SDS-PAGE and in-gel fluorescence

Precast Novex NuPAGE Bis-Tris 4–12% or 12–20% gels (Thermo) were used for all SDS-PAGE analyses, running at 200 V for 30–40 min with NuPAGE MES SDS buffer (Thermo). SeeBlue Plus2 (Thermo) pre-stained protein marker was used as standard. Gels were imaged using a NuGenius gel imager (Syngene) either before staining using the UV fluorescence mode, or after staining with InstantBlue (Abcam) using a white light converter.

Western blot for detection of biotinylated proteins

An unstained SDS-PAGE gel was transferred to a nitrocellulose membrane by dry-blotting using an iBlot™ 2 device (ThermoFisher), applying 20 V for 1 min, 23 V for 4 min and finally 25 V for 2 min (GFP experiments) or 20 V for 1 min, 23 V for 3.5 min and 25 V for 1 min (β2m experiments). The membrane was blocked using 5 % BSA in TBS (50 mM Tris + 150 mM NaCl, pH 7.4), in a 50 mL tube, on a tube roller at 6 °C overnight (or 2 h at room temperature in Figure 5h and S11). The membrane was washed twice with TBST (TBS + 0.1 % Tween-20 (w/v)) and twice with TBS, before being incubated with 5 % BSA + 0.5 µg/mL Streptavidin–Alexa Fluor™ 647 conjugate (Invitrogen) (1:4,000 dilution) in TBS for 60 min. Then, the membrane was washed twice with TBST and twice with TBS, and imaged using a ChemiDoc™ MP Imager (Bio-Rad). Band and lane fluorescence intensity were quantified using Image Lab (Bio-Rad), and the total intensities of untreated control lanes were subtracted from each respective treated sample intensity to calculate the signal of non-specific biotinylation by PEABS.

Traditional Western blot for beta 2-microglobulin

An unstained SDS-PAGE gel was transferred to a nitrocellulose membrane by dry-blotting using an iBlot™ 2 device (ThermoFisher), applying 20 V for 1 min, 23 V for 4 min and finally 25 V for 2 min. The membrane was blocked using 5 % BSA in TBS in a 50 mL tube, on a tube roller for 2 h at room temperature. The membrane was washed twice with TBST and twice with TBS, before being incubated with 5 % BSA + 1:10,000 rabbit monoclonal anti-β2m antibody (Abcam ab75853) in TBS for 90 min. Then, the membrane was washed twice with TBST and twice with TBS, before being incubated with 5 % BSA + 1:20,000 donkey anti-rabbit IgG bound to IRDye 680RD (LICORbio 926-68073) in TBS for 60 min. Finally, the membrane was washed twice with TBST and twice with TBS, and imaged using a ChemiDoc™ MP Imager (Bio-Rad).

UV/vis spectrophotometry

Samples were transferred to a UV/vis-transparent cuvette and spectra were recorded between 220–340 nm on a Cary 400 spectrophotometer. Samples of low volume (e.g.

plasmid DNA; or proteins prepared for PEABS generation by reduction and precipitation of aggregates) were measured by depositing a 2 µL sample on a NanoDrop 2000 spectrophotometer (Thermo). In both techniques, the buffer was measured and subtracted as a blank.

For determining protein concentration, absorbance at 280 nm was used according to Beer-Lambert law ($c = \frac{A}{\varepsilon l}$, where $l = 1$ cm for the cuvette used and ε is the predicted attenuation coefficient of the protein).

Far-UV CD spectroscopy

Spectral CD Far-UV CD spectra were recorded between 198–250 nm using a Chirascan system (AppliedPhotopysics). Proteins at 5–10 µM were measured in a quartz cell with a 0.1 cm path length at 25 °C. All spectra were measured as θ (in mdeg) then corrected by subtracting the buffer spectrum. Samples were then transferred to a UV/vis cuvette and their exact concentration was measured as described. Ten spectra per sample were averaged and normalised to Mean Residue Ellipticity (MRE; in $\mathrm{deg} \cdot \mathrm{cm}^2 \cdot \mathrm{dmol}^{-1}$) using the sample concentration (in M) and the number of residues in the protein:
$\mathrm{MRE} = \frac{\theta}{\frac{c}{10} \times 0.1\ \mathrm{cm} \times \#_{\mathrm{residues}}}$

Thermal denaturation CD Samples were prepared as above and covered in a thin layer of mineral oil to prevent evaporation. Circular dichroism was measured at 207 nm (1 nm bandwidth) and five measurements taken over ten seconds were averaged. The signal was recorded every 0.2 °C from 25 °C to 95 °C (cell temperature) at a rate of 1 °C min^{-1} and corrected using the sample temperature, measured by a probe. A two-state unfolding model was used to fit the data for determination of melting temperature.

Nano differential scanning fluorimetry (nanoDSF) nanoDSF was measured using a Tycho™ instrument (NanoTemper) following manufacturer instructions.

Intact protein LC-MS

Samples were prepared by transferring 20 µL into 1.5 mL microcentrifuge tubes and centrifuging for 7 min / 4 °C / 20000 × g. 12 µL of the supernatant liquids were then transferred to 300 µL screw thread vials (Supelco) with pre-slit screw thread caps (Fisher Scientific), and injected to an Acquity UPLC system using an Acquity UPLC Protein BEH C4 column (300 Å pore diameter, 1.7 µm, 2.1 mm × 50 mm) held at 40 °C. Solvent A consisted of 0.1 % formic acid in water, and solvent B of 5 % water + 0.1 % formic acid in acetonitrile. At a flow rate of 0.2 mL/min, a gradient of 7.29 min was utilised for most samples (hold at 5 % B for 0.93 min, increase to 100 % B over 4.28 min, hold for another 1.04 min then decrease back to 5 % B over 1.04 min). For better separation of some samples, a longer gradient of 19.9 min was used (hold at 5 % B for 1.0 min, jump to 24 % B in 0.1 min and hold for 1.9 min, then slowly up to 55 % B over 14 min, jump to 100 % B in 0.1 min and hold for another 1.7 min then decrease back to 5 % B over 0.1 min and hold for

1.1 min). Data were recorded on a Waters Xevo G2-S qTOF mass spectrometer using an electrospray ionisation (ESI) source at 120 °C with a capillary voltage of 3.0 kV and a cone voltage of 40 V. Nitrogen was used as the desolvation gas at a flow rate of 600 L/h. LockSpray was infused throughout the measurement for internal calibration.

Basic analysis The recorded 3-dimensional spectra (retention time × m/z × intensity) were searched for protein peaks by a combination of manual inspection and a custom-written python library. Each peak was then 'summed' (marginalised over retention times) to receive a 2d spectrum of the entire peak (m/z × intensity). These spectra were deconvoluted using UniDec [16] with the following parameters:

- For nanobodies (~15 kDa): m/z range 600–2000; charge range 6–55; mass range 12000–32000 or 12000–16000; sampling resolution 1 Da.
- For GFP (~27 kDa): m/z range 600–1800; charge range 12–55; mass range 24000–32000; sampling resolution 1 Da.

Resulting spectra (intact mass × intensity), internally normalised to the range 0–1, were used to identify the abundant species in solution.

Semi-quantitative analysis After identification of the existing species in each sample, the peaks representing ions corresponding to each species were identified using a custom-written python library, using a combination of predictions and observed signals. In brief, the first prediction for a given mass M consisted of 20 ion peaks, with its "anchor ion" closest to 1000 Da (e.g. for $M = 14179$, the anchor ion would be $z = 14$, $m/z = \frac{14179+14}{14} = 1013.79$), and ranging from two z-values below (e.g. $z = 12$, $m/z = \frac{14179+12}{12} = 1182.59$) to 18 z-values above the anchor (e.g. $z = 32$, $m/z = \frac{14179+32}{32} = 444.10$). In parallel, the theoretical span of masses for the species arising from isotopic variability was predicted using an estimation of the number of carbon atoms and a 1.11 % natural abundance of carbon-13. Based on this, each peak was given the predicted width of this span divided by the corresponding z-value. Using these predictions and an iterative process incorporated the recorded data for the sample, these predictions were refined to catch as many ion peaks as possible without including m/z values arising from other species in solution or from measurement noise.

Once predictions were refined, the corresponding m/z ranges were sliced out from the 3-dimensional spectrum and marginalised over the m/z axis to form a 2-dimensional extracted ion chromatogram (XIC) representing the species. The process was repeated for each relevant species. To further increase signal-to-noise ratios, the retention time (rt) range corresponding to a group of species (e.g. all nanobody derivatives) was identified by manual inspection of the XICs.

Quantification of species followed directly by summing their corresponding XICs in the identified rt range. As discussed, the absolute values derived from this quantification cannot be used directly, but the relative values within a group of species was found to

capture their relative abundances well, and was consistent across independent experiments conduced over a range of months or years and a range of concentrations and injection volumes.

Surface plasmon resonance (SPR)

SPR analysis was carried out on a Biacore™ T200 instrument (GE Healthcare). Nanobodies were immobilised on a Series S Sensor Chip CM5 (Cytiva): the chip surface was primed with the running buffer (PBS pH 7.4 + 0.01% Tween-20) and activated with 50 mM N-hydroxysuccinimide (NHS) and 200 mM 1-ethyl-3-(3-dimethylaminopropyl) carbodiimide hydrochloride (EDC) at a flow rate of 10 µL/min for 420 seconds. The ligand was diluted to 1 µM in 10 mM sodium acetate, pH 5.5 and loaded onto the sample flow cell surface at a flow rate of 5 µL/min in short injections totalling between 540–840 seconds. The sensor chip surface was deactivated with 1 M ethanolamine·HCl, pH 8.5 at a flow rate of 10 µL/min for 420 seconds. Surface activity was tested by performing four injections of 30 seconds each of 10 mM glycine, pH 2.0 at a flow rate of 30 µL/min.

Recombinant GFP analyte was injected in 7 concentrations ranging 0.03–333 nM in duplicate, at a flow rate of 30 µL/min for 240 seconds, followed by a dissociation time of 1200 seconds. After each cycle, the sensor surface was regenerated by performing two injections of 30 seconds each of 10 mM glycine, pH 2.0 at a flow rate of 30 µL/min, followed by a stabilisation period of 60 seconds. The binding sensorgrams were fitted according to a 1 : 1 binding model and the data were processed using Biacore T200 Evaluation software.

Biolayer interferometry (BLI)

BLI measurements were conducted using an Octet-BLI K2 system (ForteBio) with streptavidin (SA) sensors. To prevent non-specific interactions with the sensors, all experiments were carried out in PBS supplemented with 0.05% Tween-20 (Sigma). A black 96-well plate (Greiner 655209) was used, with 200 µl per well, and the sensors were pre-hydrated in the assay buffer for at least 15 minutes before use. The assay plate was maintained at 30°C / 1000 rpm. The loading wells were loaded with 50 nM of biotinylated β2-microglobulin, as previously described [12].

Each experimental run consisted of a baseline step, a loading step, a second baseline step, followed by multiple association and brief dissociation steps. The association phase was observed in wells containing six different concentrations of nb24 nanobody variants ranging 25–400 nM. In all experiments, a reference sensor, treated in the same way as the assay sensors but placed in buffer wells during association steps, was employed. Its signal was subtracted from the signal of each assay sensor before data analysis. The binding data were then globally fitted with a 1:1 partial dissociation binding model, using Rmax, on-rate, and off-rate as global parameters, and $\mathtt{Y}_{t\to\mathrm{inf}}$ as a local parameter.

Small molecule nuclear magnetic resonance (NMR) spectroscopy

Samples for NMR were dissolved in deuterated solvents as indicated, at concentrations ranging from 10 mg/mL (short ^{1}H spectra) to 50 mg/mL (overnight ^{13}C or other complex spectra). Once fully dissolved, samples were transferred to Wilmad 528-PP 7 inch × 5 mm NMR tubes and loaded onto a 400 MHz Avance III HD Smart Probe spectrometer (Bruker), Bruker 500-AVIII Dual 13C 1H cryoprobe, Bruker 500- AVIII or Bruker 700-AVII+ TCO cryoprobe, as indicated. Data were analysed using Bruker TopSpin. Chemical shifts are reported in parts per million (ppm) with spectra calibrated to the residual solvent peaks (^{1}H-NMR: $CDCl_3$ δH 7.26 ppm, DMSO-d_6 δH 2.50 ppm, MeOD δH 3.31 ppm, acteone-d_6 δH 2.05 ppm, ^{13}C-NMR: $CDCl_3$ δC 77.2 ppm, DMSO-d_6 δC 39.5 ppm, MeOD δC 49.0 ppm, acteone-d_6 δC 29.8 ppm). All NMR measurements were acquired at 298 K.

Small molecule preperative HPLC

High Performance Liquid Chromatography (HPLC) was performed on an Agilent Infinity 1260 II system with a semi-preparative Column ZORBAX 5 Eclipse Plus C18 21.2 × 250 mm (ID x L) P. N. 595250-902. The crude product was dissolved in 1 : 1 acetonitrile (ACN) : water and sterile-filtered at 0.2 µm with EMD Millipore™ polyethersulfone syringe filters. Crude product aliquots were injected into the column and flow was maintained at 20.0 mL/min with solvent mixtures of ACN/H_2O + 0.1 % TFA. UV-vis absorbance was monitored at 210 and 254 nm to indicate when to begin manual collection of fractions. ACN was evaporated from the fractions under a nitrogen flow, and the remaining water/TFA solution was flash frozen and lyophilised.

Small molecule LC-MS

Samples were prepared by dissolving in water or water:acetonitrile mixtures of up to 90 % acetonitrile. Once fully dissolved, the sample was filtered using a 4 mm PVDF syringe filter with a 0.22 µm pore size (Millipore) into a 300 µL screw thread vial (Supelco) and covered with a pre-slit screw thread cap (Fisher Scientific). Samples were injected on an Acquity UPLC system equiped with an Acquity UPLC BEH C18 column (130 Å pore diameter, 1.7 µm, 2.1 mm × 50 mm). Solvent A consisted of 0.1 % formic acid in water, and solvent B of 0.1 % formic acid in acetonitrile. At a flow rate of 0.37 mL/min, a gradient of 8.00 min was utilised (hold at 10 % B for 0.38 min, increase to 100 % B over 6.8 min, hold for another 0.38 min then decrease back to 10 % B over 0.44 min). Data were recorded on a Waters Xevo G2-S qTOF mass spectrometer using an electrospray ionisation (ESI) source at 120 °C with a capillary voltage of 3.0 kV and a cone voltage of 40 V. Nitrogen was used as the desolvation gas at a flow rate of 600 L/h. LockSpray was infused throughout the measurement for internal calibration.

Instrument Reports

File Properties

Evaluation File
Name: N/A

User Information
Performed By: Administrator
Current User: Administrator

Created With Software
Name: Biacore T200 Evaluation Software
Version: 1.0

Notebook

Result

Result File
Name: 20230225_MCK_GFP-analyte_aGFP-RSGGH-ligand_FC4-3.blr
Path: C:\Bia Users\Vaidehi vr358
Size: 6 625 280 bytes

Run Information
Type: Kinetics/Affinity
Cycles: 21
Start: 3/25/2023 7:43:02 PM
End: 3/26/2023 7:44:59 AM

Instrument
Instrument Type: BiacoreT200
Instrument Id: 1646191
IFC: TYPE105

User Information
Run Performed By: Administrator

Created With Software
Name: Biacore T200 Control Software
Version: 1.0

Chip Information
Chip Id: Vaidehi 230321 aGFP wt
Chip Lot No: 10335900
Chip Name: CM5
First Dock Date: 3/21/2023 1:14:13 PM
Last Modification Date: 3/21/2023 1:14:13 PM
Last Use Date: 3/25/2023 11:26:54 AM

Immobilization in Fc=1
Immobilization Date:
Immobilization Result:
Ligand:
Final Response [RU]:

Immobilization in Fc=2
Immobilization Date:
Immobilization Result:
Ligand:
Final Response [RU]:

Immobilization in Fc=3
Immobilization Date:
Immobilization Result:
Ligand:
Final Response [RU]:

Immobilization in Fc=4
Immobilization Date:

File Properties (continued)

Immobilization Result:
Ligand:
Final Response [RU]:

Notebook

Kinetics: 'GFP 2', fit: '1. 1:1 Binding'

Curve: Fc=4-3 Ligand: N/A Sample: GFP Temp: 25°C

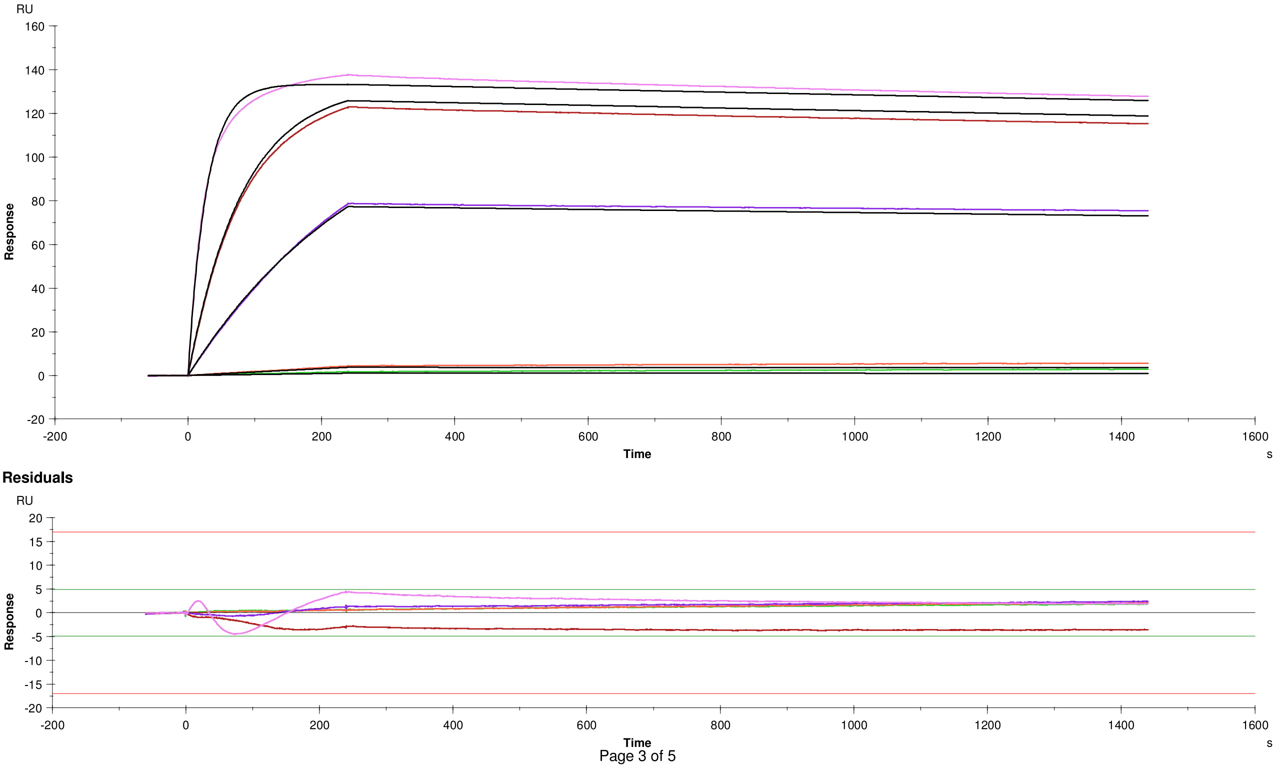

Kinetics: ‘GFP 2’, fit: ‘1. 1:1 Binding’ (continued)

Quality Control

✓	Kinetic constants are within instrument specifications.
✓	Kinetic constants appear to be uniquely determined.
➡	Bulk contributions (RI) were not evaluated. The RI parameter is set to constant.
➡	Check that sensorgrams have sufficient curvature.
➡	Examine the residual plot. Pay attention to systematic and non-random deviations.

Kinetics: ‘GFP 2’, fit: ‘1. 1:1 Binding’ (continued)

Report table

Curve	ka (1/Ms)	kd (1/s)	KD (M)	Rmax (RU)	Conc (M)	tc	Flow (ul/min)	kt (RU/Ms)	RI (RU)	Chi^2 (RU^2)	U-value
	1.093E+6	4.756E-5	4.350E-11	133.5		1.820E+19				4.83	7
Cycle: 6 0.03 nM					3.000E-11		30.00	5.655E+19	0.000		
Cycle: 7 0.11 nM					1.100E-10		30.00	5.655E+19	0.000		
Cycle: 8 3.33 nM					3.330E-9		30.00	5.655E+19	0.000		
Cycle: 9 11.11 nM					1.111E-8		30.00	5.655E+19	0.000		
Cycle: 10 33.33 nM					3.333E-8		30.00	5.655E+19	0.000		

Parameters table

Curve	ka (1/Ms)	SE(ka)	kd (1/s)	SE(kd)	Rmax (RU)	SE(Rmax)	Conc (M)	tc	SE(tc)	f (ul/min)	RI (RU)
	1.093E+6	4.9E+2	4.756E-5	2.5E-7	133.5	0.026		1.820E+19	5.7E+21		
Cycle: 6 0.03 nM							3E-11			30	0.0
Cycle: 7 0.11 nM							1.1E-10			30	0.0
Cycle: 8 3.33 nM							3.33E-09			30	0.0
Cycle: 9 11.11 nM							1.111E-08			30	0.0
Cycle: 10 33.33 nM							3.333E-08			30	0.0

File Properties

Evaluation File

Name:	20230221_MCK_GFP-analyte_aGFP-S54C-RSGGH_FC4-3_333-removed.bme
Path:	C:\Bia Users\Vaidehi vr358
Size:	10 861 056

User Information

Performed By:	Administrator
Current User:	Administrator

Created With Software

Name:	Biacore T200 Evaluation Software
Version:	1.0

Notebook

Result

Result File

Name:	20230221_MCK_GFP-analyte_aGFP-S54C-RSGGH_FC4-3.blr
Path:	C:\Bia Users\Vaidehi vr358
Size:	6 625 280 bytes

Run Information

Type:	Kinetics/Affinity
Cycles:	21
Start:	2/21/2023 8:02:06 PM
End:	2/22/2023 7:03:41 AM

Instrument

Instrument Type:	BiacoreT200
Instrument Id:	1646191
IFC:	TYPE105

User Information

Run Performed By:	Administrator

Created With Software

Name:	Biacore T200 Control Software
Version:	1.0

Chip Information

Chip Id:	230220-1251:Vaidehi
Chip Lot No:	10331800
Chip Name:	CM5
First Dock Date:	2/20/2023 12:54:32 PM
Last Modification Date:	2/20/2023 12:54:32 PM
Last Use Date:	2/21/2023 2:32:53 PM

Immobilization in Fc=1

Immobilization Date:
Immobilization Result:
Ligand:
Final Response [RU]:

Immobilization in Fc=2

Immobilization Date:
Immobilization Result:
Ligand:
Final Response [RU]:

Immobilization in Fc=3

Immobilization Date:
Immobilization Result:
Ligand:
Final Response [RU]:

File Properties (continued)

Immobilization in Fc=4
Immobilization Date:
Immobilization Result:
Ligand:
Final Response [RU]:

Notebook

Kinetics: 'GFP 2', fit: '1. 1:1 Binding'

Curve: Fc=4-3 Ligand: N/A Sample: GFP Temp: 25°C

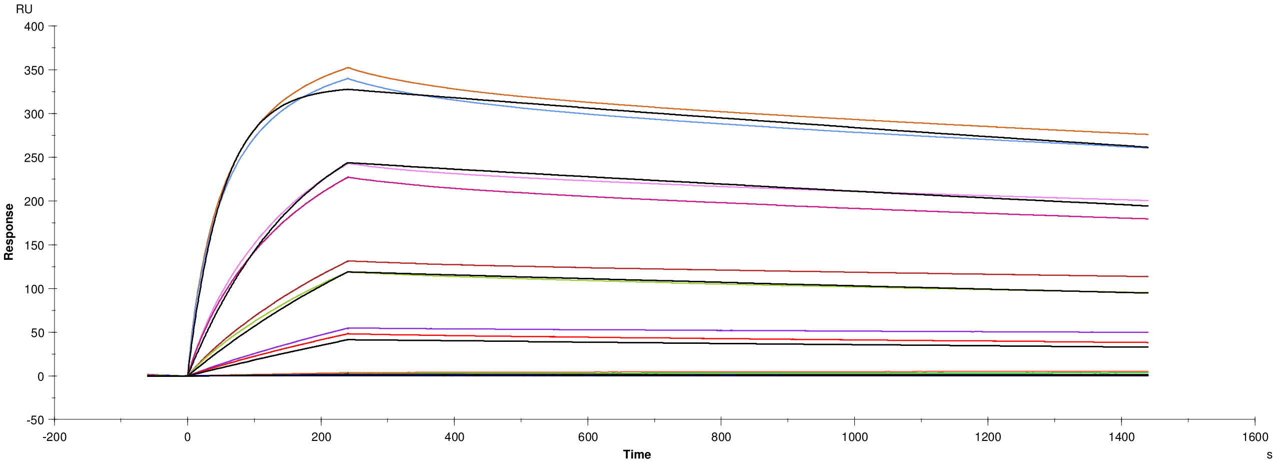

Residuals

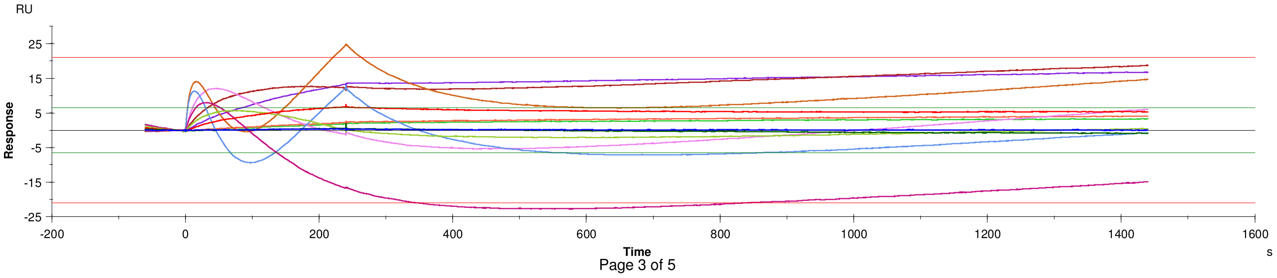

Kinetics: 'GFP 2', fit: '1. 1:1 Binding' (continued)

Quality Control

✓	Kinetic constants are within instrument specifications.
✓	Kinetic constants appear to be uniquely determined.
⇨	Bulk contributions (RI) were not evaluated. The RI parameter is set to constant.
⇨	Check that sensorgrams have sufficient curvature.
⇨	Examine the residual plot. Pay attention to systematic and non-random deviations.

Kinetics: 'GFP 2', fit: '1. 1:1 Binding' (continued)

Report table

Curve	ka (1/Ms)	kd (1/s)	KD (M)	Rmax (RU)	Conc (M)	tc	Flow (ul/min)	kt (RU/Ms)	RI (RU)	Chi² (RU²)	U-value
	1.690E+5	1.883E-4	1.115E-9	334.7		8.011E+17				77.5	4
Cycle: 6 0.03 nM					3.000E-11		30.00	2.489E+18	0.000		
Cycle: 7 0.11 nM					1.100E-10		30.00	2.489E+18	0.000		
Cycle: 8 3.33 nM					3.330E-9		30.00	2.489E+18	0.000		
Cycle: 9 11.11 nM					1.111E-8		30.00	2.489E+18	0.000		
Cycle: 10 33.33 nM					3.333E-8		30.00	2.489E+18	0.000		
Cycle: 11 111.11 nM					1.111E-7		30.00	2.489E+18	0.000		
Cycle: 14 111.11 nM					1.111E-7		30.00	2.489E+18	0.000		
Cycle: 15 33.33 nM					3.333E-8		30.00	2.489E+18	0.000		
Cycle: 16 11.11 nM					1.111E-8		30.00	2.489E+18	0.000		
Cycle: 17 3.33 nM					3.330E-9		30.00	2.489E+18	0.000		
Cycle: 18 0.11 nM					1.100E-10		30.00	2.489E+18	0.000		
Cycle: 19 0.03 nM					3.000E-11		30.00	2.489E+18	0.000		

Parameters table

Curve	ka (1/Ms)	SE(ka)	kd (1/s)	SE(kd)	Rmax (RU)	SE(Rmax)	Conc (M)	tc	SE(tc)	f (ul/min)	RI (RU)
	1.690E+5	91	1.883E-4	3.7E-7	334.7	0.096		8.011E+17	1.9E+20		
Cycle: 6 0.03 nM							3E-11			30	0.0
Cycle: 7 0.11 nM							1.1E-10			30	0.0
Cycle: 8 3.33 nM							3.33E-09			30	0.0
Cycle: 9 11.11 nM							1.111E-08			30	0.0
Cycle: 10 33.33 nM							3.333E-08			30	0.0
Cycle: 11 111.11 nM							1.111E-07			30	0.0
Cycle: 14 111.11 nM							1.111E-07			30	0.0
Cycle: 15 33.33 nM							3.333E-08			30	0.0
Cycle: 16 11.11 nM							1.111E-08			30	0.0
Cycle: 17 3.33 nM							3.33E-09			30	0.0
Cycle: 18 0.11 nM							1.1E-10			30	0.0
Cycle: 19 0.03 nM							3E-11			30	0.0

File Properties

Evaluation File

Name:	20230425_MCK_GFP-analyte_aGFP-S54C-RSGGH-2b-ligand_FC2-1_Run-2.bme
Path:	C:\Bia Users\Vaidehi vr358
Size:	21 491 200

User Information

Performed By:	Administrator
Current User:	Administrator

Created With Software

Name:	Biacore T200 Evaluation Software
Version:	1.0

Notebook

Result

Result File

Name:	20230425_MCK_GFP-analyte_aGFP-S54C-RSGGH-2b-ligand_FC2-1_Run-2.blr
Path:	C:\Bia Users\Vaidehi vr358
Size:	15 421 952 bytes

Run Information

Type:	Kinetics/Affinity
Cycles:	25
Start:	4/26/2023 4:47:36 AM
End:	4/27/2023 6:53:38 AM

Instrument

Instrument Type:	BiacoreT200
Instrument Id:	1646191
IFC:	TYPE105

User Information

Run Performed By:	Administrator

Created With Software

Name:	Biacore T200 Control Software
Version:	1.0

Chip Information

Chip Id:	230425:Vaidehi PEABS*
Chip Lot No:	10331800
Chip Name:	CM5
First Dock Date:	4/25/2023 1:51:16 PM
Last Modification Date:	4/25/2023 1:51:16 PM
Last Use Date:	4/25/2023 1:52:41 PM

Immobilization in Fc=1

Immobilization Date:
Immobilization Result:
Ligand:
Final Response [RU]:

Immobilization in Fc=2

Immobilization Date:
Immobilization Result:
Ligand:
Final Response [RU]:

Immobilization in Fc=3

Immobilization Date:
Immobilization Result:
Ligand:
Final Response [RU]:

File Properties (continued)

Immobilization in Fc=4
Immobilization Date:
Immobilization Result:
Ligand:
Final Response [RU]:

Notebook

C:\Bia Users\Vaidehi vr358\20230425_MCK_GFP-analyte_aGFP-S54C-RSGGH-2b-ligand_FC2-1_Run-2.bme (unsaved)

Instr. id: 1646191 Biacore T200 Evaluation Software, version 1.0 Print date: 10/30/2023 4:36:33 AM

Kinetics: 'GFP 3', fit: '1. 1:1 Binding'

Curve: Fc=2-1 Ligand: N/A Sample: GFP Temp: 25°C

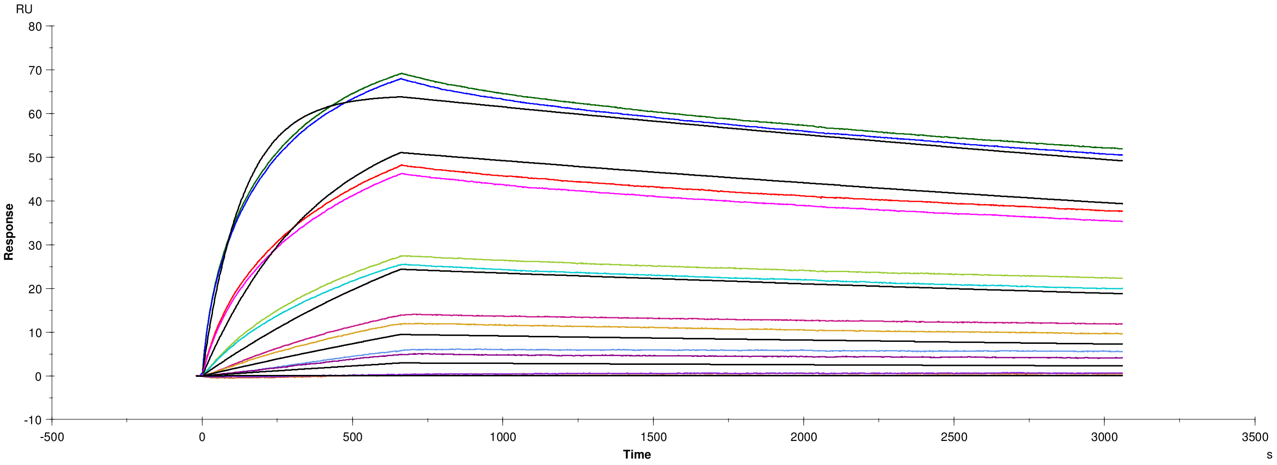

Residuals

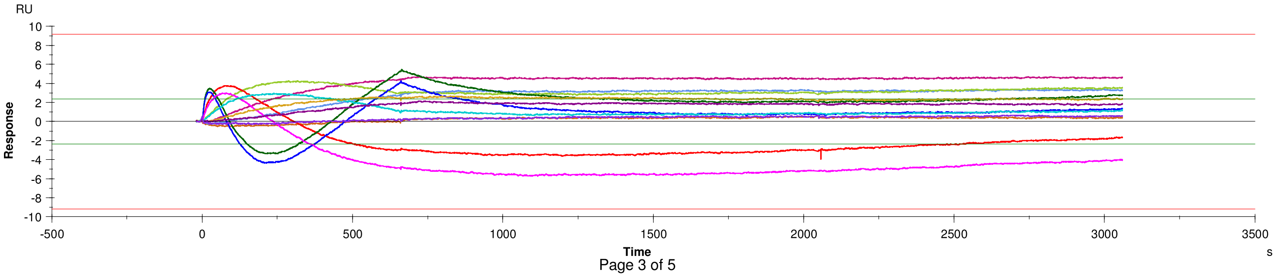

Kinetics: 'GFP 3', fit: '1. 1:1 Binding' (continued)

Quality Control

✓	Kinetic constants are within instrument specifications.
✓	Kinetic constants appear to be uniquely determined.
⇨	Bulk contributions (RI) were not evaluated. The RI parameter is set to constant.
⇨	Check that sensorgrams have sufficient curvature.
⇨	Examine the residual plot. Pay attention to systematic and non-random deviations.

Kinetics: ‘GFP 3’, fit: ‘1. 1:1 Binding’ (continued)

Report table

Curve	ka (1/Ms)	kd (1/s)	KD (M)	Rmax (RU)	Conc (M)	tc	Flow (ul/min)	kt (RU/Ms)	RI (RU)	Chi² (RU²)	U-value
	2.222E+5	1.087E-4	4.894E-10	65.25		2.139E+18				7.31	5
Cycle: 7 0.01 nM					1.000E-11		30.00	6.647E+18	0.000		
Cycle: 10 0.33 nM					3.300E-10		30.00	6.647E+18	0.000		
Cycle: 11 1.11 nM					1.110E-9		30.00	6.647E+18	0.000		
Cycle: 12 3.33 nM					3.330E-9		30.00	6.647E+18	0.000		
Cycle: 13 11.11 nM					1.111E-8		30.00	6.647E+18	0.000		
Cycle: 14 33.33 nM					3.333E-8		30.00	6.647E+18	0.000		
Cycle: 15 33.33 nM					3.333E-8		30.00	6.647E+18	0.000		
Cycle: 16 11.11 nM					1.111E-8		30.00	6.647E+18	0.000		
Cycle: 17 3.33 nM					3.330E-9		30.00	6.647E+18	0.000		
Cycle: 18 1.11 nM					1.110E-9		30.00	6.647E+18	0.000		
Cycle: 19 0.33 nM					3.300E-10		30.00	6.647E+18	0.000		
Cycle: 22 0.01 nM					1.000E-11		30.00	6.647E+18	0.000		

Parameters table

Curve	ka (1/Ms)	SE(ka)	kd (1/s)	SE(kd)	Rmax (RU)	SE(Rmax)	Conc (M)	tc	SE(tc)	f (ul/min)	RI (RU)
	2.222E+5	1.3E+2	1.087E-4	1.9E-7	65.3	0.020		2.139E+18	1.1E+21		
Cycle: 7 0.01 nM							1E-11			30	0.0
Cycle: 10 0.33 nM							3.3E-10			30	0.0
Cycle: 11 1.11 nM							1.11E-09			30	0.0
Cycle: 12 3.33 nM							3.33E-09			30	0.0
Cycle: 13 11.11 nM							1.111E-08			30	0.0
Cycle: 14 33.33 nM							3.333E-08			30	0.0
Cycle: 15 33.33 nM							3.333E-08			30	0.0
Cycle: 16 11.11 nM							1.111E-08			30	0.0
Cycle: 17 3.33 nM							3.33E-09			30	0.0
Cycle: 18 1.11 nM							1.11E-09			30	0.0
Cycle: 19 0.33 nM							3.3E-10			30	0.0
Cycle: 22 0.01 nM							1E-11			30	0.0

File Properties

Evaluation File
Name: N/A

User Information
Performed By: Administrator
Current User: Administrator

Created With Software
Name: Biacore T200 Evaluation Software
Version: 1.0

Notebook

Result

Result File
Name: 20230831_MCK_FC2-1_3-1_4-1_aGFPS54C-mutants.blr
Path: C:\Bia Users\Vaidehi vr358
Size: 25 669 120 bytes

Run Information
Type: Method Builder
Method: C:\Bia Users\Methods And Templates\Vaidehi vr358\20230902_MCK_2-1_3-1_4-1_METHOD.Method
Cycles: 19
Start: 9/2/2023 9:41:03 PM
End: 9/3/2023 3:12:01 PM

Instrument
Instrument Type: BiacoreT200
Instrument Id: 1646191
IFC: TYPE105

User Information
Run Performed By: Administrator

Created With Software
Name: Biacore T200 Control Software
Version: 1.0

Chip Information
Chip Id: 230806-0953: Vaidehi CM5
Chip Lot No:
Chip Name: CM5
First Dock Date: 8/6/2023 9:54:13 PM
Last Modification Date: 8/6/2023 9:54:13 PM
Last Use Date: 8/31/2023 5:44:33 PM

Immobilization in Fc=1
Immobilization Date:
Immobilization Result:
Ligand:
Final Response [RU]:

Immobilization in Fc=2
Immobilization Date:
Immobilization Result:
Ligand:
Final Response [RU]:

Immobilization in Fc=3
Immobilization Date:
Immobilization Result:
Ligand:
Final Response [RU]:

Immobilization in Fc=4

File Properties (continued)

Immobilization Date:
Immobilization Result:
Ligand:
Final Response [RU]:

Notebook

Kinetics: 'M1_FC4-1', fit: '1. 1:1 Binding'

Curve: Fc=4-1 Ligand: N/A Sample: GFP Temp: 25°C

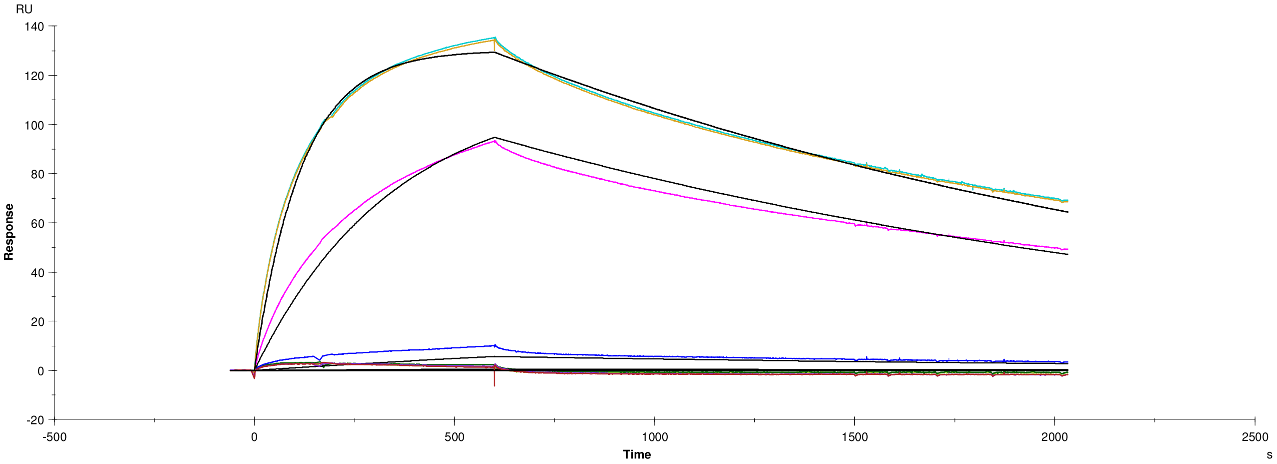

Residuals

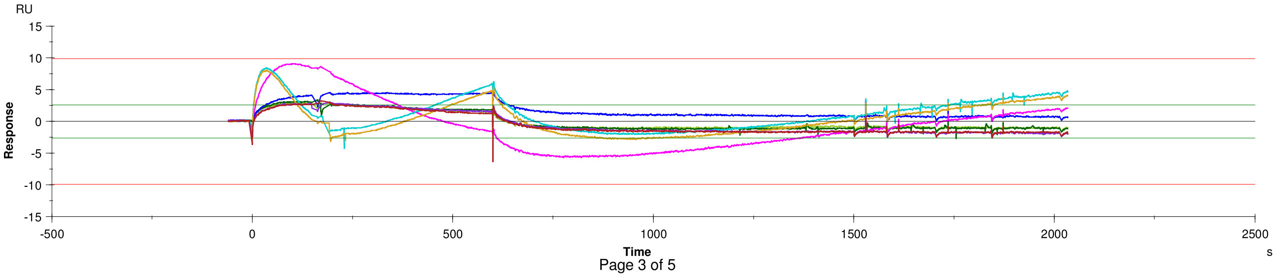

Kinetics: ‘M1_FC4-1’, fit: ‘1. 1:1 Binding’ (continued)

Quality Control

✓	Kinetic constants are within instrument specifications.
✓	Kinetic constants appear to be uniquely determined.
➡	Bulk contributions (RI) were not evaluated. The RI parameter is set to constant.
➡	Check that sensorgrams have sufficient curvature.
➡	Examine the residual plot. Pay attention to systematic and non-random deviations.

Kinetics: ‘M1_FC4-1’, fit: ‘1. 1:1 Binding’ (continued)

Report table

Curve	ka (1/Ms)	kd (1/s)	KD (M)	Rmax (RU)	Conc (M)	tc	Flow (ul/min)	kt (RU/Ms)	RI (RU)	Chi² (RU²)	U-value
	7.248E+4	4.868E-4	6.716E-9	138.1		3.583E+17				5.65	1
Cycle: 6 0.003 nM					3.000E-12		30.00	1.113E+18	0.000		
Cycle: 8 0.11 nM					1.100E-10		30.00	1.113E+18	0.000		
Cycle: 9 1.11 nM					1.110E-9		30.00	1.113E+18	0.000		
Cycle: 10 33.33 nM					3.333E-8		30.00	1.113E+18	0.000		
Cycle: 11 111.11 nM					1.111E-7		30.00	1.113E+18	0.000		
Cycle: 12 111.11 nM					1.111E-7		30.00	1.113E+18	0.000		
Cycle: 16 0.03 nM					3.000E-11		30.00	1.113E+18	0.000		
Cycle: 17 0.003 nM					3.000E-12		30.00	1.113E+18	0.000		

Parameters table

Curve	ka (1/Ms)	SE(ka)	kd (1/s)	SE(kd)	Rmax (RU)	SE(Rmax)	Conc (M)	tc	SE(tc)	f (ul/min)	RI (RU)
	7.248E+4	43	4.868E-4	2.6E-7	138.1	0.030		3.583E+17	1.6E+20		
Cycle: 6 0.003 nM							3E-12			30	0.0
Cycle: 8 0.11 nM							1.1E-10			30	0.0
Cycle: 9 1.11 nM							1.11E-09			30	0.0
Cycle: 10 33.33 nM							3.333E-08			30	0.0
Cycle: 11 111.11 nM							1.111E-07			30	0.0
Cycle: 12 111.11 nM							1.111E-07			30	0.0
Cycle: 16 0.03 nM							3E-11			30	0.0
Cycle: 17 0.003 nM							3E-12			30	0.0

File Properties

Evaluation File

Name:	N/A

User Information

Performed By:	Administrator
Current User:	Administrator

Created With Software

Name:	Biacore T200 Evaluation Software
Version:	1.0

Notebook

Result

Result File

Name:	20230831_MCK_FC2-1_3-1_4-1_aGFPS54C-mutants.blr
Path:	C:\Bia Users\Vaidehi vr358
Size:	25 669 120 bytes

Run Information

Type:	Method Builder
Method:	C:\Bia Users\Methods And Templates\Vaidehi vr358\20230902_MCK_2-1_3-1_4-1_METHOD.Method
Cycles:	19
Start:	9/2/2023 9:41:03 PM
End:	9/3/2023 3:12:01 PM

Instrument

Instrument Type:	BiacoreT200
Instrument Id:	1646191
IFC:	TYPE105

User Information

Run Performed By:	Administrator

Created With Software

Name:	Biacore T200 Control Software
Version:	1.0

Chip Information

Chip Id:	230806-0953: Vaidehi CM5
Chip Lot No:	
Chip Name:	CM5
First Dock Date:	8/6/2023 9:54:13 PM
Last Modification Date:	8/6/2023 9:54:13 PM
Last Use Date:	8/31/2023 5:44:33 PM

Immobilization in Fc=1

Immobilization Date:
Immobilization Result:
Ligand:
Final Response [RU]:

Immobilization in Fc=2

Immobilization Date:
Immobilization Result:
Ligand:
Final Response [RU]:

Immobilization in Fc=3

Immobilization Date:
Immobilization Result:
Ligand:
Final Response [RU]:

Immobilization in Fc=4

File Properties (continued)

Immobilization Date:
Immobilization Result:
Ligand:
Final Response [RU]:

Notebook

Kinetics: 'M3_FC3-1', fit: '1. 1:1 Binding'

Curve: Fc=3-1 Ligand: N/A Sample: GFP Temp: 25°C

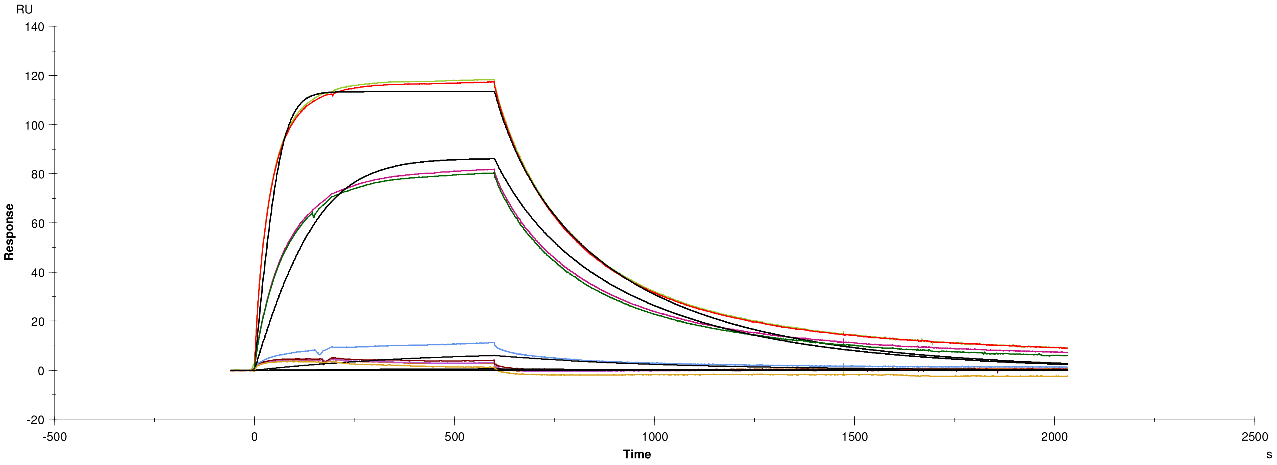

Residuals

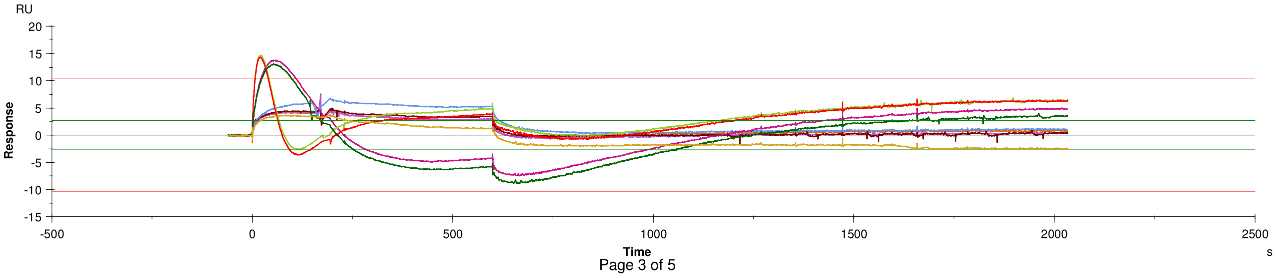

Kinetics: ‘M3_FC3-1’, fit: ‘1. 1:1 Binding’ (continued)

Quality Control

✓	Kinetic constants are within instrument specifications.
✓	Kinetic constants appear to be uniquely determined.
➔	Bulk contributions (RI) were not evaluated. The RI parameter is set to constant.
➔	Check that sensorgrams have sufficient curvature.
➔	Examine the residual plot. Pay attention to systematic and non-random deviations.

Kinetics: ‘M3_FC3-1’, fit: ‘1. 1:1 Binding’ (continued)

Report table

Curve	ka (1/Ms)	kd (1/s)	KD (M)	Rmax (RU)	Conc (M)	tc	Flow (ul/min)	kt (RU/Ms)	RI (RU)	Chi² (RU²)	U-value
	3.827E+5	0.006635	1.733E-8	131.2		8.025E+6				12.0	5
Cycle: 6 0.003 nM					3.000E-12		30.00	2.493E+7	0.000		
Cycle: 7 0.03 nM					3.000E-11		30.00	2.493E+7	0.000		
Cycle: 8 0.11 nM					1.100E-10		30.00	2.493E+7	0.000		
Cycle: 9 1.11 nM					1.110E-9		30.00	2.493E+7	0.000		
Cycle: 10 33.33 nM					3.333E-8		30.00	2.493E+7	0.000		
Cycle: 11 111.11 nM					1.111E-7		30.00	2.493E+7	0.000		
Cycle: 12 111.11 nM					1.111E-7		30.00	2.493E+7	0.000		
Cycle: 13 33.33 nM					3.333E-8		30.00	2.493E+7	0.000		
Cycle: 17 0.003 nM					3.000E-12		30.00	2.493E+7	0.000		

Parameters table

Curve	ka (1/Ms)	SE(ka)	kd (1/s)	SE(kd)	Rmax (RU)	SE(Rmax)	Conc (M)	tc	SE(tc)	f (ul/min)	RI (RU)
	3.827E+5	1.6E+2	0.006635	2.5E-6	131.2	0.030		8.025E+6	3.3E+3		
Cycle: 6 0.003 nM							3E-12			30	0.0
Cycle: 7 0.03 nM							3E-11			30	0.0
Cycle: 8 0.11 nM							1.1E-10			30	0.0
Cycle: 9 1.11 nM							1.11E-09			30	0.0
Cycle: 10 33.33 nM							3.333E-08			30	0.0
Cycle: 11 111.11 nM							1.111E-07			30	0.0
Cycle: 12 111.11 nM							1.111E-07			30	0.0
Cycle: 13 33.33 nM							3.333E-08			30	0.0
Cycle: 17 0.003 nM							3E-12			30	0.0

File Properties

Evaluation File
Name: N/A

User Information
Performed By: Administrator
Current User: Administrator

Created With Software
Name: Biacore T200 Evaluation Software
Version: 1.0

Notebook

Result

Result File
Name: 20231027-GFP-mutants-analyte-1-5_aGFP-S54C-RSGGH_FC2-1.blr
Path: C:\Bia Users\Vaidehi vr358
Size: 24 225 280 bytes

Run Information
Type: Kinetics/Affinity
Cycles: 70
Start: 10/27/2023 1:54:59 PM
End: 10/29/2023 5:29:48 AM

Instrument
Instrument Type: BiacoreT200
Instrument Id: 1646191
IFC: TYPE105

User Information
Run Performed By: Administrator

Created With Software
Name: Biacore T200 Control Software
Version: 1.0

Chip Information
Chip Id: 231023 Vaidehi CM5
Chip Lot No: 10345041
Chip Name: CM5
First Dock Date: 10/23/2023 11:57:59 PM
Last Modification Date: 10/23/2023 11:57:59 PM
Last Use Date: 10/23/2023 11:59:23 PM

Immobilization in Fc=1
Immobilization Date:
Immobilization Result:
Ligand:
Final Response [RU]:

Immobilization in Fc=2
Immobilization Date:
Immobilization Result:
Ligand:
Final Response [RU]:

Immobilization in Fc=3
Immobilization Date:
Immobilization Result:
Ligand:
Final Response [RU]:

Immobilization in Fc=4
Immobilization Date:

File Properties (continued)

Immobilization Result:
Ligand:
Final Response [RU]:

Notebook

Kinetics: ‘41’, fit: ‘1. 1:1 Binding’

Curve: Fc=2-1 Ligand: N/A Sample: 41 Temp: 25°C

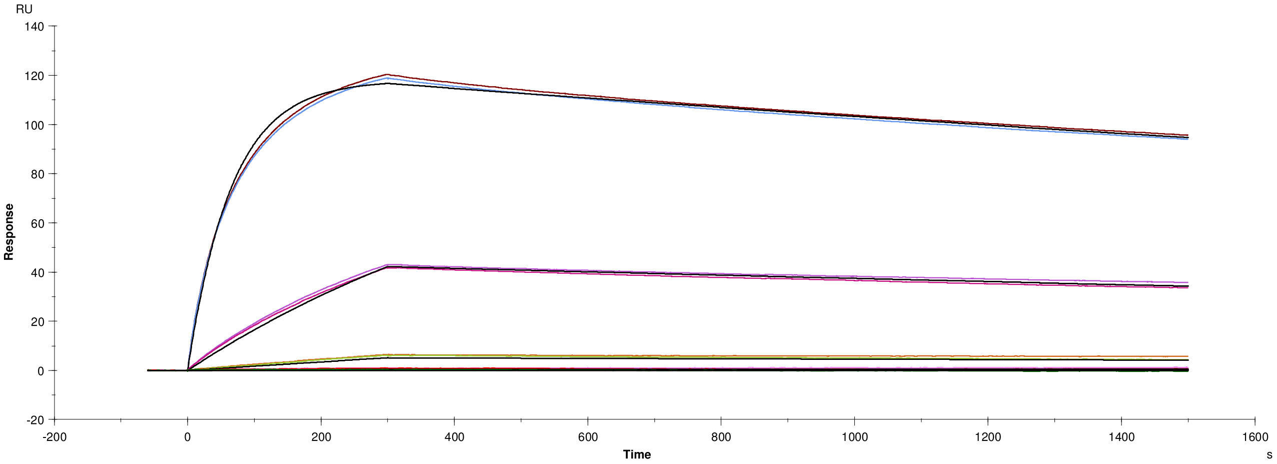

Residuals

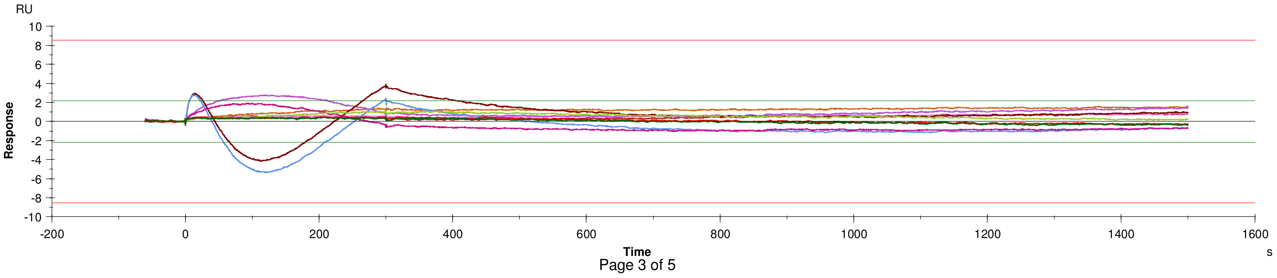

Kinetics: ‘41’, fit: ‘1. 1:1 Binding’ (continued)

Quality Control

✓	Kinetic constants are within instrument specifications.
✓	Kinetic constants appear to be uniquely determined.
➔	Bulk contributions (RI) were not evaluated. The RI parameter is set to constant.
➔	Check that sensorgrams have sufficient curvature.
➔	Examine the residual plot. Pay attention to systematic and non-random deviations.

Kinetics: ‘41’, fit: ‘1. 1:1 Binding’ (continued)

Report table

Curve	ka (1/Ms)	kd (1/s)	KD (M)	Rmax (RU)	Conc (M)	tc	Flow (ul/min)	kt (RU/Ms)	RI (RU)	Chi² (RU²)	U-value
	1.503E+5	1.740E-4	1.158E-9	119.3		3.725E+17				1.01	2
Cycle: 3 0.01 nM					1.000E-11		30.00	1.158E+18	0.000		
Cycle: 4 0.1 nM					1.000E-10		30.00	1.158E+18	0.000		
Cycle: 5 1 nM					1.000E-9		30.00	1.158E+18	0.000		
Cycle: 6 10 nM					1.000E-8		30.00	1.158E+18	0.000		
Cycle: 7 100 nM					1.000E-7		30.00	1.158E+18	0.000		
Cycle: 8 100 nM					1.000E-7		30.00	1.158E+18	0.000		
Cycle: 9 10 nM					1.000E-8		30.00	1.158E+18	0.000		
Cycle: 10 1 nM					1.000E-9		30.00	1.158E+18	0.000		
Cycle: 11 0.1 nM					1.000E-10		30.00	1.158E+18	0.000		
Cycle: 12 0.01 nM					1.000E-11		30.00	1.158E+18	0.000		

Parameters table

Curve	ka (1/Ms)	SE(ka)	kd (1/s)	SE(kd)	Rmax (RU)	SE(Rmax)	Conc (M)	tc	SE(tc)	f (ul/min)	RI (RU)
	1.503E+5	33	1.740E-4	1.4E-7	119.3	0.012		3.725E+17	6.2E+19		
Cycle: 3 0.01 nM							1E-11			30	0.0
Cycle: 4 0.1 nM							1E-10			30	0.0
Cycle: 5 1 nM							1E-09			30	0.0
Cycle: 6 10 nM							1E-08			30	0.0
Cycle: 7 100 nM							1E-07			30	0.0
Cycle: 8 100 nM							1E-07			30	0.0
Cycle: 9 10 nM							1E-08			30	0.0
Cycle: 10 1 nM							1E-09			30	0.0
Cycle: 11 0.1 nM							1E-10			30	0.0
Cycle: 12 0.01 nM							1E-11			30	0.0

File Properties

Evaluation File
Name: N/A

User Information
Performed By: Administrator
Current User: Administrator

Created With Software
Name: Biacore T200 Evaluation Software
Version: 1.0

Notebook

Result

Result File
Name: 20231027-GFP-mutants-analyte-1-5_aGFP-S54C-RSGGH_FC2-1.blr
Path: C:\Bia Users\Vaidehi vr358
Size: 24 225 280 bytes

Run Information
Type: Kinetics/Affinity
Cycles: 70
Start: 10/27/2023 1:54:59 PM
End: 10/29/2023 5:29:48 AM

Instrument
Instrument Type: BiacoreT200
Instrument Id: 1646191
IFC: TYPE105

User Information
Run Performed By: Administrator

Created With Software
Name: Biacore T200 Control Software
Version: 1.0

Chip Information
Chip Id: 231023 Vaidehi CM5
Chip Lot No: 10345041
Chip Name: CM5
First Dock Date: 10/23/2023 11:57:59 PM
Last Modification Date: 10/23/2023 11:57:59 PM
Last Use Date: 10/23/2023 11:59:23 PM

Immobilization in Fc=1
Immobilization Date:
Immobilization Result:
Ligand:
Final Response [RU]:

Immobilization in Fc=2
Immobilization Date:
Immobilization Result:
Ligand:
Final Response [RU]:

Immobilization in Fc=3
Immobilization Date:
Immobilization Result:
Ligand:
Final Response [RU]:

Immobilization in Fc=4
Immobilization Date:

File Properties (continued)

Immobilization Result:
Ligand:
Final Response [RU]:

Notebook

Kinetics: '131', fit: '1. 1:1 Binding'

Curve: Fc=2-1 Ligand: N/A Sample: 131 Temp: 25°C

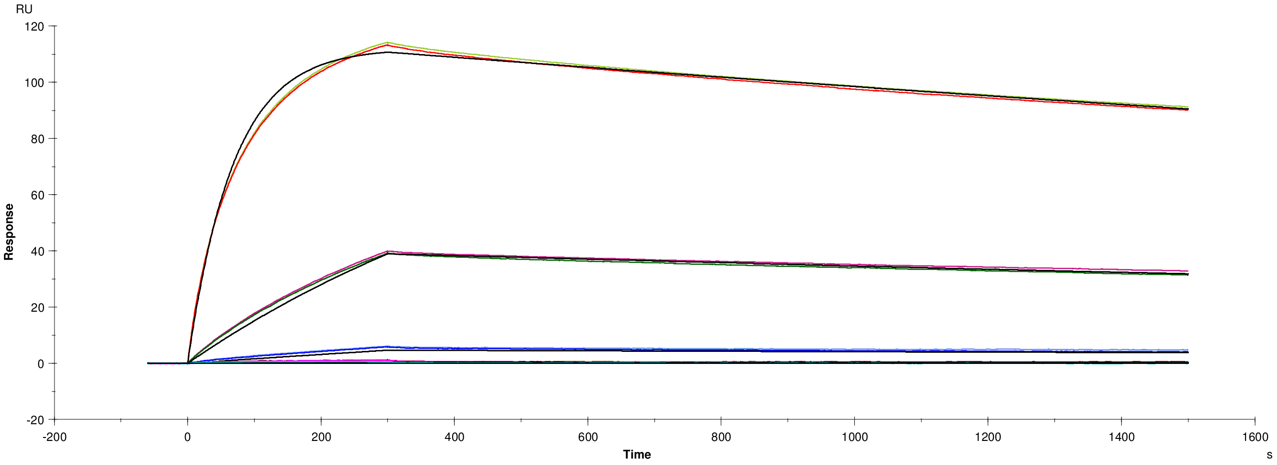

Residuals

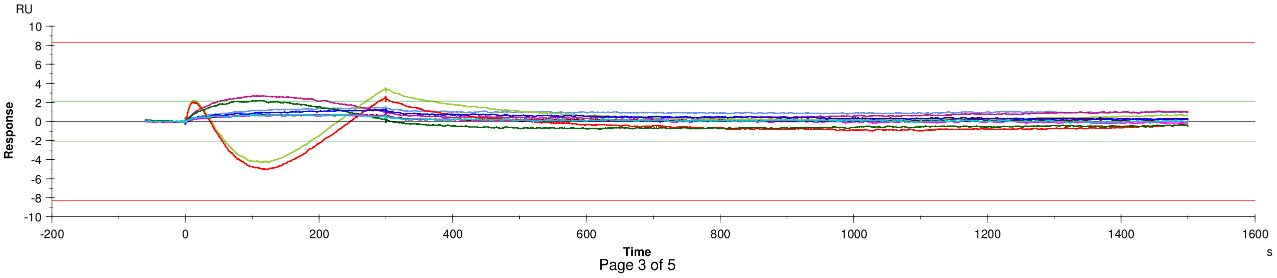

Kinetics: '131', fit: '1. 1:1 Binding' (continued)

Quality Control

✓	Kinetic constants are within instrument specifications.
✓	Kinetic constants appear to be uniquely determined.
⇨	Bulk contributions (RI) were not evaluated. The RI parameter is set to constant.
⇨	Check that sensorgrams have sufficient curvature.
⇨	Examine the residual plot. Pay attention to systematic and non-random deviations.

Kinetics: '131', fit: '1. 1:1 Binding' (continued)

Report table

Curve	ka (1/Ms)	kd (1/s)	KD (M)	Rmax (RU)	Conc (M)	tc	Flow (ul/min)	kt (RU/Ms)	RI (RU)	Chi² (RU²)	U-value
	1.445E+5	1.680E-4	1.162E-9	113.5		6.070E+18				0.779	2
Cycle: 17 0.01 nM					1.000E-11		30.00	1.886E+19	0.000		
Cycle: 18 0.1 nM					1.000E-10		30.00	1.886E+19	0.000		
Cycle: 19 1 nM					1.000E-9		30.00	1.886E+19	0.000		
Cycle: 20 10 nM					1.000E-8		30.00	1.886E+19	0.000		
Cycle: 21 100 nM					1.000E-7		30.00	1.886E+19	0.000		
Cycle: 22 100 nM					1.000E-7		30.00	1.886E+19	0.000		
Cycle: 23 10 nM					1.000E-8		30.00	1.886E+19	0.000		
Cycle: 24 1 nM					1.000E-9		30.00	1.886E+19	0.000		
Cycle: 25 0.1 nM					1.000E-10		30.00	1.886E+19	0.000		
Cycle: 26 0.01 nM					1.000E-11		30.00	1.886E+19	0.000		

Parameters table

Curve	ka (1/Ms)	SE(ka)	kd (1/s)	SE(kd)	Rmax (RU)	SE(Rmax)	Conc (M)	tc	SE(tc)	f (ul/min)	RI (RU)
	1.445E+5	30	1.680E-4	1.3E-7	113.5	0.011		6.070E+18	1.5E+21		
Cycle: 17 0.01 nM							1E-11			30	0.0
Cycle: 18 0.1 nM							1E-10			30	0.0
Cycle: 19 1 nM							1E-09			30	0.0
Cycle: 20 10 nM							1E-08			30	0.0
Cycle: 21 100 nM							1E-07			30	0.0
Cycle: 22 100 nM							1E-07			30	0.0
Cycle: 23 10 nM							1E-08			30	0.0
Cycle: 24 1 nM							1E-09			30	0.0
Cycle: 25 0.1 nM							1E-10			30	0.0
Cycle: 26 0.01 nM							1E-11			30	0.0

File Properties

Evaluation File
Name: N/A

User Information
Performed By: Administrator
Current User: Administrator

Created With Software
Name: Biacore T200 Evaluation Software
Version: 1.0

Notebook

Result

Result File
Name: 20231027-GFP-mutants-analyte-1-5_aGFP-S54C-RSGGH_FC2-1.blr
Path: C:\Bia Users\Vaidehi vr358
Size: 24 225 280 bytes

Run Information
Type: Kinetics/Affinity
Cycles: 70
Start: 10/27/2023 1:54:59 PM
End: 10/29/2023 5:29:48 AM

Instrument
Instrument Type: BiacoreT200
Instrument Id: 1646191
IFC: TYPE105

User Information
Run Performed By: Administrator

Created With Software
Name: Biacore T200 Control Software
Version: 1.0

Chip Information
Chip Id: 231023 Vaidehi CM5
Chip Lot No: 10345041
Chip Name: CM5
First Dock Date: 10/23/2023 11:57:59 PM
Last Modification Date: 10/23/2023 11:57:59 PM
Last Use Date: 10/23/2023 11:59:23 PM

Immobilization in Fc=1
Immobilization Date:
Immobilization Result:
Ligand:
Final Response [RU]:

Immobilization in Fc=2
Immobilization Date:
Immobilization Result:
Ligand:
Final Response [RU]:

Immobilization in Fc=3
Immobilization Date:
Immobilization Result:
Ligand:
Final Response [RU]:

Immobilization in Fc=4
Immobilization Date:

File Properties (continued)

Immobilization Result:
Ligand:
Final Response [RU]:

Notebook

Kinetics: '140', fit: '1. 1:1 Binding'

Curve: Fc=2-1 Ligand: N/A Sample: 140 Temp: 25°C

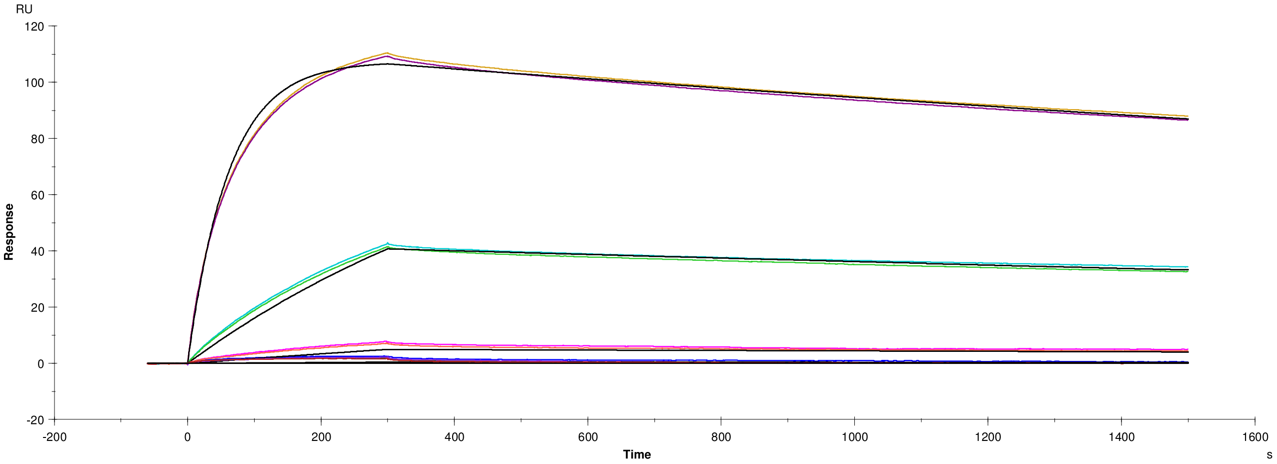

Residuals

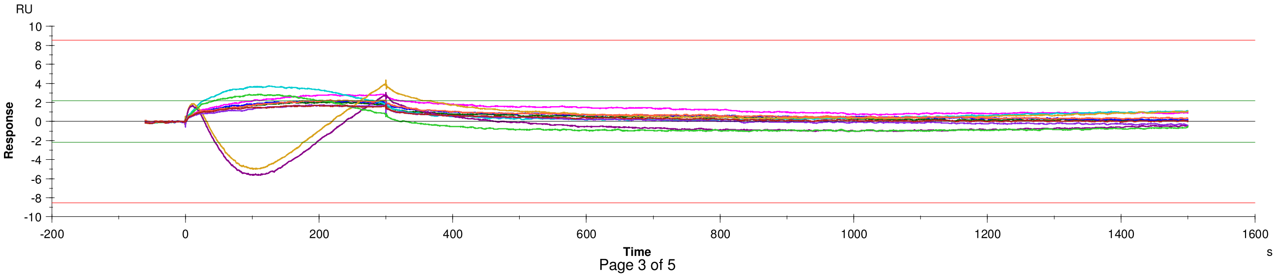

Kinetics: ‘140’, fit: ‘1. 1:1 Binding’ (continued)

Quality Control

✓	Kinetic constants are within instrument specifications.
✓	Kinetic constants appear to be uniquely determined.
⇨	Bulk contributions (RI) were not evaluated. The RI parameter is set to constant.
⇨	Check that sensorgrams have sufficient curvature.
⇨	Examine the residual plot. Pay attention to systematic and non-random deviations.

Kinetics: ‘140’, fit: ‘1. 1:1 Binding’ (continued)

Report table

Curve	ka (1/Ms)	kd (1/s)	KD (M)	Rmax (RU)	Conc (M)	tc	Flow (ul/min)	kt (RU/Ms)	RI (RU)	Chi² (RU²)	U-value
	1.619E+5	1.693E-4	1.046E-9	108.5		6.769E+18				1.47	2
Cycle: 59 0.01 nM					1.000E-11		30.00	2.103E+19	0.000		
Cycle: 60 0.1 nM					1.000E-10		30.00	2.103E+19	0.000		
Cycle: 61 1 nM					1.000E-9		30.00	2.103E+19	0.000		
Cycle: 62 10 nM					1.000E-8		30.00	2.103E+19	0.000		
Cycle: 63 100 nM					1.000E-7		30.00	2.103E+19	0.000		
Cycle: 64 100 nM					1.000E-7		30.00	2.103E+19	0.000		
Cycle: 65 10 nM					1.000E-8		30.00	2.103E+19	0.000		
Cycle: 66 1 nM					1.000E-9		30.00	2.103E+19	0.000		
Cycle: 67 0.1 nM					1.000E-10		30.00	2.103E+19	0.000		
Cycle: 68 0.01 nM					1.000E-11		30.00	2.103E+19	0.000		

Parameters table

Curve	ka (1/Ms)	SE(ka)	kd (1/s)	SE(kd)	Rmax (RU)	SE(Rmax)	Conc (M)	tc	SE(tc)	f (ul/min)	RI (RU)
	1.619E+5	45	1.693E-4	1.8E-7	108.5	0.014		6.769E+18	5.6E+21		
Cycle: 59 0.01 nM							1E-11			30	0.0
Cycle: 60 0.1 nM							1E-10			30	0.0
Cycle: 61 1 nM							1E-09			30	0.0
Cycle: 62 10 nM							1E-08			30	0.0
Cycle: 63 100 nM							1E-07			30	0.0
Cycle: 64 100 nM							1E-07			30	0.0
Cycle: 65 10 nM							1E-08			30	0.0
Cycle: 66 1 nM							1E-09			30	0.0
Cycle: 67 0.1 nM							1E-10			30	0.0
Cycle: 68 0.01 nM							1E-11			30	0.0

File Properties

Evaluation File
Name: N/A

User Information
Performed By: Administrator
Current User: Administrator

Created With Software
Name: Biacore T200 Evaluation Software
Version: 1.0

Notebook

Result

Result File
Name: 20231027-GFP-mutants-analyte-1-5_aGFP-S54C-RSGGH_FC2-1.blr
Path: C:\Bia Users\Vaidehi vr358
Size: 24 225 280 bytes

Run Information
Type: Kinetics/Affinity
Cycles: 70
Start: 10/27/2023 1:54:59 PM
End: 10/29/2023 5:29:48 AM

Instrument
Instrument Type: BiacoreT200
Instrument Id: 1646191
IFC: TYPE105

User Information
Run Performed By: Administrator

Created With Software
Name: Biacore T200 Control Software
Version: 1.0

Chip Information
Chip Id: 231023 Vaidehi CM5
Chip Lot No: 10345041
Chip Name: CM5
First Dock Date: 10/23/2023 11:57:59 PM
Last Modification Date: 10/23/2023 11:57:59 PM
Last Use Date: 10/23/2023 11:59:23 PM

Immobilization in Fc=1
Immobilization Date:
Immobilization Result:
Ligand:
Final Response [RU]:

Immobilization in Fc=2
Immobilization Date:
Immobilization Result:
Ligand:
Final Response [RU]:

Immobilization in Fc=3
Immobilization Date:
Immobilization Result:
Ligand:
Final Response [RU]:

Immobilization in Fc=4
Immobilization Date:

File Properties (continued)

Immobilization Result:
Ligand:
Final Response [RU]:

Notebook

Kinetics: '162', fit: '1. 1:1 Binding'

Curve: Fc=2-1 Ligand: N/A Sample: 162 Temp: 25°C

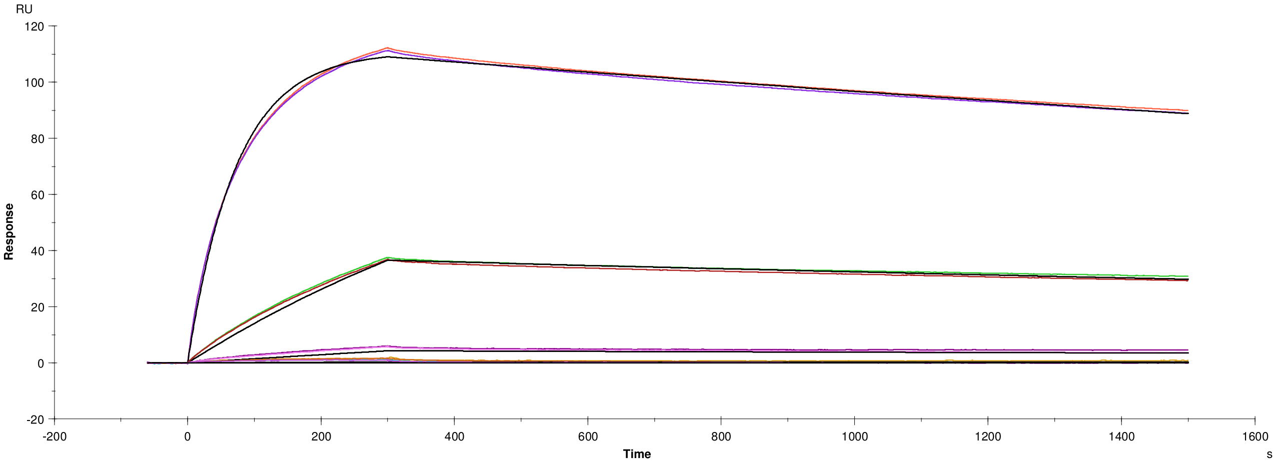

Residuals

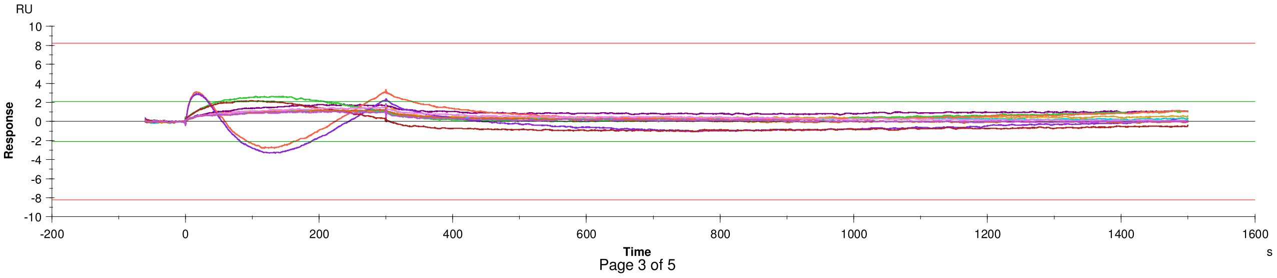

Kinetics: ‘162’, fit: ‘1. 1:1 Binding’ (continued)

Quality Control

✓	Kinetic constants are within instrument specifications.
✓	Kinetic constants appear to be uniquely determined.
⇨	Bulk contributions (RI) were not evaluated. The RI parameter is set to constant.
⇨	Check that sensorgrams have sufficient curvature.
⇨	Examine the residual plot. Pay attention to systematic and non-random deviations.

Kinetics: ‘162’, fit: ‘1. 1:1 Binding’ (continued)

Report table

Curve	ka (1/Ms)	kd (1/s)	KD (M)	Rmax (RU)	Conc (M)	tc	Flow (ul/min)	kt (RU/Ms)	RI (RU)	Chi² (RU²)	U-value
	1.354E+5	1.709E-4	1.263E-9	112.3		6.354E+14				0.705	2
Cycle: 31 0.01 nM					1.000E-11		30.00	1.974E+15	0.000		
Cycle: 32 0.1 nM					1.000E-10		30.00	1.974E+15	0.000		
Cycle: 33 1 nM					1.000E-9		30.00	1.974E+15	0.000		
Cycle: 34 10 nM					1.000E-8		30.00	1.974E+15	0.000		
Cycle: 35 100 nM					1.000E-7		30.00	1.974E+15	0.000		
Cycle: 36 100 nM					1.000E-7		30.00	1.974E+15	0.000		
Cycle: 37 10 nM					1.000E-8		30.00	1.974E+15	0.000		
Cycle: 38 1 nM					1.000E-9		30.00	1.974E+15	0.000		
Cycle: 39 0.1 nM					1.000E-10		30.00	1.974E+15	0.000		
Cycle: 40 0.01 nM					1.000E-11		30.00	1.974E+15	0.000		

Parameters table

Curve	ka (1/Ms)	SE(ka)	kd (1/s)	SE(kd)	Rmax (RU)	SE(Rmax)	Conc (M)	tc	SE(tc)	f (ul/min)	RI (RU)
	1.354E+5	28	1.709E-4	1.3E-7	112.3	0.010		6.354E+14	1.4E+13		
Cycle: 31 0.01 nM							1E-11			30	0.0
Cycle: 32 0.1 nM							1E-10			30	0.0
Cycle: 33 1 nM							1E-09			30	0.0
Cycle: 34 10 nM							1E-08			30	0.0
Cycle: 35 100 nM							1E-07			30	0.0
Cycle: 36 100 nM							1E-07			30	0.0
Cycle: 37 10 nM							1E-08			30	0.0
Cycle: 38 1 nM							1E-09			30	0.0
Cycle: 39 0.1 nM							1E-10			30	0.0
Cycle: 40 0.01 nM							1E-11			30	0.0

File Properties

Evaluation File
Name: N/A

User Information
Performed By: Administrator
Current User: Administrator

Created With Software
Name: Biacore T200 Evaluation Software
Version: 1.0

Notebook

Result

Result File
Name: 20231027-GFP-mutants-analyte-1-5_aGFP-S54C-RSGGH_FC2-1.blr
Path: C:\Bia Users\Vaidehi vr358
Size: 24 225 280 bytes

Run Information
Type: Kinetics/Affinity
Cycles: 70
Start: 10/27/2023 1:54:59 PM
End: 10/29/2023 5:29:48 AM

Instrument
Instrument Type: BiacoreT200
Instrument Id: 1646191
IFC: TYPE105

User Information
Run Performed By: Administrator

Created With Software
Name: Biacore T200 Control Software
Version: 1.0

Chip Information
Chip Id: 231023 Vaidehi CM5
Chip Lot No: 10345041
Chip Name: CM5
First Dock Date: 10/23/2023 11:57:59 PM
Last Modification Date: 10/23/2023 11:57:59 PM
Last Use Date: 10/23/2023 11:59:23 PM

Immobilization in Fc=1
Immobilization Date:
Immobilization Result:
Ligand:
Final Response [RU]:

Immobilization in Fc=2
Immobilization Date:
Immobilization Result:
Ligand:
Final Response [RU]:

Immobilization in Fc=3
Immobilization Date:
Immobilization Result:
Ligand:
Final Response [RU]:

Immobilization in Fc=4
Immobilization Date:

File Properties (continued)

Immobilization Result:
Ligand:
Final Response [RU]:

Notebook

Unsaved file

Instr. id: 1646191 Biacore T200 Evaluation Software, version 1.0 Print date: 10/30/2023 1:52:32 AM

Kinetics: '166', fit: '1. 1:1 Binding'

Curve: Fc=2-1 Ligand: N/A Sample: 166 Temp: 25°C

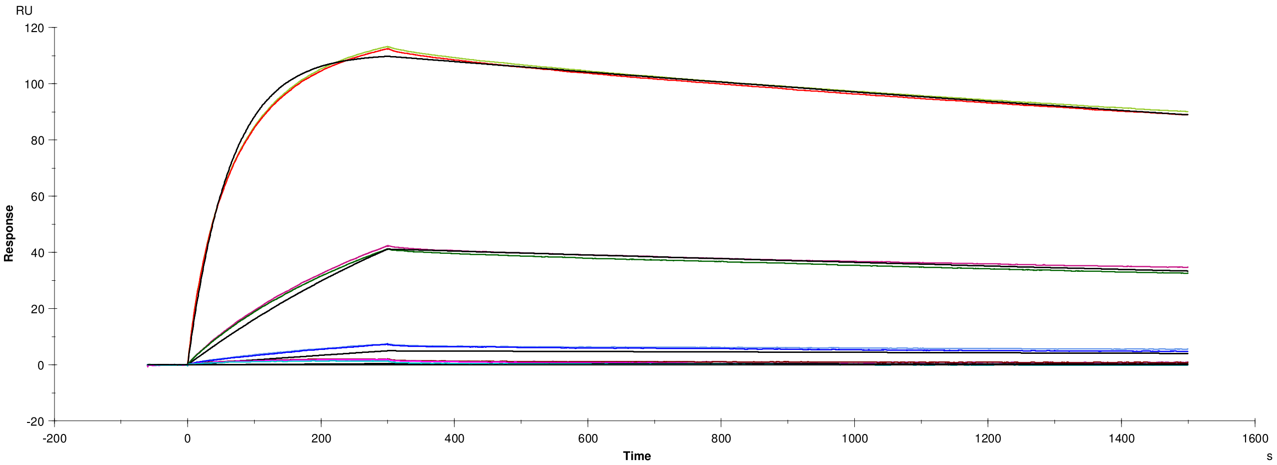

Residuals

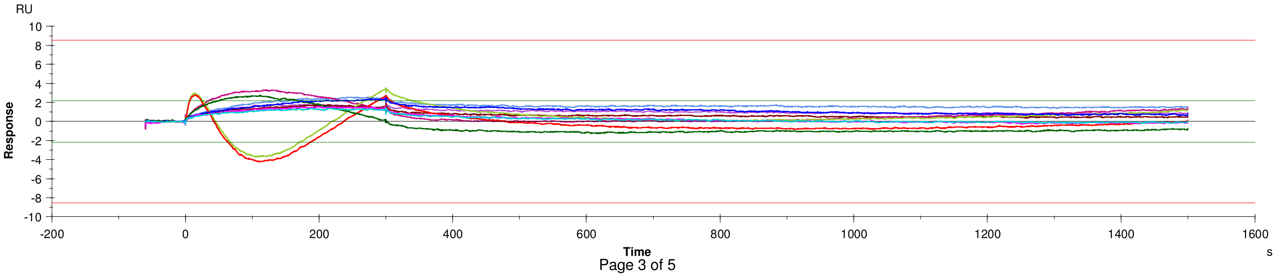

Kinetics: ‘166’, fit: ‘1. 1:1 Binding’ (continued)

Quality Control

✓	Kinetic constants are within instrument specifications.
✓	Kinetic constants appear to be uniquely determined.
⇨	Bulk contributions (RI) were not evaluated. The RI parameter is set to constant.
⇨	Check that sensorgrams have sufficient curvature.
⇨	Examine the residual plot. Pay attention to systematic and non-random deviations.

Kinetics: ‘166’, fit: ‘1. 1:1 Binding’ (continued)

Report table

Curve	ka (1/Ms)	kd (1/s)	KD (M)	Rmax (RU)	Conc (M)	tc	Flow (ul/min)	kt (RU/Ms)	RI (RU)	Chi² (RU²)	U-value
	1.579E+5	1.753E-4	1.110E-9	112.0		3.735E+18				1.32	2
Cycle: 45 0.01 nM					1.000E-11		30.00	1.161E+19	0.000		
Cycle: 46 0.1 nM					1.000E-10		30.00	1.161E+19	0.000		
Cycle: 47 1 nM					1.000E-9		30.00	1.161E+19	0.000		
Cycle: 48 10 nM					1.000E-8		30.00	1.161E+19	0.000		
Cycle: 49 100 nM					1.000E-7		30.00	1.161E+19	0.000		
Cycle: 50 100 nM					1.000E-7		30.00	1.161E+19	0.000		
Cycle: 51 10 nM					1.000E-8		30.00	1.161E+19	0.000		
Cycle: 52 1 nM					1.000E-9		30.00	1.161E+19	0.000		
Cycle: 53 0.1 nM					1.000E-10		30.00	1.161E+19	0.000		
Cycle: 54 0.01 nM					1.000E-11		30.00	1.161E+19	0.000		

Parameters table

Curve	ka (1/Ms)	SE(ka)	kd (1/s)	SE(kd)	Rmax (RU)	SE(Rmax)	Conc (M)	tc	SE(tc)	f (ul/min)	RI (RU)
	1.579E+5	41	1.753E-4	1.7E-7	112.0	0.013		3.735E+18	3.4E+21		
Cycle: 45 0.01 nM							1E-11			30	0.0
Cycle: 46 0.1 nM							1E-10			30	0.0
Cycle: 47 1 nM							1E-09			30	0.0
Cycle: 48 10 nM							1E-08			30	0.0
Cycle: 49 100 nM							1E-07			30	0.0
Cycle: 50 100 nM							1E-07			30	0.0
Cycle: 51 10 nM							1E-08			30	0.0
Cycle: 52 1 nM							1E-09			30	0.0
Cycle: 53 0.1 nM							1E-10			30	0.0
Cycle: 54 0.01 nM							1E-11			30	0.0

Image Report: admin 2023-03-11 22h39m23s analysed

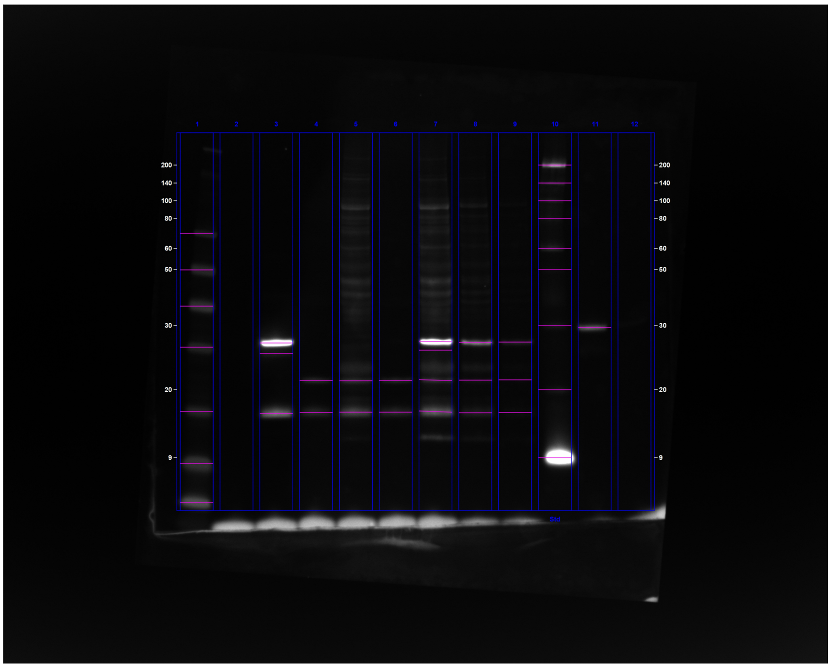

C:\Users\oded\OneDrive - University Of Cambridge\Vendruscolo\Results\Western\ChemiDoc Images 2023-03-11_22.57.16\admin 2023-03-11 22h39m23s analysed.scn

---

Acquisition Information

Imager	ChemiDoc™ MP
Exposure Time (sec)	0.050 (Manual)
Flat Field	Applied (Red Epi)
Serial Number	734BR-3787
Software Version	2.4.0.03
Application	Alexa 647
Excitation Source	Red Epi Illumination
Emission Filter	700/50 Filter

Image Information

Acquisition Date	11/03/2023 10:39:23 PM
User Name	admin
Image Area (mm)	X: 129.0 Y: 103.2
Pixel Size (µm)	X: 46.9 Y: 46.9
Data Range (Int)	370 - 20506

Analysis Settings

Detection	Lane detection:<br>Manually created lanes

	Band detection:<br>Automatically detected bands with custom sensitivity: 70<br>Manually adjusted bands<br><br>Lane Background Subtraction:<br>Lane background subtracted with disk size: 17<br><br>Lane width: 5.16 mm
Mol. Weight Analysis	Standard: BiotinMarker 7227<br>Standard lanes: 10<br>Regression method: Point to Point (semi-log)

Lane Statistics

Lane No.	Adj. Total Band Vol. (Int)	Total Band Vol. (Int)	Adj. Total Lane Vol. (Int)	Total Lane Vol. (Int)	Bkgd. Vol. (Int)	Norm. Factor
1	17,781,390	38,119,070	20,854,790	97,962,920	77,108,130	N/A
2	N/A	N/A	898,810	70,482,280	69,583,470	N/A
3	31,331,300	38,048,010	35,240,260	107,671,410	72,431,150	N/A
4	2,727,890	6,681,070	5,022,050	83,554,460	78,532,410	N/A
5	4,674,230	9,063,890	16,165,710	113,572,250	97,406,540	N/A
6	2,429,900	5,564,020	4,737,040	86,070,710	81,333,670	N/A
7	24,322,100	35,600,840	38,091,790	138,959,700	100,867,910	N/A
8	5,952,210	13,581,590	10,631,610	96,866,990	86,235,380	N/A
9	1,455,080	8,770,850	3,196,380	83,310,810	80,114,430	N/A
10	53,907,040	73,667,660	58,727,790	144,760,550	86,032,760	N/A
11	3,065,480	5,109,610	4,723,730	82,499,230	77,775,500	N/A
12	N/A	N/A	1,604,020	76,785,500	75,181,480	N/A

Lane And Band Analysis

Lane 1

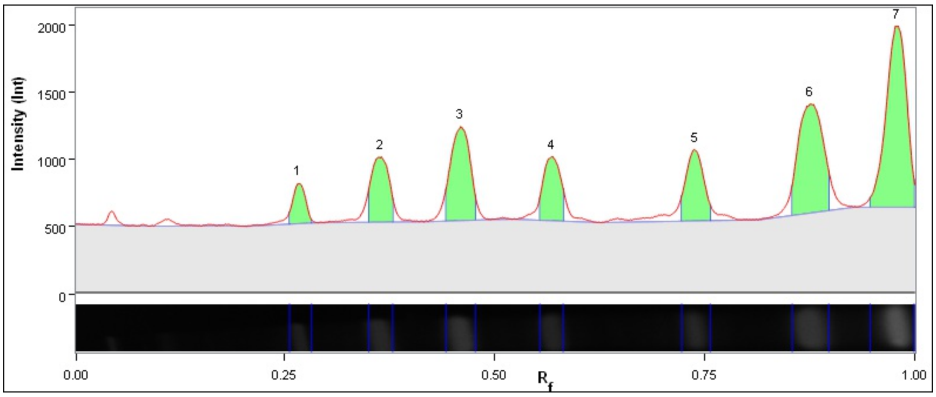

Band No.	Band Label	Mol. Wt. (KDa)	Relative Front	Adj. Volume (Int)	Volume (Int)	Abs. Quant.	Rel. Quant.	Band %	Lane %
1		69.3	0.266	734,690	2,727,120	N/A	N/A	4.1	3.5
2		49.9	0.363	1,654,620	3,865,290	N/A	N/A	9.3	7.9
3		35.8	0.459	2,596,330	5,284,730	N/A	N/A	14.6	12.4
4		26.2	0.568	1,555,840	3,751,660	N/A	N/A	8.7	7.5
5		15.5	0.738	1,816,760	4,493,500	N/A	N/A	10.2	8.7
6		9.0	0.875	3,894,440	7,611,230	N/A	N/A	21.9	18.7
7		9.0	0.979	5,528,710	10,385,540	N/A	N/A	31.1	26.5

Band Detection	Automatically detected bands with custom sensitivity: 70
Lane Background	Lane background subtracted with disk size: 17
Lane Width	5.16 mm
Regression Equation	A single equation is not available for this method

Lane 2

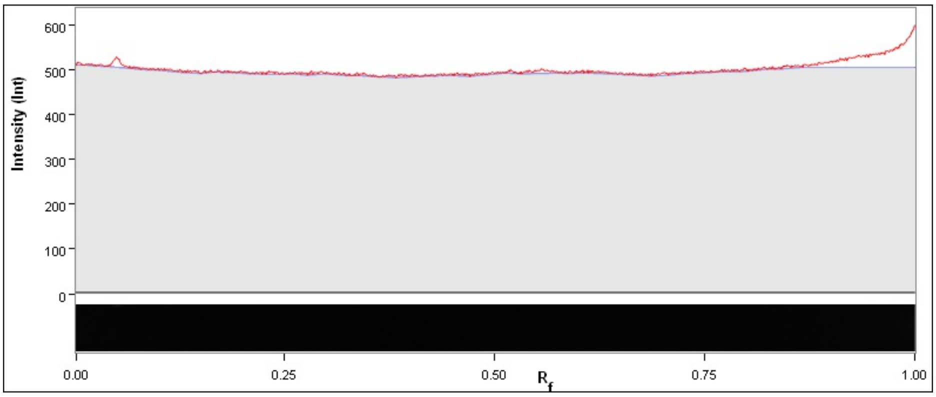

Band No.	Band Label	Mol. Wt. (KDa)	Relative Front	Adj. Volume (Int)	Volume (Int)	Abs. Quant.	Rel. Quant.	Band %	Lane %

Band Detection	Automatically detected bands with custom sensitivity: 70
Lane Background	Lane background subtracted with disk size: 17
Lane Width	5.16 mm
Regression Equation	A single equation is not available for this method

Lane 3

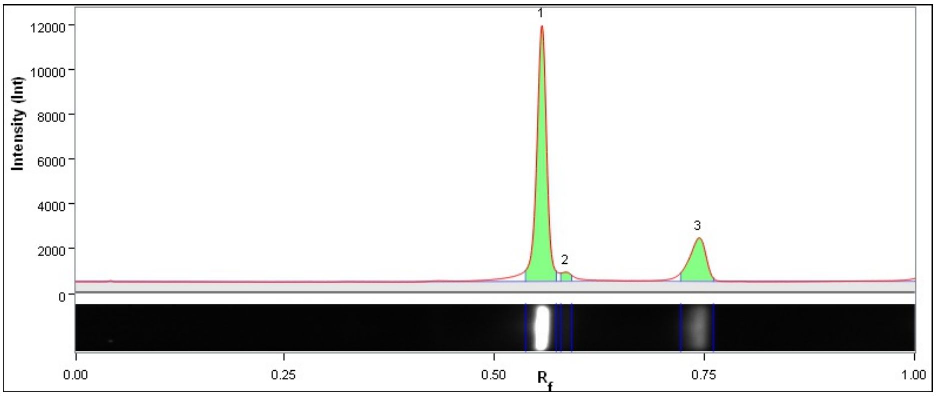

Band No.	Band Label	Mol. Wt. (KDa)	Relative Front	Adj. Volume (Int)	Volume (Int)	Abs. Quant.	Rel. Quant.	Band %	Lane %
1		26.9	0.557	23,927,750	26,688,750	N/A	N/A	76.4	67.9
2		25.2	0.584	743,050	1,752,520	N/A	N/A	2.4	2.1
3		15.1	0.743	6,660,500	9,606,740	N/A	N/A	21.3	18.9

Band Detection	Automatically detected bands with custom sensitivity: 70

Lane Background	Lane background subtracted with disk size: 17
Lane Width	5.16 mm
Regression Equation	A single equation is not available for this method

Lane 4

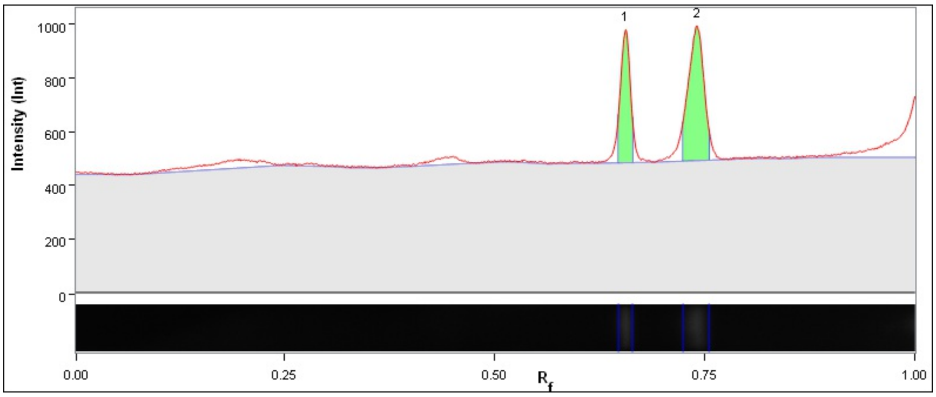

Band No.	Band Label	Mol. Wt. (KDa)	Relative Front	Adj. Volume (Int)	Volume (Int)	Abs. Quant.	Rel. Quant.	Band %	Lane %
1		21.2	0.656	1,015,190	2,403,500	N/A	N/A	37.2	20.2
2		15.3	0.741	1,712,700	4,277,570	N/A	N/A	62.8	34.1

Band Detection	Automatically detected bands with custom sensitivity: 70
Lane Background	Lane background subtracted with disk size: 17
Lane Width	5.16 mm
Regression Equation	A single equation is not available for this method

Lane 5

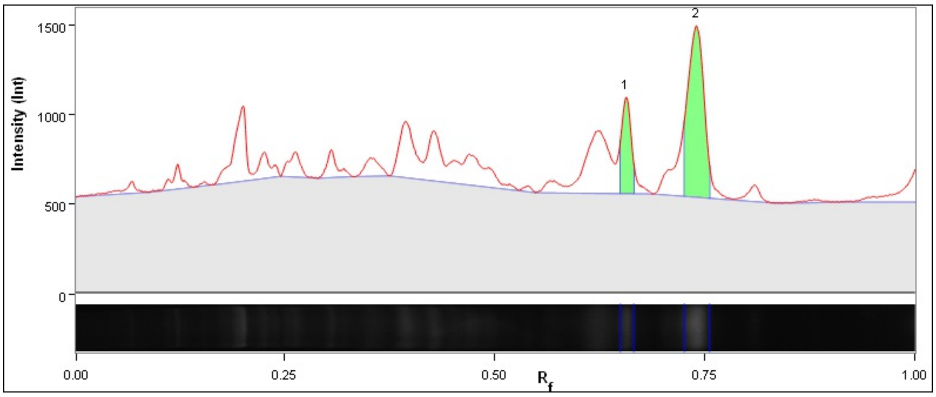

Band No.	Band Label	Mol. Wt. (KDa)	Relative Front	Adj. Volume (Int)	Volume (Int)	Abs. Quant.	Rel. Quant.	Band %	Lane %
1		21.2	0.657	1,147,960	2,721,180	N/A	N/A	24.6	7.1
2		15.4	0.740	3,526,270	6,342,710	N/A	N/A	75.4	21.8

Band Detection	Automatically detected bands with custom sensitivity: 70
Lane Background	Lane background subtracted with disk size: 17

Lane Width	5.16 mm
Regression Equation	A single equation is not available for this method

Lane 6

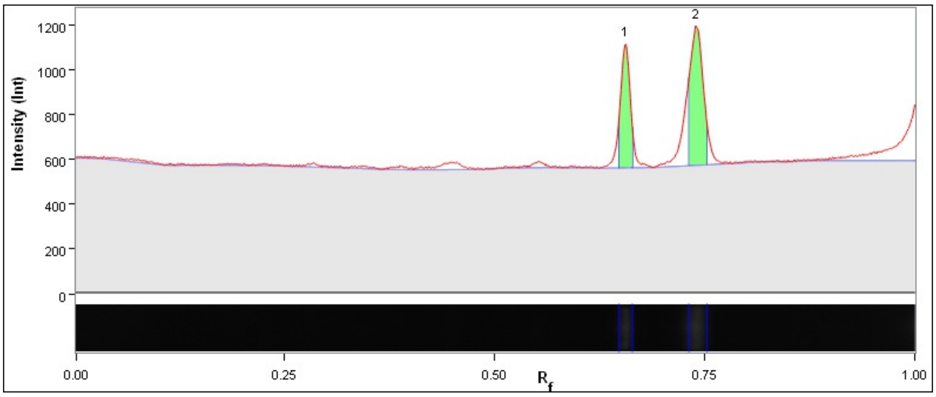

Band No.	Band Label	Mol. Wt. (KDa)	Relative Front	Adj. Volume (Int)	Volume (Int)	Abs. Quant.	Rel. Quant.	Band %	Lane %
1		21.2	0.656	950,290	2,277,330	N/A	N/A	39.1	20.1
2		15.4	0.740	1,479,610	3,286,690	N/A	N/A	60.9	31.2

Band Detection	Automatically detected bands with custom sensitivity: 70
Lane Background	Lane background subtracted with disk size: 17
Lane Width	5.16 mm
Regression Equation	A single equation is not available for this method

Lane 7

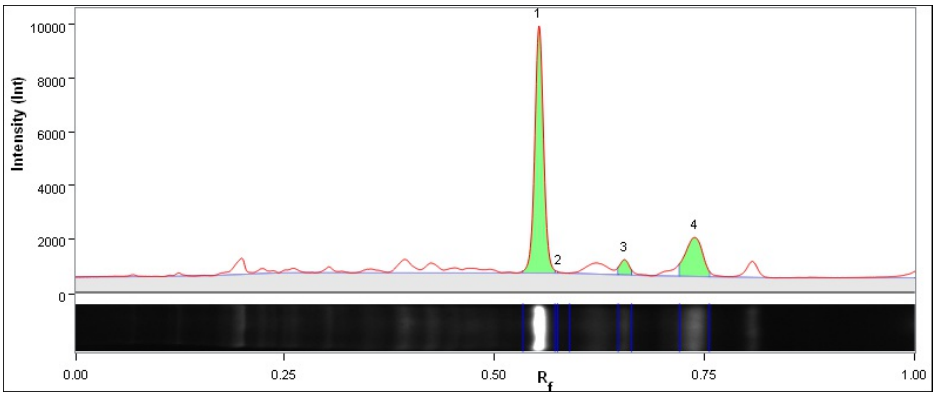

Band No.	Band Label	Mol. Wt. (KDa)	Relative Front	Adj. Volume (Int)	Volume (Int)	Abs. Quant.	Rel. Quant.	Band %	Lane %
1		27.1	0.553	17,788,870	22,277,970	N/A	N/A	73.1	46.7
2		25.7	0.576	32,890	1,729,970	N/A	N/A	0.1	0.1
3		21.3	0.655	1,105,170	2,803,350	N/A	N/A	4.5	2.9
4		15.5	0.738	5,395,170	8,789,550	N/A	N/A	22.2	14.2

Band Detection	Automatically detected bands with custom sensitivity: 70

Lane Background	Lane background subtracted with disk size: 17
Lane Width	5.16 mm
Regression Equation	A single equation is not available for this method

Lane 8

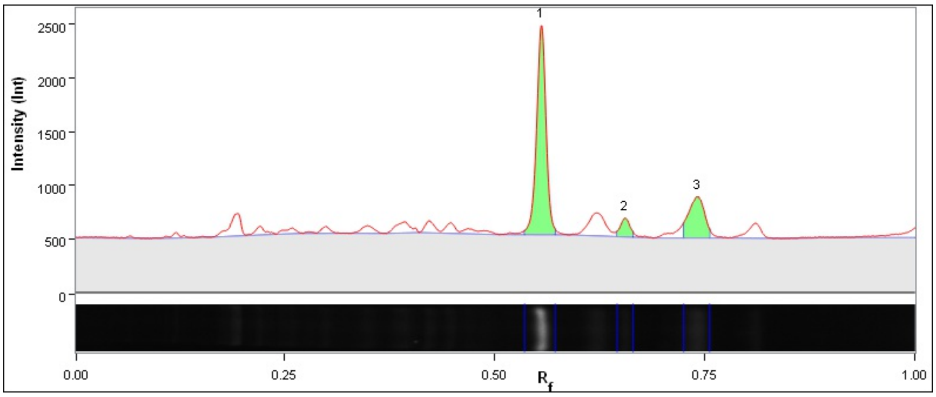

Band No.	Band Label	Mol. Wt. (KDa)	Relative Front	Adj. Volume (Int)	Volume (Int)	Abs. Quant.	Rel. Quant.	Band %	Lane %
1		27.0	0.555	4,221,360	7,516,190	N/A	N/A	70.9	39.7
2		21.3	0.655	372,130	2,057,880	N/A	N/A	6.3	3.5
3		15.3	0.741	1,358,720	4,007,520	N/A	N/A	22.8	12.8

Band Detection	Automatically detected bands with custom sensitivity: 70
Lane Background	Lane background subtracted with disk size: 17
Lane Width	5.16 mm
Regression Equation	A single equation is not available for this method

Lane 9

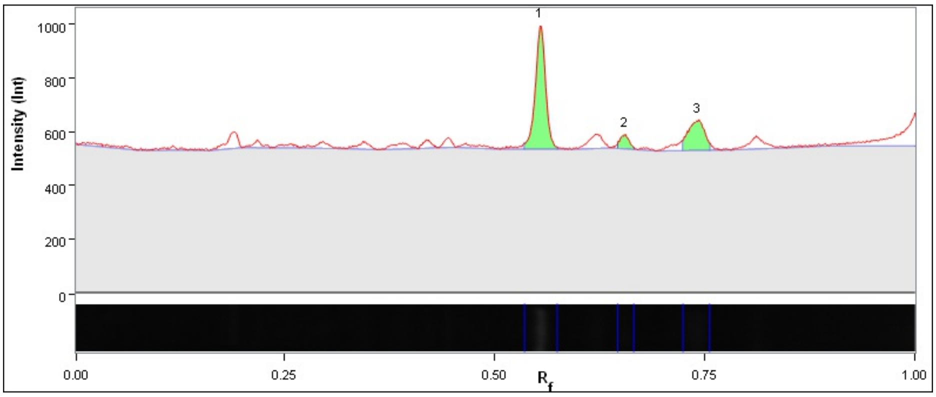

Band No.	Band Label	Mol. Wt. (KDa)	Relative Front	Adj. Volume (Int)	Volume (Int)	Abs. Quant.	Rel. Quant.	Band %	Lane %
1		27.0	0.554	978,780	4,141,280	N/A	N/A	67.3	30.6
2		21.3	0.654	97,790	1,681,240	N/A	N/A	6.7	3.1
3		15.3	0.741	378,510	2,948,330	N/A	N/A	26.0	11.8

Band Detection	Automatically detected bands with custom sensitivity: 70
Lane Background	Lane background subtracted with disk size: 17
Lane Width	5.16 mm
Regression Equation	A single equation is not available for this method

Lane 10 - BiotinMarker 7227

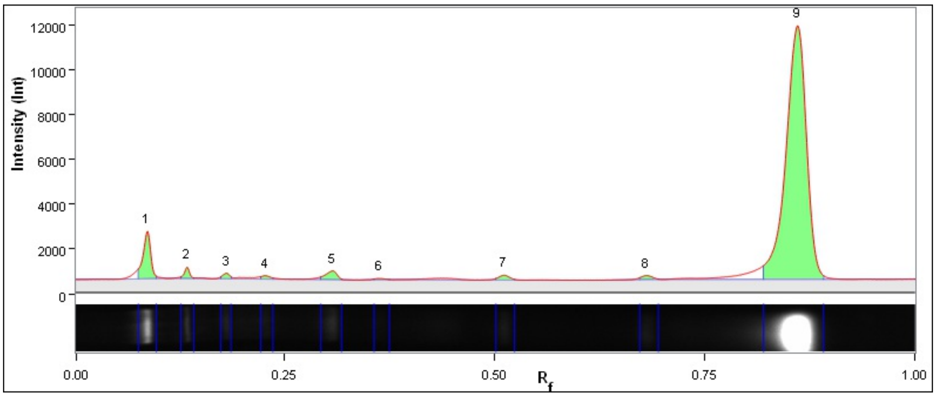

Band No.	Band Label	Mol. Wt. (KDa)	Relative Front	Adj. Volume (Int)	Volume (Int)	Abs. Quant.	Rel. Quant.	Band %	Lane %
1		200.0	0.086	3,260,510	5,206,850	N/A	N/A	6.0	5.6
2		140.0	0.133	537,790	1,998,150	N/A	N/A	1.0	0.9
3		100.0	0.180	336,600	1,483,900	N/A	N/A	0.6	0.6
4		80.0	0.227	253,440	1,589,720	N/A	N/A	0.5	0.4
5		60.0	0.306	893,750	3,040,180	N/A	N/A	1.7	1.5
6		50.0	0.362	120,670	1,717,540	N/A	N/A	0.2	0.2
7		30.0	0.511	464,420	2,353,670	N/A	N/A	0.9	0.8
8		20.0	0.680	399,080	2,360,930	N/A	N/A	0.7	0.7
9		9.0	0.860	47,640,780	53,916,720	N/A	N/A	88.4	81.1

Band Detection	Automatically detected bands with custom sensitivity: 70
Lane Background	Lane background subtracted with disk size: 17
Lane Width	5.16 mm
Regression Equation	A single equation is not available for this method

Lane 11

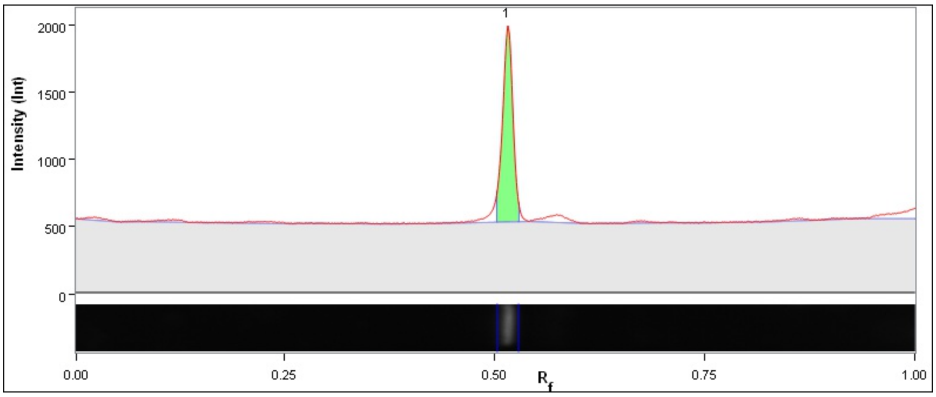

7

Band No.	Band Label	Mol. Wt. (KDa)	Relative Front	Adj. Volume (Int)	Volume (Int)	Abs. Quant.	Rel. Quant.	Band %	Lane %
1		29.7	0.515	3,065,480	5,109,610	N/A	N/A	100.0	64.9

Band Detection	Automatically detected bands with custom sensitivity: 70
Lane Background	Lane background subtracted with disk size: 17
Lane Width	5.16 mm
Regression Equation	A single equation is not available for this method

Lane 12

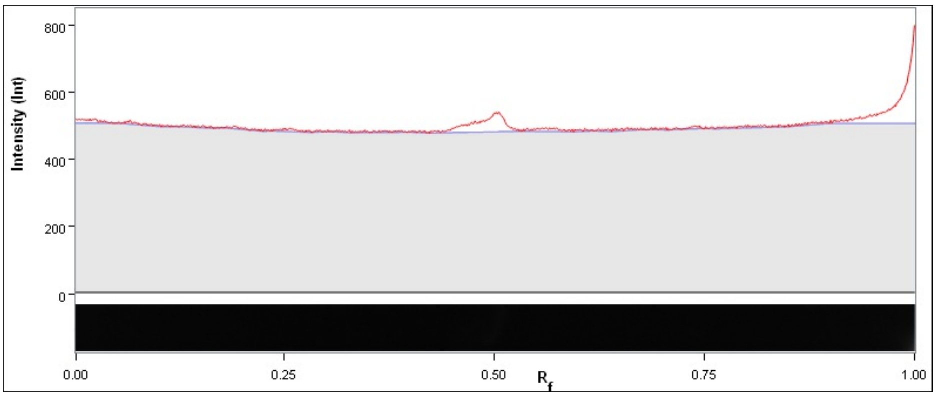

Band No.	Band Label	Mol. Wt. (KDa)	Relative Front	Adj. Volume (Int)	Volume (Int)	Abs. Quant.	Rel. Quant.	Band %	Lane %

Band Detection	Automatically detected bands with custom sensitivity: 70
Lane Background	Lane background subtracted with disk size: 17
Lane Width	5.16 mm
Regression Equation	A single equation is not available for this method

Image Report: admin 2023-03-25 20h41m30s

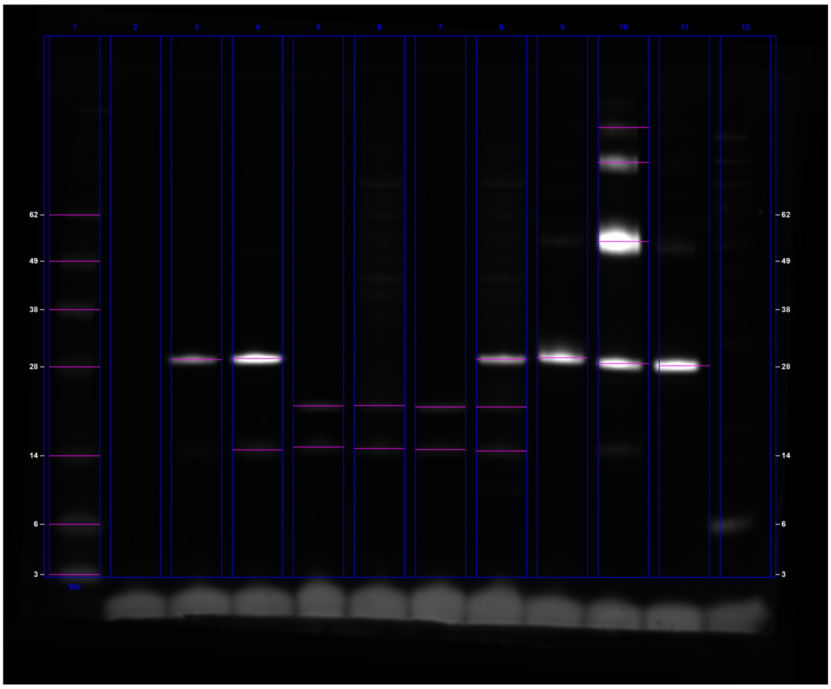

C:\Users\oded\OneDrive - University Of Cambridge\Vendruscolo\tmp\ChemiDoc Images 2023-03-25_20.48.53\admin 2023-03-25 20h41m30s.scn

---

Acquisition Information

Imager	ChemiDoc™ MP
Exposure Time (sec)	0.050 (Manual)
Flat Field	Applied (Red Epi)
Serial Number	734BR-3787
Software Version	2.4.0.03
Application	Alexa 647
Excitation Source	Red Epi Illumination
Emission Filter	700/50 Filter

Image Information

Acquisition Date	25/03/2023 8:41:30 PM
User Name	admin
Image Area (mm)	X: 89.3 Y: 73.7
Pixel Size (µm)	X: 39.5 Y: 39.5
Data Range (Int)	0 - 19859

Analysis Settings

Detection	Lane detection:<br>Manually created lanes

	Band detection:<br>Automatically detected bands with sensitivity: High<br>Manually adjusted bands<br><br>Lane Background Subtraction:<br>Lane background subtracted with disk size: 17<br><br>Lane width: 5.45 mm
Mol. Weight Analysis	Standard: SeeBlue2<br>Standard lanes: first<br>Regression method: Point to Point (semi-log)

Lane Statistics

Lane No.	Adj. Total Band Vol. (Int)	Total Band Vol. (Int)	Adj. Total Lane Vol. (Int)	Total Lane Vol. (Int)	Bkgd. Vol. (Int)	Norm. Factor
1	19,744,764	43,434,258	27,616,008	152,021,214	124,405,206	N/A
2	N/A	N/A	1,952,562	112,140,042	110,187,480	N/A
3	14,245,326	17,762,532	18,093,318	128,832,660	110,739,342	N/A
4	45,786,054	53,543,310	50,559,198	162,096,870	111,537,672	N/A
5	5,376,342	11,211,948	9,178,380	121,011,096	111,832,716	N/A
6	4,415,034	10,828,032	14,767,242	142,598,436	127,831,194	N/A
7	5,761,776	11,477,874	9,198,942	122,714,706	113,515,764	N/A
8	24,762,030	36,460,290	34,523,322	167,168,094	132,644,772	N/A
9	40,702,686	46,981,686	47,575,500	165,631,878	118,056,378	N/A
10	159,679,800	190,170,762	168,640,968	294,771,174	126,130,206	N/A
11	42,911,238	48,692,058	58,633,854	167,621,424	108,987,570	N/A
12	N/A	N/A	16,952,196	128,108,850	111,156,654	N/A

Lane And Band Analysis

Lane 1 - SeeBlue2

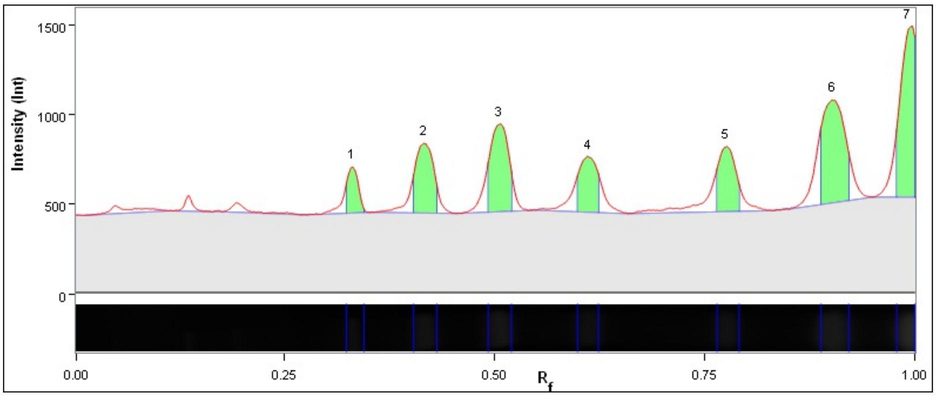

Band No.	Band Label	Mol. Wt. (KDa)	Relative Front	Adj. Volume (Int)	Volume (Int)	Abs. Quant.	Rel. Quant.	Band %	Lane %
1		62.0	0.330	1,002,570	3,613,254	N/A	N/A	5.1	3.6
2		49.0	0.416	2,444,118	5,862,792	N/A	N/A	12.4	8.9
3		38.0	0.505	3,109,002	6,587,844	N/A	N/A	15.7	11.3
4		28.0	0.611	1,912,818	5,038,656	N/A	N/A	9.7	6.9
5		14.0	0.775	2,165,082	5,482,326	N/A	N/A	11.0	7.8
6		6.0	0.902	4,476,582	9,088,266	N/A	N/A	22.7	16.2
7		3.0	0.995	4,634,592	7,761,120	N/A	N/A	23.5	16.8

Band Detection	Automatically detected bands with sensitivity: High
Lane Background	Lane background subtracted with disk size: 17
Lane Width	5.45 mm
Regression Equation	A single equation is not available for this method

Lane 2

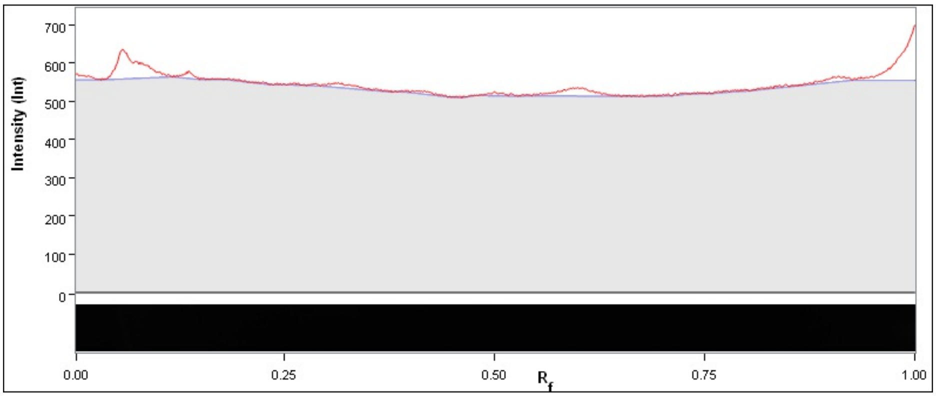

Band No.	Band Label	Mol. Wt. (KDa)	Relative Front	Adj. Volume (Int)	Volume (Int)	Abs. Quant.	Rel. Quant.	Band %	Lane %

Band Detection	Automatically detected bands with sensitivity: High
Lane Background	Lane background subtracted with disk size: 17
Lane Width	5.45 mm
Regression Equation	A single equation is not available for this method

Lane 3

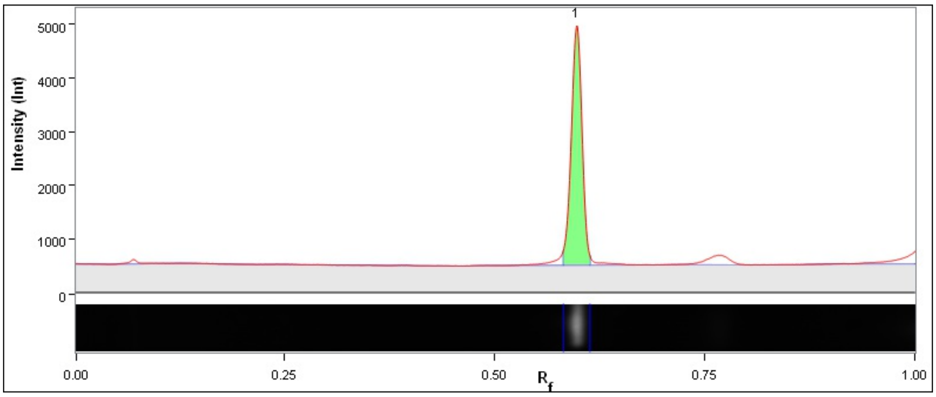

Band No.	Band Label	Mol. Wt. (KDa)	Relative Front	Adj. Volume (Int)	Volume (Int)	Abs. Quant.	Rel. Quant.	Band %	Lane %
1		29.1	0.598	14,245,326	17,762,532	N/A	N/A	100.0	78.7

Band Detection	Automatically detected bands with sensitivity: High
Lane Background	Lane background subtracted with disk size: 17
Lane Width	5.45 mm

Regression Equation	A single equation is not available for this method

Lane 4

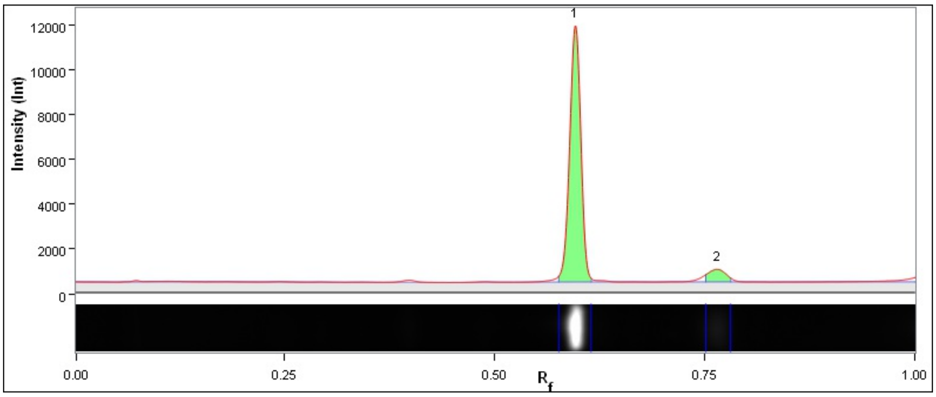

Band No.	Band Label	Mol. Wt. (KDa)	Relative Front	Adj. Volume (Int)	Volume (Int)	Abs. Quant.	Rel. Quant.	Band %	Lane %
1		29.3	0.596	42,628,062	46,973,268	N/A	N/A	93.1	84.3
2		14.7	0.764	3,157,992	6,570,042	N/A	N/A	6.9	6.2

Band Detection	Automatically detected bands with sensitivity: High
Lane Background	Lane background subtracted with disk size: 17
Lane Width	5.45 mm
Regression Equation	A single equation is not available for this method

Lane 5

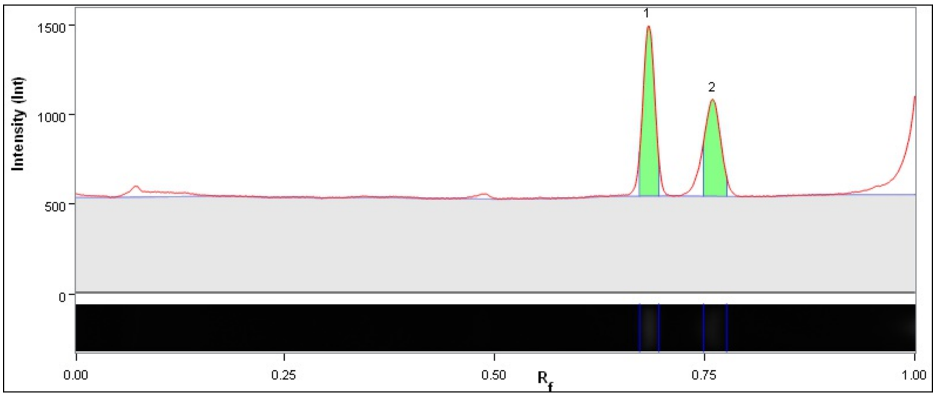

Band No.	Band Label	Mol. Wt. (KDa)	Relative Front	Adj. Volume (Int)	Volume (Int)	Abs. Quant.	Rel. Quant.	Band %	Lane %
1		20.7	0.683	3,127,218	5,783,442	N/A	N/A	58.2	34.1
2		15.0	0.759	2,249,124	5,428,506	N/A	N/A	41.8	24.5

Band Detection	Automatically detected bands with sensitivity: High
Lane Background	Lane background subtracted with disk size: 17
Lane Width	5.45 mm
Regression Equation	A single equation is not available for this method

Lane 6

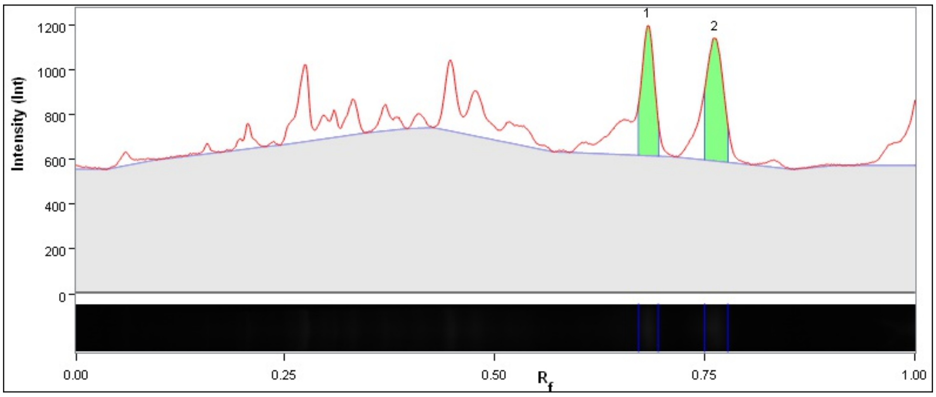

Band No.	Band Label	Mol. Wt. (KDa)	Relative Front	Adj. Volume (Int)	Volume (Int)	Abs. Quant.	Rel. Quant.	Band %	Lane %
1		20.7	0.682	2,008,038	5,032,998	N/A	N/A	45.5	13.6
2		14.8	0.762	2,406,996	5,795,034	N/A	N/A	54.5	16.3

Band Detection	Automatically detected bands with sensitivity: High
Lane Background	Lane background subtracted with disk size: 17
Lane Width	5.45 mm
Regression Equation	A single equation is not available for this method

Lane 7

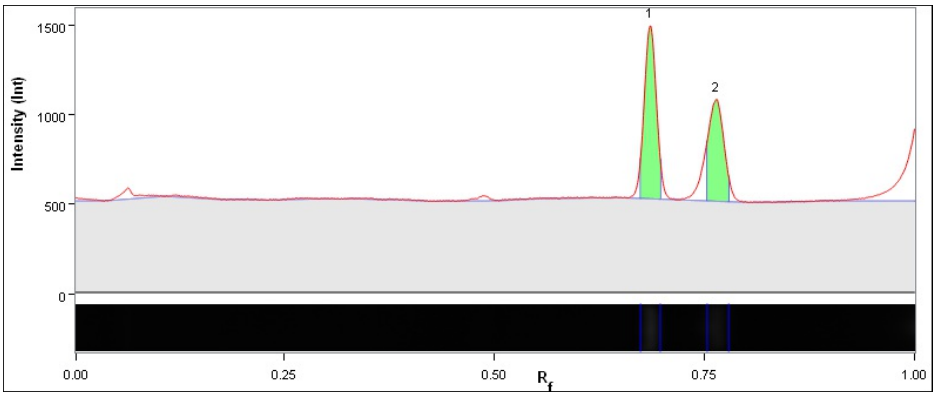

Band No.	Band Label	Mol. Wt. (KDa)	Relative Front	Adj. Volume (Int)	Volume (Int)	Abs. Quant.	Rel. Quant.	Band %	Lane %
1		20.5	0.685	3,370,650	6,153,834	N/A	N/A	58.5	36.6
2		14.7	0.764	2,391,126	5,324,040	N/A	N/A	41.5	26.0

Band Detection	Automatically detected bands with sensitivity: High
Lane Background	Lane background subtracted with disk size: 17
Lane Width	5.45 mm
Regression Equation	A single equation is not available for this method

Lane 8

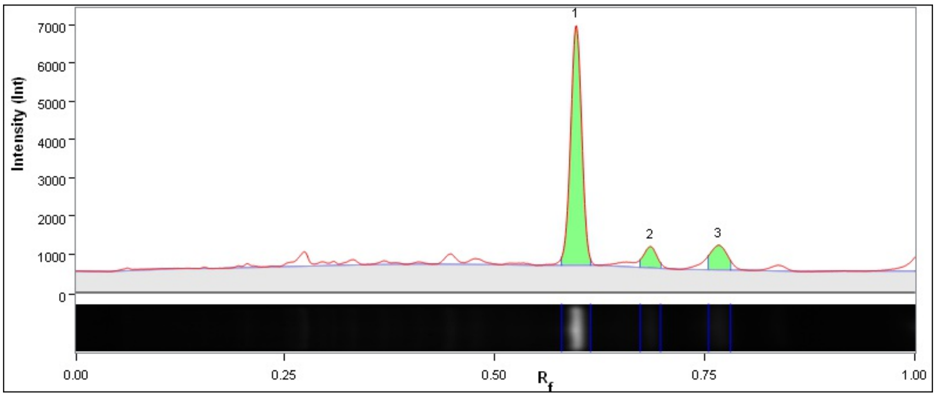

Band No.	Band Label	Mol. Wt. (KDa)	Relative Front	Adj. Volume (Int)	Volume (Int)	Abs. Quant.	Rel. Quant.	Band %	Lane %
1		29.2	0.597	20,008,206	25,131,042	N/A	N/A	80.8	58.0
2		20.5	0.685	1,969,950	5,267,046	N/A	N/A	8.0	5.7
3		14.5	0.766	2,783,874	6,062,202	N/A	N/A	11.2	8.1

Band Detection	Automatically detected bands with sensitivity: High
Lane Background	Lane background subtracted with disk size: 17
Lane Width	5.45 mm
Regression Equation	A single equation is not available for this method

Lane 9

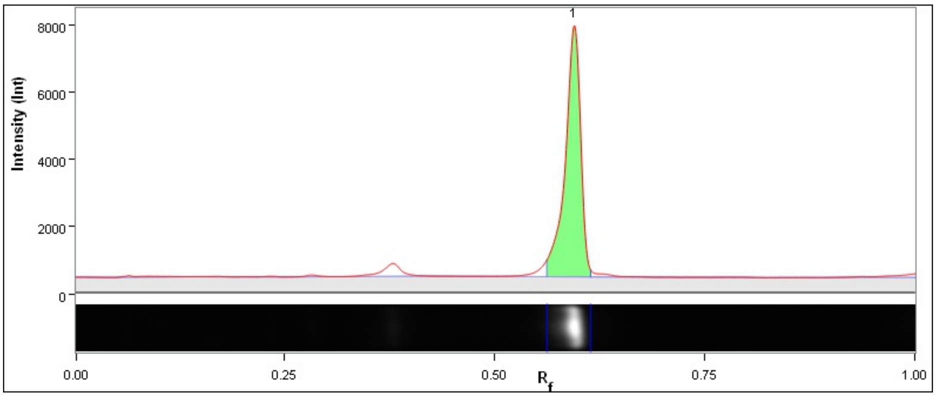

Band No.	Band Label	Mol. Wt. (KDa)	Relative Front	Adj. Volume (Int)	Volume (Int)	Abs. Quant.	Rel. Quant.	Band %	Lane %
1		29.4	0.594	40,702,686	46,981,686	N/A	N/A	100.0	85.6

Band Detection	Automatically detected bands with sensitivity: High
Lane Background	Lane background subtracted with disk size: 17
Lane Width	5.45 mm
Regression Equation	A single equation is not available for this method

Lane 10

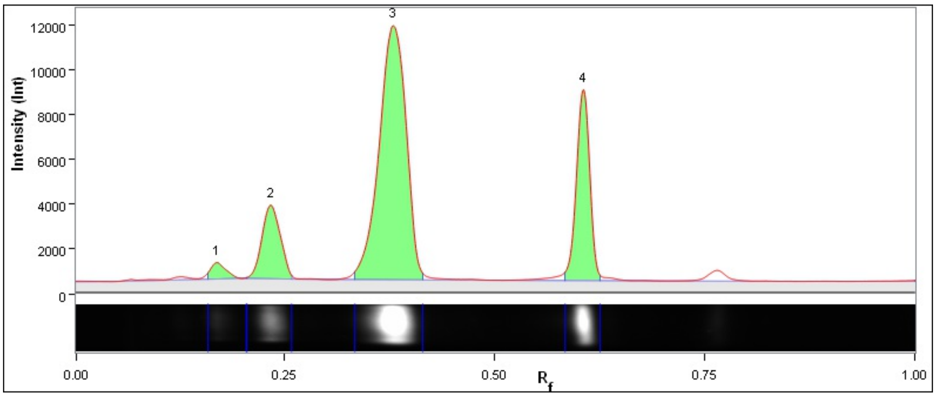

Band No.	Band Label	Mol. Wt. (KDa)	Relative Front	Adj. Volume (Int)	Volume (Int)	Abs. Quant.	Rel. Quant.	Band %	Lane %
1		62.0	0.169	3,572,544	10,247,190	N/A	N/A	2.2	2.1
2		62.0	0.234	19,669,278	27,406,938	N/A	N/A	12.3	11.7
3		54.2	0.380	99,372,558	110,198,106	N/A	N/A	62.2	58.9
4		28.5	0.605	37,065,420	42,318,528	N/A	N/A	23.2	22.0

Band Detection	Automatically detected bands with sensitivity: High
Lane Background	Lane background subtracted with disk size: 17
Lane Width	5.45 mm
Regression Equation	A single equation is not available for this method

Lane 11

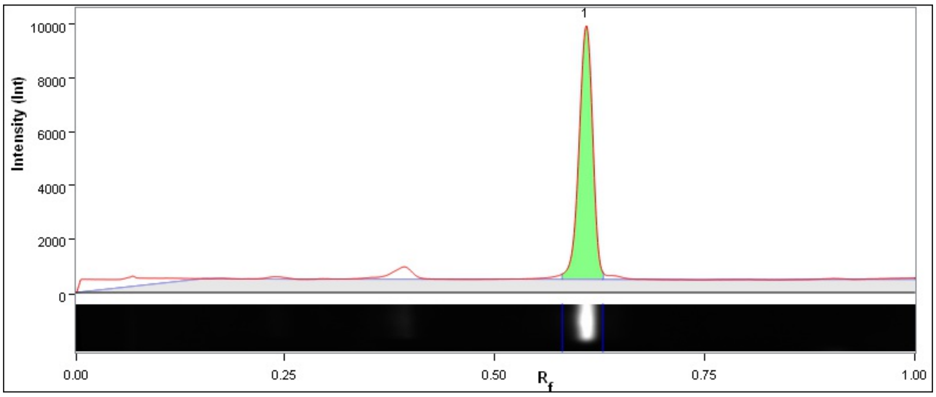

Band No.	Band Label	Mol. Wt. (KDa)	Relative Front	Adj. Volume (Int)	Volume (Int)	Abs. Quant.	Rel. Quant.	Band %	Lane %
1		28.2	0.609	42,911,238	48,692,058	N/A	N/A	100.0	73.2

Band Detection	Automatically detected bands with sensitivity: High
Lane Background	Lane background subtracted with disk size: 17
Lane Width	5.45 mm
Regression Equation	A single equation is not available for this method

Lane 12

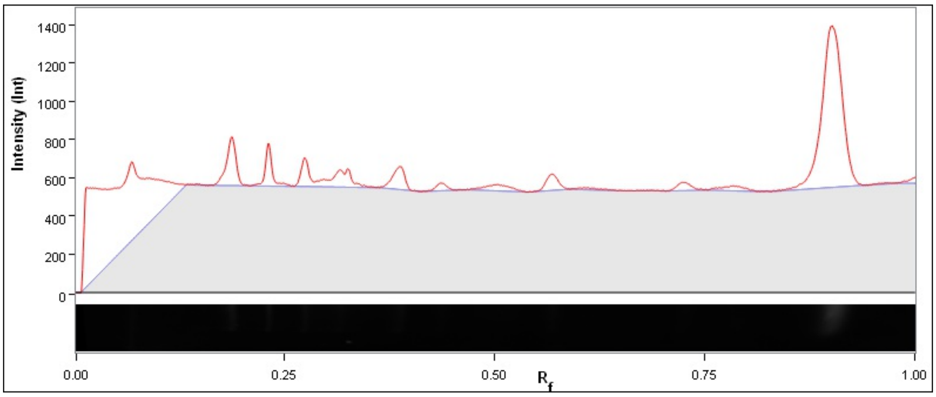

Band No.	Band Label	Mol. Wt. (KDa)	Relative Front	Adj. Volume (Int)	Volume (Int)	Abs. Quant.	Rel. Quant.	Band %	Lane %

Band Detection	Automatically detected bands with sensitivity: High
Lane Background	Lane background subtracted with disk size: 17
Lane Width	5.45 mm
Regression Equation	A single equation is not available for this method

Image Report: admin 2023-03-28 21h12m43s

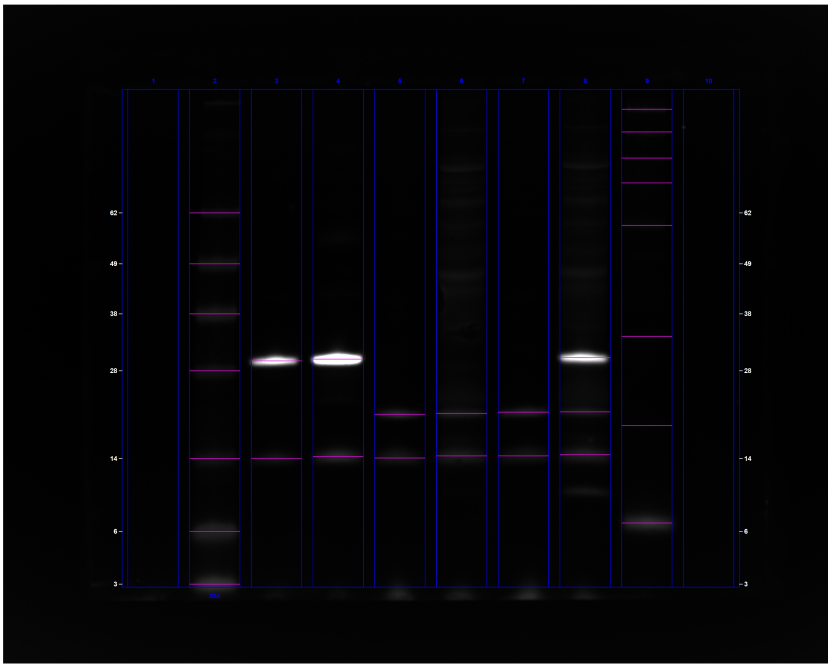

C:\Users\oded\OneDrive - University Of Cambridge\Vendruscolo\tmp\ChemiDoc Images 2023-03-28_21.19.37\admin 2023-03-28 21h12m43s.scn

Acquisition Information

Imager	ChemiDoc™ MP
Exposure Time (sec)	0.050 (Manual)
Flat Field	Applied (Red Epi)
Serial Number	734BR-3787
Software Version	2.4.0.03
Application	Alexa 647
Excitation Source	Red Epi Illumination
Emission Filter	700/50 Filter

Image Information

Acquisition Date	28/03/2023 10:12:42 PM
User Name	admin
Image Area (mm)	X: 96.8 Y: 77.4
Pixel Size (µm)	X: 35.2 Y: 35.2
Data Range (Int)	401 - 31797

Analysis Settings

Detection	Lane detection:<br>Manually created lanes

	Band detection:<br>Automatically detected bands with custom sensitivity: 77<br>Manually adjusted bands<br><br>Lane Background Subtraction:<br>Lane background subtracted with disk size: 17<br><br>Lane width: 5.91 mm
Mol. Weight Analysis	Standard: SeeBlue2<br>Standard lanes: 2<br>Regression method: Point to Point (semi-log)

Lane Statistics

Lane No.	Adj. Total Band Vol. (Int)	Total Band Vol. (Int)	Adj. Total Lane Vol. (Int)	Total Lane Vol. (Int)	Bkgd. Vol. (Int)	Norm. Factor
1	N/A	N/A	1,507,800	138,787,152	137,279,352	N/A
2	27,142,080	58,550,688	35,168,784	187,128,312	151,959,528	N/A
3	36,335,712	44,006,256	40,204,584	180,965,400	140,760,816	N/A
4	104,165,040	112,990,752	111,963,936	253,812,720	141,848,784	N/A
5	6,639,696	12,717,264	10,703,616	151,595,640	140,892,024	N/A
6	6,804,336	13,813,464	22,984,416	185,072,328	162,087,912	N/A
7	6,875,400	14,118,552	10,852,296	151,504,920	140,652,624	N/A
8	45,965,304	60,649,680	60,612,720	227,901,576	167,288,856	N/A
9	8,779,680	16,384,032	16,441,152	159,750,192	143,309,040	N/A
10	N/A	N/A	834,960	139,817,328	138,982,368	N/A

Lane And Band Analysis

Lane 1

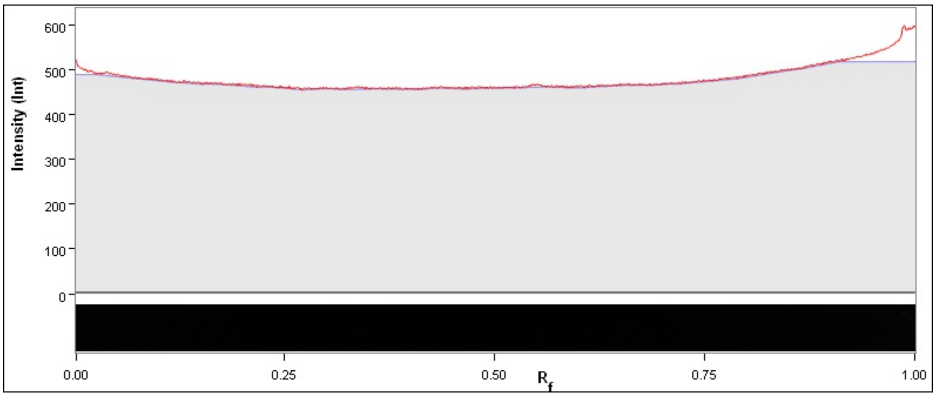

Band No.	Band Label	Mol. Wt. (KDa)	Relative Front	Adj. Volume (Int)	Volume (Int)	Abs. Quant.	Rel. Quant.	Band %	Lane %

Band Detection	Automatically detected bands with custom sensitivity: 77
Lane Background	Lane background subtracted with disk size: 17
Lane Width	5.91 mm
Regression Equation	A single equation is not available for this method

Lane 2 - SeeBlue2

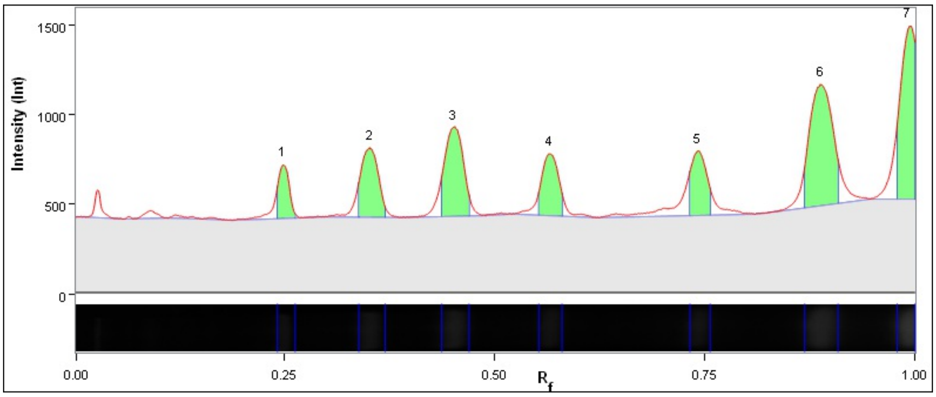

Band No.	Band Label	Mol. Wt. (KDa)	Relative Front	Adj. Volume (Int)	Volume (Int)	Abs. Quant.	Rel. Quant.	Band %	Lane %
1		62.0	0.248	1,475,040	4,613,784	N/A	N/A	5.4	4.2
2		49.0	0.350	3,070,032	7,757,568	N/A	N/A	11.3	8.7
3		38.0	0.451	4,248,216	9,176,328	N/A	N/A	15.7	12.1
4		28.0	0.565	2,612,568	6,856,584	N/A	N/A	9.6	7.4
5		14.0	0.742	2,499,336	6,216,168	N/A	N/A	9.2	7.1
6		6.0	0.888	7,245,168	14,093,016	N/A	N/A	26.7	20.6
7		3.0	0.994	5,991,720	9,837,240	N/A	N/A	22.1	17.0

Band Detection	Automatically detected bands with custom sensitivity: 77
Lane Background	Lane background subtracted with disk size: 17
Lane Width	5.91 mm
Regression Equation	A single equation is not available for this method

Lane 3

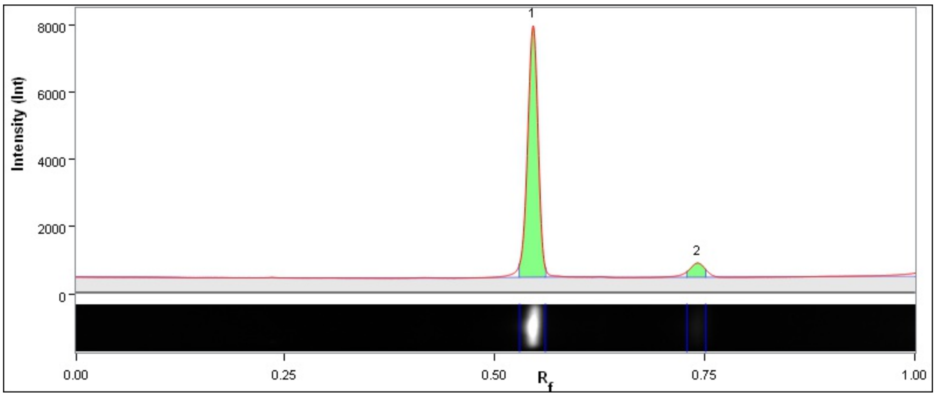

Band No.	Band Label	Mol. Wt. (KDa)	Relative Front	Adj. Volume (Int)	Volume (Int)	Abs. Quant.	Rel. Quant.	Band %	Lane %
1		29.5	0.545	33,958,008	38,393,040	N/A	N/A	93.5	84.5
2		14.0	0.741	2,377,704	5,613,216	N/A	N/A	6.5	5.9

Band Detection	Automatically detected bands with custom sensitivity: 77
Lane Background	Lane background subtracted with disk size: 17
Lane Width	5.91 mm
Regression Equation	A single equation is not available for this method

Lane 4

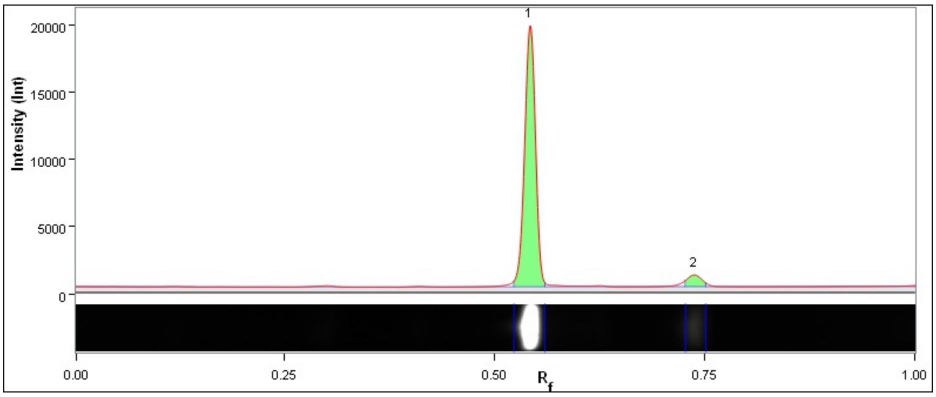

Band No.	Band Label	Mol. Wt. (KDa)	Relative Front	Adj. Volume (Int)	Volume (Int)	Abs. Quant.	Rel. Quant.	Band %	Lane %
1		29.8	0.542	98,621,712	103,882,968	N/A	N/A	94.7	88.1
2		14.2	0.738	5,543,328	9,107,784	N/A	N/A	5.3	5.0

Band Detection	Automatically detected bands with custom sensitivity: 77
Lane Background	Lane background subtracted with disk size: 17
Lane Width	5.91 mm
Regression Equation	A single equation is not available for this method

Lane 5

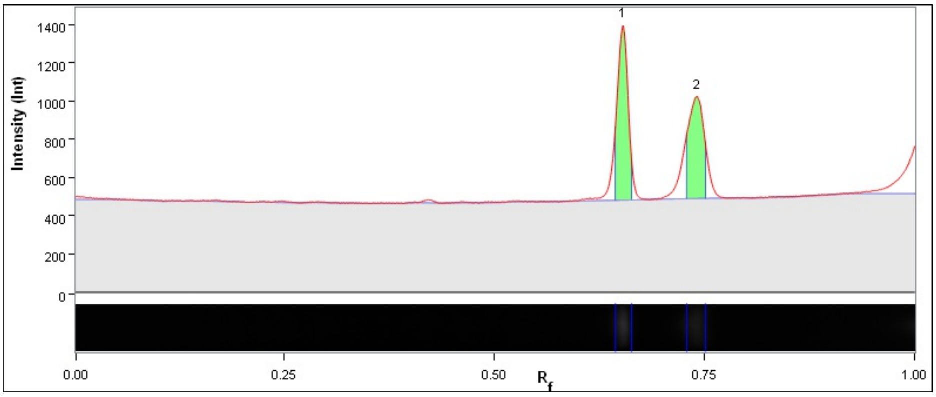

Band No.	Band Label	Mol. Wt. (KDa)	Relative Front	Adj. Volume (Int)	Volume (Int)	Abs. Quant.	Rel. Quant.	Band %	Lane %
1		19.9	0.653	3,664,752	6,464,472	N/A	N/A	55.2	34.2
2		14.1	0.741	2,974,944	6,252,792	N/A	N/A	44.8	27.8

Band Detection	Automatically detected bands with custom sensitivity: 77
Lane Background	Lane background subtracted with disk size: 17
Lane Width	5.91 mm
Regression Equation	A single equation is not available for this method

Lane 6

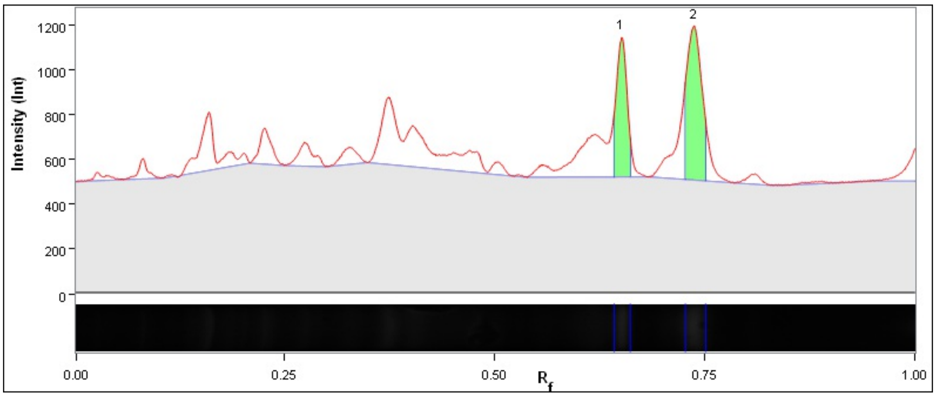

Band No.	Band Label	Mol. Wt. (KDa)	Relative Front	Adj. Volume (Int)	Volume (Int)	Abs. Quant.	Rel. Quant.	Band %	Lane %
1		20.0	0.651	2,718,744	5,894,784	N/A	N/A	40.0	11.8
2		14.3	0.736	4,085,592	7,918,680	N/A	N/A	60.0	17.8

Band Detection	Automatically detected bands with custom sensitivity: 77
Lane Background	Lane background subtracted with disk size: 17
Lane Width	5.91 mm
Regression Equation	A single equation is not available for this method

Lane 7

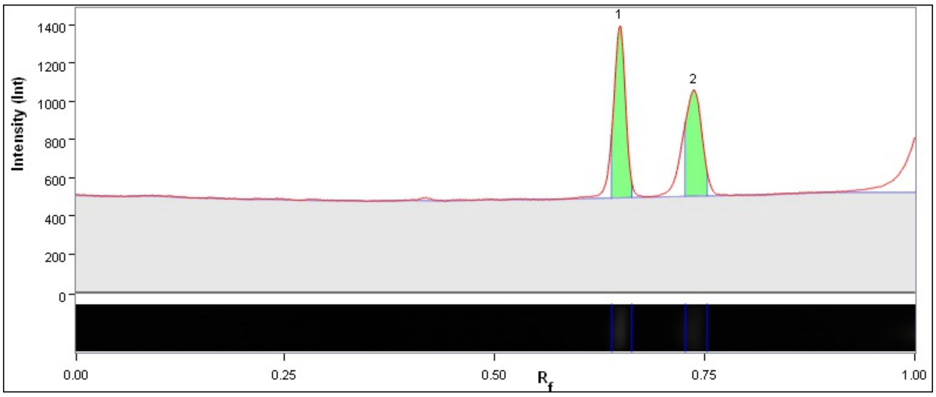

Band No.	Band Label	Mol. Wt. (KDa)	Relative Front	Adj. Volume (Int)	Volume (Int)	Abs. Quant.	Rel. Quant.	Band %	Lane %
1		20.2	0.648	3,799,320	7,262,472	N/A	N/A	55.3	35.0
2		14.3	0.736	3,076,080	6,856,080	N/A	N/A	44.7	28.3

Band Detection	Automatically detected bands with custom sensitivity: 77
Lane Background	Lane background subtracted with disk size: 17
Lane Width	5.91 mm
Regression Equation	A single equation is not available for this method

Lane 8

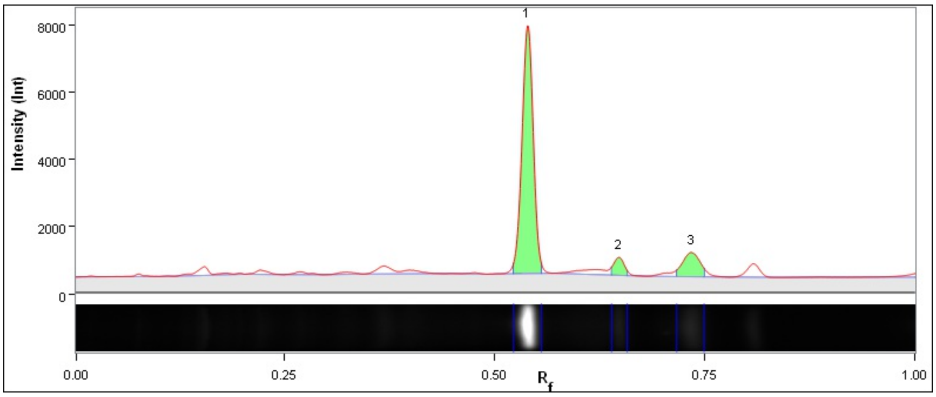

Band No.	Band Label	Mol. Wt. (KDa)	Relative Front	Adj. Volume (Int)	Volume (Int)	Abs. Quant.	Rel. Quant.	Band %	Lane %
1		30.1	0.539	37,993,704	44,271,696	N/A	N/A	82.7	62.7
2		20.2	0.648	2,445,912	5,696,544	N/A	N/A	5.3	4.0
3		14.4	0.734	5,525,688	10,681,440	N/A	N/A	12.0	9.1

Band Detection	Automatically detected bands with custom sensitivity: 77
Lane Background	Lane background subtracted with disk size: 17
Lane Width	5.91 mm
Regression Equation	A single equation is not available for this method

Lane 9

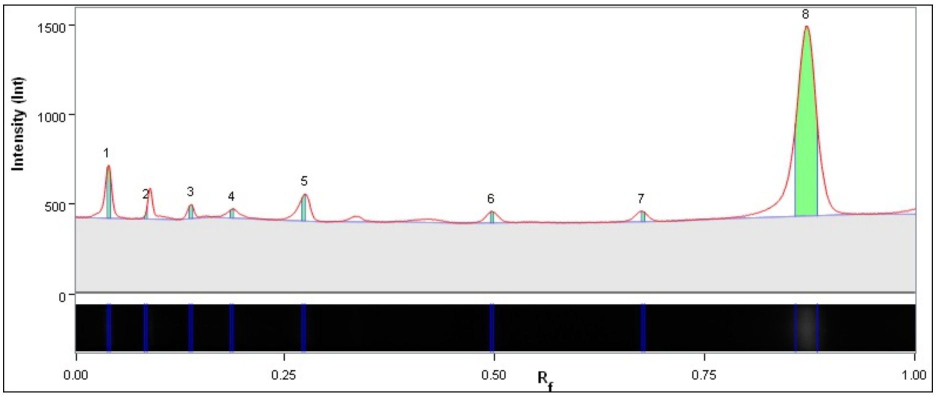

Band No.	Band Label	Mol. Wt. (KDa)	Relative Front	Adj. Volume (Int)	Volume (Int)	Abs. Quant.	Rel. Quant.	Band %	Lane %
1		62.0	0.040	350,280	878,304	N/A	N/A	4.0	2.1
2		62.0	0.085	60,144	583,296	N/A	N/A	0.7	0.4
3		62.0	0.138	95,928	621,600	N/A	N/A	1.1	0.6
4		62.0	0.188	63,336	592,872	N/A	N/A	0.7	0.4
5		58.5	0.273	184,632	692,664	N/A	N/A	2.1	1.1
6		33.7	0.496	79,800	574,728	N/A	N/A	0.9	0.5
7		18.2	0.675	69,720	573,720	N/A	N/A	0.8	0.4
8		6.6	0.871	7,875,840	11,866,848	N/A	N/A	89.7	47.9

Band Detection	Automatically detected bands with custom sensitivity: 77
Lane Background	Lane background subtracted with disk size: 17

Lane Width	5.91 mm
Regression Equation	A single equation is not available for this method

Lane 10

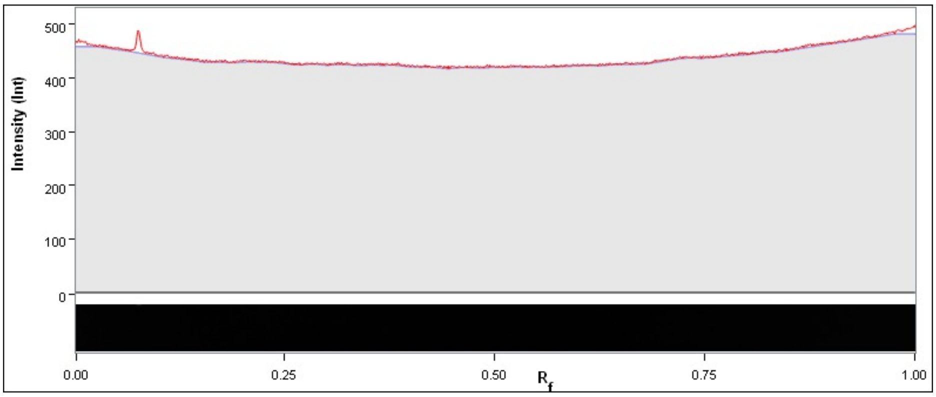

Band No.	Band Label	Mol. Wt. (KDa)	Relative Front	Adj. Volume (Int)	Volume (Int)	Abs. Quant.	Rel. Quant.	Band %	Lane %

Band Detection	Automatically detected bands with custom sensitivity: 77
Lane Background	Lane background subtracted with disk size: 17
Lane Width	5.91 mm
Regression Equation	A single equation is not available for this method

Image Report: admin 2023-10-11 13h49m54s

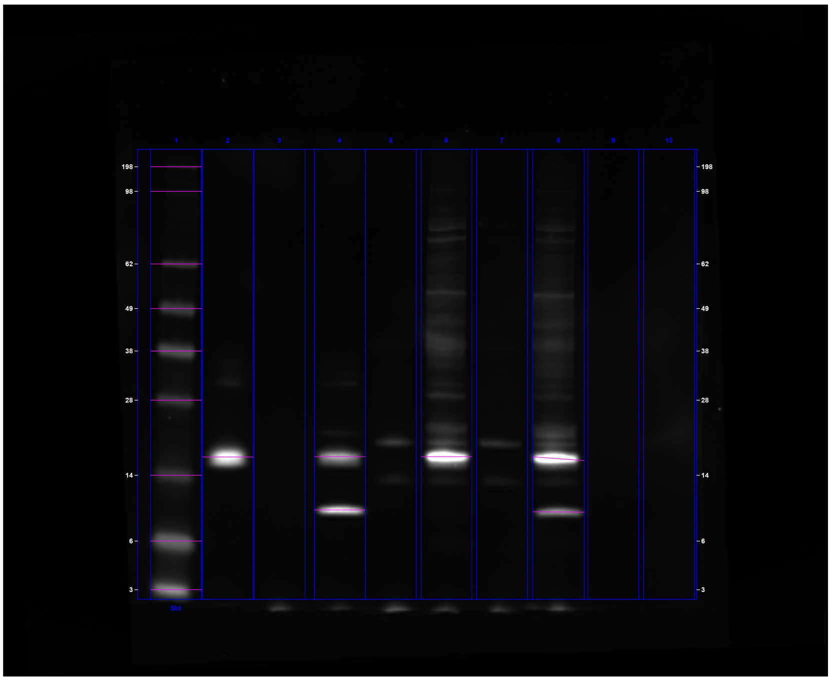

C:\Users\oded\OneDrive - University Of Cambridge\Vendruscolo\Results\Western\ChemiDoc Images 2023-10-11_19.07.33\admin 2023-10-11 13h49m54s.scn

Acquisition Information

Imager	ChemiDoc™ MP
Exposure Time (sec)	0.500 (Manual)
Flat Field	Applied (Red Epi)
Serial Number	734BR-3787
Software Version	2.4.0.03
Application	Alexa 647
Excitation Source	Red Epi Illumination
Emission Filter	700/50 Filter

Image Information

Acquisition Date	11/10/2023 2:49:54 PM
User Name	admin
Image Area (mm)	X: 111.8 Y: 91.0
Pixel Size (µm)	X: 39.4 Y: 39.4
Data Range (Int)	0 - 46274

Analysis Settings

Detection	Lane detection:<br>Manually created lanes

1

	Band detection:<br><br>Manually adjusted bands<br><br>Lane Background Subtraction:<br>Lane background subtracted with disk size: 17<br><br>Lane width: Variable
Mol. Weight Analysis	Standard: SeeBlue2-full<br>Standard lanes: first<br>Regression method: Point to Point (semi-log)

Lane Statistics

Lane No.	Adj. Total Band Vol. (Int)	Total Band Vol. (Int)	Adj. Total Lane Vol. (Int)	Total Lane Vol. (Int)	Bkgd. Vol. (Int)	Norm. Factor
1	259,340,900	394,815,750	312,136,300	723,759,225	411,622,925	N/A
2	157,019,800	176,464,750	184,947,525	454,559,000	269,611,475	N/A
3	N/A	N/A	22,922,900	275,679,950	252,757,050	N/A
4	183,521,975	210,867,125	214,878,475	480,766,300	265,887,825	N/A
5	N/A	N/A	36,054,200	305,802,350	269,748,150	N/A
6	198,652,475	220,619,875	334,509,875	761,133,275	426,623,400	N/A
7	N/A	N/A	33,145,875	329,332,325	296,186,450	N/A
8	248,770,137	287,914,926	350,548,146	814,955,790	464,407,644	N/A
9	N/A	N/A	21,283,325	304,686,550	283,403,225	N/A
10	N/A	N/A	40,710,075	373,178,050	332,467,975	N/A

Lane And Band Analysis

Lane 1 - SeeBlue2-full

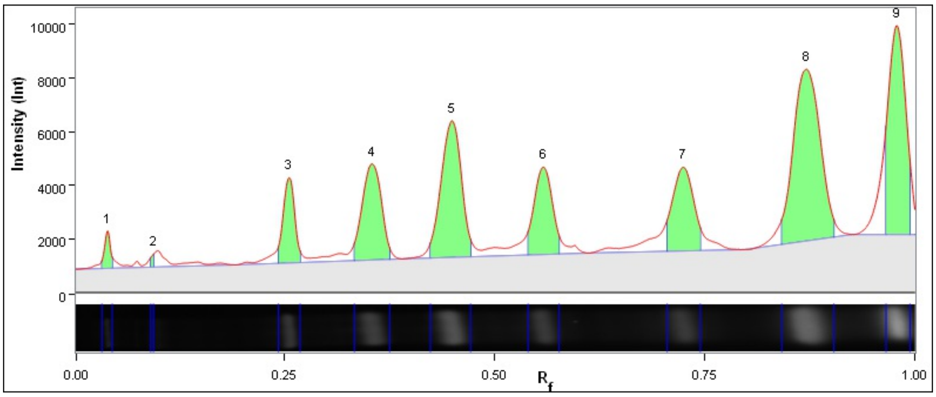

Band No.	Band Label	Mol. Wt. (KDa)	Relative Front	Adj. Volume (Int)	Volume (Int)	Abs. Quant.	Rel. Quant.	Band %	Lane %
1		198.0	0.039	3,266,550	6,695,500	N/A	N/A	1.3	1.0
2		98.0	0.094	506,800	1,588,475	N/A	N/A	0.2	0.2
3		62.0	0.255	14,820,750	23,421,650	N/A	N/A	5.7	4.7
4		49.0	0.354	27,891,150	43,129,275	N/A	N/A	10.8	8.9
5		38.0	0.448	41,808,200	60,506,425	N/A	N/A	16.1	13.4
6		28.0	0.558	23,892,750	39,825,625	N/A	N/A	9.2	7.7
7		14.0	0.724	25,997,475	44,299,850	N/A	N/A	10.0	8.3
8		6.0	0.871	69,950,125	105,619,850	N/A	N/A	27.0	22.4
9		3.0	0.979	51,207,100	69,729,100	N/A	N/A	19.7	16.4

Lane Background	Lane background subtracted with disk size: 17
Lane Width	6.90 mm
Regression Equation	A single equation is not available for this method

Lane 2

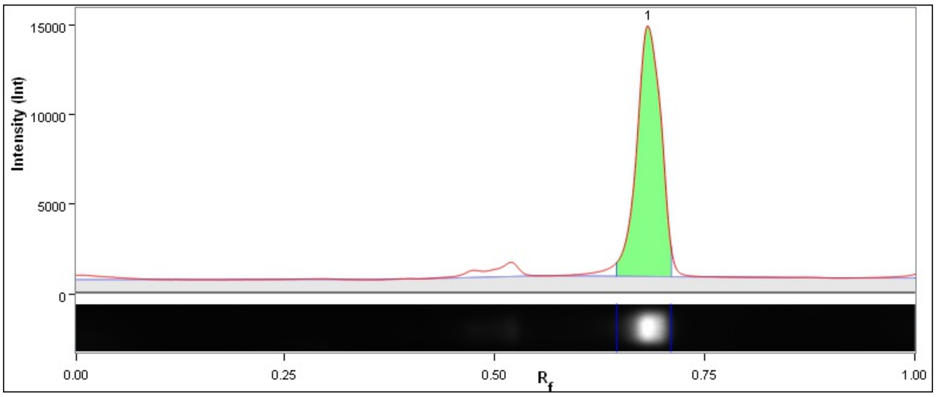

Band No.	Band Label	Mol. Wt. (KDa)	Relative Front	Adj. Volume (Int)	Volume (Int)	Abs. Quant.	Rel. Quant.	Band %	Lane %
1		16.6	0.684	157,019,800	176,464,750	N/A	N/A	100.0	84.9

Lane Background	Lane background subtracted with disk size: 17
Lane Width	6.90 mm
Regression Equation	A single equation is not available for this method

Lane 3

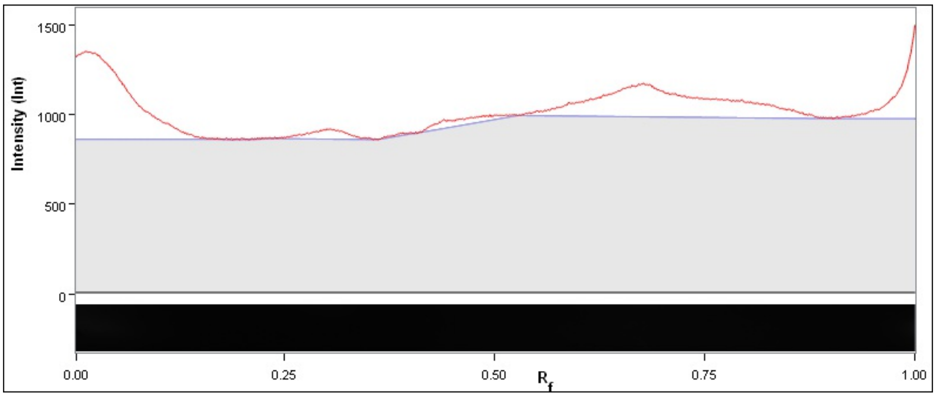

Band No.	Band Label	Mol. Wt. (KDa)	Relative Front	Adj. Volume (Int)	Volume (Int)	Abs. Quant.	Rel. Quant.	Band %	Lane %

Lane Background	Lane background subtracted with disk size: 17
Lane Width	6.90 mm
Regression Equation	A single equation is not available for this method

Lane 4

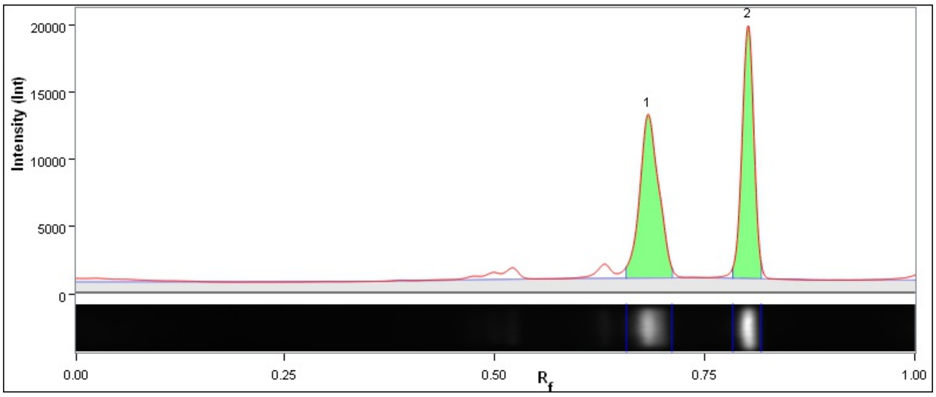

Band No.	Band Label	Mol. Wt. (KDa)	Relative Front	Adj. Volume (Int)	Volume (Int)	Abs. Quant.	Rel. Quant.	Band %	Lane %
1		16.6	0.683	93,530,150	110,710,600	N/A	N/A	51.0	43.5
2		9.0	0.801	89,991,825	100,156,525	N/A	N/A	49.0	41.9

Lane Background	Lane background subtracted with disk size: 17
Lane Width	6.90 mm
Regression Equation	A single equation is not available for this method

Lane 5

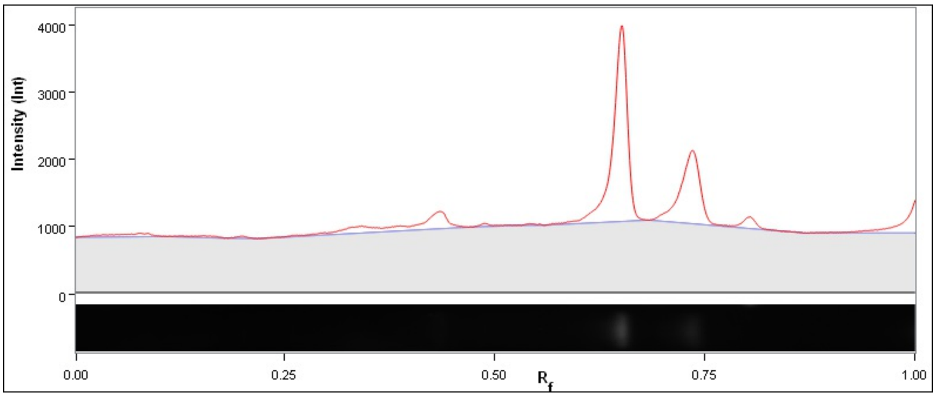

Band No.	Band Label	Mol. Wt. (KDa)	Relative Front	Adj. Volume (Int)	Volume (Int)	Abs. Quant.	Rel. Quant.	Band %	Lane %

Lane Background	Lane background subtracted with disk size: 17
Lane Width	6.90 mm
Regression Equation	A single equation is not available for this method

Lane 6

4

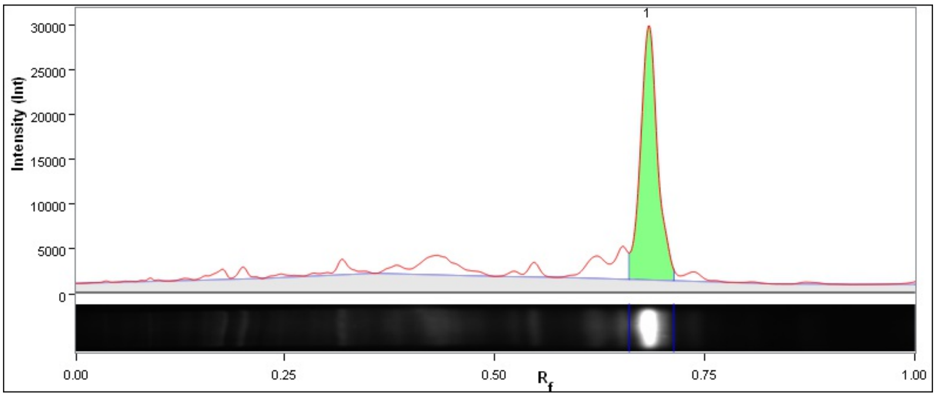

Band No.	Band Label	Mol. Wt. (KDa)	Relative Front	Adj. Volume (Int)	Volume (Int)	Abs. Quant.	Rel. Quant.	Band %	Lane %
1		16.6	0.683	198,652,475	220,619,875	N/A	N/A	100.0	59.4

Lane Background	Lane background subtracted with disk size: 17
Lane Width	6.90 mm
Regression Equation	A single equation is not available for this method

Lane 7

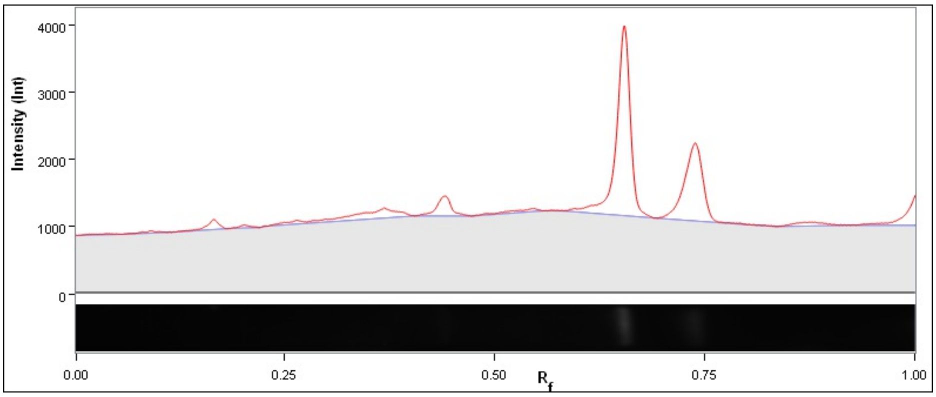

Band No.	Band Label	Mol. Wt. (KDa)	Relative Front	Adj. Volume (Int)	Volume (Int)	Abs. Quant.	Rel. Quant.	Band %	Lane %

Lane Background	Lane background subtracted with disk size: 17
Lane Width	6.90 mm
Regression Equation	A single equation is not available for this method

Lane 8

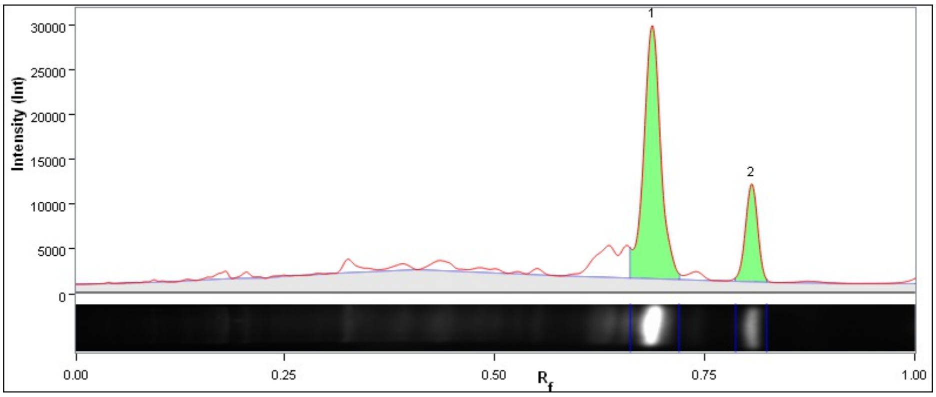

Band No.	Band Label	Mol. Wt. (KDa)	Relative Front	Adj. Volume (Int)	Volume (Int)	Abs. Quant.	Rel. Quant.	Band %	Lane %
1		16.3	0.688	194,559,285	220,717,584	N/A	N/A	78.2	55.5
2		8.7	0.806	54,210,852	67,197,342	N/A	N/A	21.8	15.5

Lane Background	Lane background subtracted with disk size: 17
Lane Width	6.98 mm
Regression Equation	A single equation is not available for this method

Lane 9

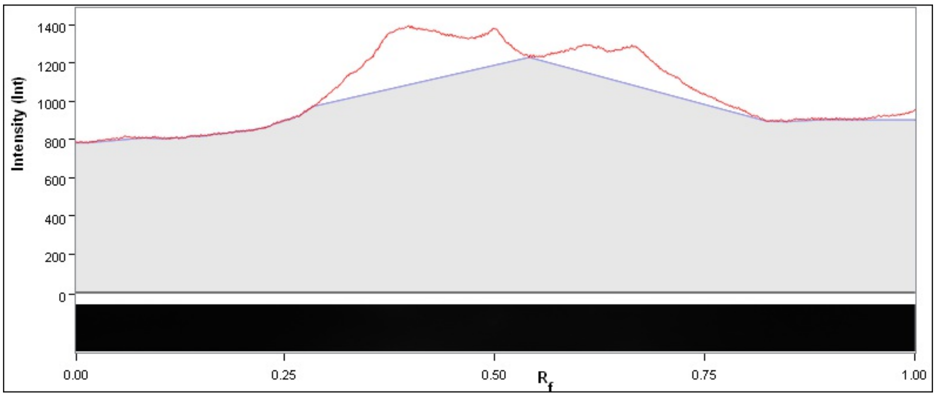

Band No.	Band Label	Mol. Wt. (KDa)	Relative Front	Adj. Volume (Int)	Volume (Int)	Abs. Quant.	Rel. Quant.	Band %	Lane %

Lane Background	Lane background subtracted with disk size: 17
Lane Width	6.90 mm
Regression Equation	A single equation is not available for this method

Lane 10

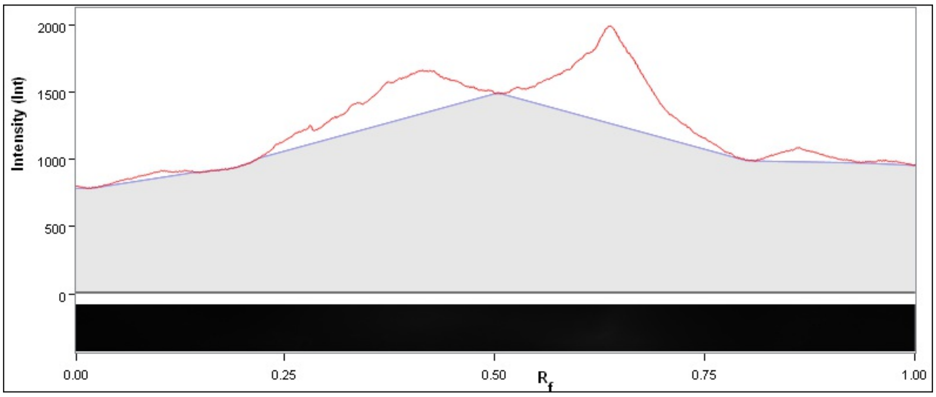

Band No.	Band Label	Mol. Wt. (KDa)	Relative Front	Adj. Volume (Int)	Volume (Int)	Abs. Quant.	Rel. Quant.	Band %	Lane %

Lane Background	Lane background subtracted with disk size: 17
Lane Width	6.90 mm
Regression Equation	A single equation is not available for this method

Image Report: admin 2023-10-12 14h11m42s

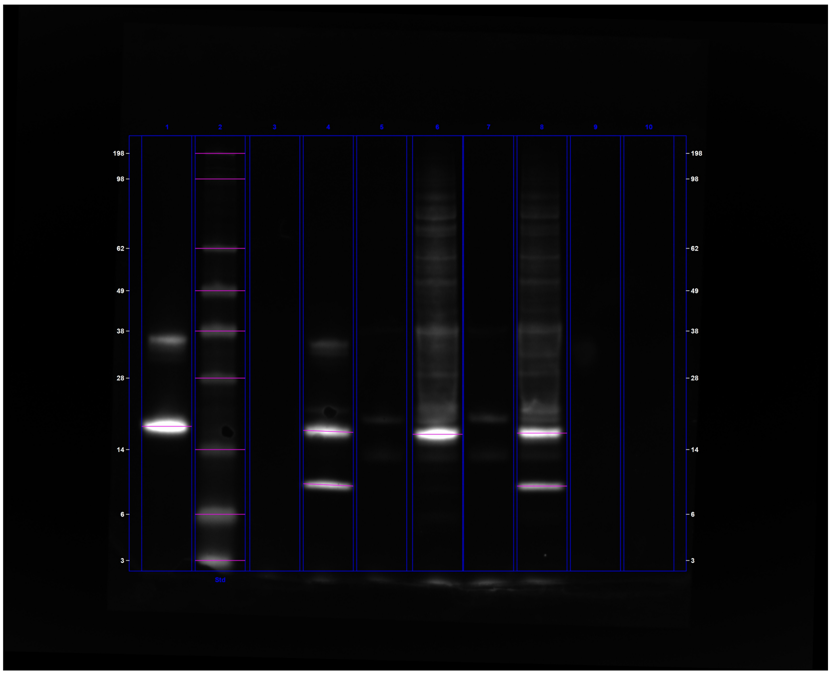

C:\Users\oded\OneDrive - University Of Cambridge\Vendruscolo\Results\Western\ChemiDoc Images 2023-10-12_19.02.38\admin 2023-10-12 14h11m42s.scn

---

Acquisition Information

Imager	ChemiDoc™ MP
Exposure Time (sec)	0.200 (Manual)
Flat Field	Applied (Red Epi)
Serial Number	734BR-3787
Software Version	2.4.0.03
Application	Alexa 647
Excitation Source	Red Epi Illumination
Emission Filter	700/50 Filter

Image Information

Acquisition Date	12/10/2023 3:11:42 PM
User Name	admin
Image Area (mm)	X: 115.0 Y: 92.9
Pixel Size (µm)	X: 41.0 Y: 41.0
Data Range (Int)	0 - 24086

Analysis Settings

Detection	Lane detection:<br>Manually created lanes

	Band detection:<br><br>Manually adjusted bands<br><br>Lane Background Subtraction:<br>Lane background subtracted with disk size: 17<br><br>Lane width: 6.98 mm
Mol. Weight Analysis	Standard: SeeBlue2-full<br>Standard lanes: 2<br>Regression method: Point to Point (semi-log)

Lane Statistics

Lane No.	Adj. Total Band Vol. (Int)	Total Band Vol. (Int)	Adj. Total Lane Vol. (Int)	Total Lane Vol. (Int)	Bkgd. Vol. (Int)	Norm. Factor
1	109,744,520	121,707,590	146,390,910	325,867,730	179,476,820	N/A
2	94,940,240	173,478,880	107,879,790	338,760,360	230,880,570	N/A
3	N/A	N/A	2,556,290	171,606,330	169,050,040	N/A
4	96,699,060	116,670,150	116,051,520	293,162,790	177,111,270	N/A
5	N/A	N/A	8,463,280	181,067,170	172,603,890	N/A
6	87,943,720	104,739,890	168,372,420	436,918,700	268,546,280	N/A
7	N/A	N/A	11,267,600	185,231,660	173,964,060	N/A
8	93,459,710	117,892,620	157,031,550	407,621,920	250,590,370	N/A
9	N/A	N/A	4,614,310	171,628,770	167,014,460	N/A
10	N/A	N/A	1,434,970	168,972,860	167,537,890	N/A

Lane And Band Analysis

Lane 1

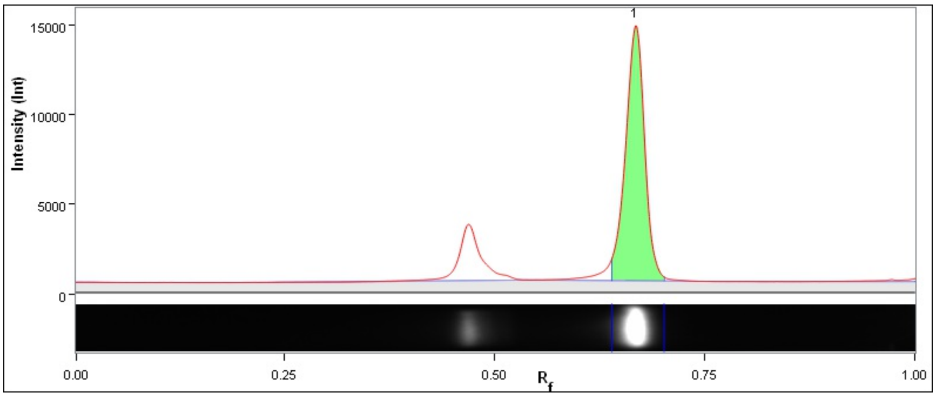

Band No.	Band Label	Mol. Wt. (KDa)	Relative Front	Adj. Volume (Int)	Volume (Int)	Abs. Quant.	Rel. Quant.	Band %	Lane %
1		17.5	0.668	109,744,520	121,707,590	N/A	N/A	100.0	75.0

Lane Background	Lane background subtracted with disk size: 17
Lane Width	6.98 mm
Regression Equation	A single equation is not available for this method

Lane 2 - SeeBlue2-full

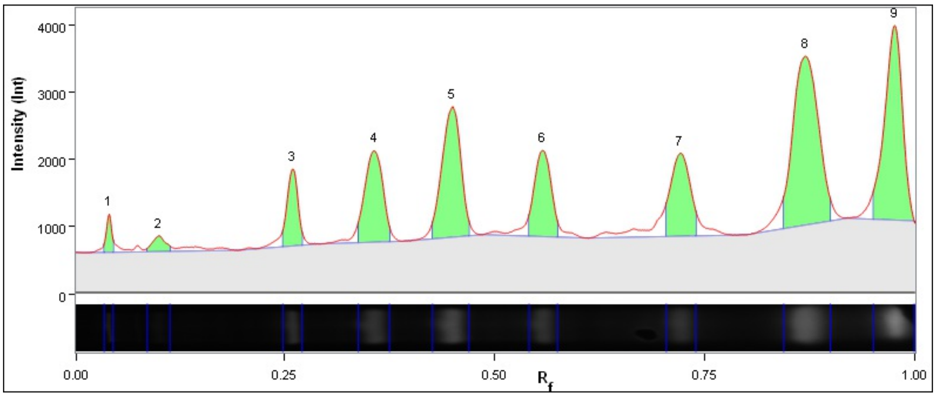

Band No.	Band Label	Mol. Wt. (KDa)	Relative Front	Adj. Volume (Int)	Volume (Int)	Abs. Quant.	Rel. Quant.	Band %	Lane %
1		198.0	0.041	1,177,420	3,151,970	N/A	N/A	1.2	1.1
2		98.0	0.099	1,203,090	6,060,500	N/A	N/A	1.3	1.1
3		62.0	0.259	4,884,950	9,598,540	N/A	N/A	5.1	4.5
4		49.0	0.356	9,675,720	17,831,130	N/A	N/A	10.2	9.0
5		38.0	0.449	14,461,900	24,838,530	N/A	N/A	15.2	13.4
6		28.0	0.557	8,450,530	16,701,140	N/A	N/A	8.9	7.8
7		14.0	0.721	9,223,520	17,859,010	N/A	N/A	9.7	8.5
8		6.0	0.870	25,322,520	41,586,930	N/A	N/A	26.7	23.5
9		3.0	0.976	20,540,590	35,851,130	N/A	N/A	21.6	19.0

Lane Background	Lane background subtracted with disk size: 17
Lane Width	6.98 mm
Regression Equation	A single equation is not available for this method

Lane 3

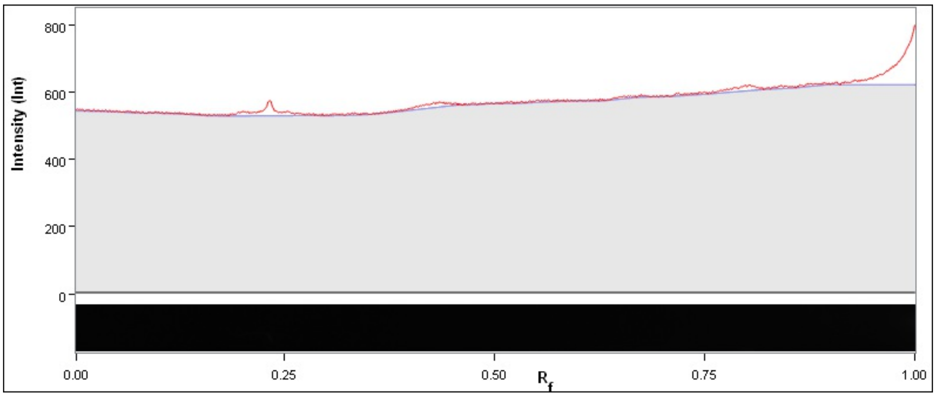

Band No.	Band Label	Mol. Wt. (KDa)	Relative Front	Adj. Volume (Int)	Volume (Int)	Abs. Quant.	Rel. Quant.	Band %	Lane %

Lane Background	Lane background subtracted with disk size: 17
Lane Width	6.98 mm
Regression Equation	A single equation is not available for this method

Lane 4

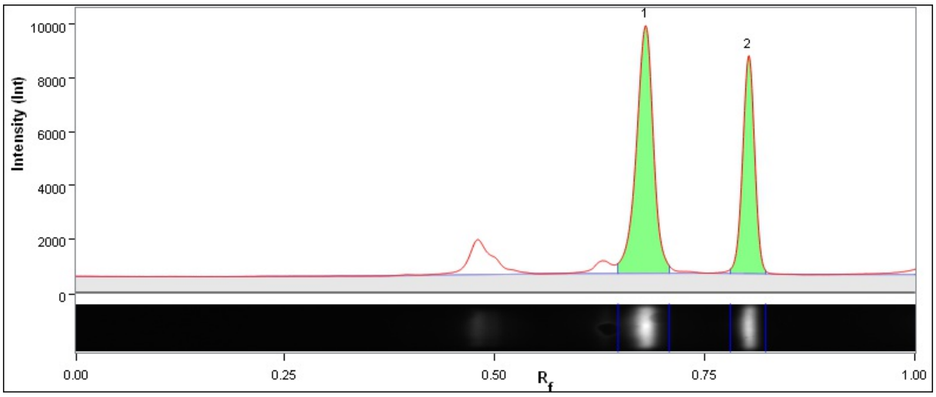

Band No.	Band Label	Mol. Wt. (KDa)	Relative Front	Adj. Volume (Int)	Volume (Int)	Abs. Quant.	Rel. Quant.	Band %	Lane %
1		16.7	0.679	59,712,500	71,603,660	N/A	N/A	61.8	51.5
2		8.8	0.802	36,986,560	45,066,490	N/A	N/A	38.2	31.9

Lane Background	Lane background subtracted with disk size: 17
Lane Width	6.98 mm
Regression Equation	A single equation is not available for this method

Lane 5

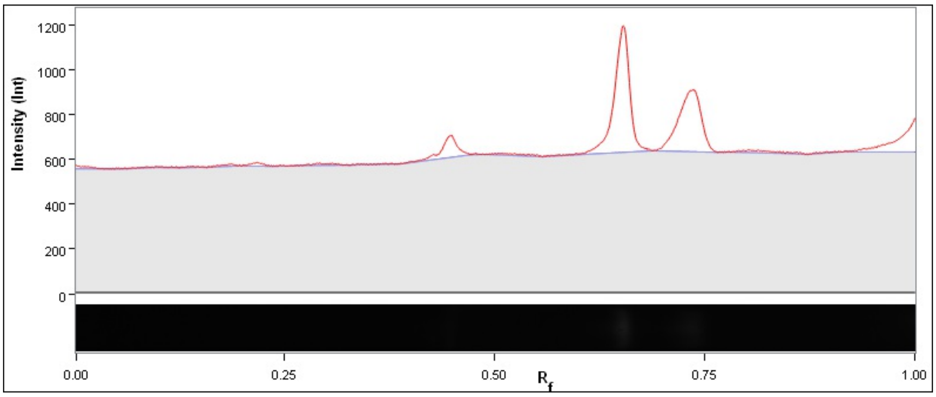

Band No.	Band Label	Mol. Wt. (KDa)	Relative Front	Adj. Volume (Int)	Volume (Int)	Abs. Quant.	Rel. Quant.	Band %	Lane %

Lane Background	Lane background subtracted with disk size: 17
Lane Width	6.98 mm
Regression Equation	A single equation is not available for this method

Lane 6

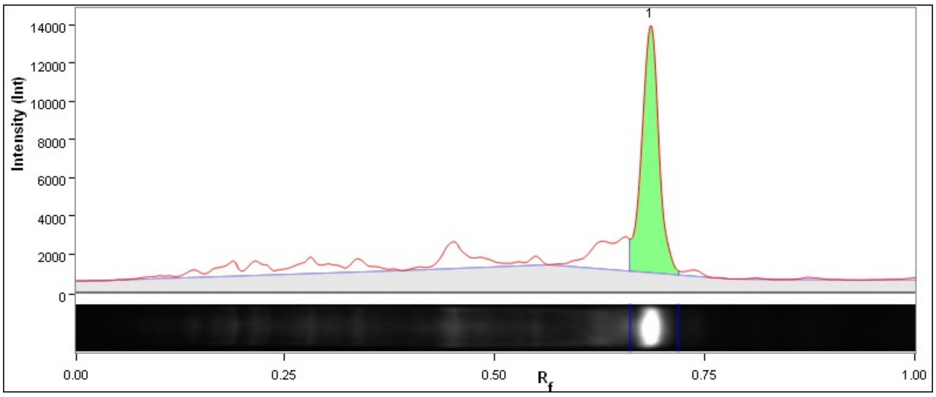

Band No.	Band Label	Mol. Wt. (KDa)	Relative Front	Adj. Volume (Int)	Volume (Int)	Abs. Quant.	Rel. Quant.	Band %	Lane %
1		16.2	0.686	87,943,720	104,739,890	N/A	N/A	100.0	52.2

Lane Background	Lane background subtracted with disk size: 17
Lane Width	6.98 mm
Regression Equation	A single equation is not available for this method

Lane 7

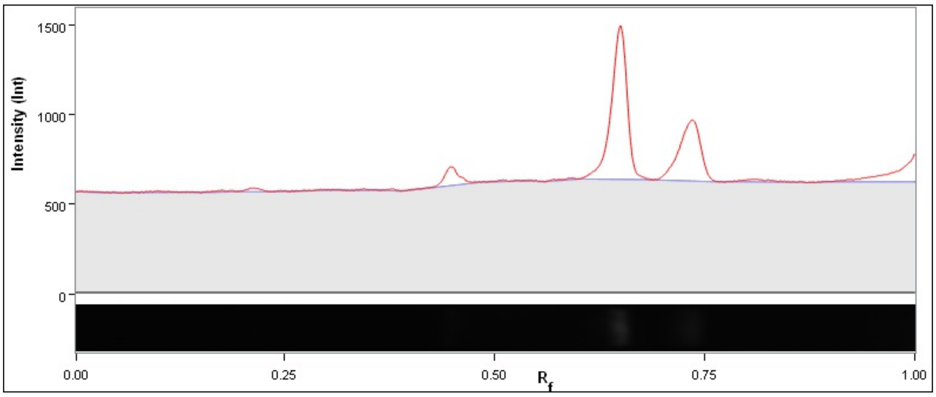

Band No.	Band Label	Mol. Wt. (KDa)	Relative Front	Adj. Volume (Int)	Volume (Int)	Abs. Quant.	Rel. Quant.	Band %	Lane %

Lane Background	Lane background subtracted with disk size: 17
Lane Width	6.98 mm
Regression Equation	A single equation is not available for this method

Lane 8

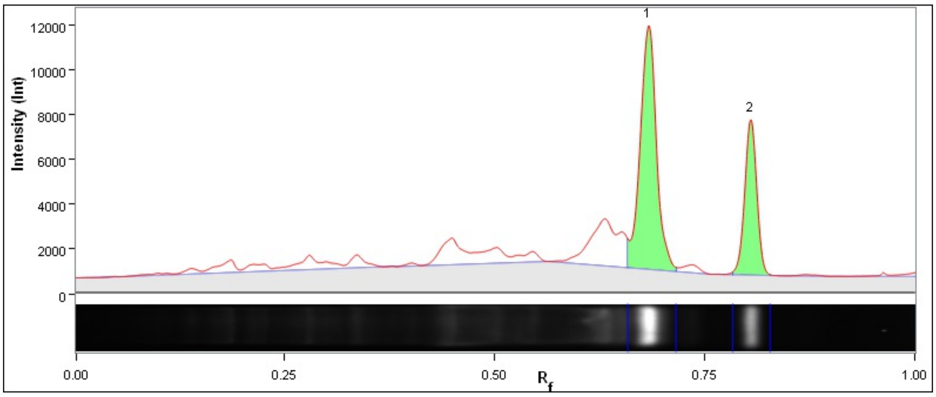

Band No.	Band Label	Mol. Wt. (KDa)	Relative Front	Adj. Volume (Int)	Volume (Int)	Abs. Quant.	Rel. Quant.	Band %	Lane %
1		16.4	0.683	63,452,500	78,796,190	N/A	N/A	67.9	40.4
2		8.7	0.805	30,007,210	39,096,430	N/A	N/A	32.1	19.1

Lane Background	Lane background subtracted with disk size: 17
Lane Width	6.98 mm
Regression Equation	A single equation is not available for this method

Lane 9

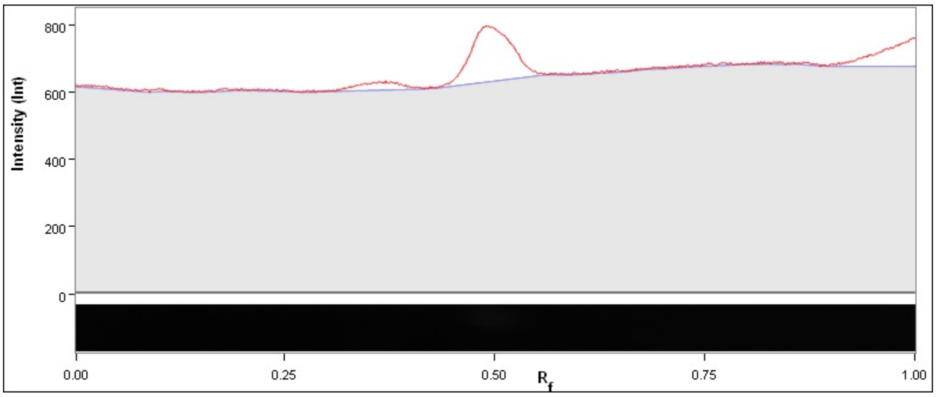

Band No.	Band Label	Mol. Wt. (KDa)	Relative Front	Adj. Volume (Int)	Volume (Int)	Abs. Quant.	Rel. Quant.	Band %	Lane %

Lane Background	Lane background subtracted with disk size: 17
Lane Width	6.98 mm
Regression Equation	A single equation is not available for this method

Lane 10

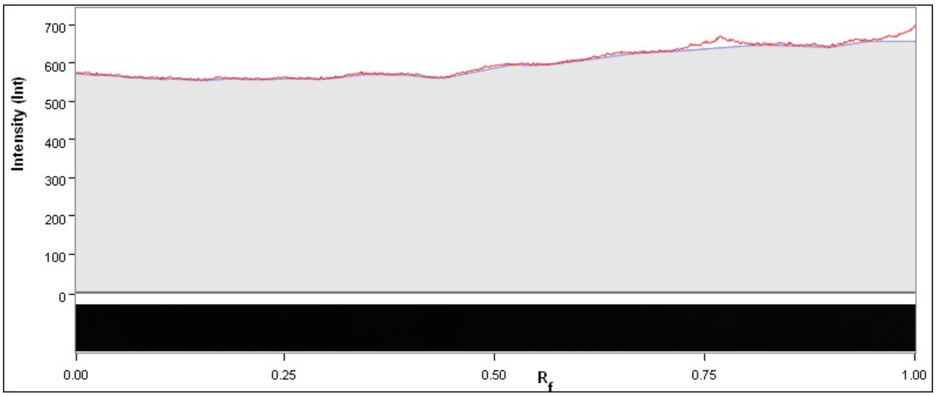

Band No.	Band Label	Mol. Wt. (KDa)	Relative Front	Adj. Volume (Int)	Volume (Int)	Abs. Quant.	Rel. Quant.	Band %	Lane %

Lane Background	Lane background subtracted with disk size: 17
Lane Width	6.98 mm
Regression Equation	A single equation is not available for this method

Image Report: admin 2023-10-13 14h06m50s

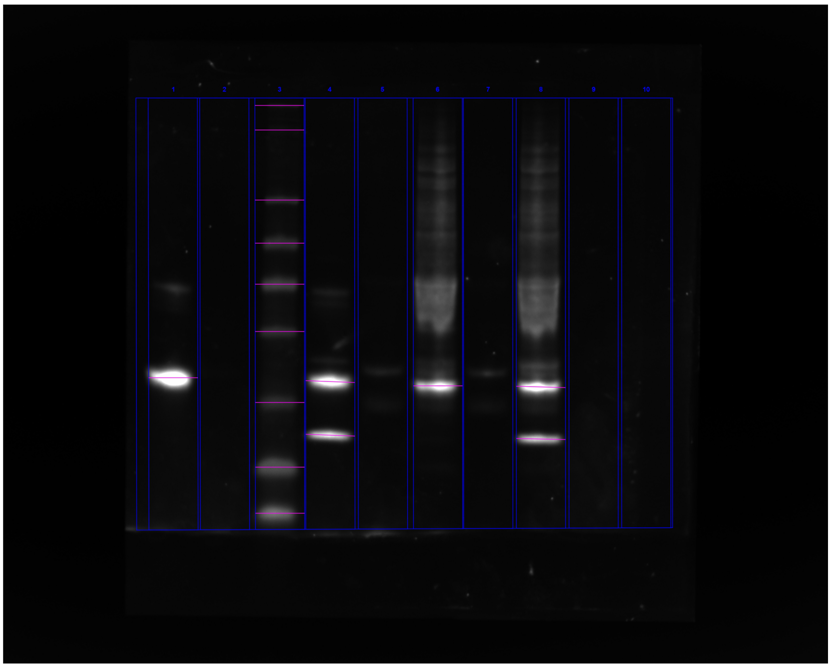

C:\Users\oded\OneDrive - University Of Cambridge\Vendruscolo\Results\Western\ChemiDoc Images 2023-10-13_14.07.42\admin 2023-10-13 14h06m50s.scn

---

Acquisition Information

Imager	ChemiDoc™ MP
Exposure Time (sec)	0.200 (Manual)
Flat Field	Applied (Red Epi)
Serial Number	734BR-3787
Software Version	2.4.0.03
Application	Alexa 647
Excitation Source	Red Epi Illumination
Emission Filter	700/50 Filter

Image Information

Acquisition Date	13/10/2023 3:06:50 PM
User Name	admin
Image Area (mm)	X: 117.5 Y: 94.0
Pixel Size (μm)	X: 42.7 Y: 42.7
Data Range (Int)	397 - 33545

Analysis Settings

Detection	Lane detection:<br>Manually created lanes

	Band detection:<br>Automatically detected bands with sensitivity: Low<br>Manually adjusted bands<br><br>Lane Background Subtraction:<br>Lane background subtracted with disk size: 7<br><br>Lane width: 7.00 mm

Lane Statistics

Lane No.	Adj. Total Band Vol. (Int)	Total Band Vol. (Int)	Adj. Total Lane Vol. (Int)	Total Lane Vol. (Int)	Bkgd. Vol. (Int)	Norm. Factor
1	134,328,956	152,699,416	154,314,324	384,231,664	229,917,340	N/A
2	N/A	N/A	4,811,924	227,122,616	222,310,692	N/A
3	110,721,156	250,304,508	115,003,524	395,984,560	280,981,036	N/A
4	126,761,176	155,714,720	141,992,020	374,855,456	232,863,436	N/A
5	N/A	N/A	13,780,592	233,733,292	219,952,700	N/A
6	70,694,168	87,575,180	185,675,552	519,711,736	334,036,184	N/A
7	N/A	N/A	13,248,248	225,282,864	212,034,616	N/A
8	119,098,276	153,574,356	184,316,812	599,866,244	415,549,432	N/A
9	N/A	N/A	4,797,164	232,455,568	227,658,404	N/A
10	N/A	N/A	4,521,480	224,646,052	220,124,572	N/A

Lane And Band Analysis

Lane 1

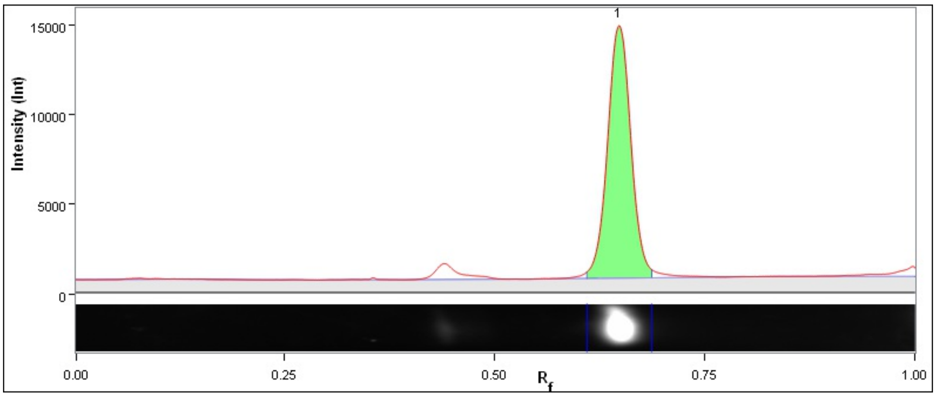

Band No.	Band Label	Mol. Wt. (KDa)	Relative Front	Adj. Volume (Int)	Volume (Int)	Abs. Quant.	Rel. Quant.	Band %	Lane %
1		N/A	0.648	134,328,956	152,699,416	N/A	N/A	100.0	87.0

Band Detection	Automatically detected bands with sensitivity: Low
Lane Background	Lane background subtracted with disk size: 7
Lane Width	7.00 mm

Lane 2

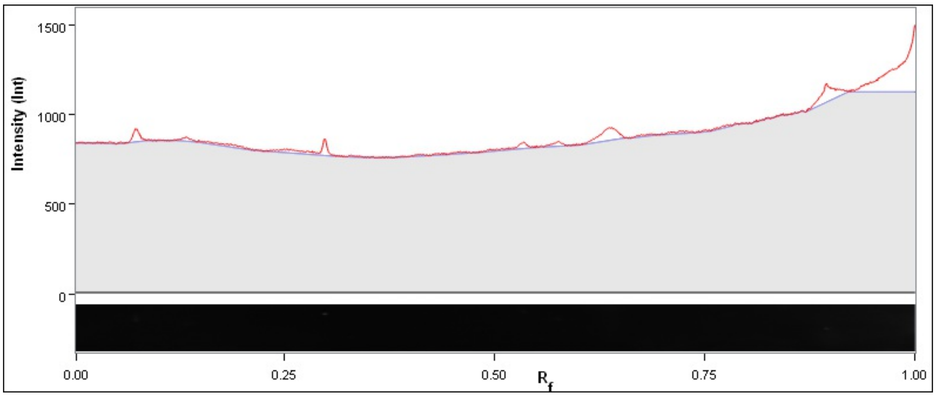

Band No.	Band Label	Mol. Wt. (KDa)	Relative Front	Adj. Volume (Int)	Volume (Int)	Abs. Quant.	Rel. Quant.	Band %	Lane %

Band Detection	Automatically detected bands with sensitivity: Low
Lane Background	Lane background subtracted with disk size: 7
Lane Width	7.00 mm

Lane 3

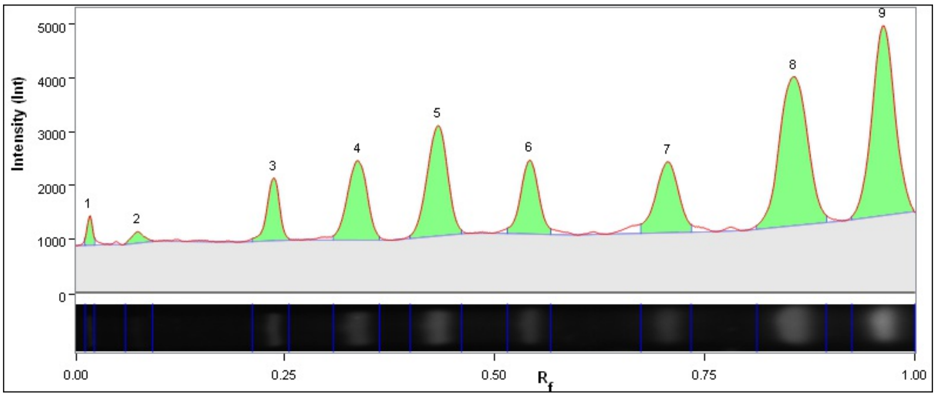

Band No.	Band Label	Mol. Wt. (KDa)	Relative Front	Adj. Volume (Int)	Volume (Int)	Abs. Quant.	Rel. Quant.	Band %	Lane %
1		N/A	0.017	1,071,084	3,767,736	N/A	N/A	1.0	0.9
2		N/A	0.074	968,584	8,950,792	N/A	N/A	0.9	0.8
3		N/A	0.237	5,366,244	16,457,728	N/A	N/A	4.8	4.7
4		N/A	0.337	10,614,408	24,685,280	N/A	N/A	9.6	9.2
5		N/A	0.432	15,263,480	32,099,064	N/A	N/A	13.8	13.3
6		N/A	0.541	8,885,848	23,614,688	N/A	N/A	8.0	7.7
7		N/A	0.706	10,933,716	28,378,232	N/A	N/A	9.9	9.5
8		N/A	0.856	28,416,608	55,255,044	N/A	N/A	25.7	24.7
9		N/A	0.963	29,201,184	57,095,944	N/A	N/A	26.4	25.4

Band Detection	Automatically detected bands with sensitivity: Low
Lane Background	Lane background subtracted with disk size: 7
Lane Width	7.00 mm

Lane 4

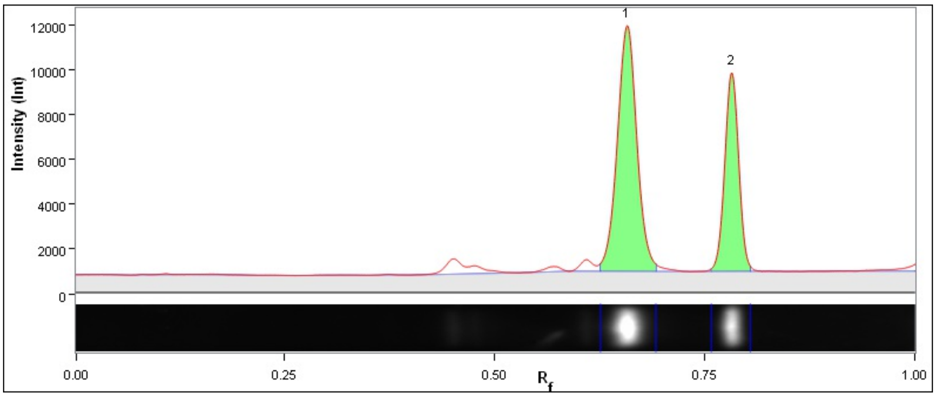

Band No.	Band Label	Mol. Wt. (KDa)	Relative Front	Adj. Volume (Int)	Volume (Int)	Abs. Quant.	Rel. Quant.	Band %	Lane %
1		N/A	0.658	80,439,540	97,398,944	N/A	N/A	63.5	56.7
2		N/A	0.782	46,321,636	58,315,776	N/A	N/A	36.5	32.6

Band Detection	Automatically detected bands with sensitivity: Low
Lane Background	Lane background subtracted with disk size: 7
Lane Width	7.00 mm

Lane 5

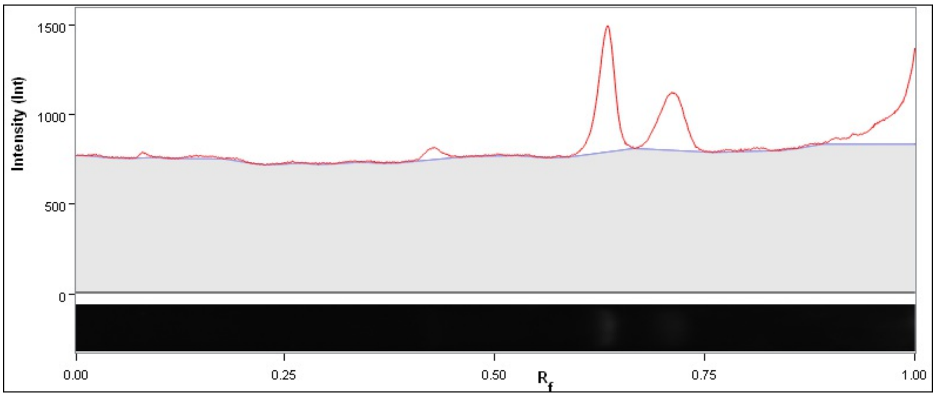

Band No.	Band Label	Mol. Wt. (KDa)	Relative Front	Adj. Volume (Int)	Volume (Int)	Abs. Quant.	Rel. Quant.	Band %	Lane %

Band Detection	Automatically detected bands with sensitivity: Low
Lane Background	Lane background subtracted with disk size: 7
Lane Width	7.00 mm

Lane 6

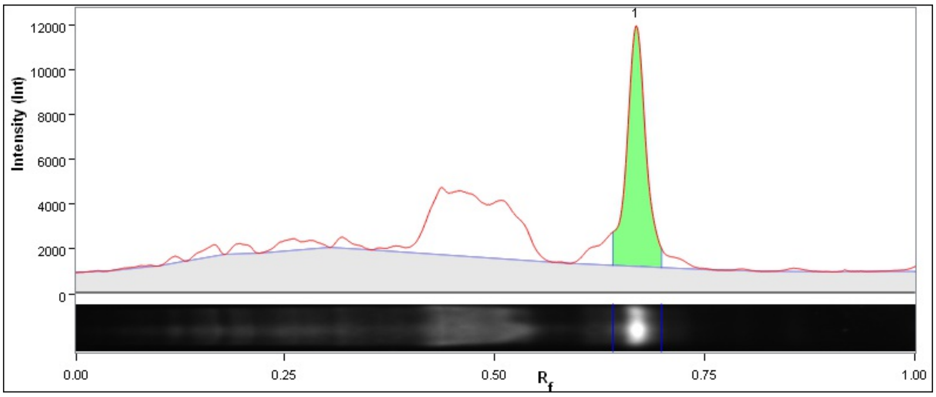

Band No.	Band Label	Mol. Wt. (KDa)	Relative Front	Adj. Volume (Int)	Volume (Int)	Abs. Quant.	Rel. Quant.	Band %	Lane %
1		N/A	0.668	70,694,168	87,575,180	N/A	N/A	100.0	38.1

Band Detection	Automatically detected bands with sensitivity: Low
Lane Background	Lane background subtracted with disk size: 7
Lane Width	7.00 mm

Lane 7

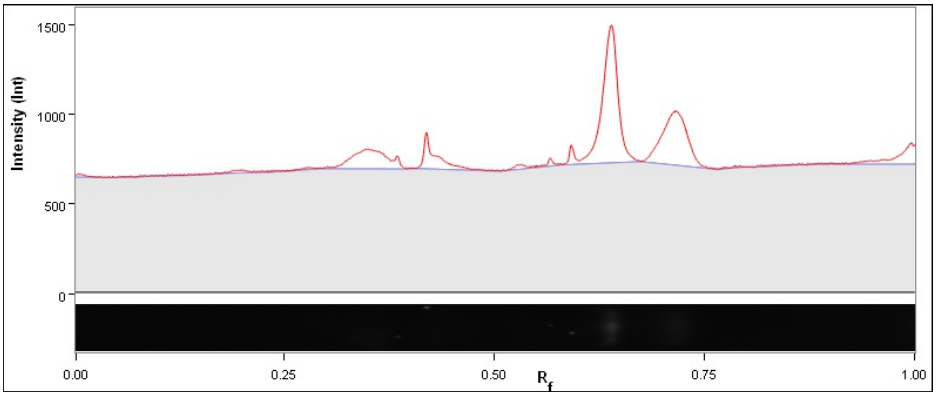

Band No.	Band Label	Mol. Wt. (KDa)	Relative Front	Adj. Volume (Int)	Volume (Int)	Abs. Quant.	Rel. Quant.	Band %	Lane %

Band Detection	Automatically detected bands with sensitivity: Low
Lane Background	Lane background subtracted with disk size: 7
Lane Width	7.00 mm

Lane 8

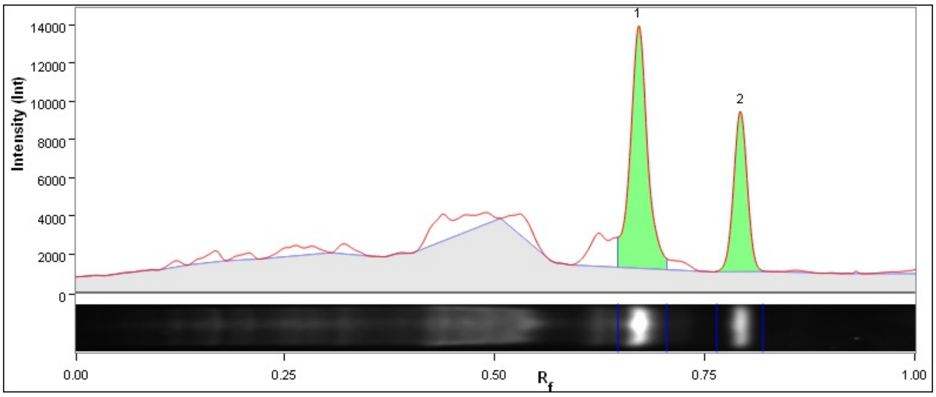

Band No.	Band Label	Mol. Wt. (KDa)	Relative Front	Adj. Volume (Int)	Volume (Int)	Abs. Quant.	Rel. Quant.	Band %	Lane %
1		N/A	0.672	77,819,968	96,745,240	N/A	N/A	65.3	42.2
2		N/A	0.793	41,278,308	56,829,116	N/A	N/A	34.7	22.4

Band Detection	Automatically detected bands with sensitivity: Low
Lane Background	Lane background subtracted with disk size: 7
Lane Width	7.00 mm

Lane 9

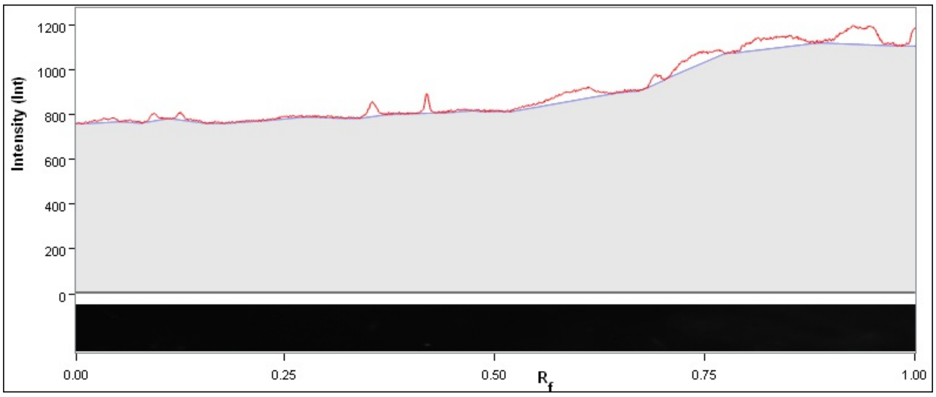

Band No.	Band Label	Mol. Wt. (KDa)	Relative Front	Adj. Volume (Int)	Volume (Int)	Abs. Quant.	Rel. Quant.	Band %	Lane %

Band Detection	Automatically detected bands with sensitivity: Low
Lane Background	Lane background subtracted with disk size: 7
Lane Width	7.00 mm

Lane 10

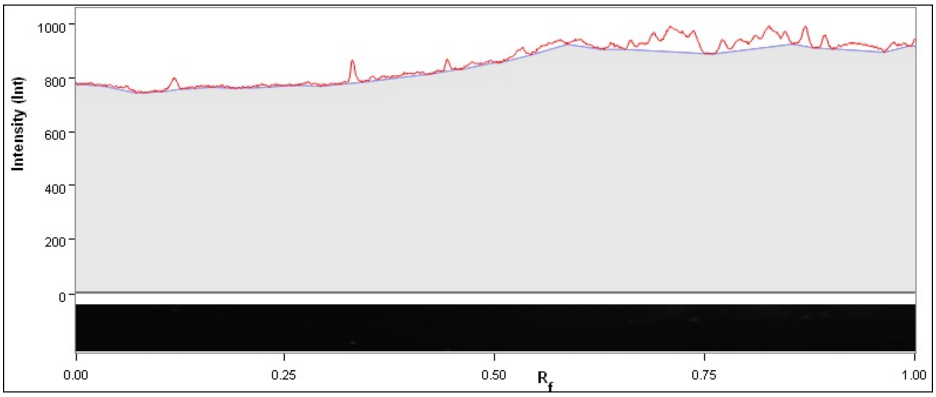

Band No.	Band Label	Mol. Wt. (KDa)	Relative Front	Adj. Volume (Int)	Volume (Int)	Abs. Quant.	Rel. Quant.	Band %	Lane %

Band Detection	Automatically detected bands with sensitivity: Low
Lane Background	Lane background subtracted with disk size: 7
Lane Width	7.00 mm

7

References

[1] A. D. Becke, "Density-functional exchange-energy approximation with correct asymptotic behavior," *Physical Review A*, vol. 38, no. 6, pp. 3098–3100, 1988.

[2] C. Lee, E. Yang, and R. G. Parr, "Development of the Colic-Salvetti correlation-energy formula into a functional of the electron density," *Physical Review B*, vol. 37, no. 2, pp. 785–9, 1988.

[3] P. M. Gill, B. G. Johnson, J. A. Pople, and M. J. Frisch, "The performance of the Becke—Lee—Yang—Parr (B—LYP) density functional theory with various basis sets," *Chemical Physics Letters*, vol. 197, no. 4-5, pp. 499–505, 1992.

[4] M. J. Frisch, G. W. Trucks, H. B. Schlegel, G. E. Scuseria, M. A. Robb, J. R. Cheeseman, J. J. A. Montgomery, T. Vreven, K. N. Kudin, J. C. Burant, J. M. Millam, S. S. Iyengar, J. Tomasi, V. Barone, B. Mennucci, M. Cossi, G. Scalmani, N. Rega, G. A. Petersson, H. Nakatsuji, M. Hada, M. Ehara, K. Toyota, R. Fukuda, J. Hasegawa, M. Ishida, T. Nakajima, Y. Honda, O. Kitao, H. Nakai, M. Klene, X. Li, J. E. Knox, H. P. Hratchian, J. B. Cross, V. Bakken, C. Adamo, J. Jaramillo, R. Gomperts, R. E. Stratmann, O. Yazyev, A. J. Austin, R. Cammi, C. Pomelli, J. W. Ochterski, P. Y. Ayala, K. Morokuma, G. A. Voth, P. Salvador, J. J. Dannenberg, V. G. Zakrzewski, S. Dapprich, A. D. Daniels, M. C. Strain, O. Farkas, D. K. Malick, A. D. Rabuck, K. Raghavachari, J. B. Foresman, J. V. Ortiz, Q. Cui, A. G. Baboul, S. Clifford, J. Cioslowski, B. B. Stefanov, G. Liu, A. Liashenko, P. Piskorz, I. Komaromi, R. L. Martin, D. J. Fox, T. Keith, M. A. Al-Laham, C. Y. Peng, A. Nanayakkara, M. Challacombe, P. M. W. Gill, B. Johnson, W. Chen, M. W. Wong, C. Gonzalez, and J. A. Pople, "Gaussian 03," 2004.

[5] C. Peng, P. Y. Ayala, H. B. Schlegel, and M. J. Frisch, "Using redundant internal coordinates to optimize equilibrium geometries and transition states," *Journal of Computational Chemistry*, vol. 17, no. 1, pp. 49–56, 1996.

[6] C. Gonzalez and H. B. Schlegel, "Reaction path following in mass-weighted internal coordinates," *The Journal of Physical Chemistry*, vol. 94, no. 14, pp. 5523–5527, 1990.

[7] B. Bernardim, P. M. Cal, M. J. Matos, B. L. Oliveira, N. Martínez-Sáez, I. S. Albuquerque, E. Perkins, F. Corzana, A. C. Burtoloso, G. Jiménez-Osés, and G. J. L. Bernardes, "Stoichiometric and irreversible cysteine-selective protein modification using carbonylacrylic reagents," *Nature Communications*, vol. 7, no. 1, p. 13128, 2016.

[8] H. Inoue, H. Nojima, and H. Okayama, "High efficiency transformation of Escherichia coli with plasmids," *Gene*, vol. 96, no. 1, pp. 23–28, 1990.

[9] A. Rosace, A. Bennett, M. Oeller, M. M. Mortensen, L. Sakhnini, N. Lorenzen, C. Poulsen, and P. Sormanni, "Automated optimisation of solubility and conforma-

tional stability of antibodies and proteins," *Nature Communications*, vol. 14, no. 1, p. 1937, 2023.

[10] H. Liu and J. H. Naismith, "An efficient one-step site-directed deletion, insertion, single and multiple-site plasmid mutagenesis protocol," *BMC Biotechnology*, vol. 8, no. 1, p. 91, 2008.

[11] G. Esposito, R. Michelutti, G. Verdone, P. Viglino, H. Hernández, C. V. Robinson, A. Amoresano, F. D. Piaz, M. Monti, P. Pucci, P. Mangione, M. Stoppini, G. Merlini, G. Ferri, and V. Bellotti, "Removal of the N-terminal hexapeptide from human β2-microglobulin facilitates protein aggregation and fibril formation," *Protein Science*, vol. 9, no. 5, pp. 831–845, 2000.

[12] A. Ramon, M. Ali, M. Atkinson, A. Saturnino, K. Didi, C. Visentin, S. Ricagno, X. Xu, M. Greenig, and P. Sormanni, "Assessing antibody and nanobody nativeness for hit selection and humanization with AbNatiV," *Nature Machine Intelligence*, vol. 6, no. 1, pp. 74–91, 2024.

[13] Y. Y. Lau, H. Zhai, and L. L. Schafer, "Catalytic Asymmetric Synthesis of Morpholines. Using Mechanistic Insights To Realize the Enantioselective Synthesis of Piperazines," *The Journal of Organic Chemistry*, vol. 81, no. 19, pp. 8696–8709, 2016.

[14] H. Miyabe, E. Yoshioka, I. Minato, and H. Takashima, "Synthesis of Oxygen-Heterocycles Having Linker Components for Trapping Cysteine Derivatives," *HETEROCYCLES*, vol. 101, no. 1, p. 263, 2020.

[15] M. Badertscher, P. Bühlmann, and E. Pretsch, *Structure Determination of Organic Compounds*. Springer-Verlag, 2009.

[16] M. T. Marty, A. J. Baldwin, E. G. Marklund, G. K. Hochberg, J. L. Benesch, and C. V. Robinson, "Bayesian deconvolution of mass and ion mobility spectra: From binary interactions to polydisperse ensembles," *Analytical Chemistry*, vol. 87, no. 8, pp. 4370–4376, 2015.
